# Severe Acute Respiratory Syndrome Coronavirus 2 Transmission in Georgia, USA, February 1–July 13, 2020

**DOI:** 10.3201/eid2710.210061

**Published:** 2021-10

**Authors:** Yuke Wang, Casey Siesel, Yangping Chen, Ben Lopman, Laura Edison, Michael Thomas, Carly Adams, Max Lau, Peter F.M. Teunis

**Affiliations:** Rollins School of Public Health at Emory University, Atlanta, Georgia, USA (Y. Wang, C. Siesel, Y. Chen, B. Lopman, C. Adams, M. Lau, P.F.M. Teunis);; Centers for Disease Control and Prevention, Atlanta (L. Edison);; Georgia Department of Public Health, Atlanta (L. Edison, M. Thomas)

**Keywords:** 2019 novel coronavirus disease, coronavirus disease, COVID-19, severe acute respiratory syndrome coronavirus 2, SARS-CoV-2, viruses, respiratory infections, zoonoses, transmission, serial interval, reproduction number, shelter-in-place, Georgia, United States

## Abstract

The serial interval and effective reproduction number for coronavirus disease (COVID-19) are heterogenous, varying by demographic characteristics, region, and period. During February 1–July 13, 2020, we identified 4,080 transmission pairs in Georgia, USA, by using contact tracing information from COVID-19 cases reported to the Georgia Department of Public Health. We examined how various transmission characteristics were affected by symptoms, demographics, and period (during shelter-in-place and after subsequent reopening) and estimated the time course of reproduction numbers for all 159 Georgia counties. Transmission varied by time and place but also by persons’ sex and race. The mean serial interval decreased from 5.97 days in February–April to 4.40 days in June–July. Younger adults (20–50 years of age) were involved in most transmission events occurring during or after reopening. The shelter-in-place period was not long enough to prevent sustained virus transmission in densely populated urban areas connected by major transportation links.

Coronavirus disease (COVID-19) is an infectious disease caused by severe acute respiratory syndrome coronavirus 2 (SARS-CoV-2). After it was first reported in Wuhan, China, in December 2019, COVID-19 spread rapidly across the world as an ongoing global pandemic. As of July 9, 2021, most confirmed COVID-19 cases (33,792,898 cases) and deaths (606,487) in the world were in the United States (*1*), and 906,136 confirmed cases and 18,544 deaths were in the state of Georgia (*2*).

Transmission of COVID-19 varies by region (*3*,*4*), setting (long-term care facilities, prisons, and factories) (*5*), population demographics (age, sex, and race), and even among individual persons (physiologic and behavioral differences) (*6*). During the early phases of transmission in the United States, new cases were mainly imported by travelers and transmission was associated with human mobility (*7*). Local transmission was more intense in regions with high population density and in populations with frequent social contacts (*3*,*8*,*9*). When SARS-CoV-2 was introduced into high-risk settings (e.g., long-term care facilities), transmission rates were intense, and the outcomes were often fatal (*10*).

To study transmission of SARS-CoV-2, we examined the serial interval for symptom onset (defined as the time interval between symptom onset in a primary case-patient and symptom onset in a secondary case-patient infected by the primary case-patient) and the effective reproduction number R_t_ (the expected number of cases directly caused by any single infectious person). R_t_ has been shown to vary strongly; some case-patients have caused superspreading events (*11*,*12*). Such heterogeneity influences the spread as well as the control of COVID-19, as documented by studies of nonpharmaceutical interventions in China (*13*,*14*) and Europe (*15*) at the province and country levels.

After the first case of COVID-19 was reported in the state of Georgia on March 2, 2020, a series of events and interventions followed (Appendix Table 1). On April 3, state officials announced a shelter-in-place order, requiring all residents and visitors to remain in their residence and take every possible precaution to limit social interactions. On April 24, officials allowed some businesses to reopen, and on April 30 the shelter-in-place order was lifted. On June 1, state officials further relaxed restrictions. During June–July 2020, as new COVID-19 cases continued to surge in Georgia and other states, knowing how shelter-in-place and the subsequent reopening events affected the transmission of SARS-CoV-2 in different regions became crucial.

Identifying a large number of the primary and secondary case-patient pairs enabled us to estimate the distribution of the serial interval for symptom onset. Using the serial interval distribution, we can estimate the time-varying R_t_ (*16*). With R_t_s over time, we can study the spatial distribution of transmission across all 159 Georgia counties as well as the effects of shelter-in-place and subsequent gradual reopening.

The Georgia Department of Public Health (GDPH) Institutional Review Board determined that this analysis was exempt from the requirement for review and approval, and informed consent was not required. This activity was reviewed by the Centers for Disease Control and Prevention and was consistent with their applicable policy and with federal law.

## Methods

### Data Source

GDPH provided data for all 118,491 confirmed COVID-19 cases in all 159 counties of Georgia during February 1–July 13, 2020. Available data included demographic characteristics (age, sex, and race), clinical characteristics (dates of symptom onset, recorded symptoms, hospitalization, and ventilator use), and social contacts (contacts between confirmed case-patients and if cases were part of a confirmed outbreak) (Table; Appendix Table 2). Missing values in the data were common; large percentages of values for clinical characteristics were missing. With regard to events possibly driving transmission, periods were categorized as early transmission and shelter-in-place during February–April, after reopening (shelter-in-place order was lifted) in May, and further reopening (more restrictions were relaxed) during June–July (Appendix Table 1). For this study, we defined a COVID-19 case as SARS-CoV-2 infection confirmed by reverse transcription PCR irrespective of clinical signs and symptoms.

**Table:** Demographic and clinical information for persons with confirmed coronavirus disease, Georgia, USA, during 3 periods, February 1–July 15, 2020

Variable	February–April, no. (%), n = 31,575*	May, no. (%), n = 19,270†	June–July, no. (%), n = 67,646‡	Total, no. (%), n = 118,491§
Sex				
M	13,770 (43.6)	9,142 (47.4)	30,247 (44.7)	53,159 (44.9)
F	17,308 (54.8)	9,747 (50.6)	33,828 (50)	60,883 (51.4)
Missing	497 (1.6)	381 (2.0)	3,571 (5.3)	4,449 (3.7)
Race				
Black	13,010 (41.2)	4,639 (24.1)	13,878 (20.5)	31,527 (26.6)
White	11,418 (36.2)	7,168 (37.2)	17,500 (25.9)	36,086 (30.5)
Other	2,818 (8.9)	2,029 (10.5)	6,158 (9.1)	11,005 (9.3)
Missing	4,329 (13.7)	5,434 (28.2)	30,110 (44.5)	39,873 (33.6)
Hospitalized				
Yes	6,714 (21.3)	2,099 (10.9)	4,523 (6.7)	13,336 (11.3)
No	15,627 (49.5)	10,729 (55.7)	27,926 (41.3)	54,282 (45.8)
Missing	9,234 (29.2)	6,442 (33.4)	35,197 (52)	50,873 (42.9)
Ventilator use				
Yes	1,046 (3.3)	184 (1.0)	258 (0.4)	1,488 (1.3)
No	12,188 (38.6)	8,404 (43.6)	19,313 (28.6)	39,905 (33.7)
Missing	18,341 (58.1)	10,682 (55.4)	48,075 (71.1)	77,098 (65.0)
Abnormal chest radiograph finding				
Yes	2,602 (8.2)	494 (2.6)	742 (1.1)	3,838 (3.2)
No	10,151 (32.1)	8,081 (41.9)	18,246 (27.0)	36,478 (30.8)
Missing	18,822 (59.6)	10,695 (55.5)	48,658 (71.9)	78,175 (66.0)
Death				
Yes	2,127 (6.7)	558 (2.9)	320 (0.5)	3,005 (2.5)
No	15,766 (49.9)	10,183 (52.8)	26,304 (38.9)	52,253 (44.1)
Missing	13,682 (43.3)	8,529 (44.3)	41,022 (60.6)	63,233 (53.4)
Fever				
Yes	10,094 (32.0)	4,005 (20.8)	11,787 (17.4)	25,886 (21.8)
No	8,489 (26.9)	7,951 (41.3)	19,655 (29.1)	36,095 (30.5)
Missing	12,992 (41.1)	7,314 (38)	36,204 (53.5)	56,510 (47.7)
Cough				
Yes	12,417 (39.3)	4,992 (25.9)	15,319 (22.6)	32,728 (27.6)
No	6,462 (20.5)	7,059 (36.6)	16,434 (24.3)	29,955 (25.3)
Missing	12,696 (40.2)	7,219 (37.5)	35,893 (53.1)	55,808 (47.1)
Shortness of breath				
Yes	8,504 (26.9)	2,952 (15.3)	7,325 (10.8)	18,781 (15.9)
No	9,807 (31.1)	8,960 (46.5)	23,542 (34.8)	42,309 (35.7)
Missing	13,264 (42)	7,358 (38.2)	36,779 (54.4)	57,401 (48.4)
Diarrhea				
Yes	4,410 (14)	1,971 (10.2)	6,072 (9.0)	12,453 (10.5)
No	12,718 (40.3)	9,589 (49.8)	23,936 (35.4)	46,243 (39.0)
Missing	14,447 (45.8)	7,710 (40.0)	37,638 (55.6)	59,795 (50.5)

*Median age (Q1–Q3) 51 (37­–65) y. †Median age (Q1–Q3) 43 (28–59) y. ‡Median age (Q1–Q3) 34 (23–50) y. §Median age (Q1–Q3) 40 (26–56) y.

### Tracked Pairs: Serial Intervals and Characteristics of Transmission

On the basis of reported contacts with confirmed case-patients, we identified pairs of primary and secondary case-patients by using the following procedure. First, most transmission pairs could be established as a unique close contact with a confirmed case-patient. We assumed that symptom onset for any primary case-patient in a confirmed pair occurred before symptom onset of the secondary case-patient. Second, when an outbreak involved multiple cases, we assigned primary case-patients according to review of the epidemiologic time lines. Usually, there was 1 case-patient whose symptom onset was several days earlier than that of the rest of the case-patients in the cluster, and this case-patient was designated as the primary case-patient. Thus, serial intervals were assumed to be always positive. To examine the influence of ignoring negative serial intervals on R_t_ estimation, we conducted a sensitivity analysis (Appendix Supplemental Material C). Transmission pairs with serial intervals >15 days were dropped because such long intervals are unlikely, as shown in previous studies (*17*,*18*). We modeled the serial interval as a gamma distribution and obtained maximum-likelihood estimators of shape and scale parameters. Furthermore, we explored whether the duration of the serial interval varied by demographic characteristics, various disease symptoms, and periods of symptom onset for primary case-patients. The large numbers of tracked case-patient pairs also enabled us to examine variation in transmission within and between different groups by age, sex, and race.

### Confirmed Cases: Reproduction Numbers

We estimated probabilities of transmission between any pairs of case-patients in an outbreak by using a transmission probability matrix method (Appendix Supplemental Material B) (*16*). Using GDPH data for confirmed COVID-19 cases during February 1–July 13, 2020, we estimated R_t_s by date, and we used dates of symptom onset and social contact information (wherever available) in each county independently by estimating the transmission probability matrix.

Among 118,491 confirmed cases, the date of symptom onset was missing for 48,893 (41.3%). These missing symptom-onset dates were imputed according to dates of first specimen collection if available or dates of laboratory report if not (Appendix Supplemental Material A).

The most recent data are incomplete because not all incident cases have been reported and not all persons have become symptomatic. Therefore, estimates of R_t_ approaching the present date are biased. Because one of our study goals was to examine the timing and magnitude of the first 2 waves of SARS-CoV-2 transmission in Georgia (and not to nowcast transmission), we removed R_t_ estimates of the most recent 4 weeks (June 16–July 13) from the analysis. SARS-CoV-2 transmission in Georgia seemed to include multiple waves and varied considerably among counties. The time-varying average R_t_ estimates were smoothed by using LOESS regression, and local maximums/minimum were identified for each individual county. On the basis of our review of the epidemic curves and curves, we defined the following 5 transmission patterns:

- Consistent spreading: there was sustained transmission of SARS-CoV-2 (R_t_ >1) during the shelter-in-place period. Consequently, numbers of cases remained high and increased rapidly at reopening.
- Two strong waves: a first wave of early transmission was followed by a slowdown (R_t_ <1) during the shelter-in-place period and a new surge in cases (1 ≤ R_t_ <2) after reopening.
- Strong first wave: there was a considerable number of cases during the initial period of the outbreak. During the shelter-in-place period, spreading was controlled; after reopening no new surge in cases occurred (R_t_ <1).
- Strong second wave: there were few cases during the early transmission period, but new cases surged (R_t_ ≥2) after reopening.
- Small case number (<200): SARS-CoV-2 transmission was rare.

We generated maps to spatially examine the spreading of the COVID-19 first wave. We evaluated the effect of shelter-in-place, reopening, and further reopening by the trend of reproduction numbers before and after those events in different regions of Georgia.

## Results

### Tracked Pairs: Serial Intervals

**Figure 1:**
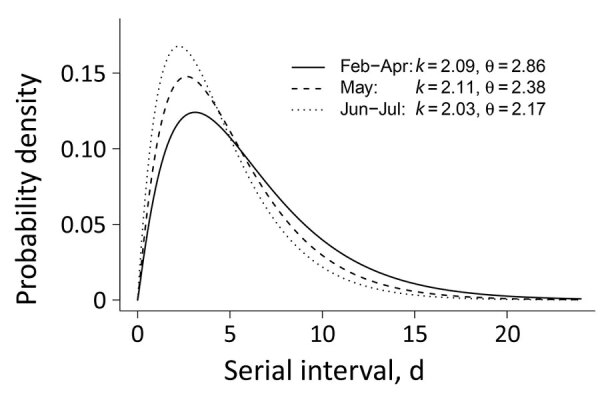
Estimated serial interval distribution for 3 periods in study of severe acute respiratory syndrome coronavirus 2 transmission in Georgia, USA: early transmission and shelter-in-place (February–April 2020); after reopening (May); and further reopening (June–July). *k* and θ indicate the scale and shape parameters for the gamma distribution. The y-axis represents the estimated probability density of having a certain serial interval.

On the basis of 4,080 tracked pairs of primary and linked secondary case-patients in Georgia (Appendix Table 3), we estimated the serial interval distribution as a gamma distribution with a mean (10th–90th percentile) of 4.99 (1.32–9.71) days. Generally, the serial interval was longer when outcomes for primary case-patients were severe, such as hospitalization, undergoing ventilation, having an abnormal chest radiograph result, or death as final outcome (Appendix Table 4). Specific signs/symptoms in primary case-patients (i.e., fever, cough, shortness of breath, or diarrhea) did not shorten serial intervals. Serial intervals did not differ across demographic categories (i.e., age, sex, race, or location). The mean (10th–90th percentile) serial interval was 5.97 (1.65–11.50) days in February–April, 5.03 (1.41–9.65) days in May, and 4.40 (1.18–8.52) days in June–July (Figure 1). The average serial interval became shorter over time: from 5.97 (1.65–11.50) days in February–April, to 5.03 (1.41–9.65) days in May, and then to 4.40 (1.18–8.52) days in June–July (Appendix Figure 1).

### Tracked Pairs: Characteristics of Transmission

**Figure 2:**
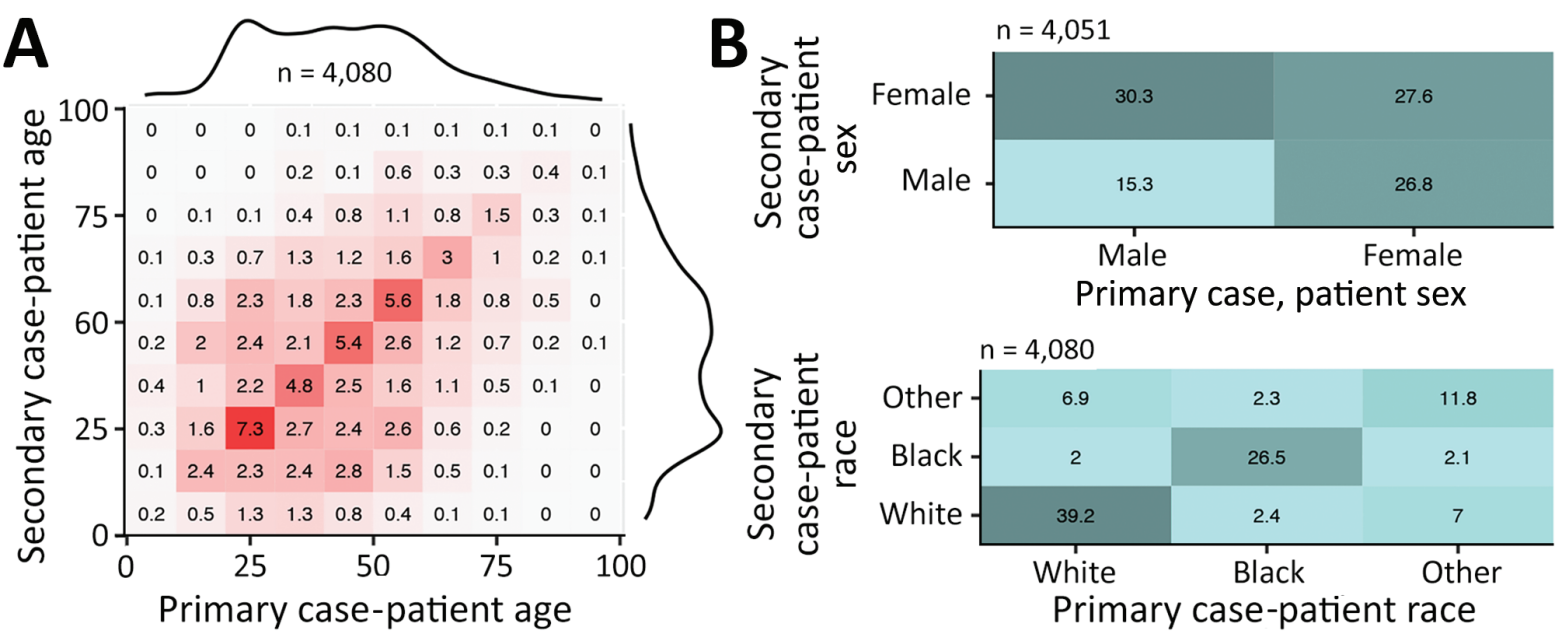
Patterns of severe acute respiratory syndrome coronavirus 2 transmission by patient age (A), sex (B), and race (B), based on 4,080 tracked pairs of coronavirus disease cases from Georgia, USA, during February–July 2020. The matrix graphs show numbers of transmission pairs as a percentage of the total, with primary case-patients as columns and their secondary case-patients as rows. Darker colors indicate a higher percentage of fraction of tracked pairs observed. In panel A, marginal totals are shown as density curves to illustrate the age distribution of case-patients.

To study the variation in transmission by demographic characteristic (i.e., age, sex, and race), the observed frequencies in transmission pairs can be shown in a matrix (Figure 2, panels A, B). Male case-patients were twice as likely to transmit infection to a female than a male contact, whereas female case-patients were equally likely to transmit infection to a male or a female contact. Transmission between races was strongly assortative. White and Black persons were more likely to transmit infection to persons of their own races than to persons of other races; White persons were 4.4 times as likely to transmit infection to White persons, and Black persons were 5.6 times as likely to transmit infection to Black persons.

**Figure 3:**
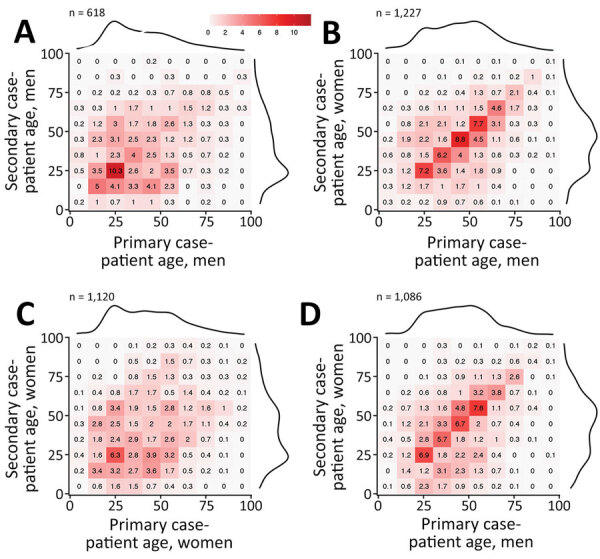
Patterns of severe acute respiratory syndrome coronavirus 2 transmission according to patient sex and age, based on 4,080 tracked pairs of coronavirus disease cases in Georgia, USA, February–July 2020. A) Male-to-male transmission; B) male-to-female transmission; C) female-to-female transmission; D) female-to-male transmission. The matrix graphs show numbers of transmission pairs as a percentage of the total, with primary case-patients as columns and their secondary case-patients as rows. Darker colors indicate a higher percentage of fraction of tracked pairs observed. Marginal totals are shown as density curves to illustrate the age distribution of case-patients.

**Figure 4:**
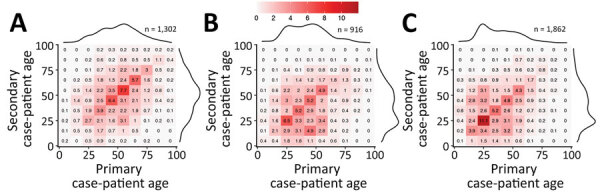
Patterns of severe acute respiratory syndrome coronavirus 2 transmission in Georgia, USA, February–July 2020, by age group, in 3 successive periods. A) Early transmission and shelter-in-place (February–April); B) after reopening (May); C) further reopening (June–July). The matrix graphs show numbers of transmission pairs as a percentage of the total, with primary case-patients as columns and their secondary case-patients as rows. Darker colors indicate a higher percentage of fraction of tracked pairs observed. Marginal totals are shown as density curves to illustrate the age distribution of case-patients.

SARS-CoV-2 seemed to mainly spread from adults 20–60 years of age during February–July 2020; transmission between children (<20 years) and elderly persons (>60 years) was observed less often, suggesting that transmission occurred more frequently between persons of similar ages (Figure 3, panels A–D). Transmission between persons of different sexes was mainly among those in the same age group. Cases in persons 10–30 years of age were associated with most transmission pairs of the same sex. Over the study period, most transmission pairs shifted from 40–70 years of age (median age for primary case-patients was 52 years and for secondary case-patients was 50 years) in February–April to 20–50 years of age (primary case-patient median age 36 years and secondary case-patient median age 34 years) in June–July (Figure 4).

### Temporal and Spatial Patterns of Transmission

**Figure 5:**
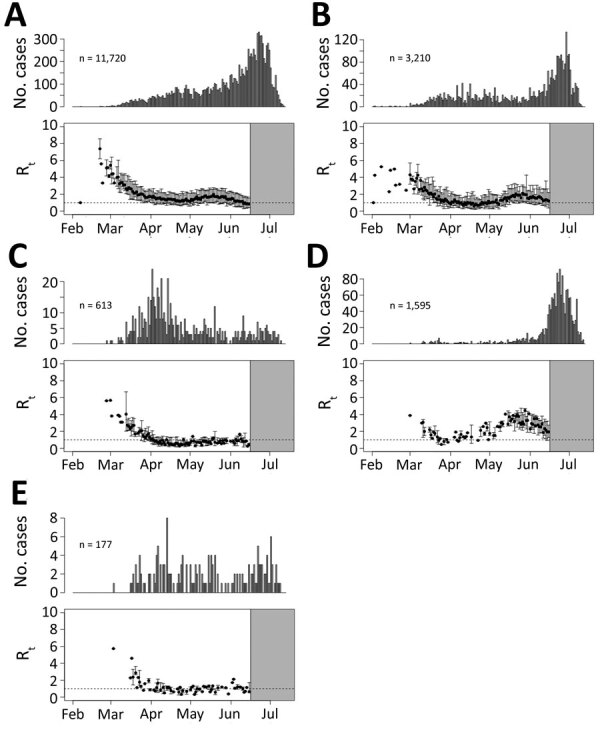
Examples of the 5 categories of severe acute respiratory syndrome coronavirus 2 spreading patterns in counties in Georgia, USA, February–July 2020. Shown are epidemic curves from the start of the outbreak until July 13, 2020, and effective reproduction number (R_t_) estimates until June 15, in Gwinnett (A), Clayton (B), Sumter (C), Glynn (D), and Dawson (E) Counties. Tick marks indicate the first day of the month. The x-axis represents the date of symptom onset for patients with confirmed cases. The y-axis in the top plot shows the number of cases; the y-axis in the bottom plot shows the estimated median reproduction numbers. Error bars represent 2.5th–97.5th percentile ranges of R_t_s. The gray area shows where R_t_ estimates were truncated on June 15.

During February and March, R_t_s were >1 and then decreased until late April and early May, considered the first wave in Georgia. R_t_ usually decreased to a (mathematical) local minimum during the shelter-in-place period and started to increase again as the second wave began. As during the first wave, R_t_s peaked and then started to decrease again during the second wave (Figure 5). Although the number of reported cases was lower in first wave, R_t_ was much higher in the first wave (≈3.5) than in the second wave (≈1.7).

**Figure 6:**
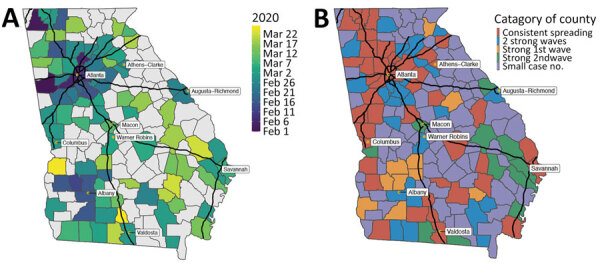
Spatial patterns of transmission of severe acute respiratory syndrome coronavirus 2 in Georgia, USA, February–July 2020. A) Date of reaching the peak (local maximum of effective reproduction number) for the first wave; B) spatial distribution of the 5 categories of virus transmission patterns by June 15, 2020. The black lines represent interstate highways.

**Figure 7:**
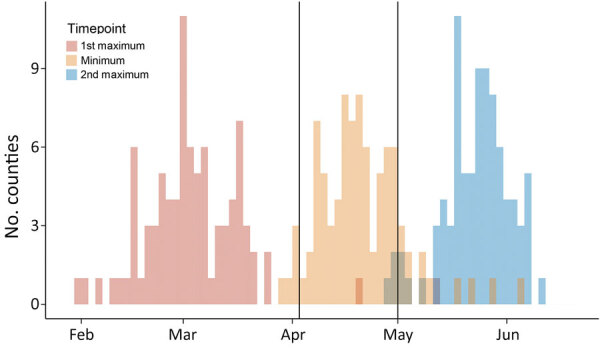
Distributions of estimated dates of first maximum, minimum, and second maximum in effective reproduction numbers for severe acute respiratory syndrome coronavirus 2 transmission in 87 counties in Georgia, USA, with 200 cumulative cases by July 13, 2020, and dates of key events possibly driving virus transmission.

Although the general pattern of SARS-CoV-2 transmission was similar across all counties, the dates of local maximums/minimum (i.e., first peak, local minimum, and second peak) and the magnitude of R_t_ at these extremes varied among counties. The peak dates for the first wave in counties with cumulative case numbers was >200 cases by July 13, 2020 (Figure 6, panel A). At that time, counties with high numbers of COVID-19 cases were located around cities and along highways. Starting in early February, COVID-19 spread radially and along the interstate highway from Atlanta and Albany, the 2 initial outbreak sources. Outbreaks occurred later in other cities, including Augusta and Savannah. A total of 65 (74.7%) of 87 counties with >200 cumulative cases by July 13th reached a local minimum in R_t_ during the shelter-in-place period (April 3–April 30) (Figure 7). After reopening, many counties experienced a second wave of COVID-19 and increased numbers of cases were reported. On the basis of the magnitude of R_t_ at the first peak, local minimum, and second peak, we categorized case data into the 5 transmission patterns (Figure 5; Appendix Figures 11–169).

Consistent spreading occurred in Georgia counties around some major cities (e.g., Atlanta, Athens, Columbus, Savannah) and counties along interstate highways (Figure 6, panel B). In counties bordering other counties with consistent spreading, there were 2 strong waves or only a strong second wave. An early intense first wave but not a strong second wave occurred in counties around the city of Albany (Lee, Sumter, Terrell, Mitchell, Crisp, and Dooly Counties). Fewer cases occurred in counties not connected by interstate highways.

## Discussion

During February–July, the estimated serial intervals for onset of COVID-19 symptoms in the state of Georgia seemed to become shorter (Figure 1). Such a phenomenon was also observed in mainland China during January–February 2020 (*19*). Shorter serial intervals imply more rapid transmission. During February–July, disease prevalence increased in Georgia; by August 25, Georgia had the fifth highest number of confirmed COVID-19 cases in the United States. One cause of contracting serial intervals could be that persons had more contacts after reopening; in particular, younger persons (20–50 years) might play a larger role in SARS-CoV-2 transmission. Also, Kenah et al. showed that increasingly more infectious case-patients are present in the local population, competing to infect susceptible persons, and the expected time until a new infection is shortened (*20*).

The serial interval estimation could also be affected by changing testing practices and contact tracing over the duration of the pandemic. COVID-19 testing capacity and contact tracing ability in Georgia were limited during earlier stages of the pandemic; thus, identification and isolation of COVID-19 case-patients and their close contacts were often delayed. With improved testing capacity, symptomatic case-patients were tested more promptly and isolated more quickly, which led to fewer exposures during their infectious periods. Rapid isolation and contact tracing could truncate transmission and lead to shorten serial intervals. More recent data collected when testing and contact tracing have improved are less likely to be affected by delayed testing and isolation. Contraction of serial intervals continued into May through early July, so the changes may still be explained at least partly by increased prevalence and increased contact rates (Figure 1).

Transmission of a respiratory infection such as COVID-19 depends on behavioral factors and in particular on social contacts. Studies of contact behavior have shown that persons tend to have social contact with peers of similar age and demographic backgrounds (*21*). The tracked transmission pairs in this study show that such assortative mixing also applies to SARS-CoV-2 transmission (Figure 2). The transmission pairs in this study were more likely to be tracked when case-patients knew each other (e.g., family members, friends, or colleagues), whereas transmission in public spaces (e.g., stores or restaurants) usually could not be tracked. Transmission occurs frequently among persons in the same age group and less frequently among those in different age groups (Figure 3), although transmission may have been across generations (e.g., between parents and children, or grandparents and grandchildren) (*22*).

A primary case-patient who was male was more likely to transmit infection to a female contact than to a male contact. Female case-patients were infected by male case-patients across a wide range of ages (Figure 3, panel B), and male case-patients were mainly infected by young male case-patients (Figure 3, panel A). A possible explanation may be that female persons tend to be caregivers, taking care of sick persons in the household, and young male persons may be more likely to acquire infection outside the household.

Similar to the serial interval, transmission patterns also changed as the pandemic continued. The major contribution to spreading SARS-CoV-2 shifted over time to the younger generation. This shift could be caused by elderly persons becoming more careful to protect themselves from infection by taking measures such as staying at home, wearing face masks in public spaces, and observing good hand hygiene. At the same time, younger persons might have been less compliant with quarantine measures and more likely to attend indoor gatherings such as parties or to have visited bars, gyms, and clubs while not wearing face masks.

Previous pandemics, such as the 1918 influenza and the 2009 swine influenza (H1N1) pandemics, caused multiple waves of infections (*23*). In Georgia, we have so far observed 2 waves of SARS-CoV-2 transmission separated by the shelter-in-place period. The COVID-19 cases of the first wave were first observed in Atlanta, the state capital with one of the busiest US airports, and Albany, the eighth largest city in Georgia. The outbreak in Albany resulted from 2 superspreading funeral events. However, the connectivity of these 2 cities differs: Atlanta is a transportation hub that connects multiple interstate highways, whereas Albany has no interstate highways. During the first wave, SARS-CoV-2 spread radially from both cities to the surrounding areas. For Atlanta, cases also started to appear along the interstate highways (Figure 6, panel A). Concentrations of increased transmission along highways, as links connecting population centers, suggest that commuter links might have been effective transmission links.

During the shelter-in-place period (April 3–April 30), SARS-CoV-2 transmission slowed and R_t_s reached a local minimum in most counties. However, before reopening, R_t_s were still >1 in many counties even at the local minimum, indicating continued disease spread (Figure 6, panel B). After reopening, transmission again increased across Georgia. These data suggest that the 3 or 4 weeks of shelter-in-place orders were not long enough to sufficiently suppress SARS-CoV-2 transmission (local and imported) in densely populated urban areas connected by major transportation links.

Thus far, the second wave has been heterogenous in time and magnitude in different counties. Local prevalence was different at the time of reopening, and counties where prevalence was high (i.e., counties bordering cities and along interstate highways) experienced a stronger second wave. Counties not connected by major transportation links (e.g., around Albany) often also saw a second wave of COVID-19 but on a relatively small scale. Some counties that experienced an early and intense first wave (e.g., Lee, Sumter, Terrell, and Mitchell) did not experience a second wave. Possibly, inhabitants of those counties were more compliant with the prevention and control measures.

A limitation of our study is that although data were available for >100,000 cases, clinical information and contacts with a confirmed case-patient were missing on some records. Absence of clinical information may depend on several factors. For example, reporting rates tend to be lower and clinical information more frequently missing for case-patients with mild or no symptoms than for case-patients with severe symptoms. A subgroup analysis showed similar distributions of serial intervals for transmission pairs with complete clinical information and transmission pairs with missing clinical information. This finding lends credibility to the assumption that the absence of clinical information does not affect the overall serial interval.

Data on tracked pairs were not missing at random because contact tracing is voluntary and its capacity was limited during the early stages of the pandemic. Tracked pairs were more likely to be recorded when they involved known contacts. Identifying transmission links in public spaces or within clusters of cases remains challenging.

In this study, presymptomatic transmission leading to negative serial intervals was ignored because infectors could rarely be determined by exposure information or travel history. On the basis of the sensitivity analysis (Appendix Supplemental Material C), the influence of a small proportion of negative serial intervals on R_t_ estimates could safely be ignored.

When examining the time course of transmission of SARS-CoV-2 in Georgia, asymptomatic transmission was ignored. The observed numbers of case-patients thus underestimate the numbers of infected (infectious) persons, but this underestimation does not imply that R_t_ is underestimated by the same amount. Both the numbers of primary case-patients (transmitting infection) and the numbers of secondary case-patients (acquiring infection) are underestimated, so the estimated rate of increase is likely to be less affected (*24*).

In conclusion, transmission of SARS-CoV-2 in Georgia changed over time during February–July 2020. The mean serial interval decreased from 5.97 days in February–April to 4.40 days in June–July. The younger population (20–50 years of age) was involved in most transmission events during or after reopening subsequent to the shelter-in-place period. By mid-July, 2 waves of SARS-CoV-2 transmission were apparent, separated by the shelter-in-place period in Georgia. Transmission was more intense in counties around major cities and along interstate highways. These transmission patterns can be used to help predict and guide states in COVID-19 prevention and control according to population and region.

Appendix Supplemental results for study of severe acute respiratory syndrome coronavirus 2 transmission in Georgia, USA, February 1–July 13, 2020.
