# Supplementary material for: Severe Acute Respiratory Syndrome Coronavirus 2 Transmission in Georgia, USA, February 1–July 13, 2020
Source: Emerg Infect Dis. 2021 Oct;27(10):2578–87. doi: 10.3201/eid2710.210061 (PMC8462336; doi:10.3201/eid2710.210061)
Supplement: Appendix — Supplemental results for study of severe acute respiratory syndrome coronavirus 2 transmission in Georgia, USA, February 1–July 13, 2020. [file 21-0061-Techapp-s1.pdf]

Article DOI: https://doi.org/10.3201/eid2710.210061

Severe Acute Respiratory Syndrome Coronavirus 2 Transmission in Georgia, USA, February 1–July 13, 2020

Appendix

Supplemental Material A. Imputation for Symptom Onset Dates

Among 118,497 confirmed cases between February 1–July 13, 2020, 48,893 (41.3%) cases had missing symptom onset dates. For each case, dates were also recorded when the first specimen was collected for testing and when the case was confirmed and reported. The delay of testing can be calculated as the difference between the date of first specimen collected and the date of symptom onset. And the delay of reporting can be calculated as the difference between the date of laboratory report and the date of symptom onset. Appendix Figure 2 (a) and (b) shows that the average delay of testing decreased gradually over time while the average delay of reporting was only stabilized after June 2020. We imputed the delay of testing (or the delay of reporting) using two negative binomial regression models with the date of first specimen collected or date of laboratory report as the predictor to account for decreasing trend of the delays. Missing symptom onset dates can be imputed based on the models and the date of first specimen collected (or date of laboratory report). Since the delay of testing was more stable compared to delay of reporting, the model with the date of first specimen collected as predictor was prioritized over the model with the date of laboratory report as predictor during the imputation. The procedure of imputation can be summarized as a flow chart (Appendix Figure 3). Appendix Figure 2 compares observed and imputed delays of testing and reporting.

To access the impact of imputing large proportion of missing symptom onset on the R_t estimation results, we repeated the analysis omitting cases with missing symptom onset in Clayton, Glynn, and Sumter counties. Appendix Figures 4–6 show the comparison of R_t estimation with and without imputed symptom onsets.

Supplemental Material B. Transmission Probability Matrix Method

Based on the transmission probability matrix method proposed by Teunis et al (*1*), we estimated probabilities of transmission between any pairs of case-patients in an outbreak. For an outbreak with n observed case-patients, a transmission probability matrix $\underset{n\times n}{V}$ can be defined with any element $v_{i,j}$ representing the probability that case-patient i was infected by case-patient j. When two cases are linked by their serial interval, the likelihood of transmission between these two case-patients can be calculated using the serial interval distribution as a kernel density (*1*). Additional information at an individual level (e.g., evidence of social contact between case-patients i and j) is accounted for by a $n \times n$ weighting matrix (*1*). The transmission probability matrix V can be estimated in a Markov chain Monte Carlo procedure (*1*,*2*).

When the transmission probability matrix is known, it can be used to calculate reproduction numbers. Elements of row i show the probabilities of case-patient i having received their infection from any other case-patient in the observed population. Rows of V must therefore add to 1. Likewise, elements of column j show the probabilities that case-patient j has transmitted their infection to any other case-patient in the observed population. Columns of V therefore add to an estimate of the number of cases infected by case-patient j: its reproduction number.

Supplemental Material C. Sensitivity Analysis of Ignoring Negative Serial Intervals

Pre-symptomatic transmission resulting in negative serial intervals, is possible for COVID-19. Unlike the early outbreak studies in China (*3*,*4*), we could not use the travel history to identify potential exposure and infer the direction of transmission in our study at the state of Georgia. When the onset dates of the two cases in a pair were close, their order (who was infector, who was infectee) could not always be verified. Since the proportion of transmission pairs with negative serial interval was found to be small (*3*,*4*), we ignored negative serial intervals and assumed the person with earlier symptom onset was the infector. We conducted a sensitivity analysis to examine the impact of this assumption on the R_t estimates. Instead of arbitrarily assigning the subject with earlier symptom onset to be the infector, we can swap the infector and infectee for such pairs. As it seems implausible that such reversed order should

occur when the onset dates are several days apart, we only deal with small negative serial intervals: -3 – -1 days and a minor fraction (10%). Appendix Figure 7 shows the empirical and estimated cumulative density function (CDF) of serial interval distribution with and without negative interval. Although negative serial intervals would drastically change the transmission network (who infected whom), this would not necessarily influence the average reproduction number of infectious subjects. In an earlier publication by Wang and Teunis (*2*), we have established that negative serial intervals have only minor influence on reproduction number estimates. Appendix Figures 8–10 show comparisons of R_t for three counties (Clayton, Glynn, Sumter): ignoring negative serial intervals did not impact the estimation of R_t.

References

1. Teunis P, Heijne JC, Sukhrie F, van Eijkeren J, Koopmans M, Kretzschmar M. Infectious disease transmission as a forensic problem: who infected whom? J R Soc Interface. 2013;10:20120955. PubMed https://doi.org/10.1098/rsif.2012.0955

2. Wang Y, Teunis P. Strongly heterogeneous transmission of covid-19 in mainland china: Local and regional variation. Front Med (Lausanne). 2020;7:329. 10.3389/fmed.2020.00329 PubMed https://doi.org/10.3389/fmed.2020.00329

3. Ali ST, Wang L, Lau EHY, Xu XK, Du Z, Wu Y, et al. Serial interval of SARS-CoV-2 was shortened over time by nonpharmaceutical interventions. Science. 2020;369:1106–9. PubMed https://doi.org/10.1126/science.abc9004

4. Xu XK, Liu XF, Wu Y, Ali ST, Du Z, Bosetti P, et al. Reconstruction of transmission pairs for novel coronavirus disease 2019 (COVID-19) in mainland China: estimation of super-spreading events, serial interval, and hazard of infection. Clin Infect Dis. 2020;71:3163–7. 10.1093/cid/ciaa790 PubMed https://doi.org/10.1093/cid/ciaa790

Appendix Table 1. COVID-19 pandemic situation and state government responses*

Date	Situation and Response
January 20, 2020	First COVID-19 case reported in the United States.
March 2, 2020	First COVID-19 case reported in the state of Georgia.
March 14, 2020	Georgia governor declared a public health emergency.
March 23, 2020	Large gatherings were banned and shelter-in-place order was issued for “medically fragile” population.
March 24, 2020	Bars and clubs were ordered to close.
April 1, 2020	All K–12 schools were closed.
April 3, 2020	Statewide shelter-in-place order was issued.
April 24, 2020	Some businesses (gyms, fitness centers, bowling alleys, body art studios, barbers, cosmetologists, hair designers, nail care artists, estheticians, their respective schools, and massage therapists) were allowed to reopen with minimum basic operations.
April 27, 2020	More businesses (theaters, private social clubs, and restaurant dine-in services) were allowed to reopen with social distancing and sanitation mandates.
April 30, 2020	Reopening: Statewide shelter-in-place order was lifted.
June 1, 2020	Further Reopening: Limits on the size of public gathering were relaxed: bars and nightclubs were allowed to reopen, sports events could resume, and summer schools and camps were allowed to begin sessions.

*Executive orders from Georgia governor available at https://gov.georgia.gov/executive-action/executive-orders/2020-executive-orders.

Appendix Table 2. Data of available information about demographics, epidemiological timelines, clinical outcomes, and contact tracing

Variable	Description	Value
Demographics		
UID	Unique identifier (UID) associated with patient	patient UID
Age	Patient’s age	Positive Integer
Sex	Patient’s sex	Male; Female
Race	Patient’s race	Black; White; Other
County	County of residence	159 counties in Georgia
Epidemiological Time lines		
Reported date	Date of first report to public health	Date
Symptom onset	Date of symptom onset	Date
Date of sample collection	Date of first specimen collection	Date
Clinical Outcomes		
Hospitalized	Hospitalization of patient during illness	Yes; No
Ventilator	Patient received intubation or mechanical ventilation during hospitalization	Yes; No
Abnormal Chest X-ray	Patient had an abnormal chest X-ray	Yes; No
Death	Death occurred as a result of COVID-19 infection and was reported to public health	Yes; No
Fever	Fever of a >100.4 recorded by patient or medical provider	Yes; No
Cough	Cough (new onset or worsening of chronic cough)	Yes; No
Short of Breath	Shortness of breath (dyspnea)	Yes; No
Diarrhea	Diarrhea (≥3 loose/looser stools in 24 hours period)	Yes; No
Contact Tracing		
Close Contact	Unique identifier of confirmed COVID-19 case to which patient was exposed	Patient UID
Outbreak	Is this case part of an outbreak?	Yes; No
Outbreak ID	If this is part of an outbreak, outbreak ID?	Outbreak ID

Appendix Table 3. Clinical outcomes and demographic information for 4080 tracked pairs of primary case-patients and secondary case-patients.

Variable	Primary Case-patient		Secondary Case-patient	
Clinical Outcomes	Yes (%)	No (%)	Yes (%)	No (%)
Hospitalized	737 (18.1)	3,232 (79.2)	504 (12.4)	3,486 (85.4)
Ventilator Use	121 (3.0)	3,139 (76.9)	92 (2.3)	3,246 (79.6)
Abnormal Chest X-ray	370 (9.1)	2,697 (66.1)	216 (5.3)	2,909 (71.3)
Death	154 (3.8)	3,436 (84.2)	99 (2.4)	3,552 (87.1)
Fever	2,056 (50.4)	1,723 (42.2)	1,654 (40.5)	2,141 (52.5)
Cough	2,662 (65.2)	1,197 (29.3)	2283 (56)	1,569 (38.5)
Short of Breath	1,438 (35.2)	2,323 (56.9)	1,129 (27.7)	2,640 (64.7)
Diarrhea	1,093 (26.8)	2,565 (62.9)	916 (22.5)	2,792 (68.4)
Demographics	Male (%)	Female (%)	Male (%)	Female (%)
Sex	1,851 (45.4)	2,220 (54.4)	1,707 (41.8)	2,352 (57.6)
	White (%)	Black (%)	White (%)	Black (%)
Race	1,959 (48.0)	1,273 (31.2)	1,978 (48.5)	1,247 (30.6)
	Mean	SD	Mean	SD
Age	42.7	18.3	40.8	20.4

Appendix Table 4. Serial interval by subgroup with different clinical characteristics and demographic characteristics.

Subgroup	n	Mean (day)	10th–90th Percentile Range (day)	Shape	Scale
Clinical Outcome					
Hospitalized+	650	5.69	1.55–11.00	2.05	2.77
Hospitalized-	2,986	4.84	1.29–9.41	2.00	2.42
Ventilator+	106	6.28	1.83–11.93	2.21	2.84
Ventilator-	2,877	4.93	1.32–9.57	2.02	2.44
Abnormal Chest X-ray+	307	6.13	1.80–11.64	2.22	2.76
Abnormal Chest X-ray-	2,492	4.84	1.31–9.36	2.04	2.37
Death+	133	5.91	1.61–11.42	2.06	2.87
Death-	3,153	4.95	1.34–9.60	2.03	2.44
Fever+	1,889	5.12	1.40–9.88	2.06	2.48
Fever-	1,575	4.87	1.26–9.55	1.94	2.51
Cough+	2,435	5.09	1.38–9.85	2.05	2.49
Cough-	1,096	4.79	1.22–9.42	1.92	2.50
Short of Breath+	1,302	5.25	1.44–10.12	2.07	2.54
Short of Breath-	2,140	4.81	1.26–9.38	1.98	2.43
Diarrhea+	993	5.22	1.31–10.30	1.88	2.77
Diarrhea-	2,349	4.87	1.32–9.41	2.05	2.37
Age					
20-	412	4.10	1.07–8.00	1.96	2.09
20–40	1,369	4.78	1.30–9.24	2.05	2.33
40–60	1,330	5.29	1.44–10.23	2.05	2.58
60+	618	5.47	1.45–10.66	1.99	2.75
Sex					
Male	1,720	4.85	1.26–9.47	1.96	2.47
Female	2,009	5.11	1.39–9.89	2.05	2.50
Race					
Black	1,147	5.39	1.46–10.43	2.04	2.64
White	1,790	4.73	1.22–9.25	1.95	2.43
Other	801	4.99	1.32–9.69	2.00	2.49
Resident Area					
Metro Atlanta	839	4.88	1.33–9.43	2.06	2.37
Out of Metro Atlanta	2,899	5.02	1.33–9.78	1.99	2.53
Time Period					
Feb–Apr	1,070	5.97	1.65–11.50	2.09	2.86
May	850	5.03	1.41–9.65	2.11	2.38
Jun–Jul	1,818	4.40	1.18–8.52	2.03	2.17
Total	3,738	4.99	1.33–9.71	2.00	2.49

Subgroups were defined based on the primary case-patient characteristics. Characteristics variables were defined in Appendix Table 2. For clinical characteristics, “+” represents yes and “-” represents. The shape and scale parameters of gamma distributions were estimated using maximum likelihood estimator.

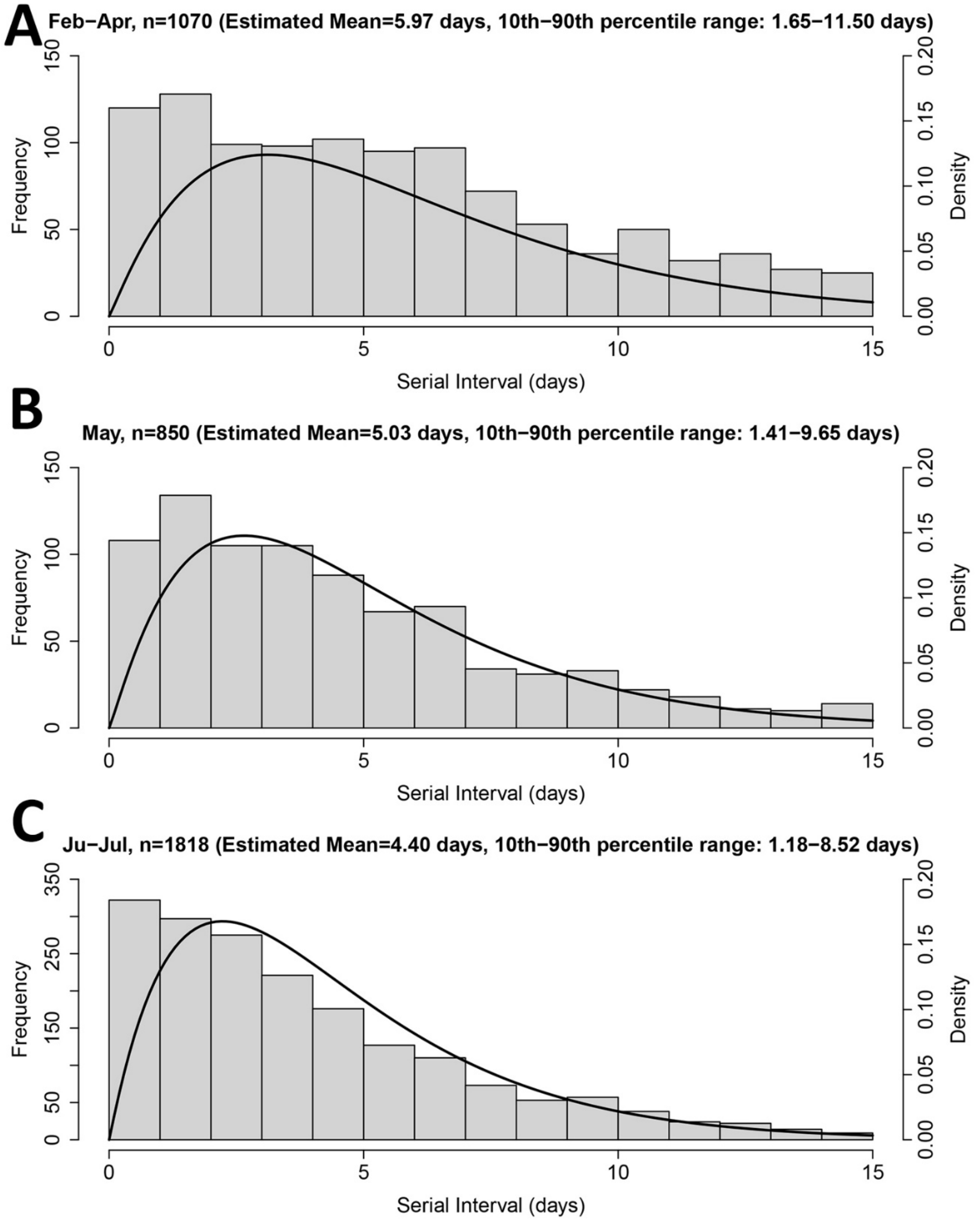

Appendix Figure 1. The observed (histograms) and estimated (density plot) distribution of the serial interval during three time periods: Early transmission and shelter-in-place (Feb–Apr); after reopening (May); further reopening (Jun–Jul).

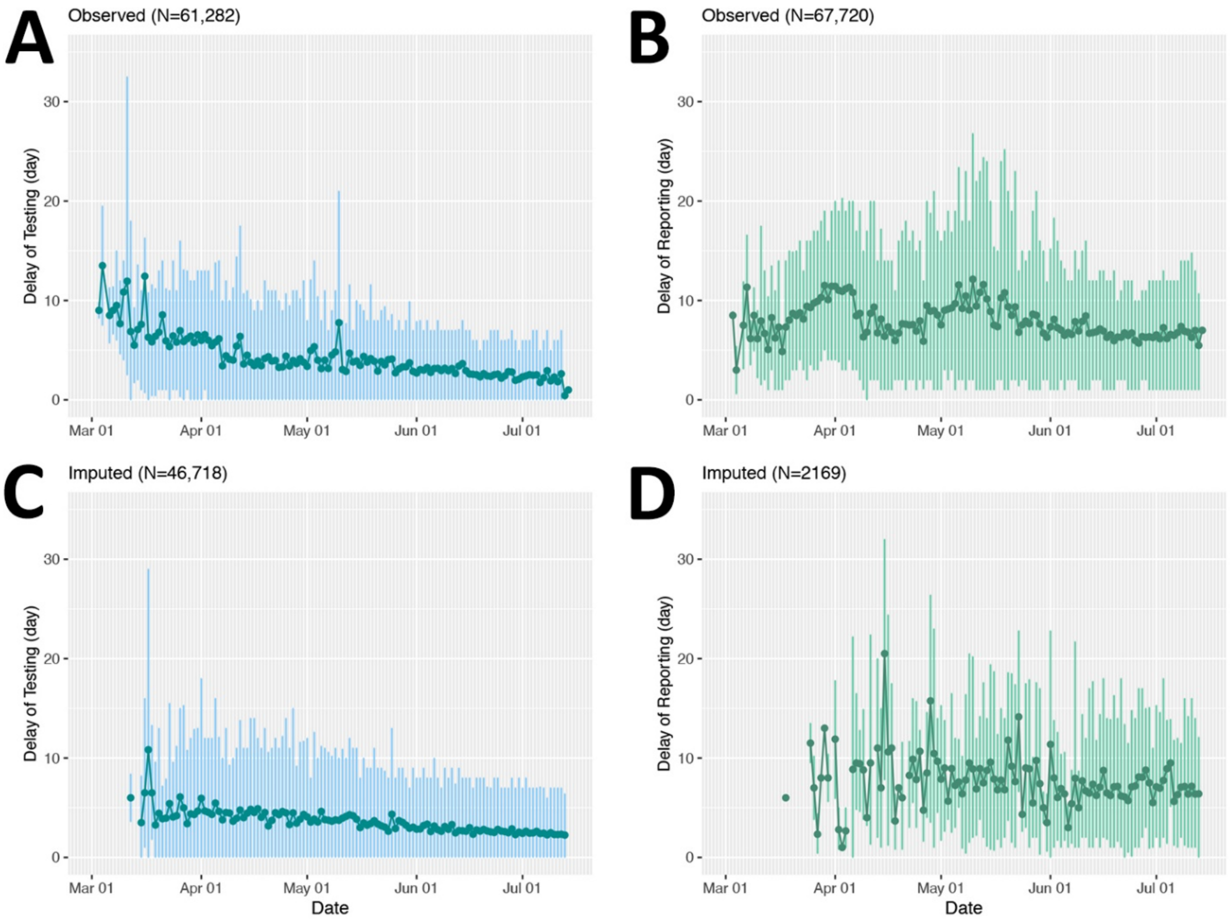

Appendix Figure 2. The observed (a) and imputed (c) delay between the date of first specimen collection and the date of symptom onset, and the observed (b) and imputed (d) delay between the date of laboratory report and the date of symptom onset between March 1–July 13, 2020. The solid lines represent the mean delay and the error bars represent the 10th and 90th percentile range.

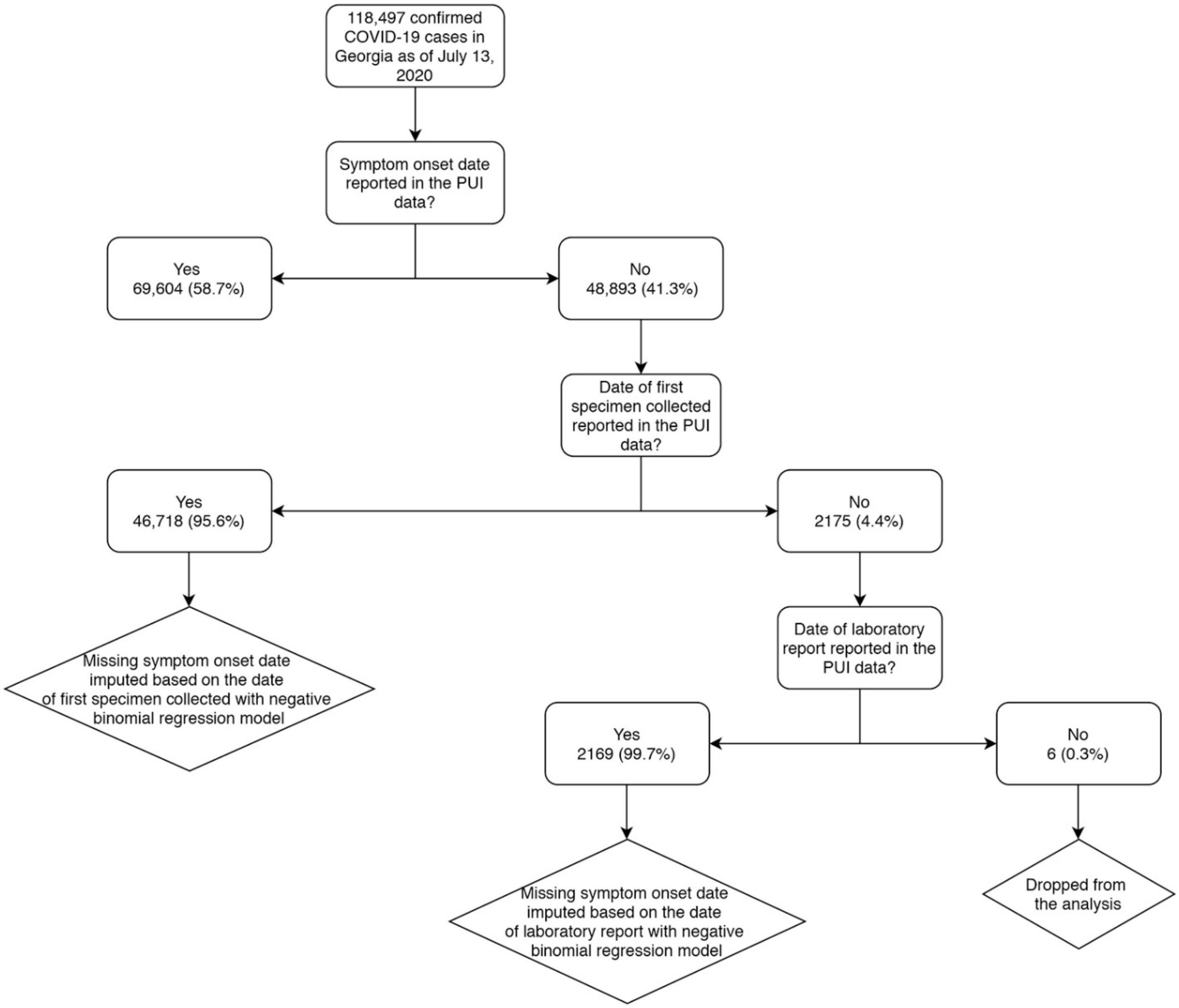

Appendix Figure 3. The flow chart of the imputation procedure for missing symptom onset dates.

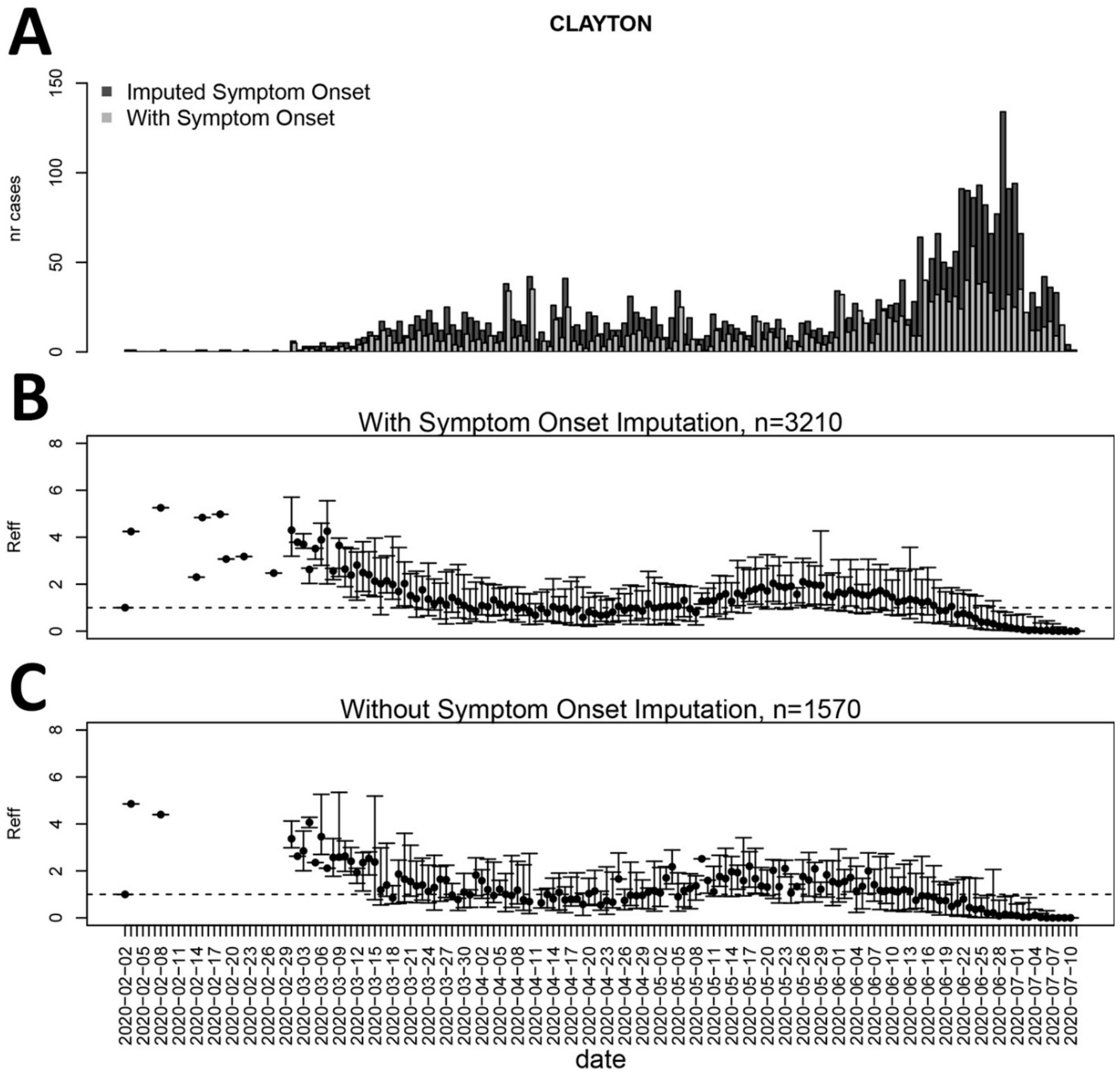

Appendix Figure 4. Epidemic curves and corresponding reproduction number estimates with and without imputed missing symptom onset until July 13th in Clayton county.

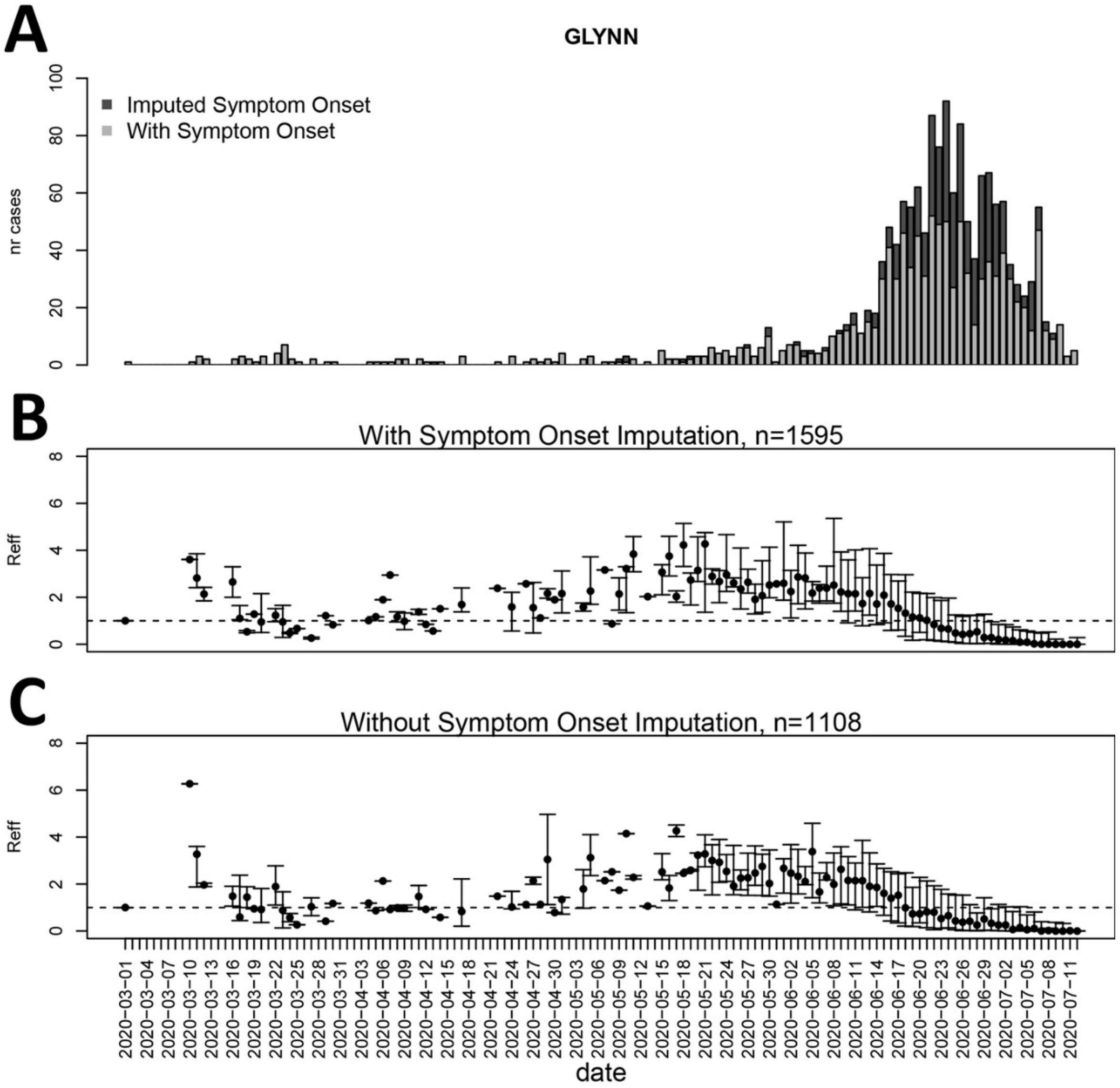

Appendix Figure 5. Epidemic curves and corresponding reproduction number estimates with and without imputed missing symptom onset until July 13th in Glynn county.

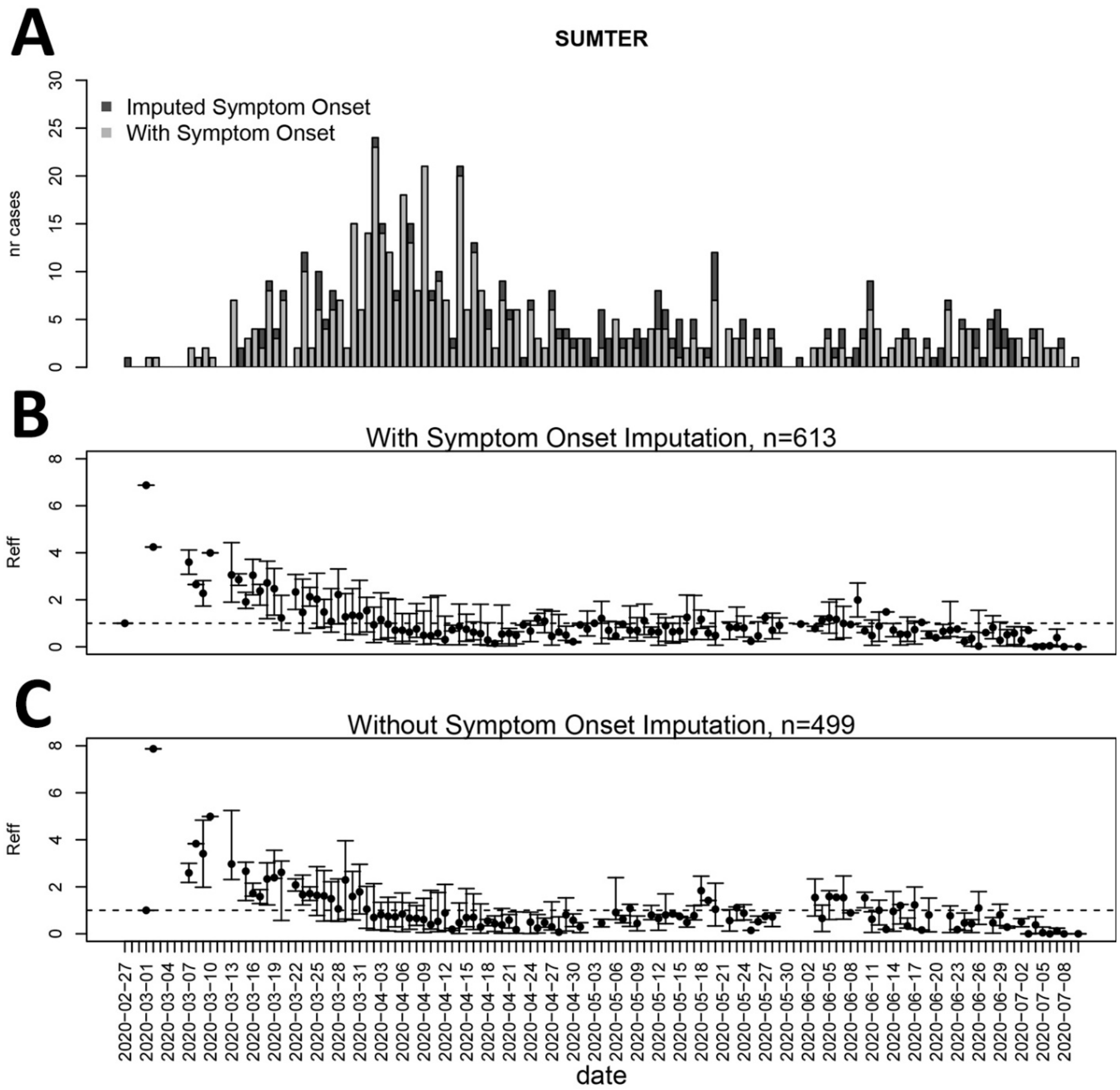

Appendix Figure 6. Epidemic curves and corresponding reproduction number estimates with and without imputed missing symptom onset until July 13th in Sumter county.

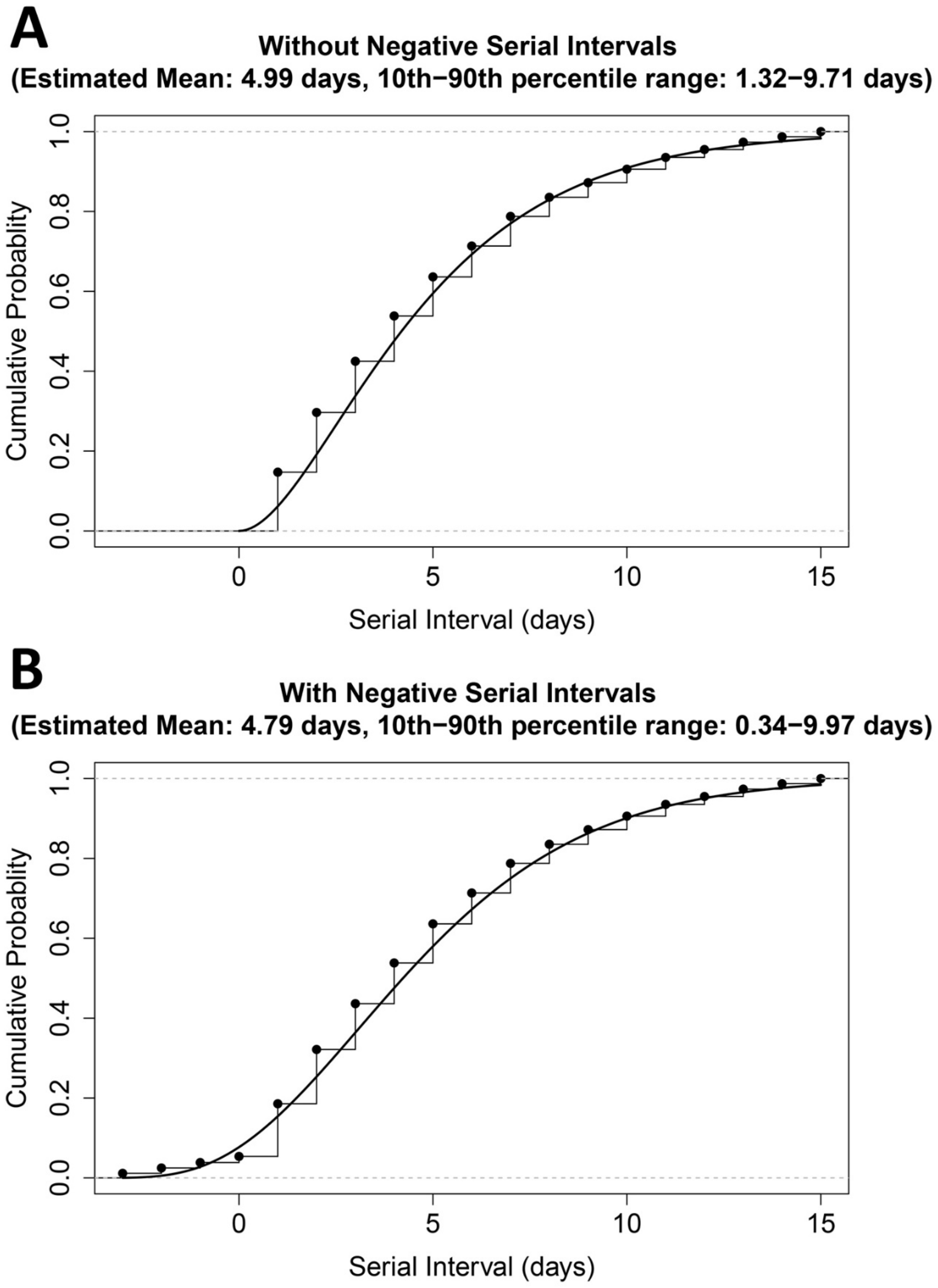

Appendix Figure 7. The empirical cumulative distribution and estimated cumulative distribution of the serial interval ignoring negative serial intervals (top) and considering a proportion (10%) of negative serial intervals.

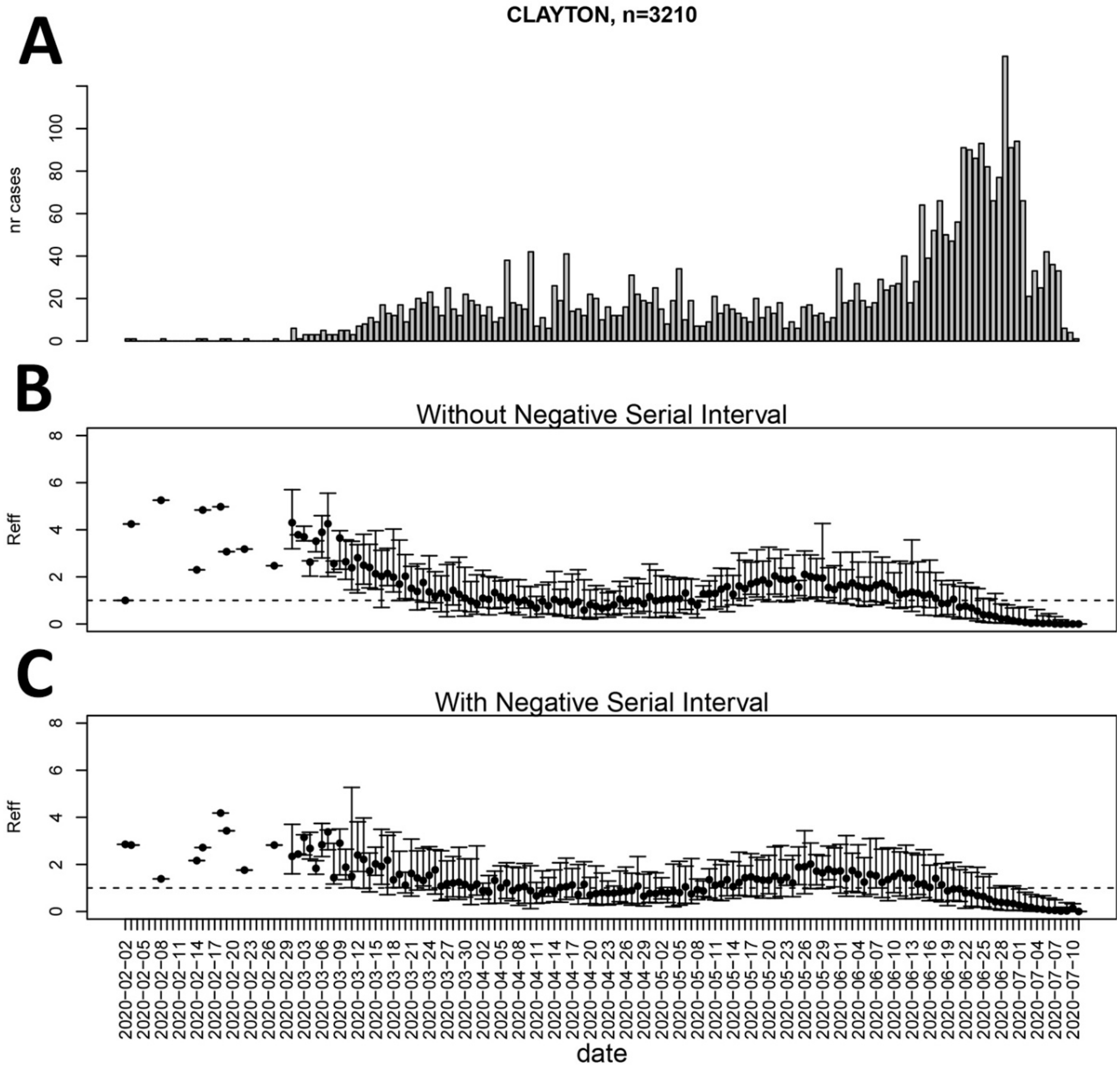

Appendix Figure 8. Epidemic curves and corresponding reproduction number estimates with and without considering negative serial intervals until July 13th in Clayton county.

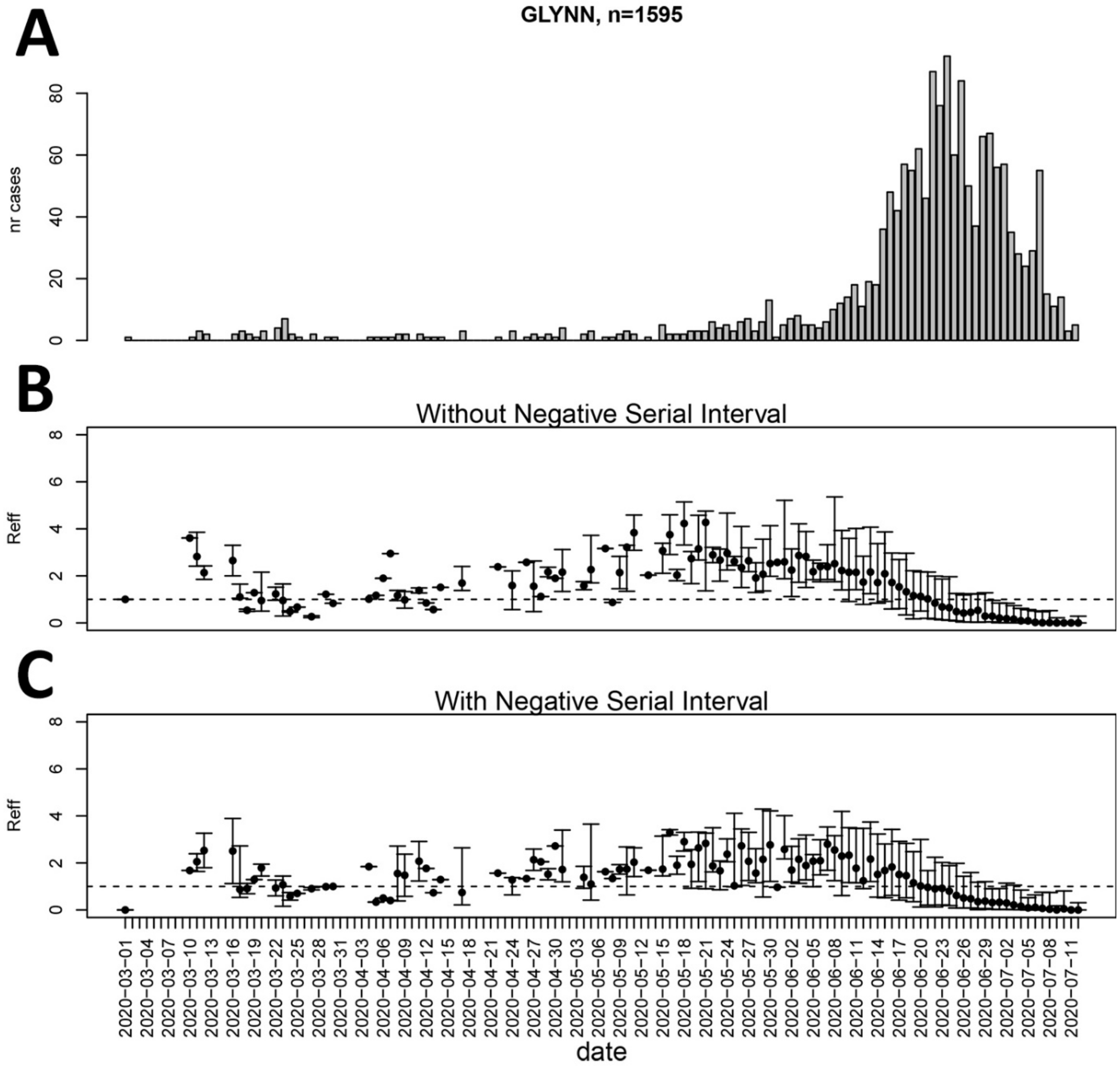

Appendix Figure 9. Epidemic curves and corresponding reproduction number estimates with and without considering negative serial intervals until July 13th in Glynn county.

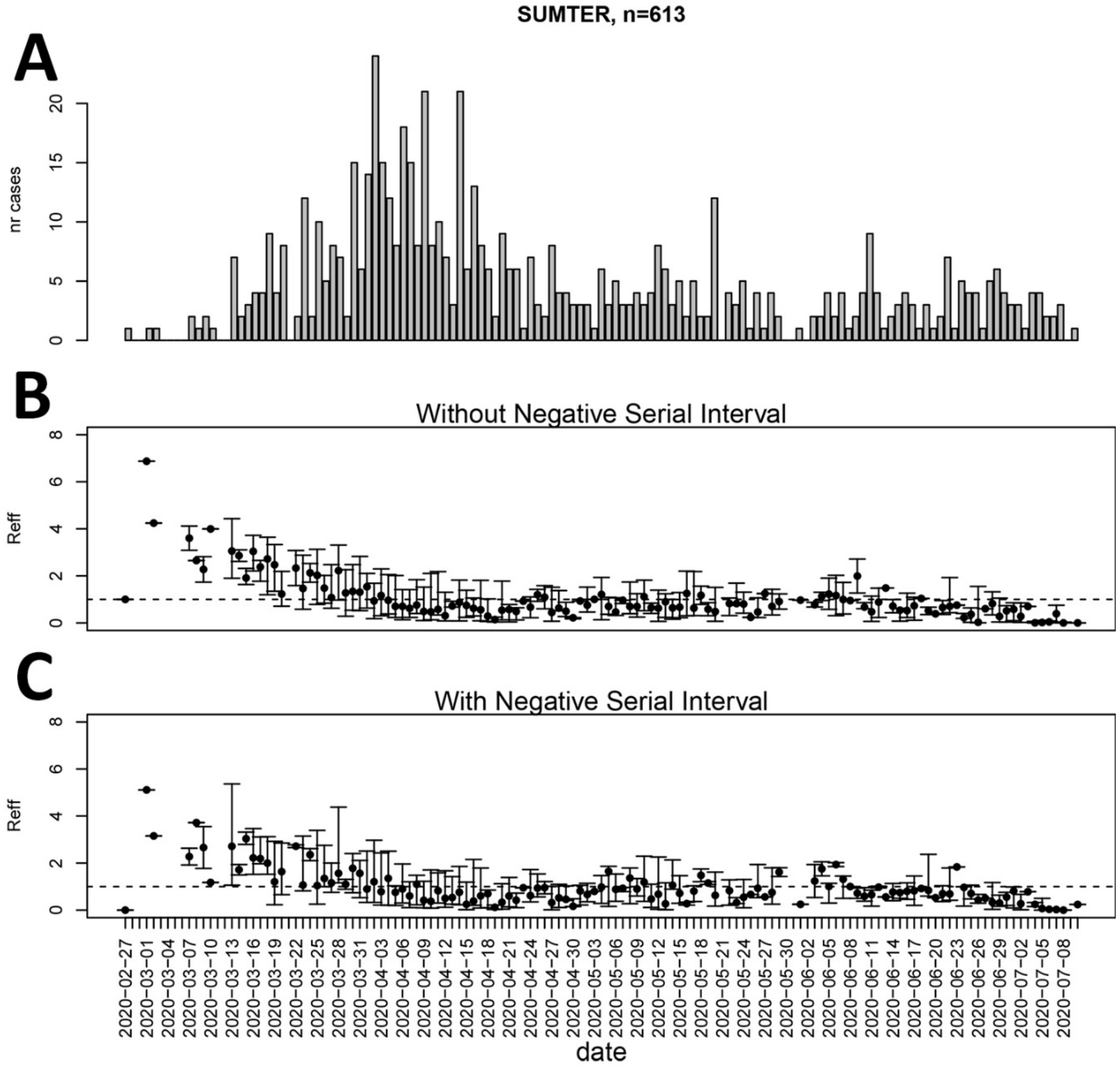

Appendix Figure 10. Epidemic curves and corresponding reproduction number estimates with and without considering negative serial intervals until July 13th in Sumter county.

Appendix Figures 11–169 (following pages)**.** Epidemic curves and reproduction number estimates until July 13th for all counties in Georgia.

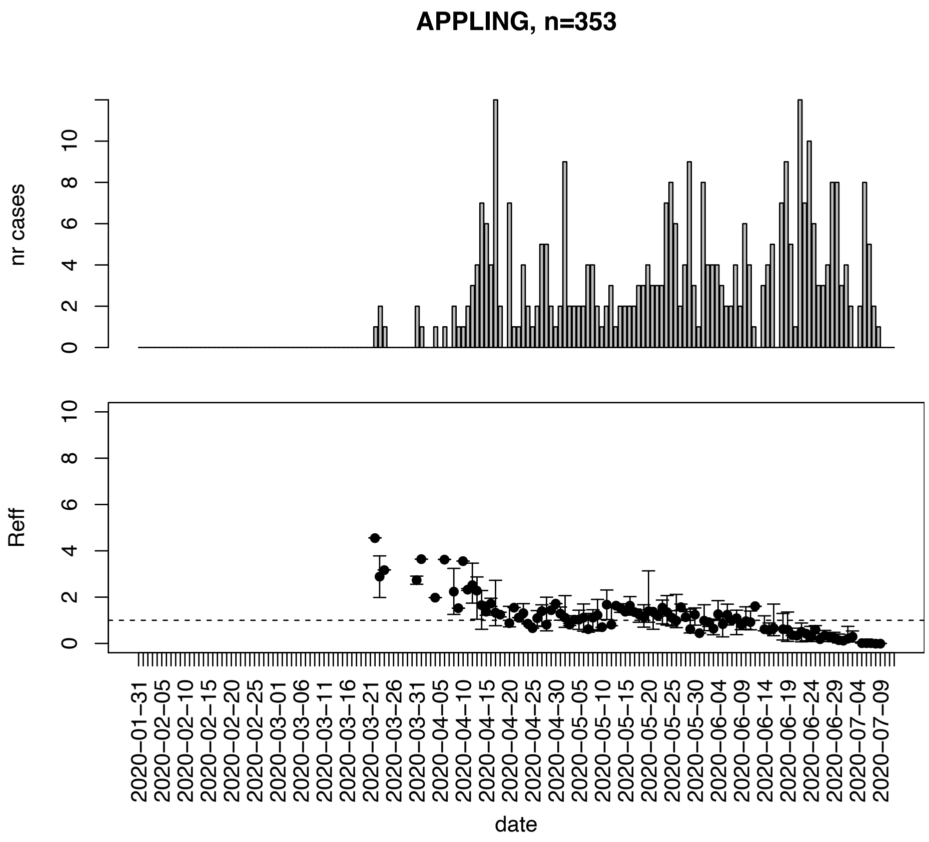

Appendix Figure 11. Epidemic curves and reproduction number estimates until July 13th in Appling county.

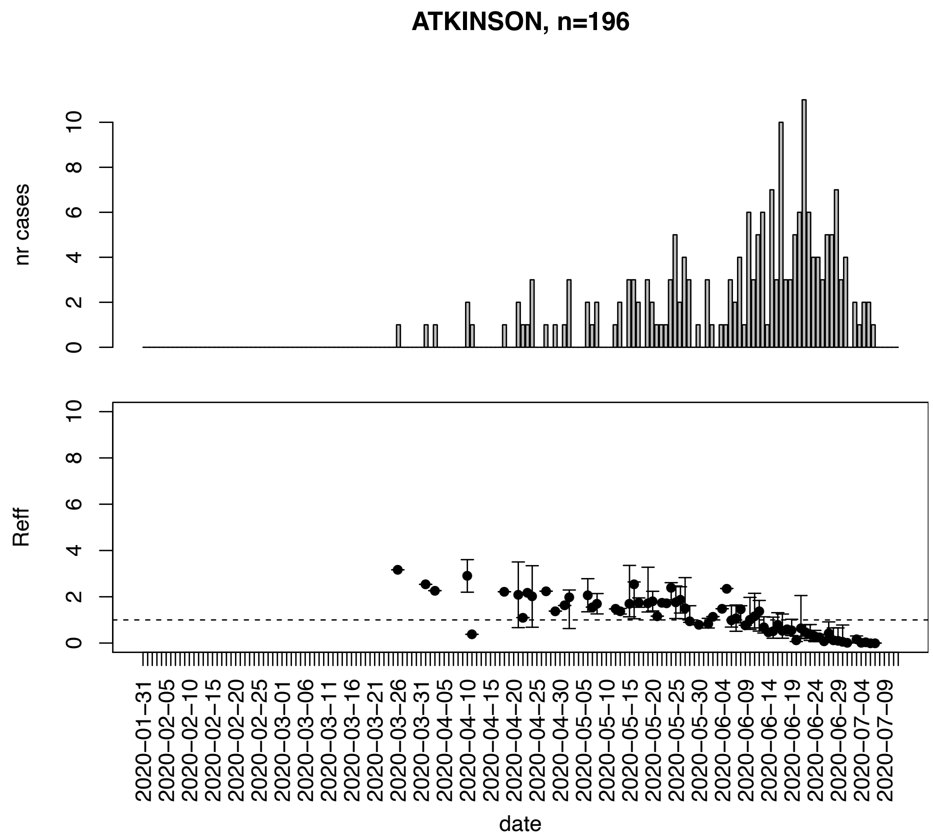

Appendix Figure 12. Epidemic curves and reproduction number estimates until July 13th in Atkinson county.

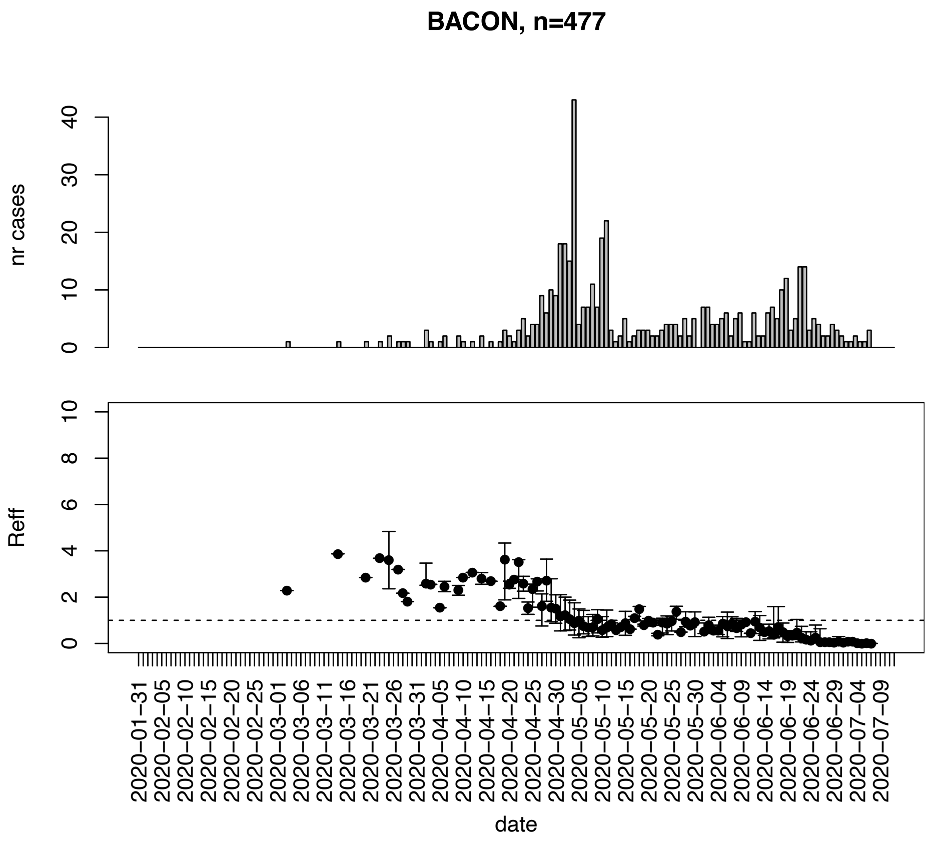

Appendix Figure 13. Epidemic curves and reproduction number estimates until July 13th in Bacon county.

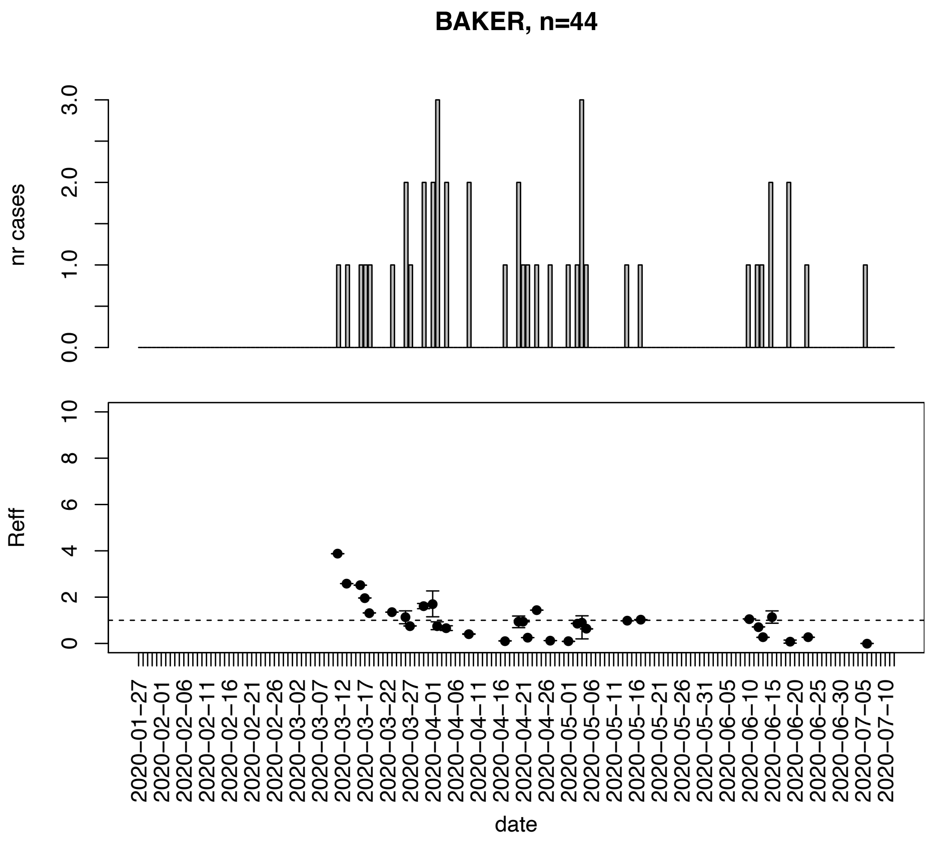

Appendix Figure 14. Epidemic curves and reproduction number estimates until July 13th in Baker county.

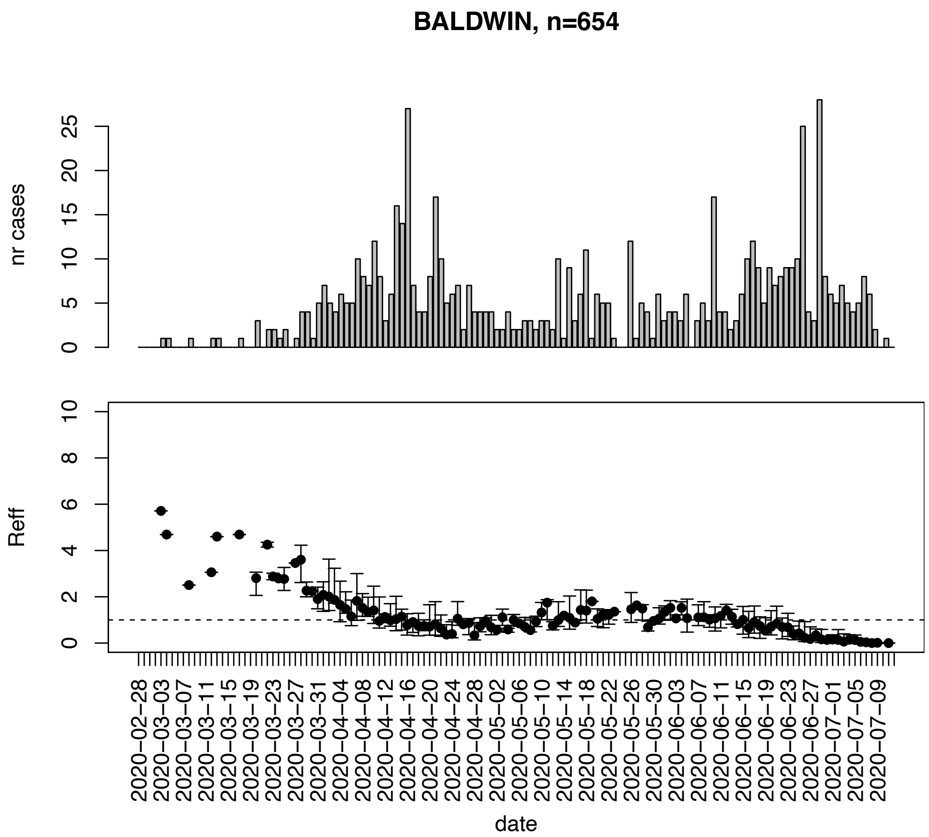

Appendix Figure 15. Epidemic curves and reproduction number estimates until July 13th in Baldwin county.

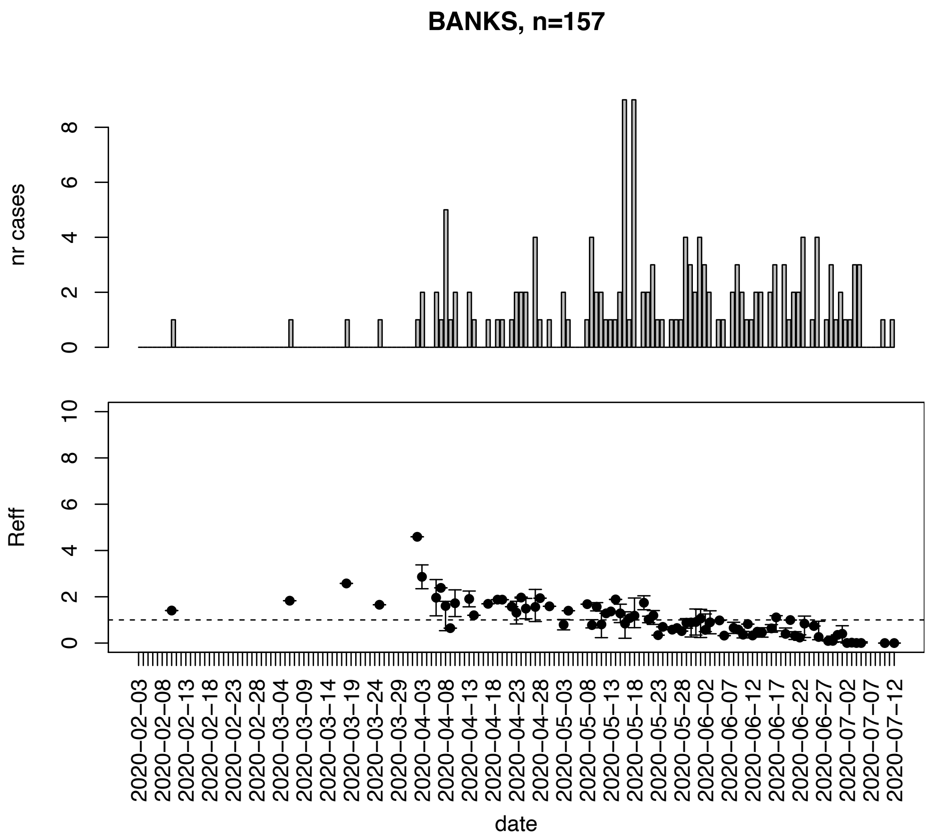

Appendix Figure 16. Epidemic curves and reproduction number estimates until July 13th in Banks county.

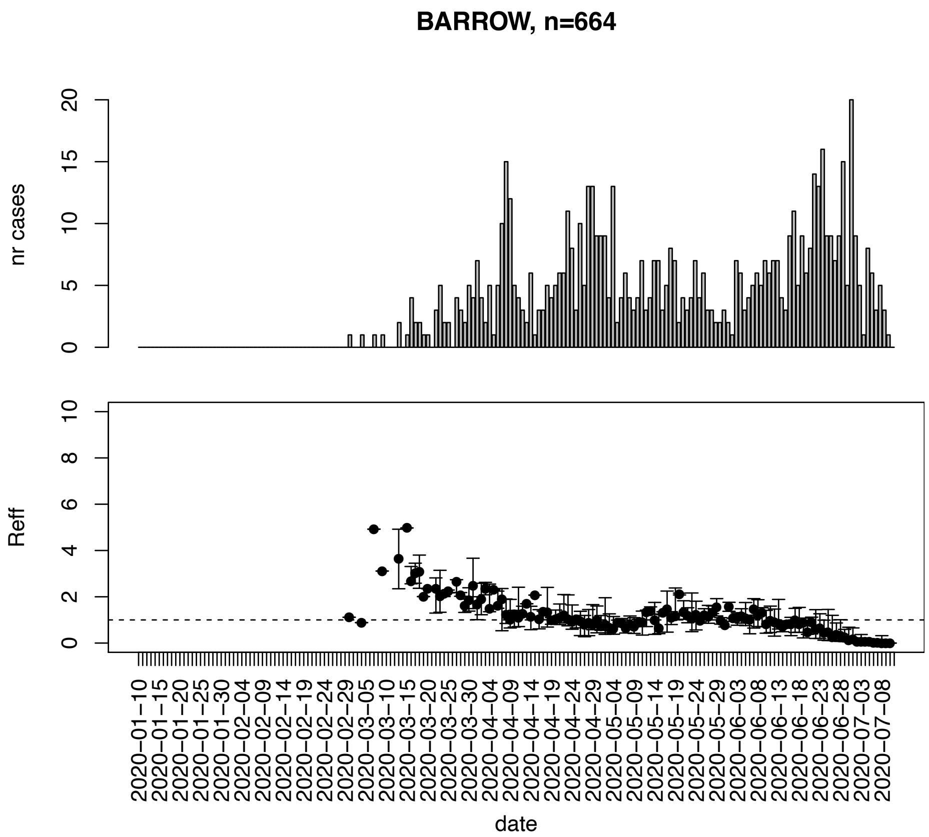

Appendix Figure 17. Epidemic curves and reproduction number estimates until July 13th in Barrow county.

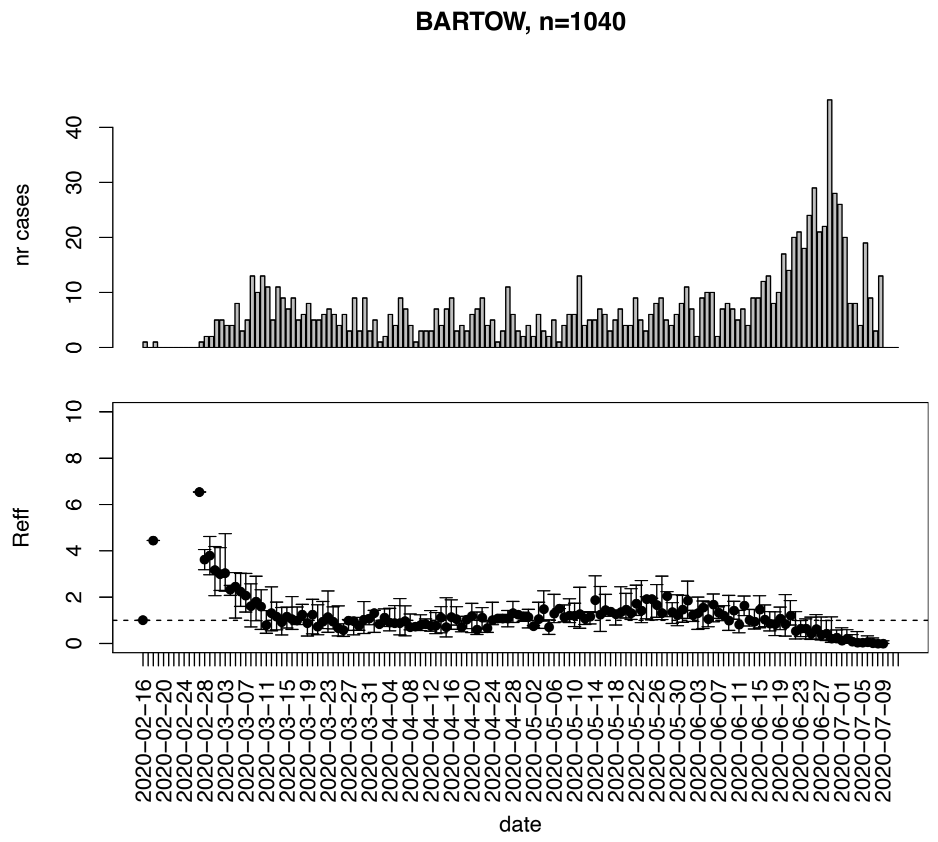

Appendix Figure 18. Epidemic curves and reproduction number estimates until July 13th in Bartow county.

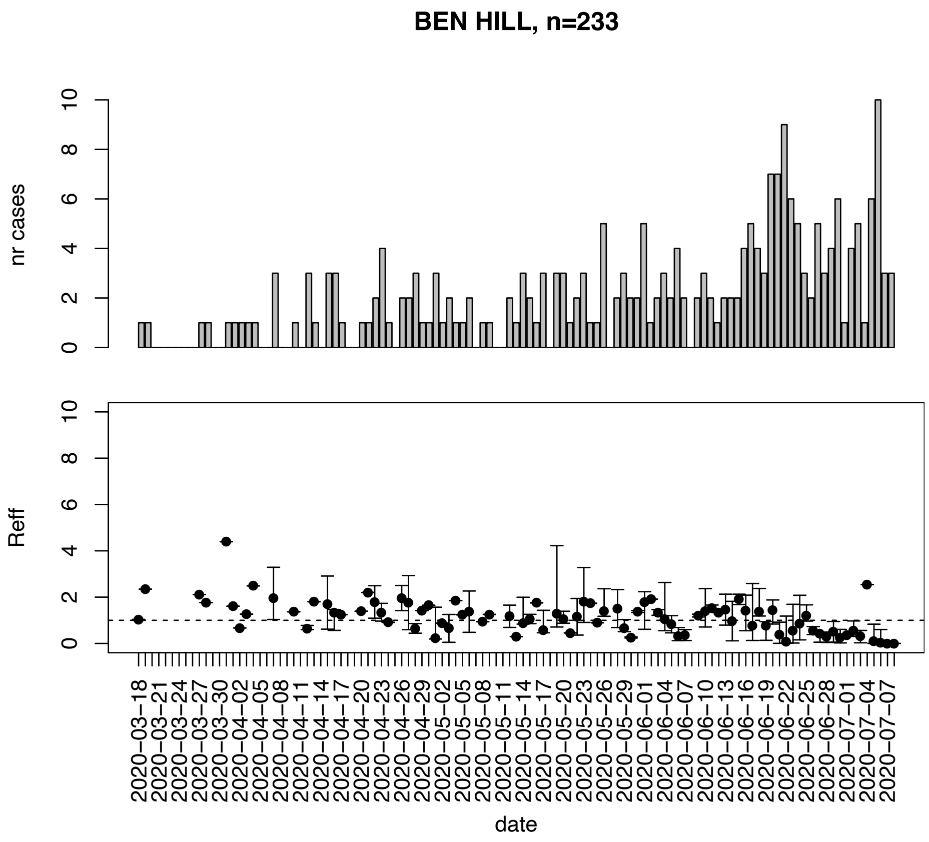

Appendix Figure 19. Epidemic curves and reproduction number estimates until July 13th in Ben Hill county.

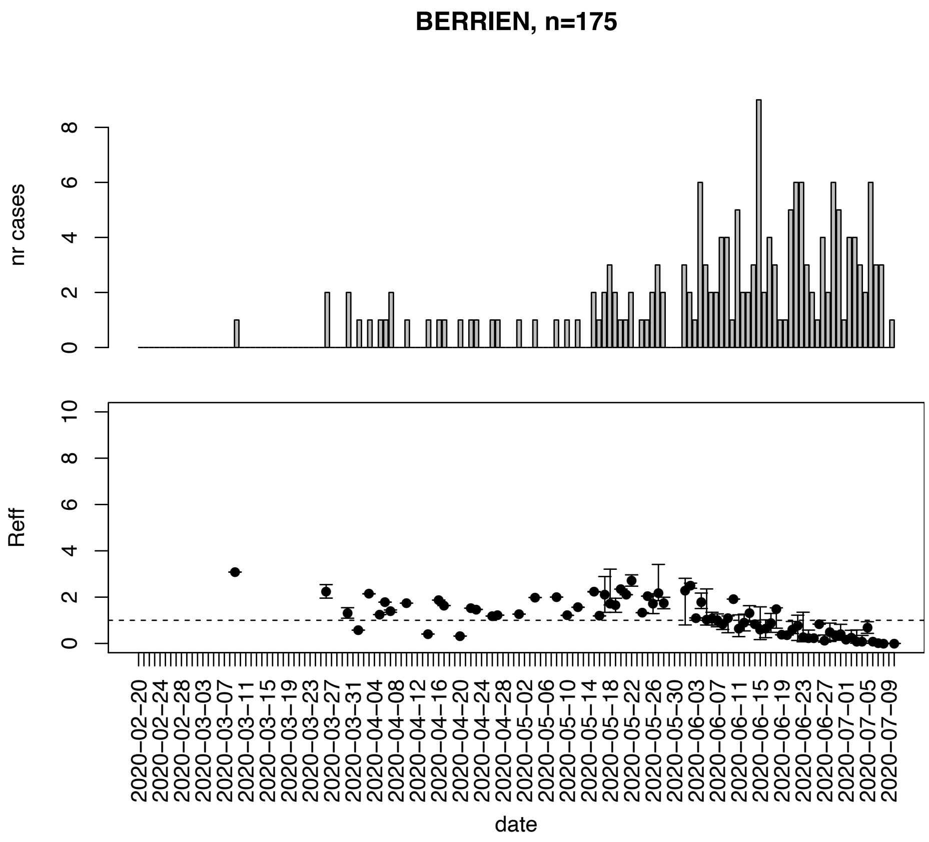

Appendix Figure 20. Epidemic curves and reproduction number estimates until July 13th in Berrien county.

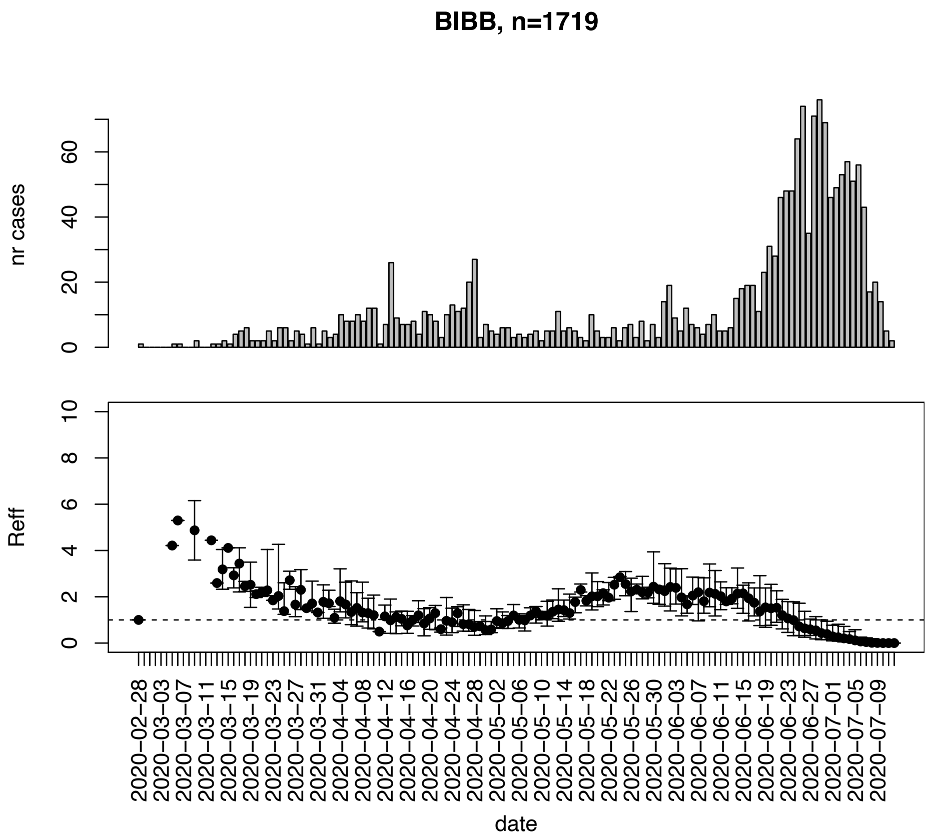

Appendix Figure 21. Epidemic curves and reproduction number estimates until July 13th in Bibb county.

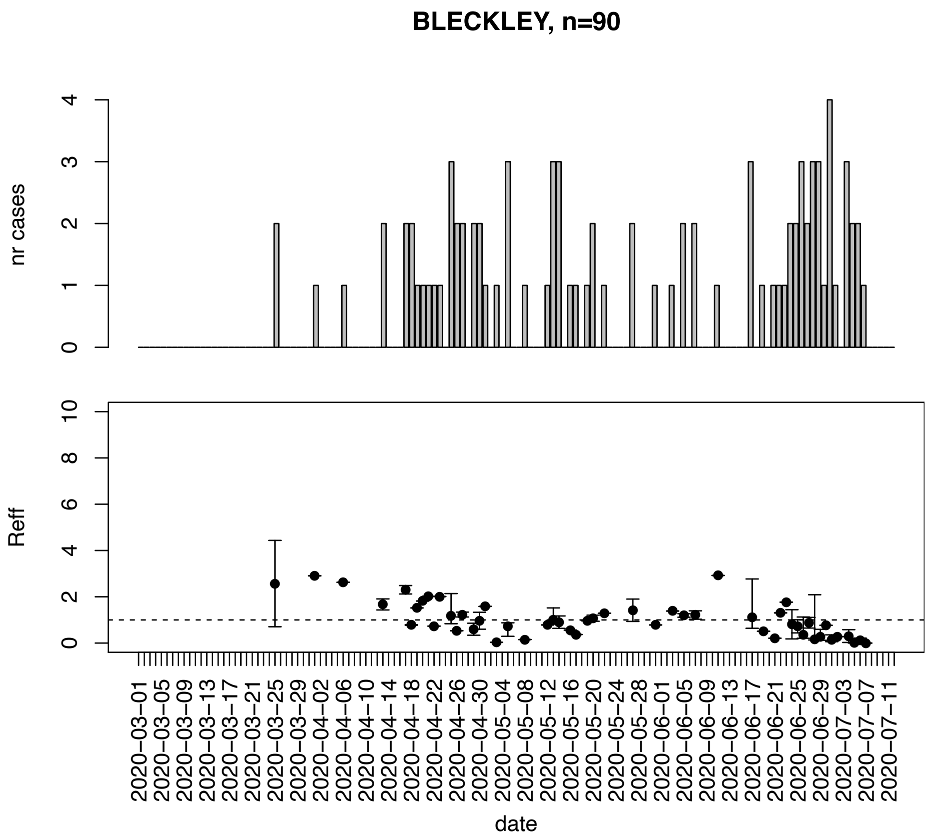

Appendix Figure 22. Epidemic curves and reproduction number estimates until July 13th in Bleckley county.

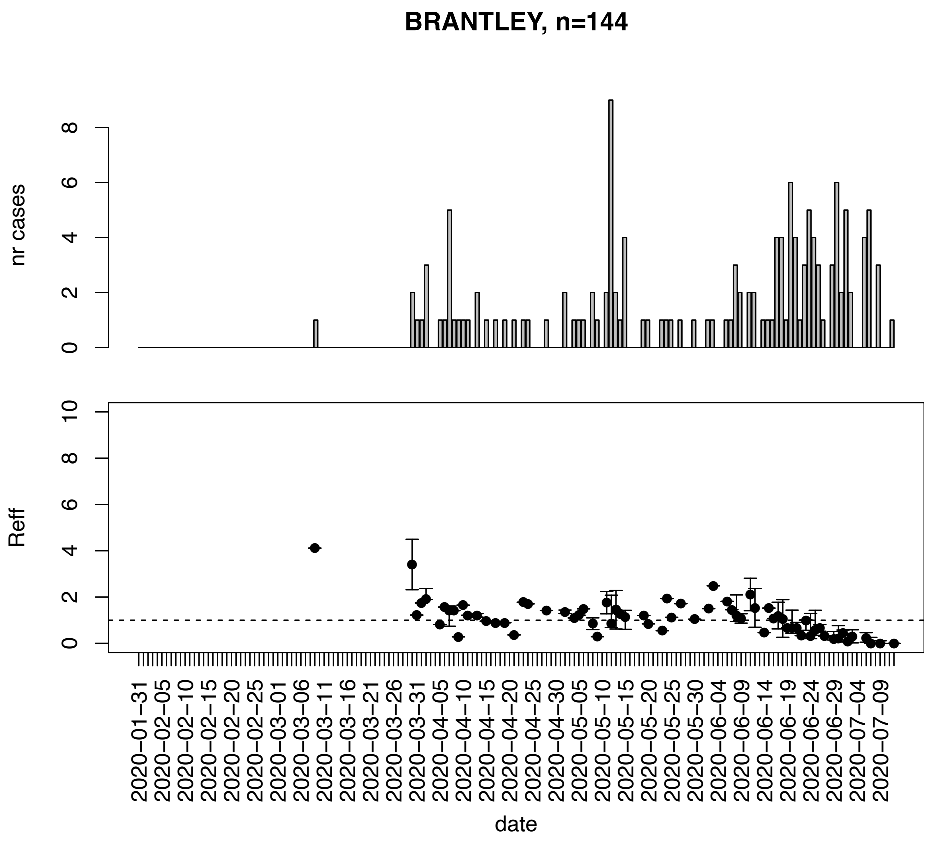

Appendix Figure 23. Epidemic curves and reproduction number estimates until July 13th in Brantley county.

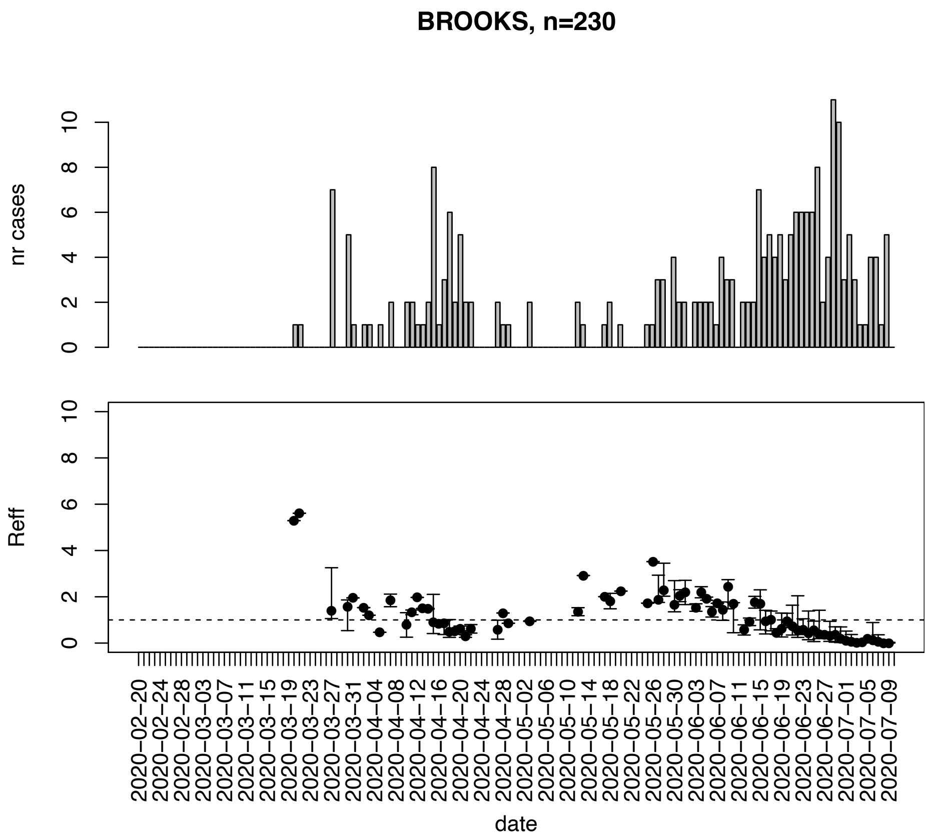

Appendix Figure 24. Epidemic curves and reproduction number estimates until July 13th in Brooks county.

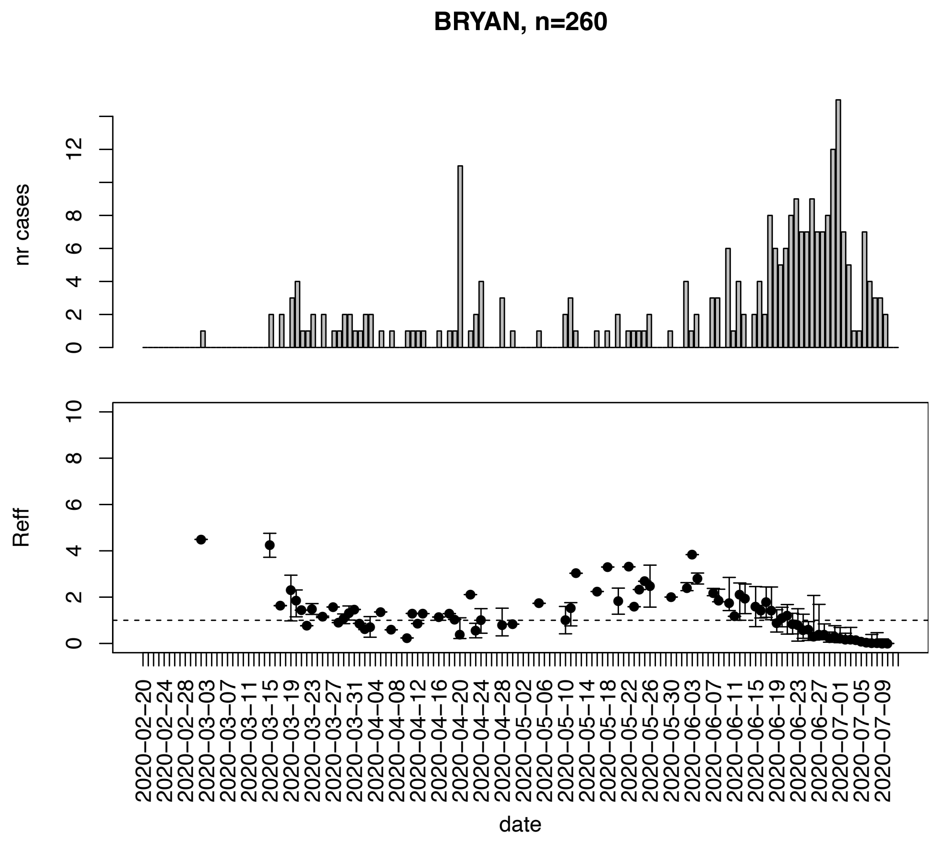

Appendix Figure 25. Epidemic curves and reproduction number estimates until July 13th in Bryan county.

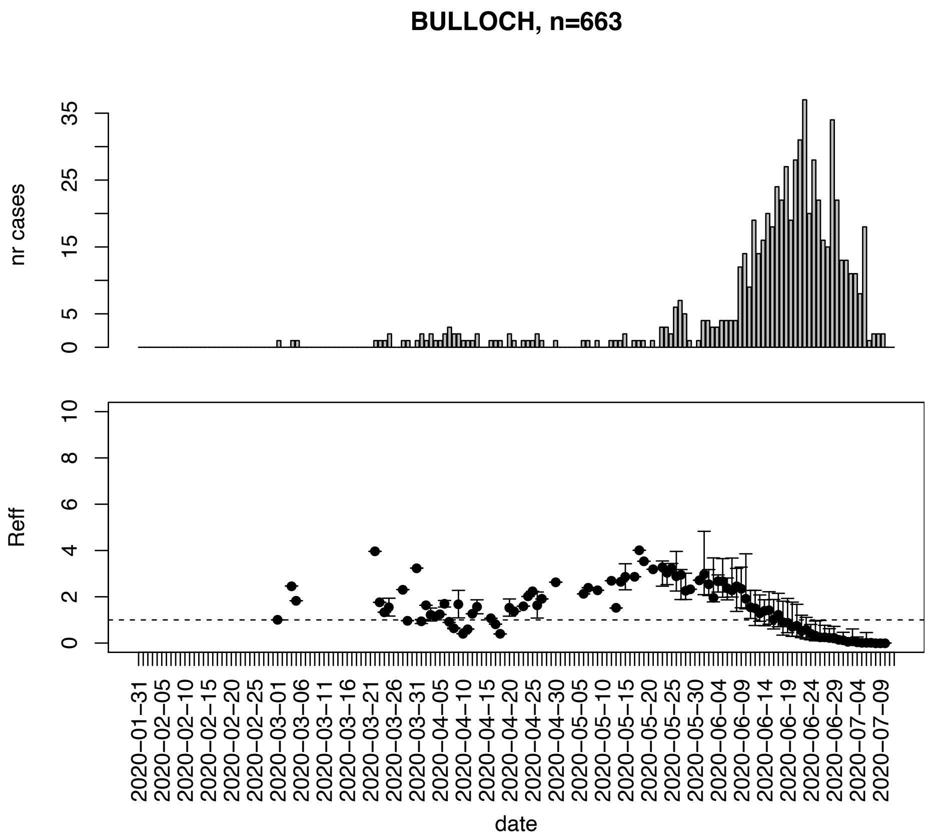

Appendix Figure 26. Epidemic curves and reproduction number estimates until July 13th in Bulloch county.

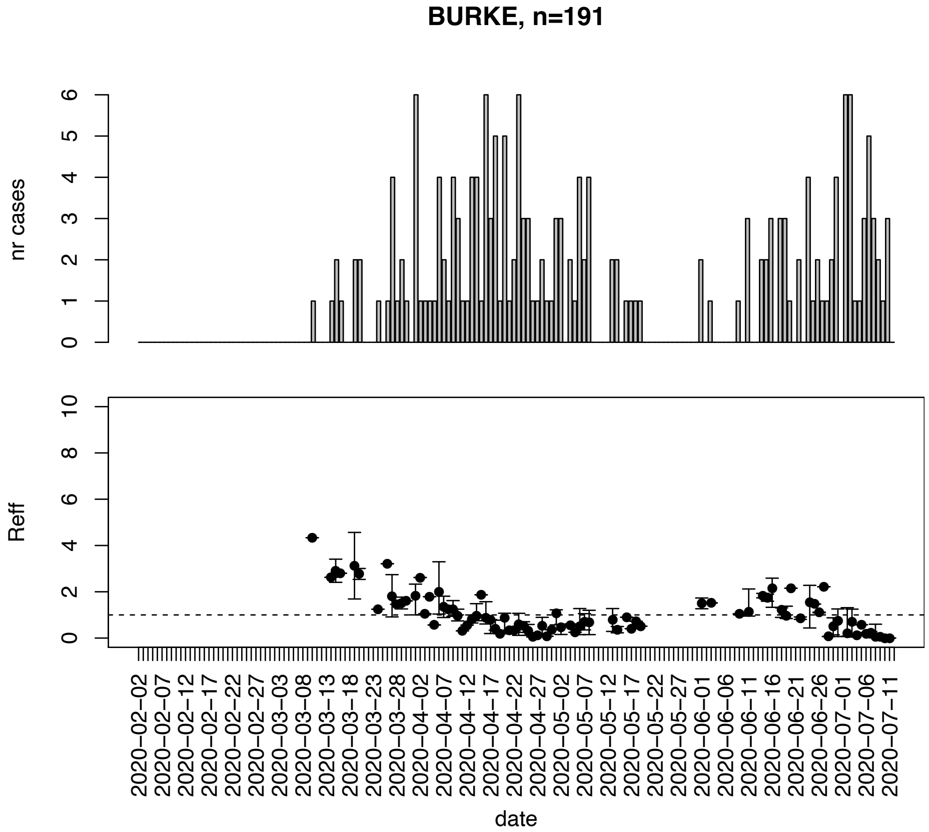

Appendix Figure 27. Epidemic curves and reproduction number estimates until July 13th in Burke county.

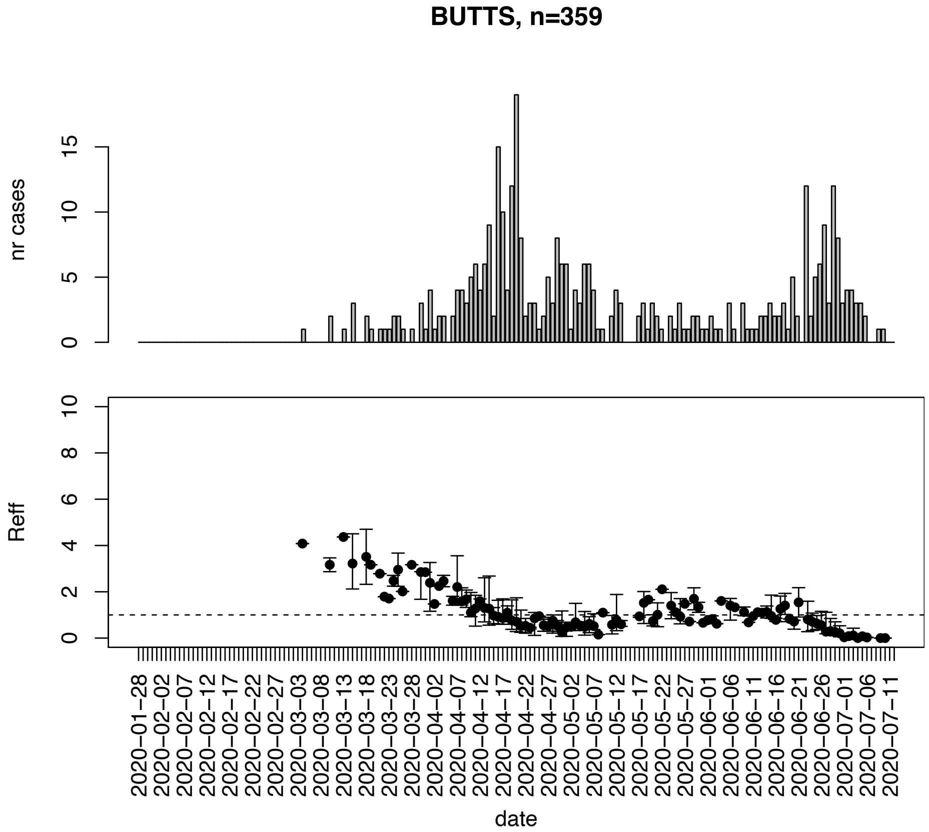

Appendix Figure 28. Epidemic curves and reproduction number estimates until July 13th in Butts county.

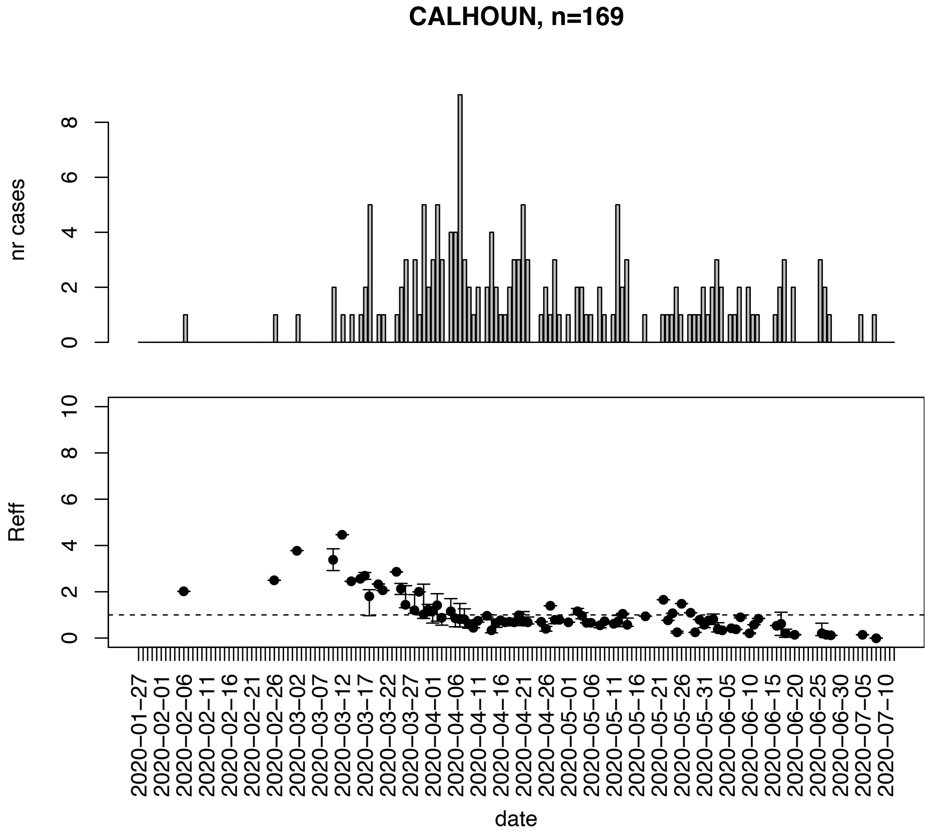

Appendix Figure 29. Epidemic curves and reproduction number estimates until July 13th in Calhoun county.

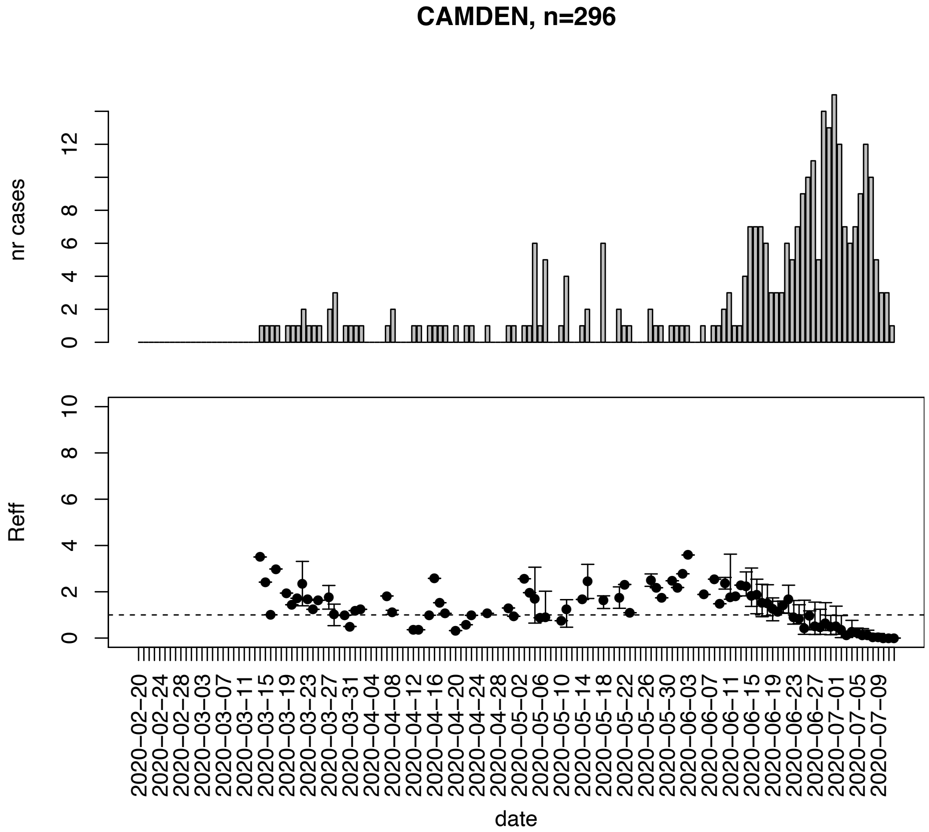

Appendix Figure 30. Epidemic curves and reproduction number estimates until July 13th in Camden county.

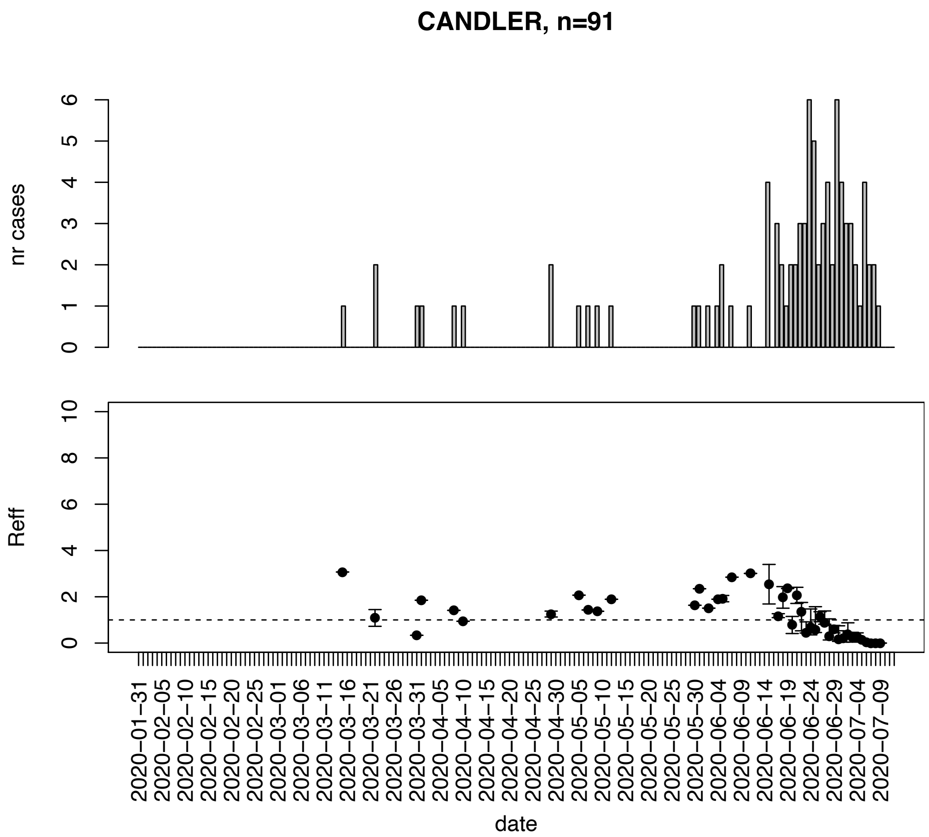

Appendix Figure 31. Epidemic curves and reproduction number estimates until July 13th in Candler county.

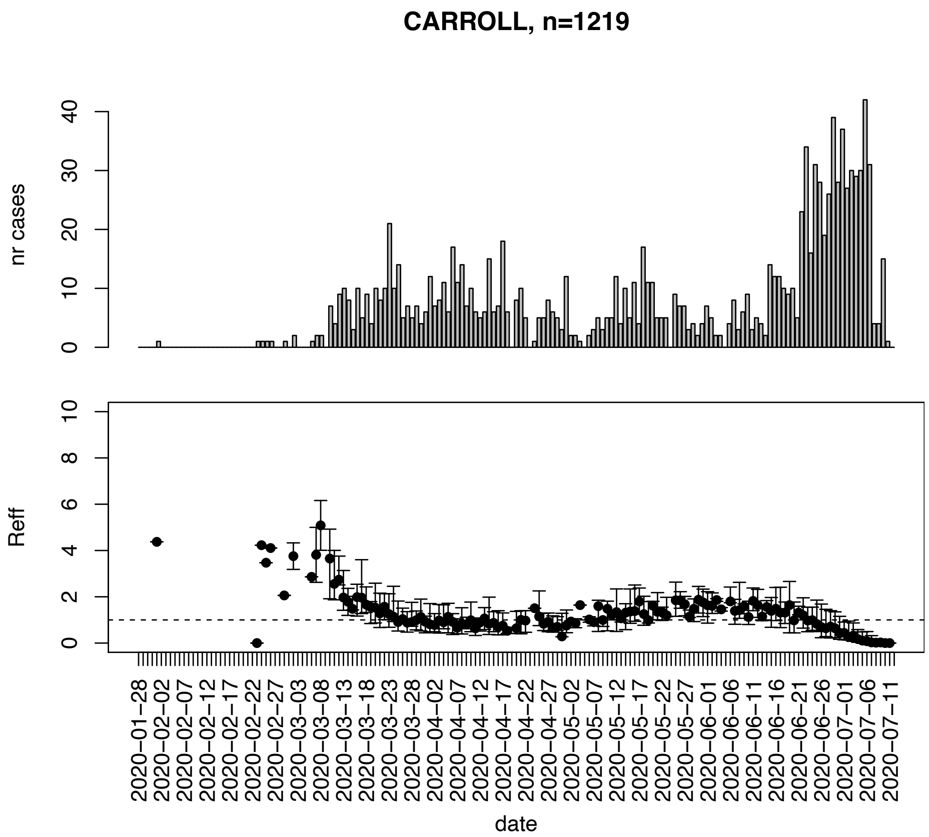

Appendix Figure 32. Epidemic curves and reproduction number estimates until July 13th in Carroll county.

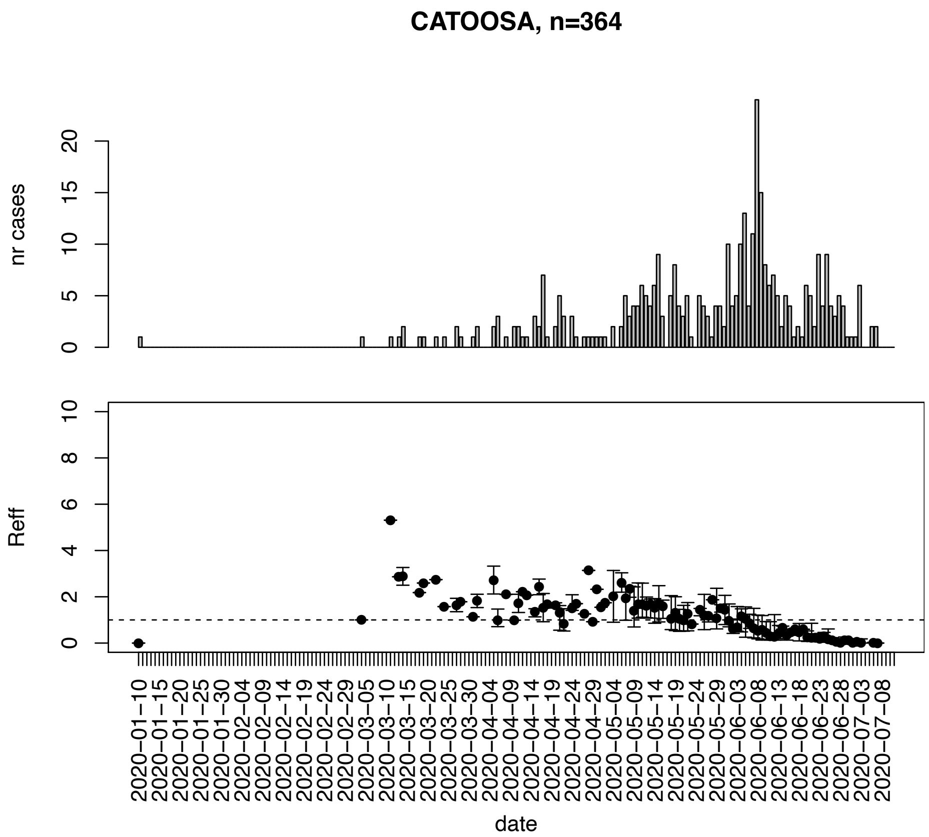

Appendix Figure 33. Epidemic curves and reproduction number estimates until July 13th in Catoosa county.

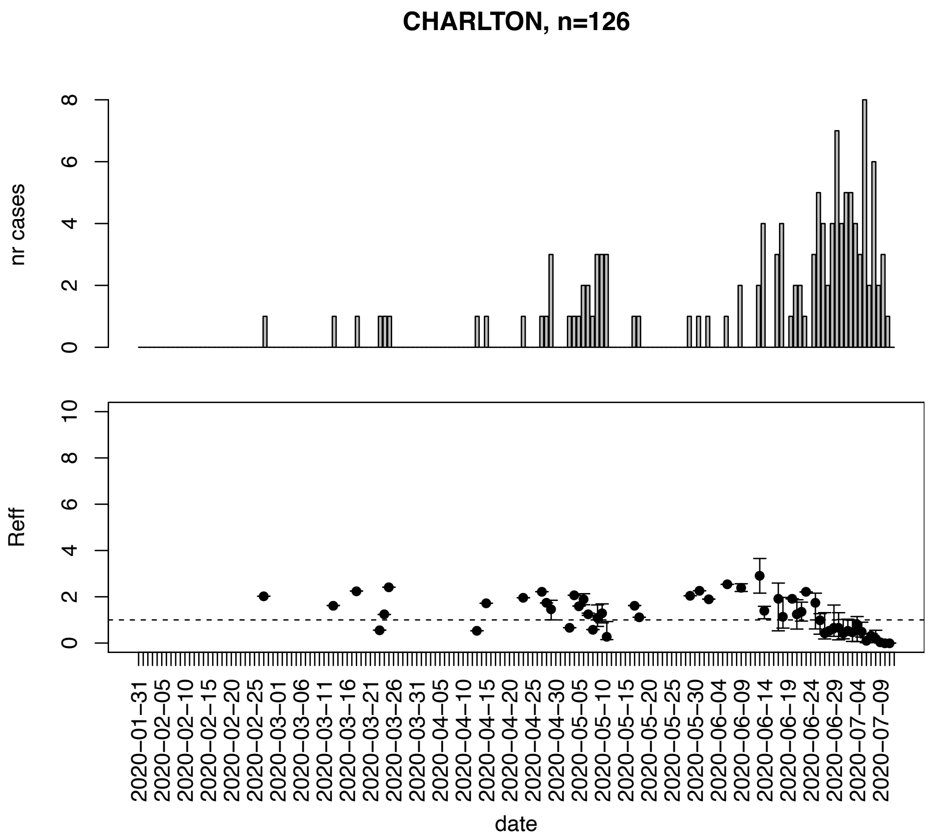

Appendix Figure 34. Epidemic curves and reproduction number estimates until July 13th in Charlton county.

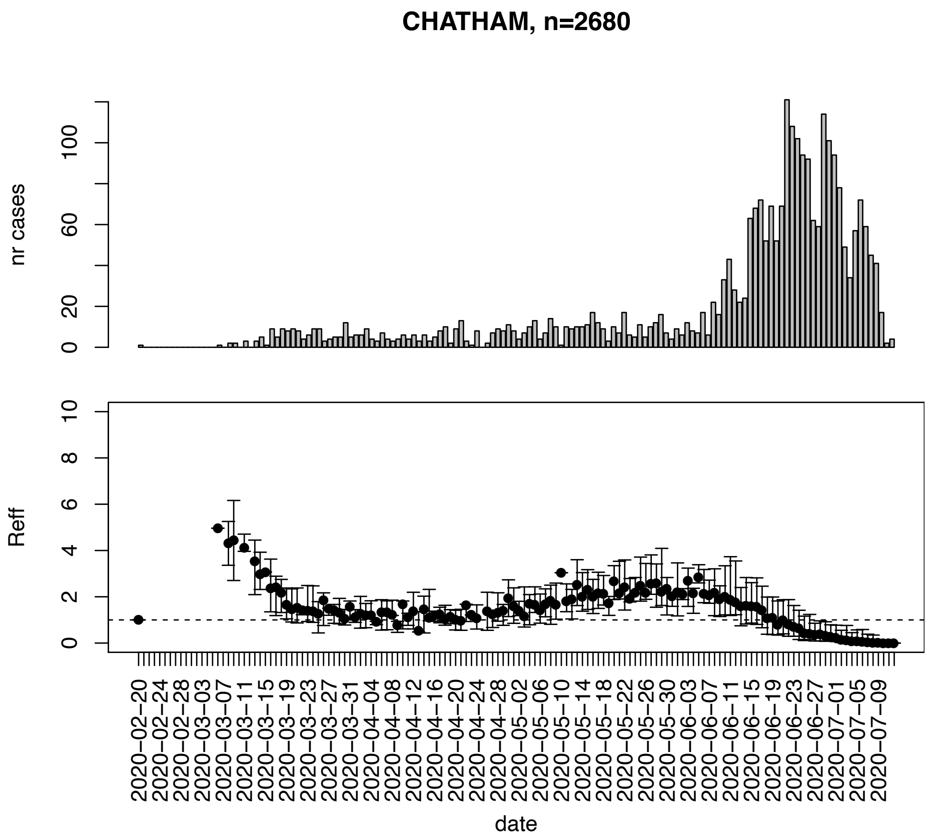

Appendix Figure 35. Epidemic curves and reproduction number estimates until July 13th in Chatham county.

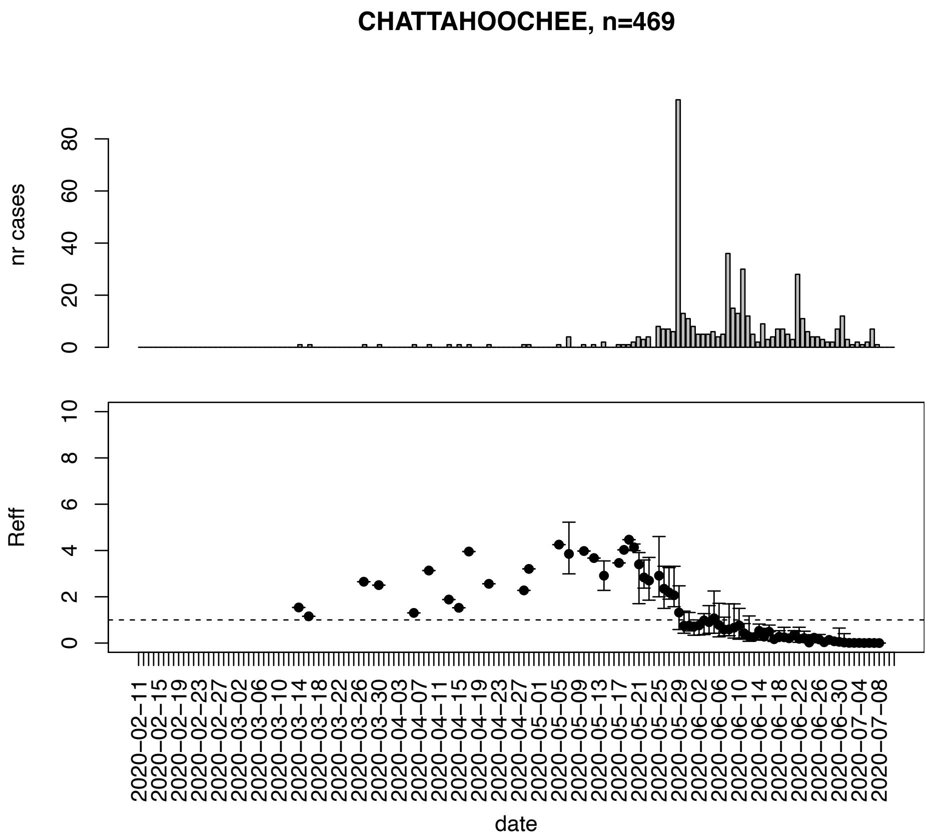

Appendix Figure 36. Epidemic curves and reproduction number estimates until July 13th in Chattahoochee county.

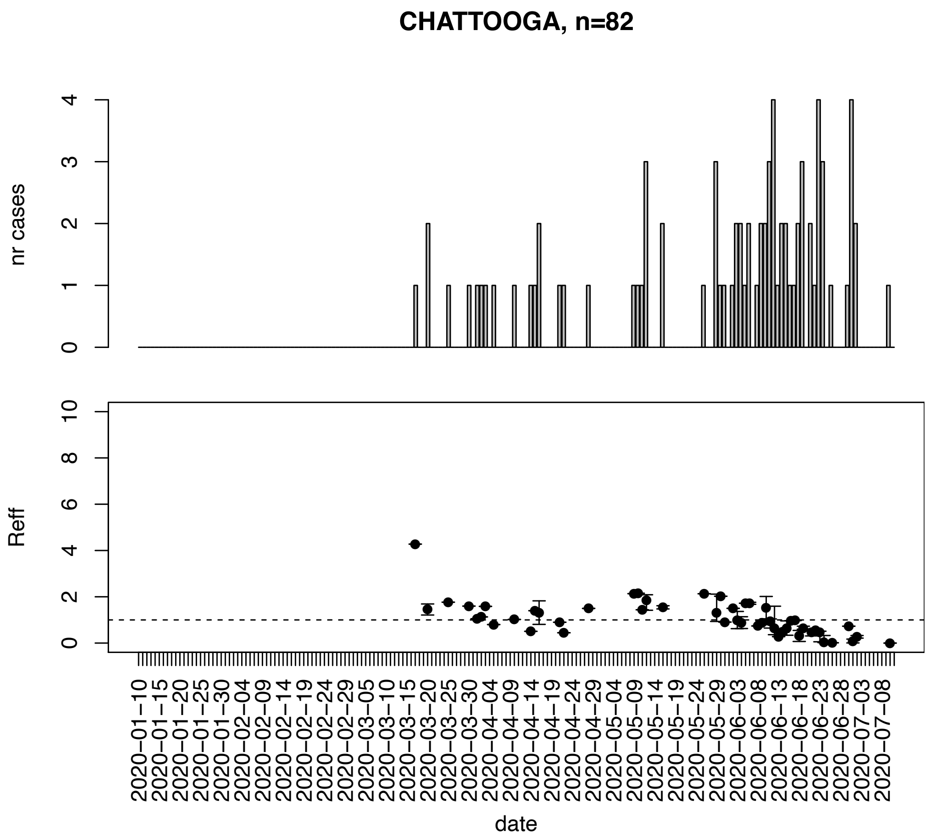

Appendix Figure 37. Epidemic curves and reproduction number estimates until July 13th in Chattooga county.

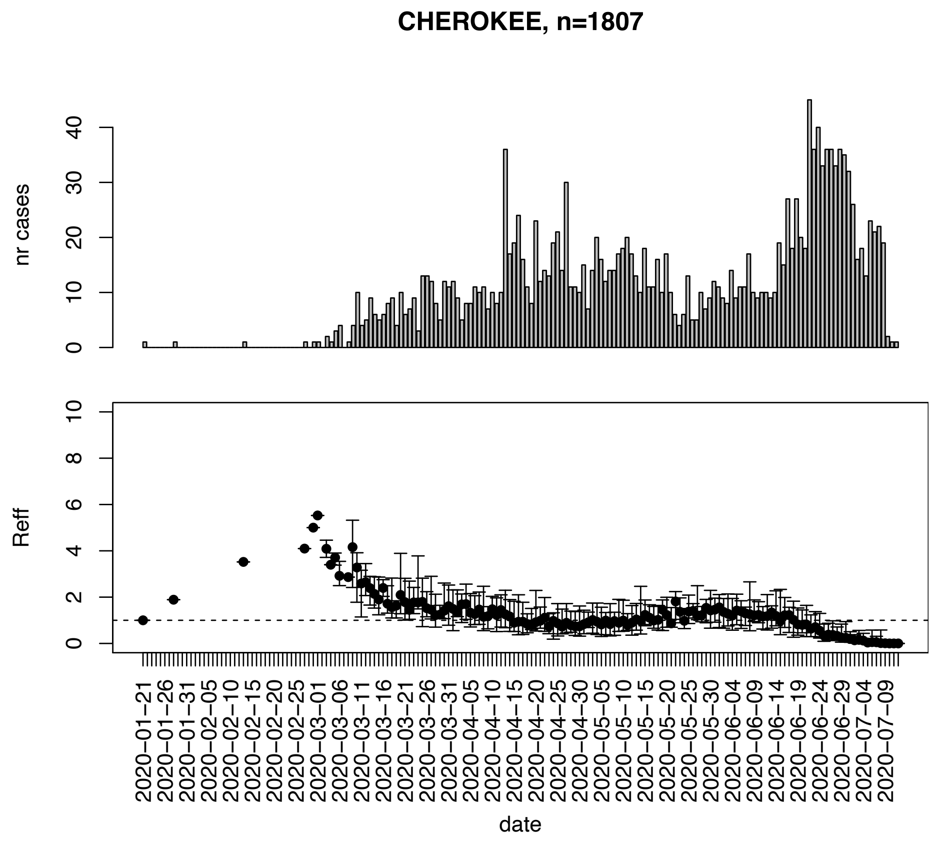

Appendix Figure 38. Epidemic curves and reproduction number estimates until July 13th in Cherokee county.

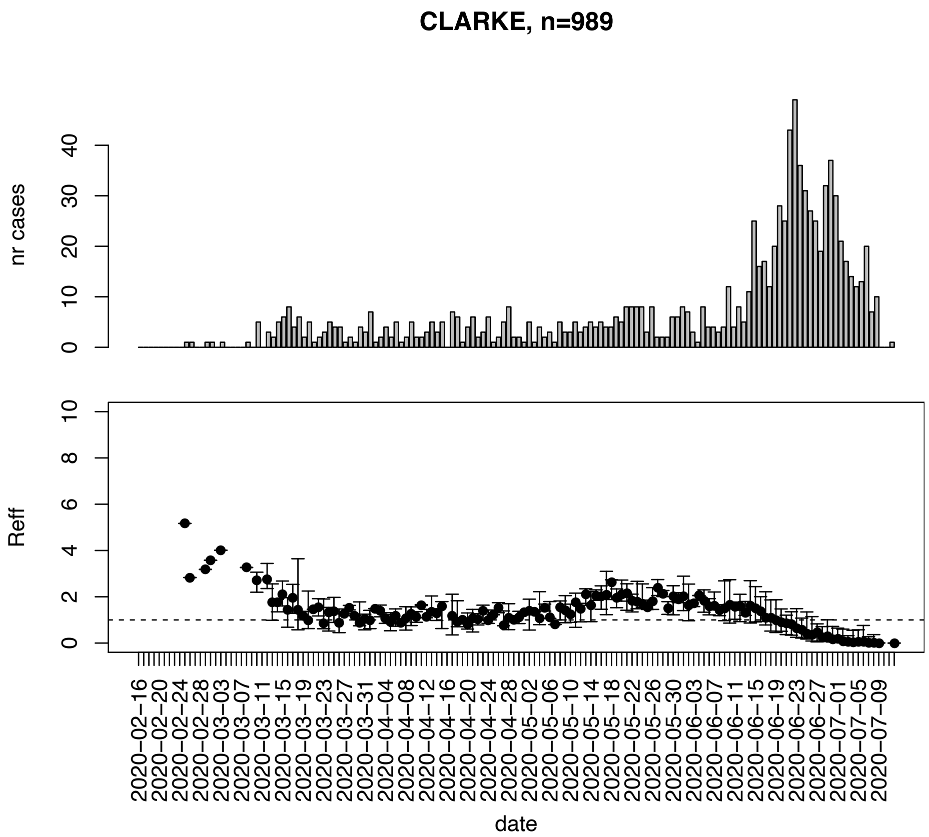

Appendix Figure 39. Epidemic curves and reproduction number estimates until July 13th in Clarke county.

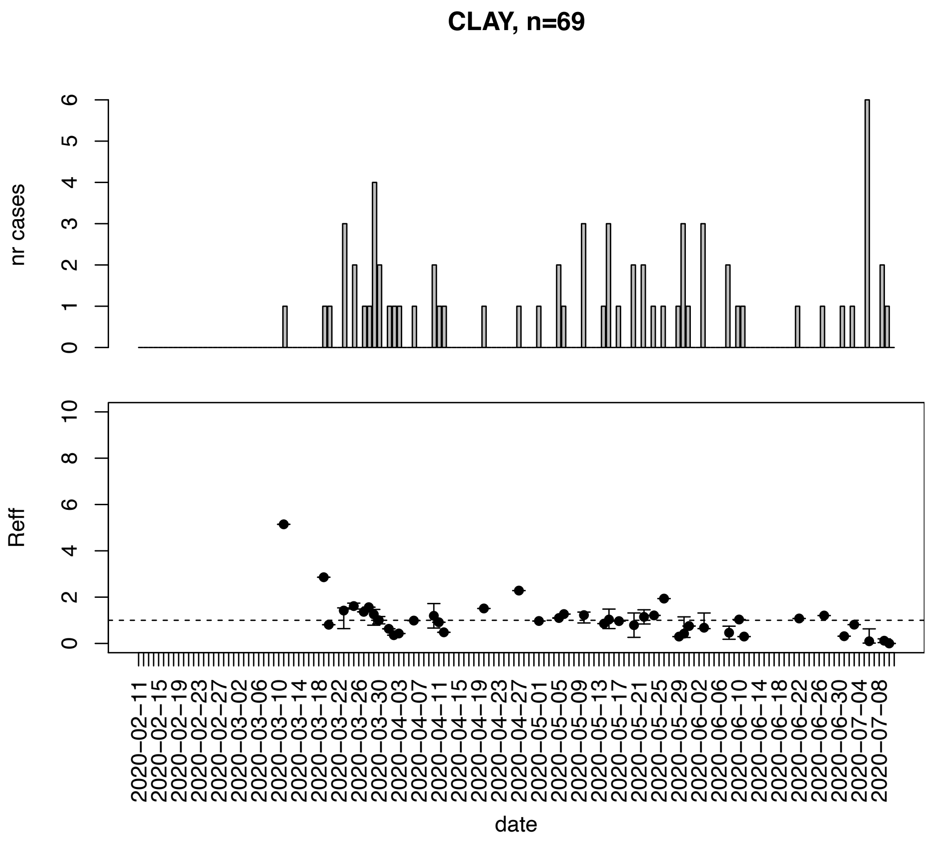

Appendix Figure 40. Epidemic curves and reproduction number estimates until July 13th in Clay county.

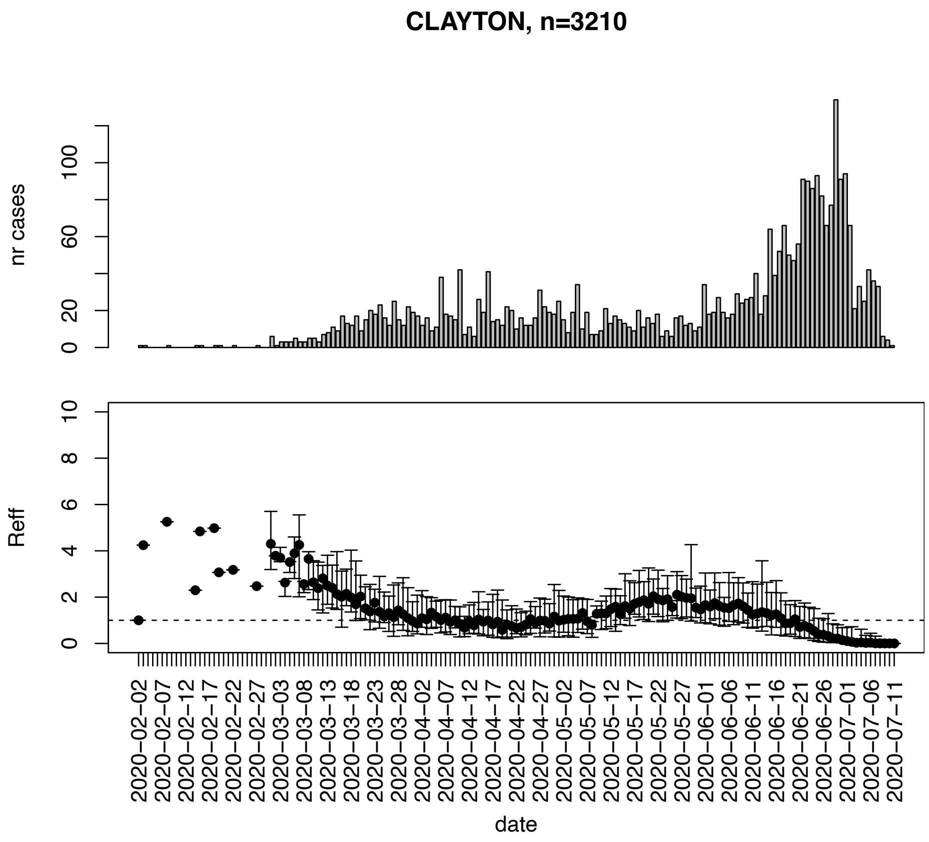

Appendix Figure 41. Epidemic curves and reproduction number estimates until July 13th in Clayton county.

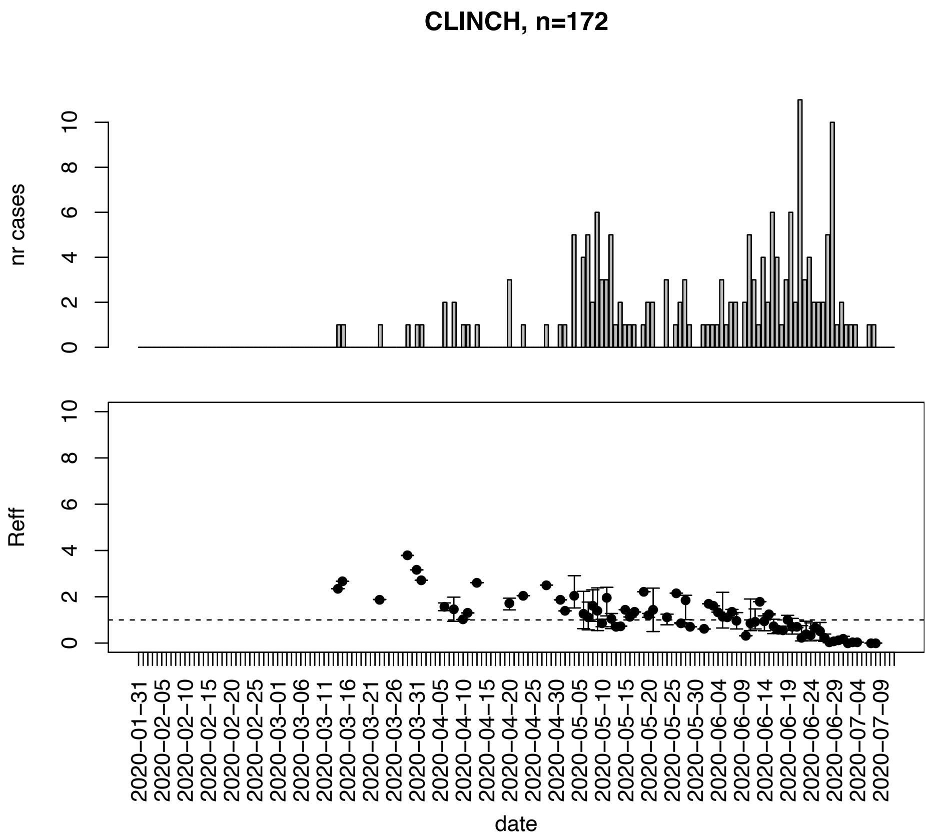

Appendix Figure 42. Epidemic curves and reproduction number estimates until July 13th in Clinch county.

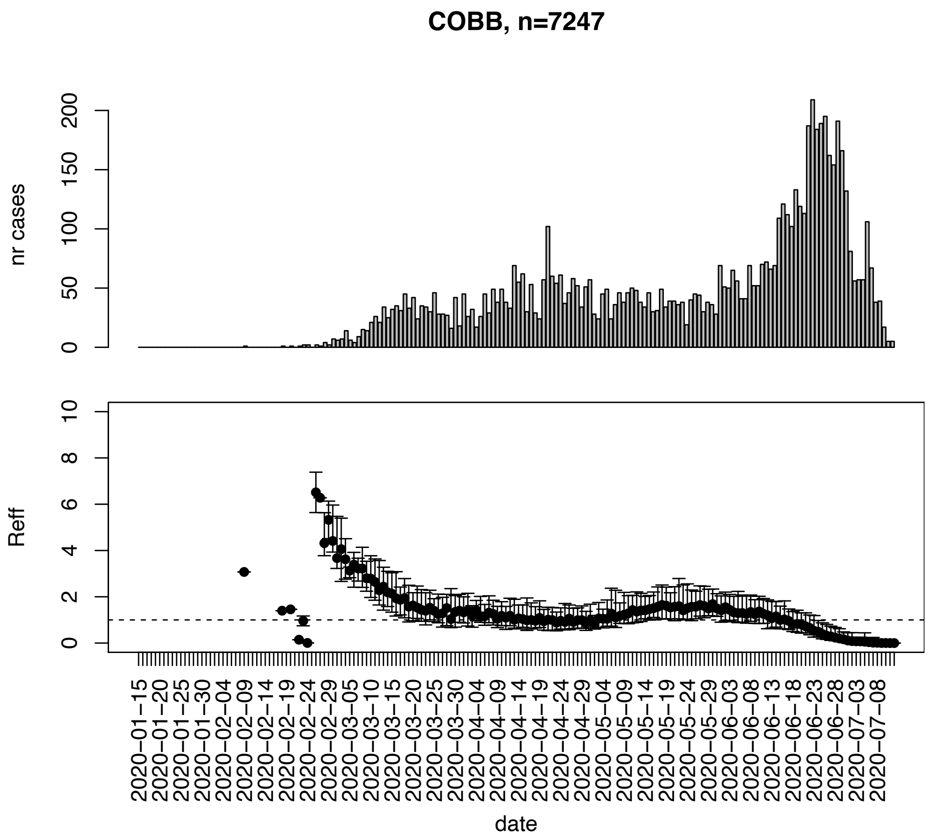

Appendix Figure 43. Epidemic curves and reproduction number estimates until July 13th in Cobb county.

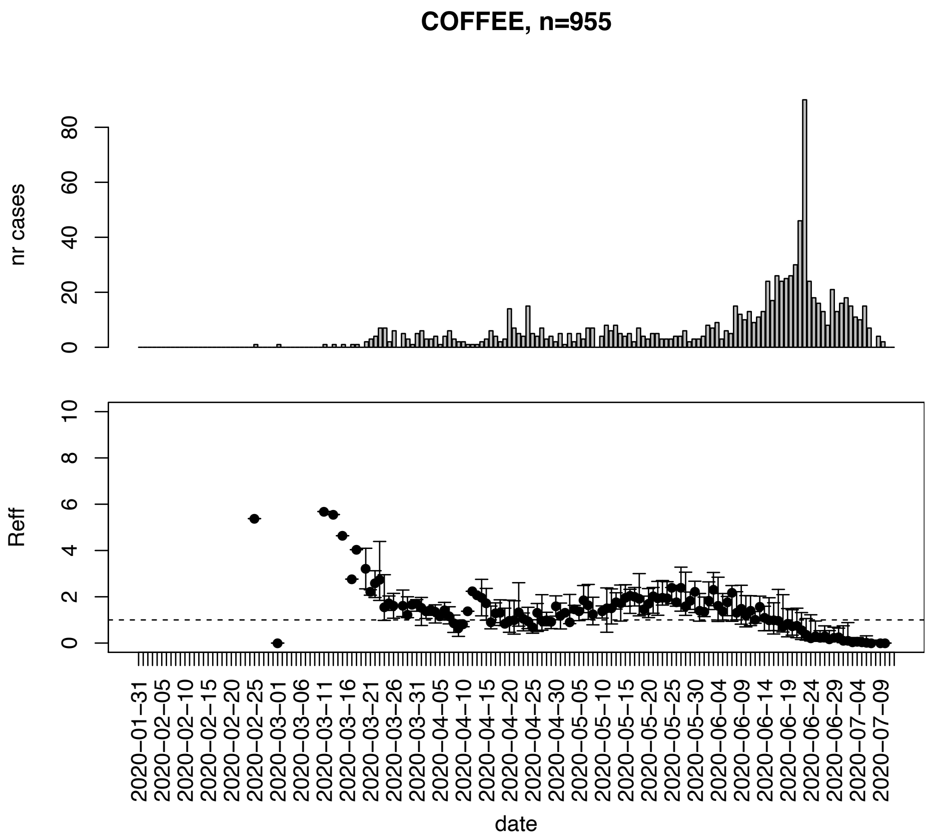

Appendix Figure 44. Epidemic curves and reproduction number estimates until July 13th in Coffee county.

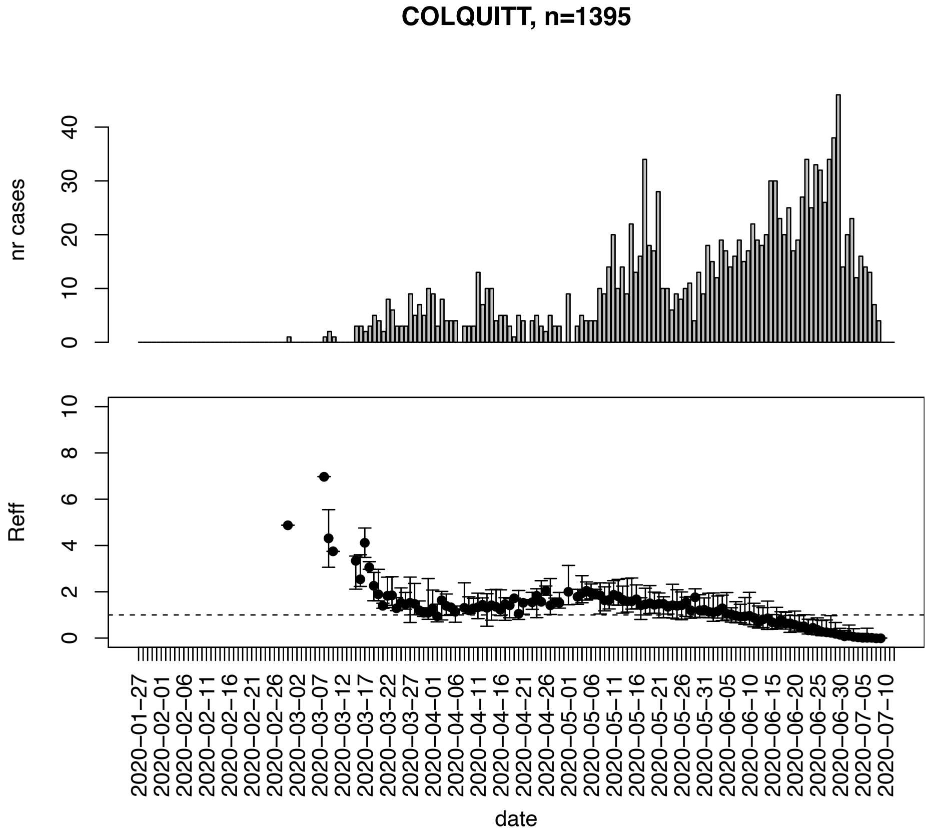

Appendix Figure 45. Epidemic curves and reproduction number estimates until July 13th in Colquitt county.

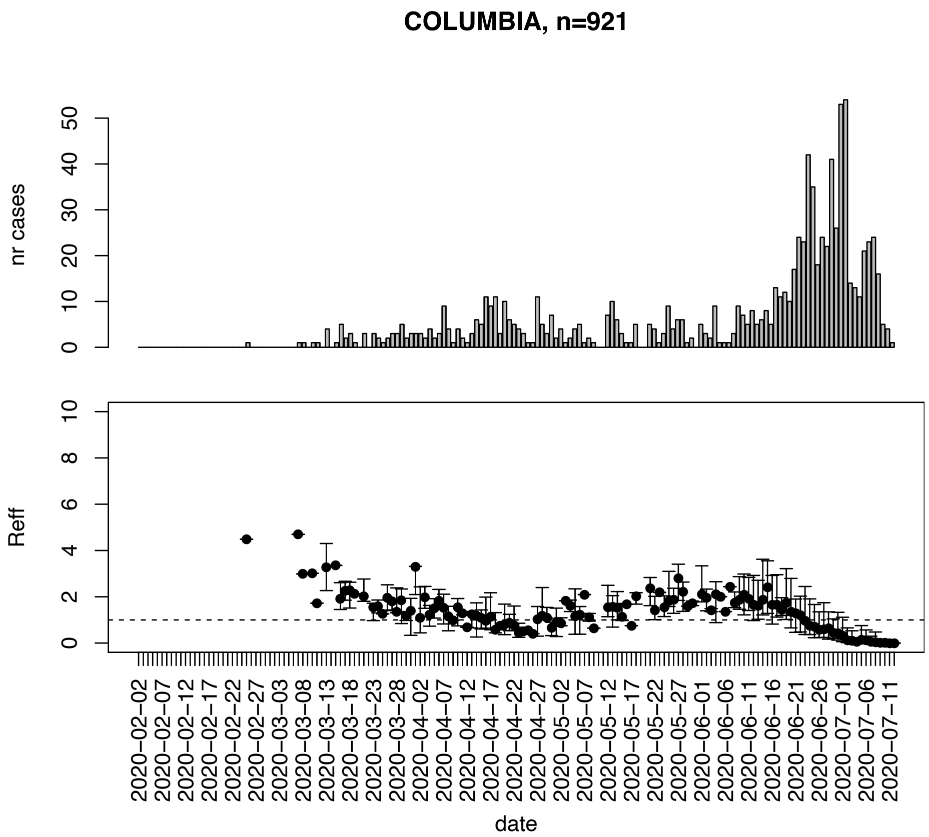

Appendix Figure 46. Epidemic curves and reproduction number estimates until July 13th in Columbia county.

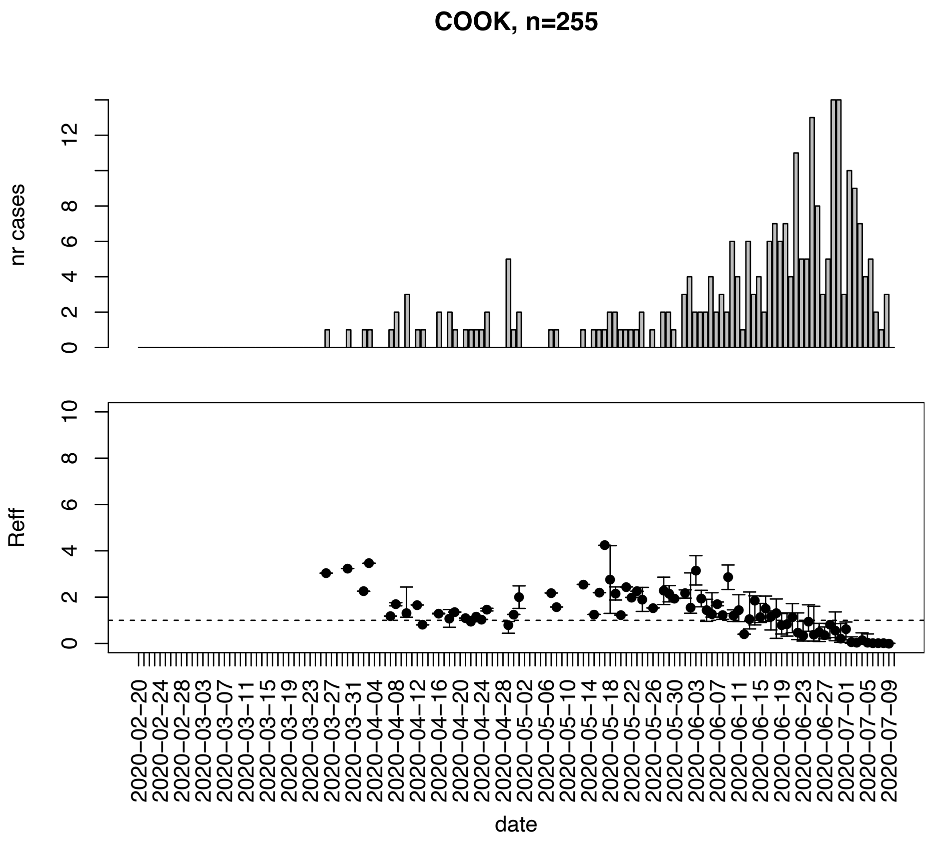

Appendix Figure 47. Epidemic curves and reproduction number estimates until July 13th in Cook county.

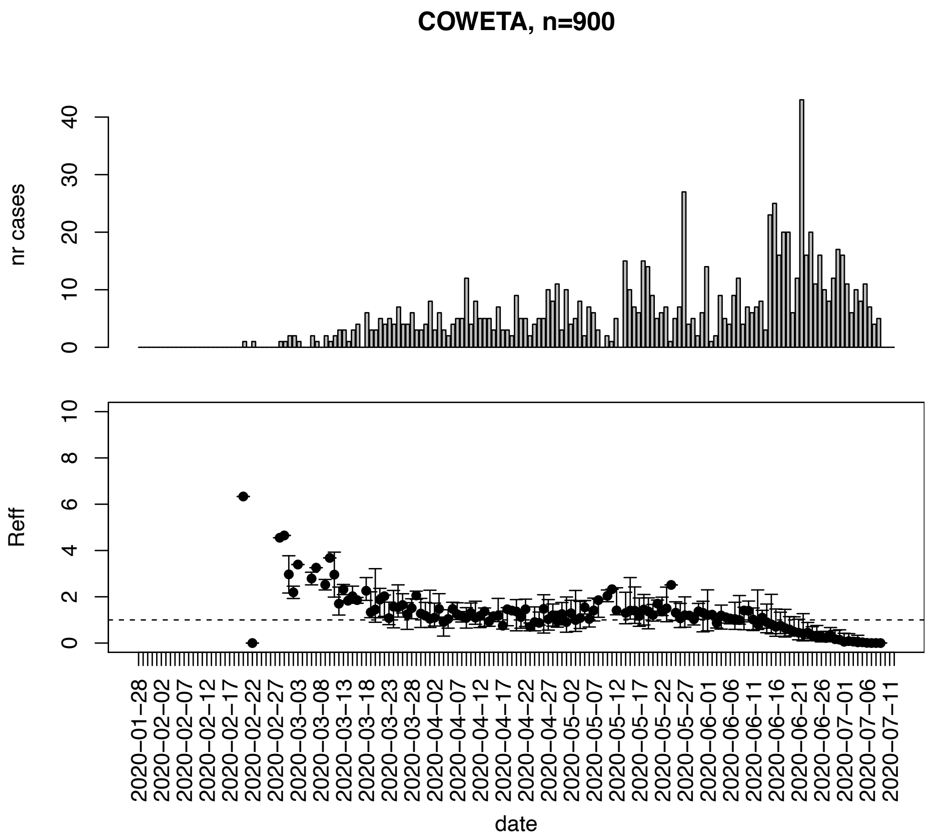

Appendix Figure 48. Epidemic curves and reproduction number estimates until July 13th in Coweta county.

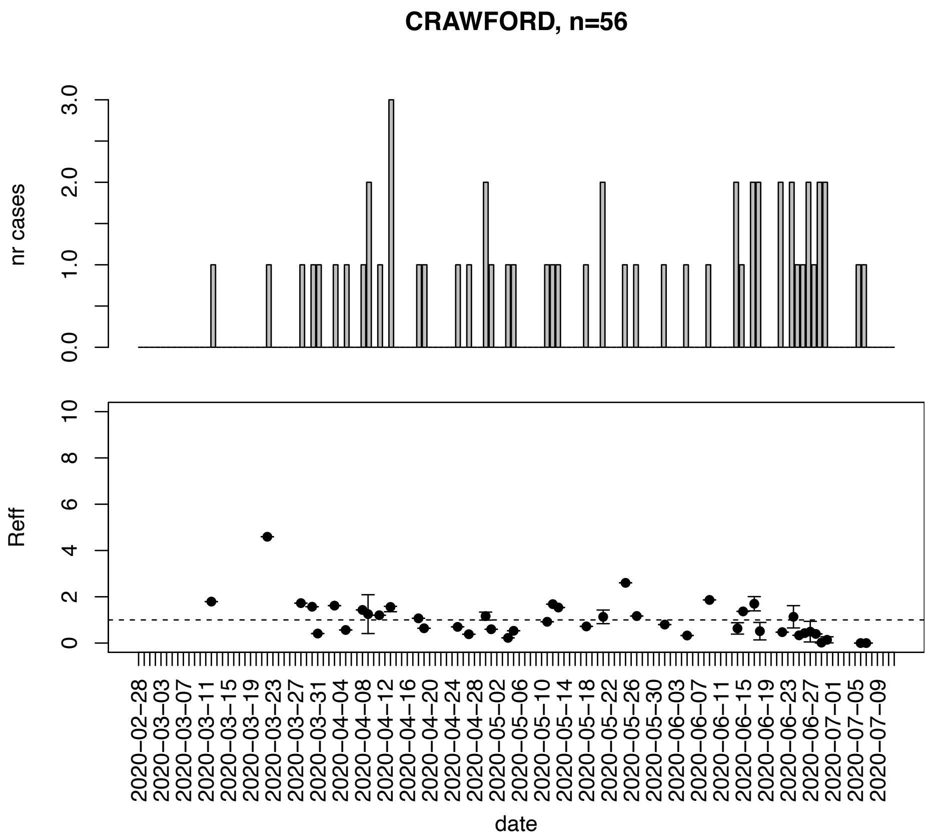

Appendix Figure 49. Epidemic curves and reproduction number estimates until July 13th in Crawford county.

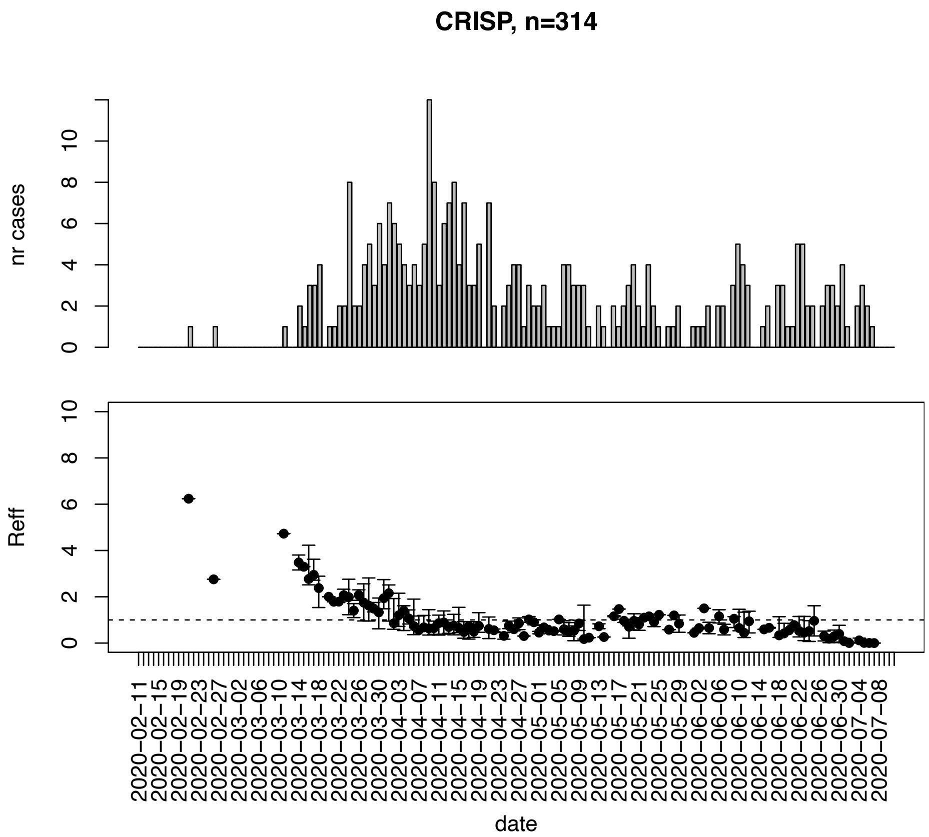

Appendix Figure 50. Epidemic curves and reproduction number estimates until July 13th in Crisp county.

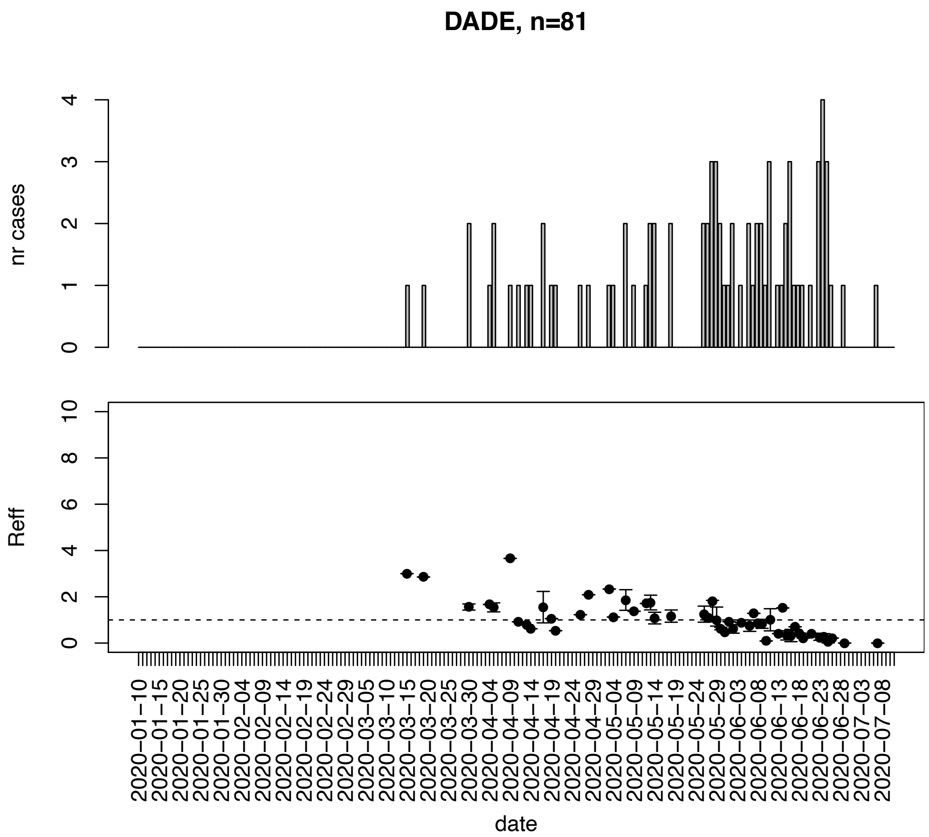

Appendix Figure 51. Epidemic curves and reproduction number estimates until July 13th in Dade county.

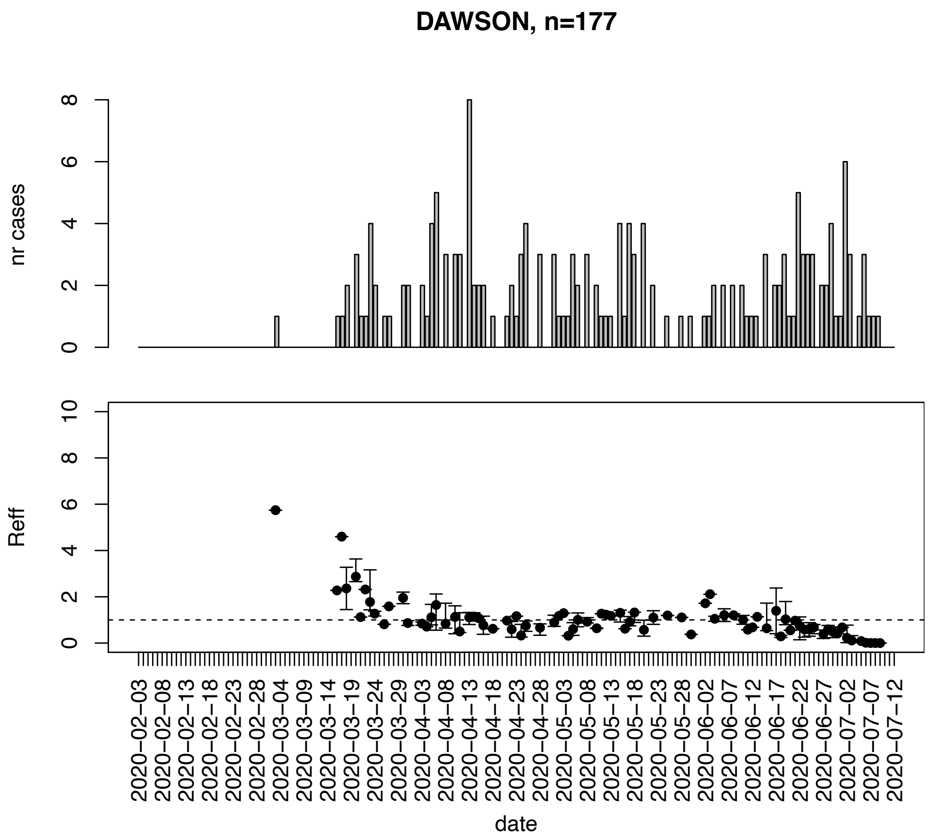

Appendix Figure 52. Epidemic curves and reproduction number estimates until July 13th in Dawson county.

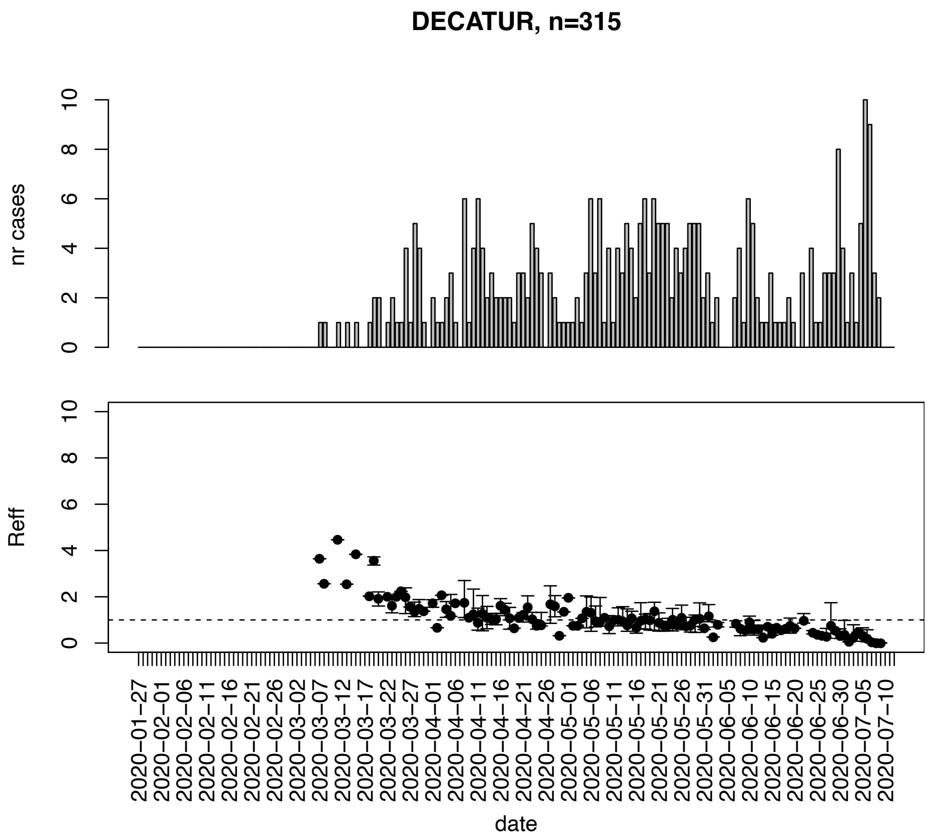

Appendix Figure 53. Epidemic curves and reproduction number estimates until July 13th in Decatur county.

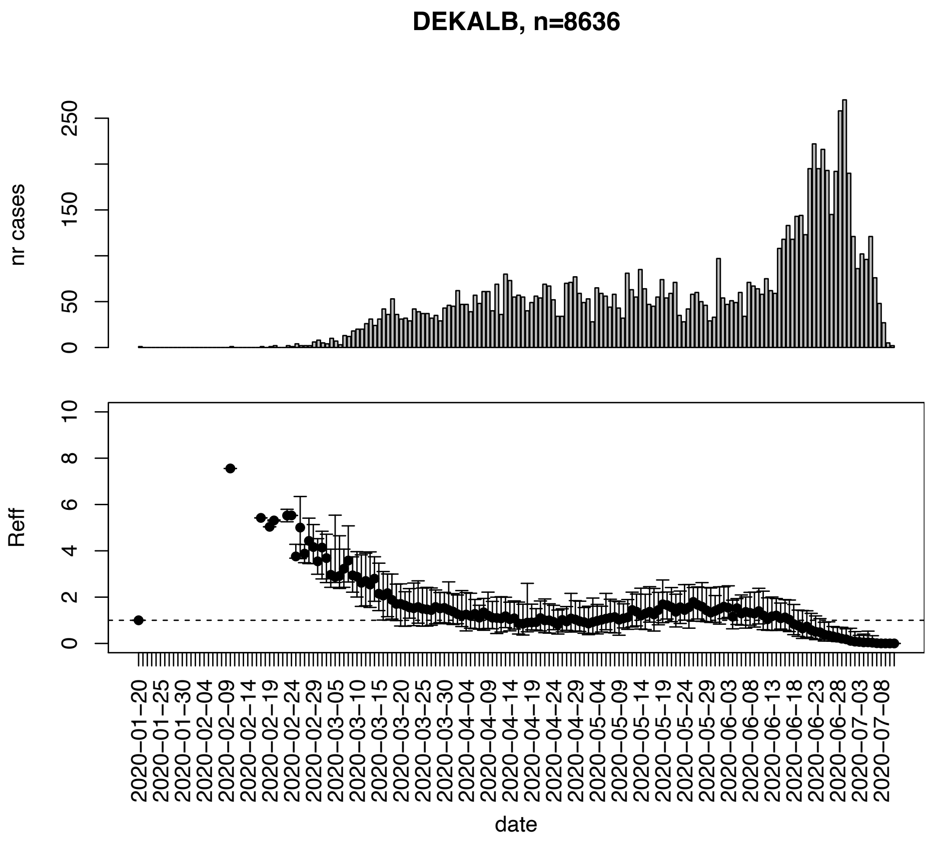

Appendix Figure 54. Epidemic curves and reproduction number estimates until July 13th in Dekalb county.

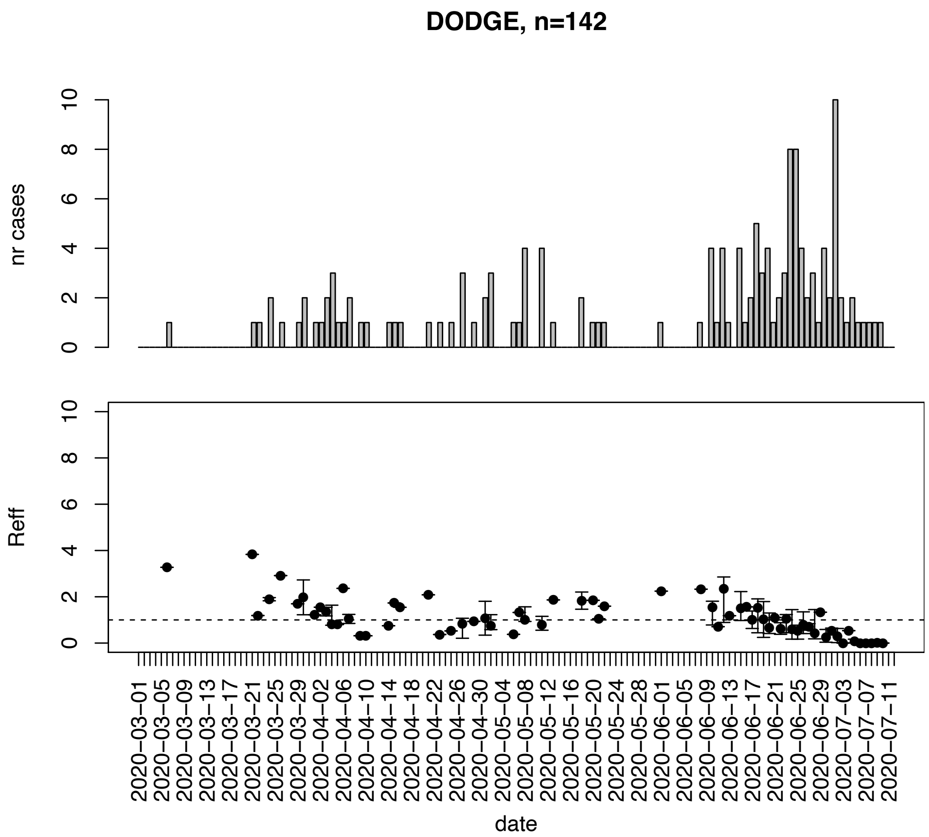

Appendix Figure 55. Epidemic curves and reproduction number estimates until July 13th in Dodge county.

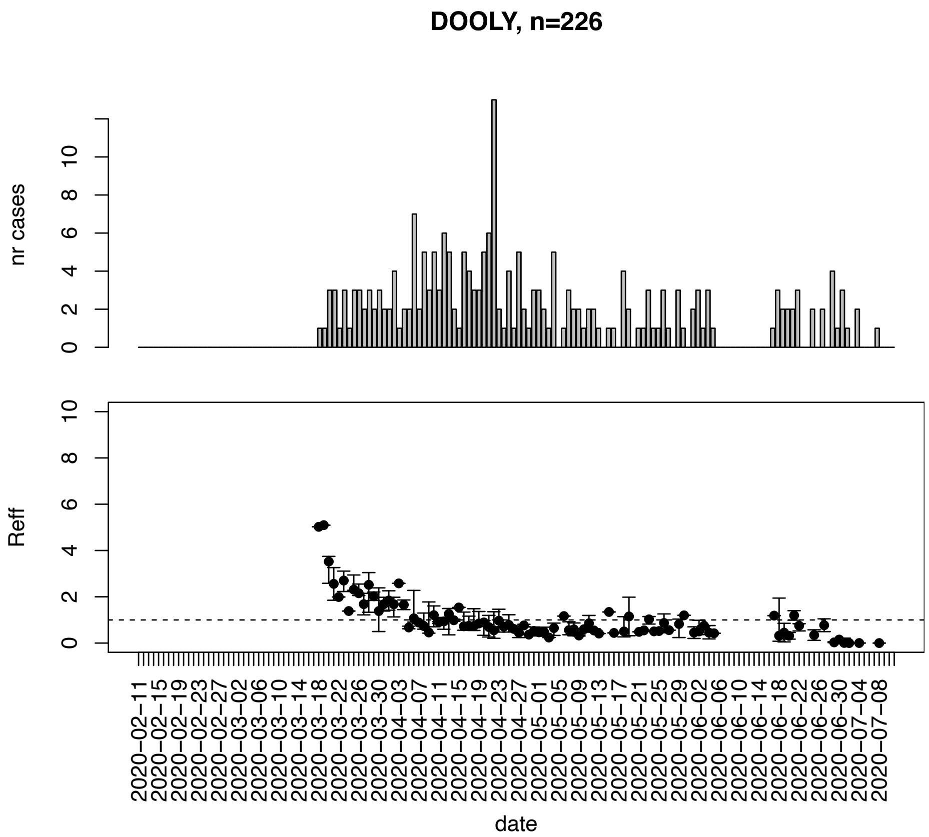

Appendix Figure 56. Epidemic curves and reproduction number estimates until July 13th in Dooly county.

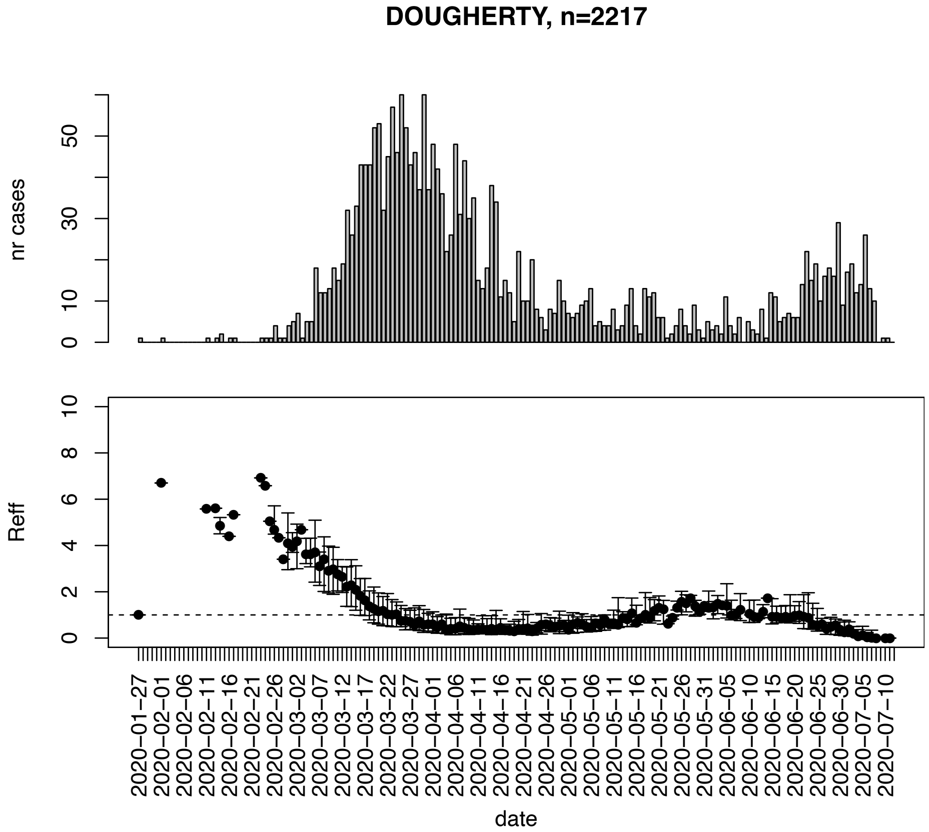

Appendix Figure 57. Epidemic curves and reproduction number estimates until July 13th in Dougherty county.

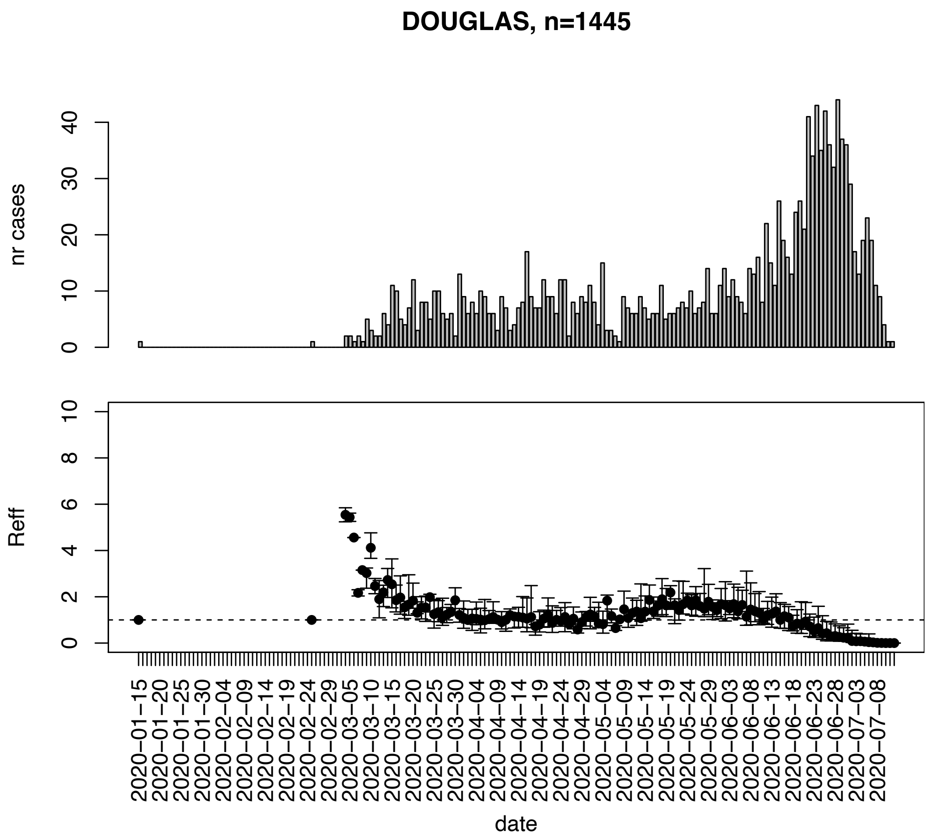

Appendix Figure 58. Epidemic curves and reproduction number estimates until July 13th in Douglas county.

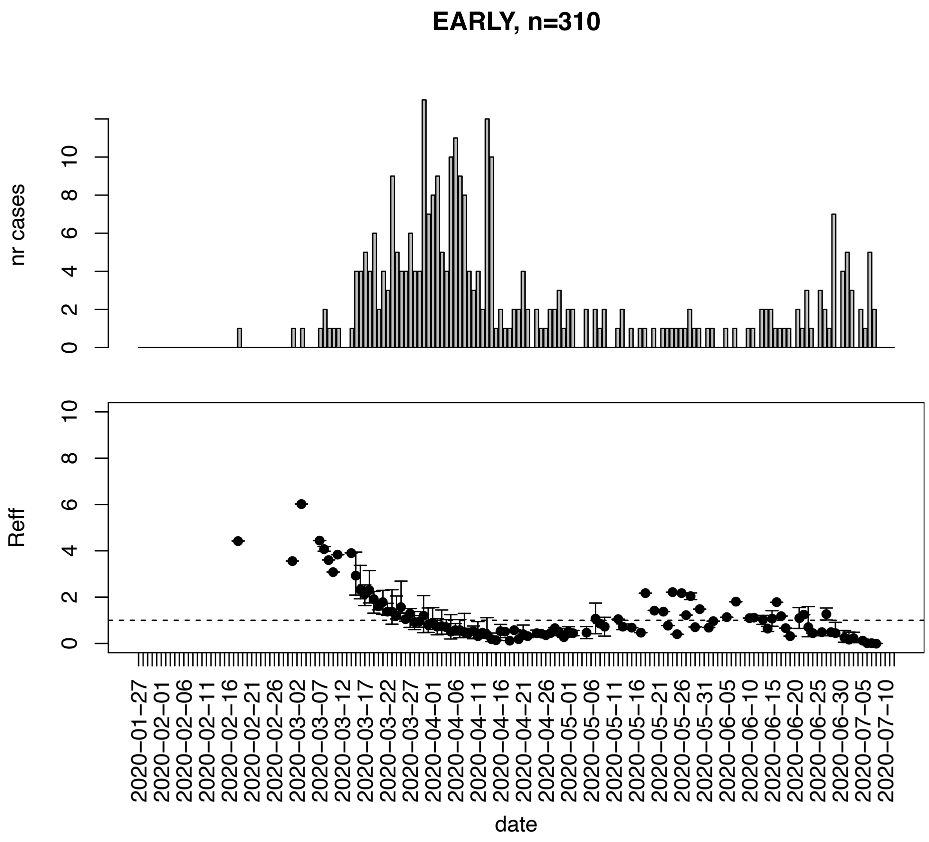

Appendix Figure 59. Epidemic curves and reproduction number estimates until July 13th in Early county.

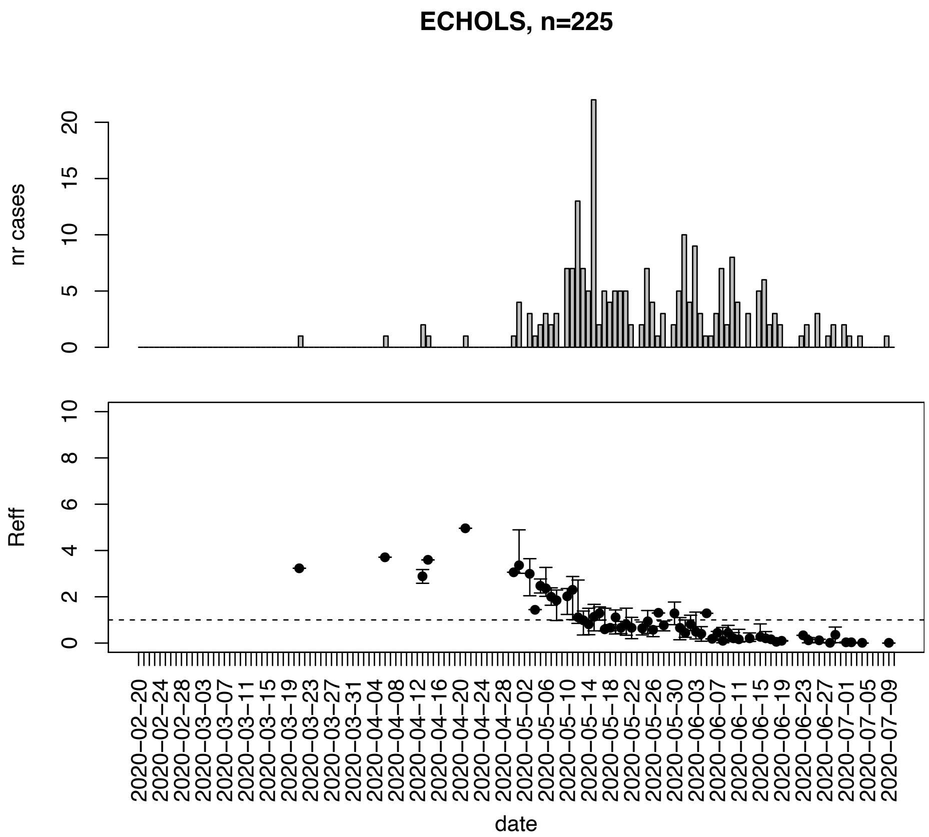

Appendix Figure 60. Epidemic curves and reproduction number estimates until July 13th in Echols county.

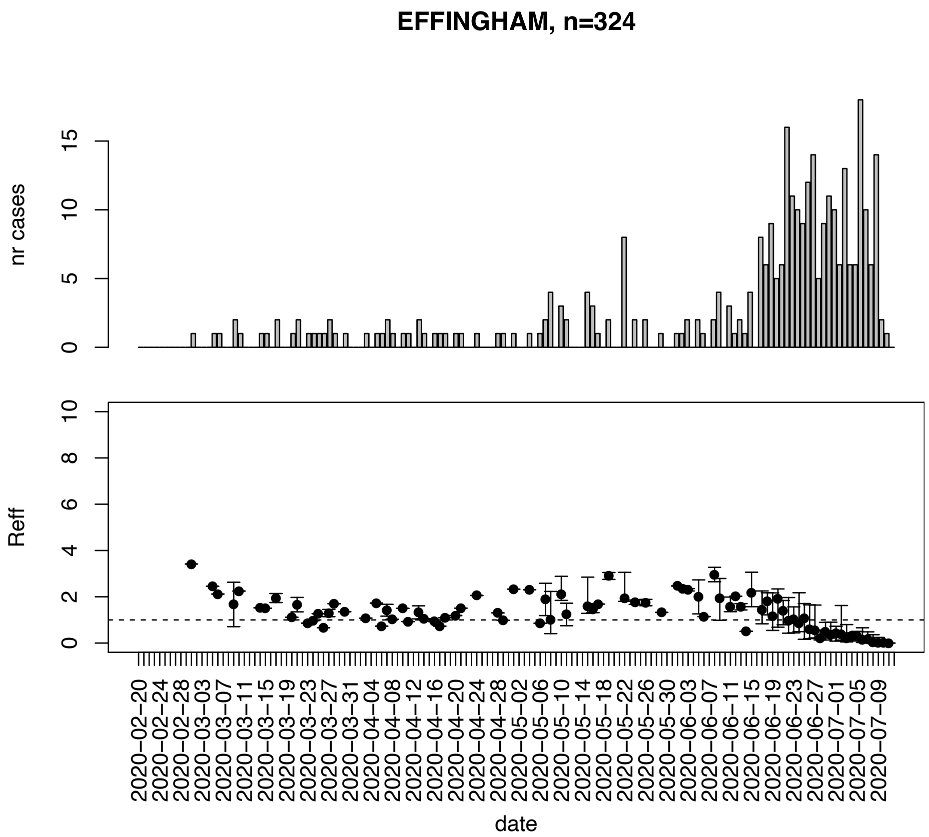

Appendix Figure 61. Epidemic curves and reproduction number estimates until July 13th in Effingham county.

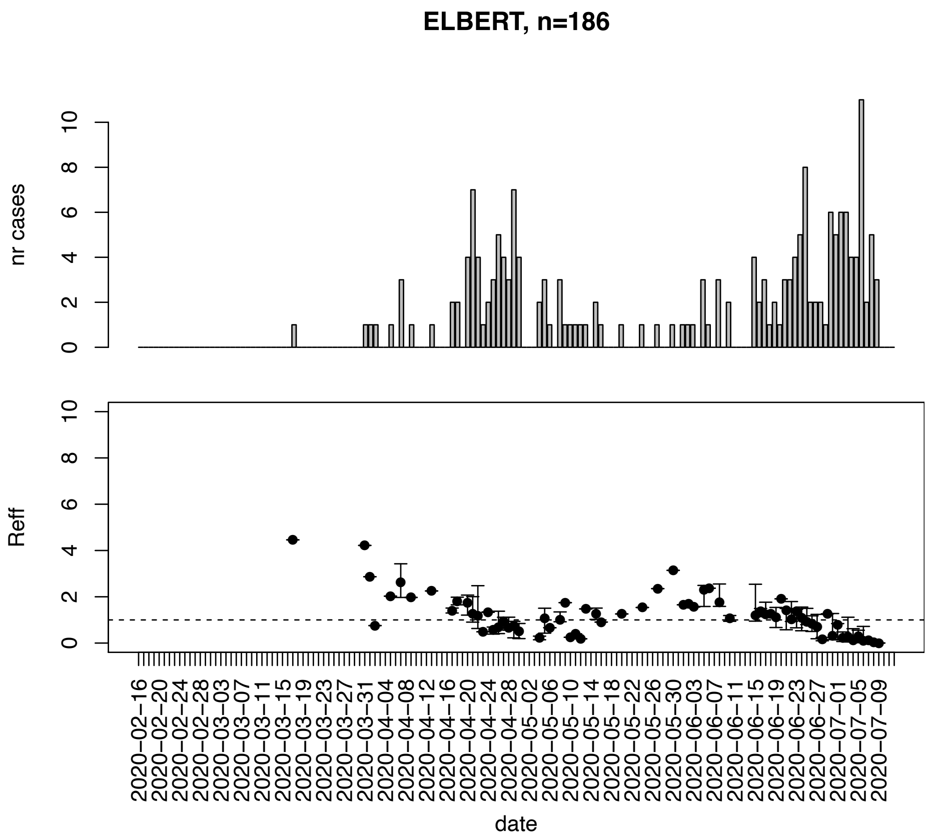

Appendix Figure 62. Epidemic curves and reproduction number estimates until July 13th in Elbert county.

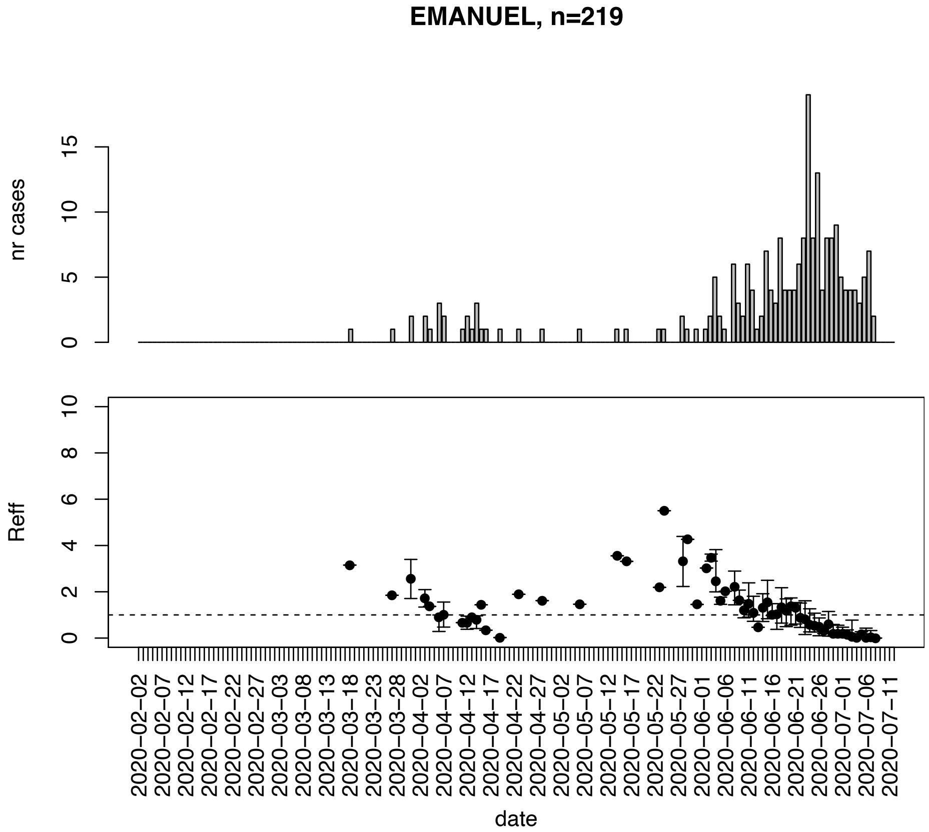

Appendix Figure 63. Epidemic curves and reproduction number estimates until July 13th in Emanuel county.

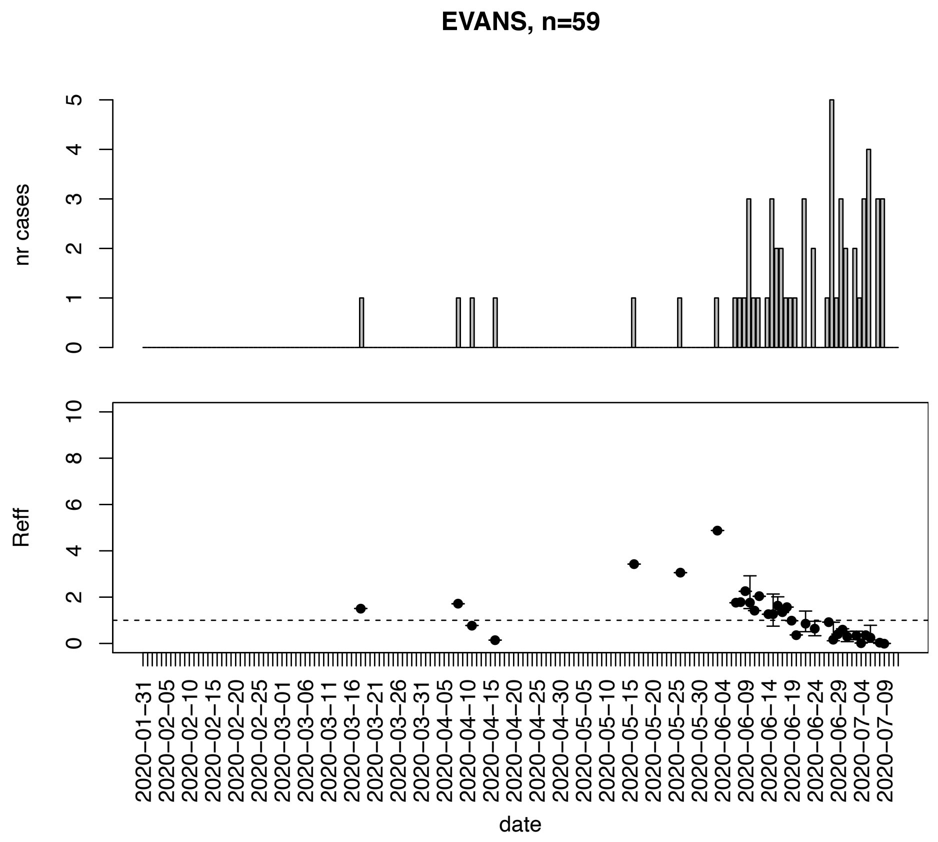

Appendix Figure 64. Epidemic curves and reproduction number estimates until July 13th in Evans county.

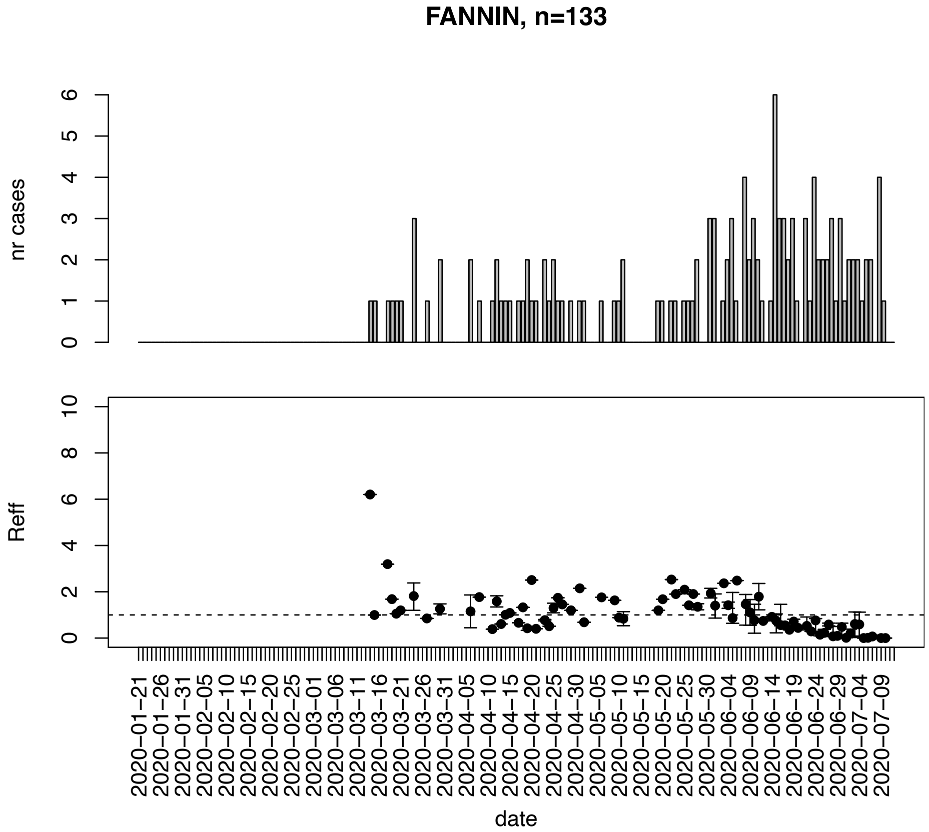

Appendix Figure 65. Epidemic curves and reproduction number estimates until July 13th in Fannin county.

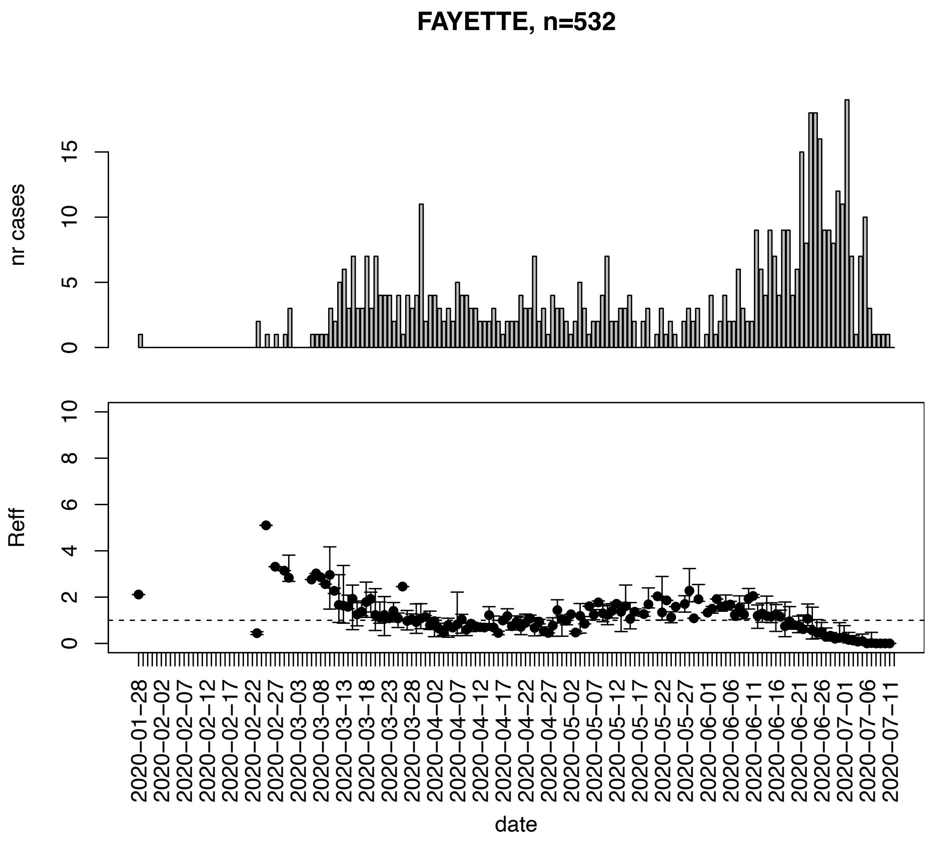

Appendix Figure 66. Epidemic curves and reproduction number estimates until July 13th in Fayette county.

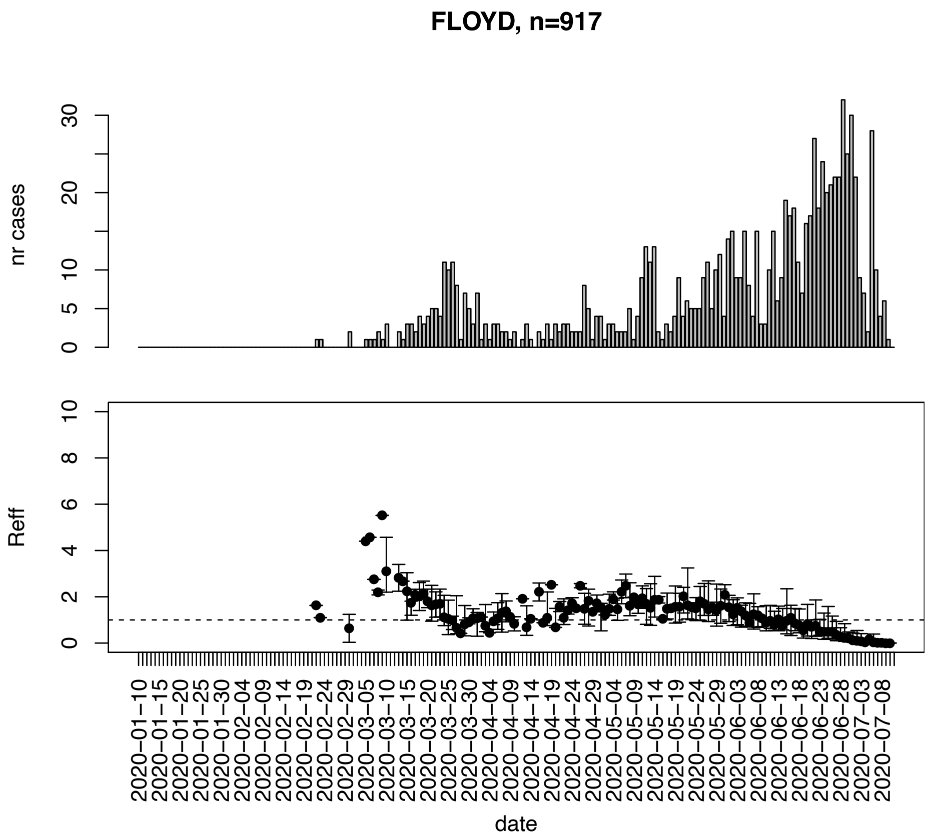

Appendix Figure 67. Epidemic curves and reproduction number estimates until July 13th in Floyd county.

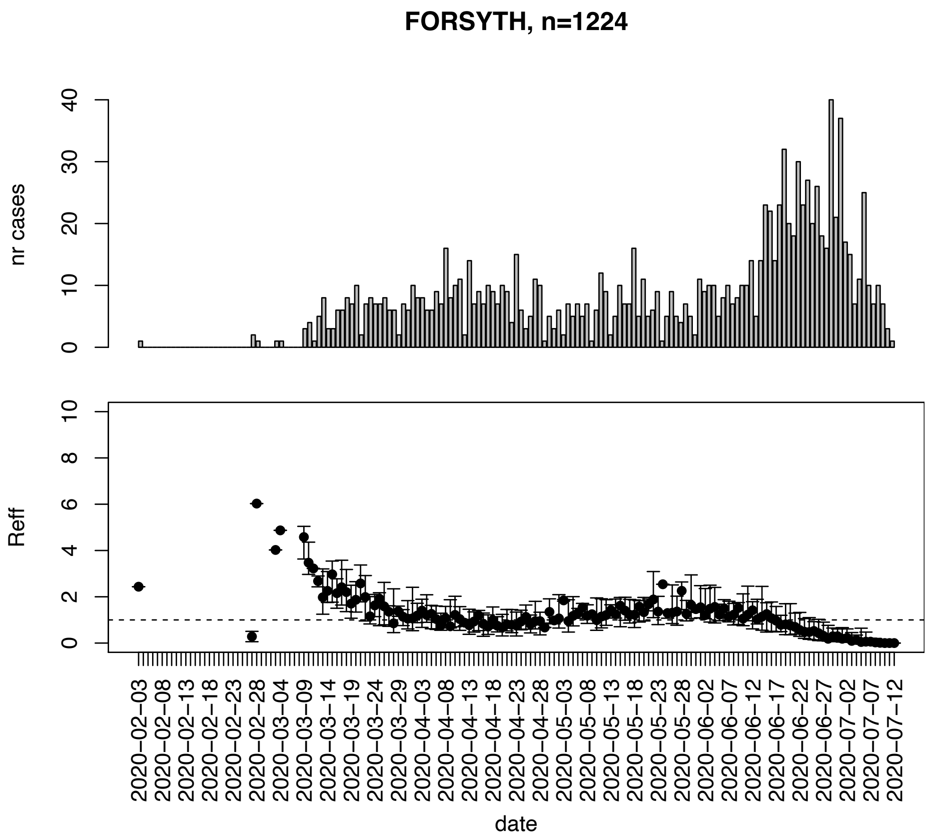

Appendix Figure 68. Epidemic curves and reproduction number estimates until July 13th in Forsyth county.

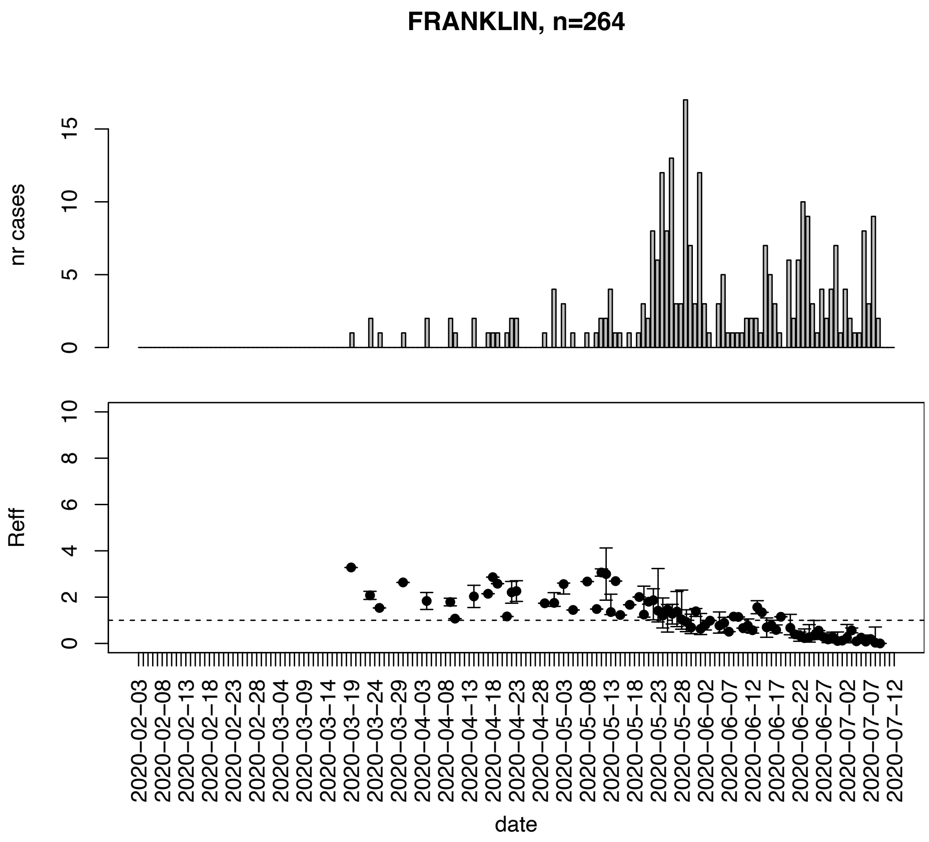

Appendix Figure 69. Epidemic curves and reproduction number estimates until July 13th in Franklin county.

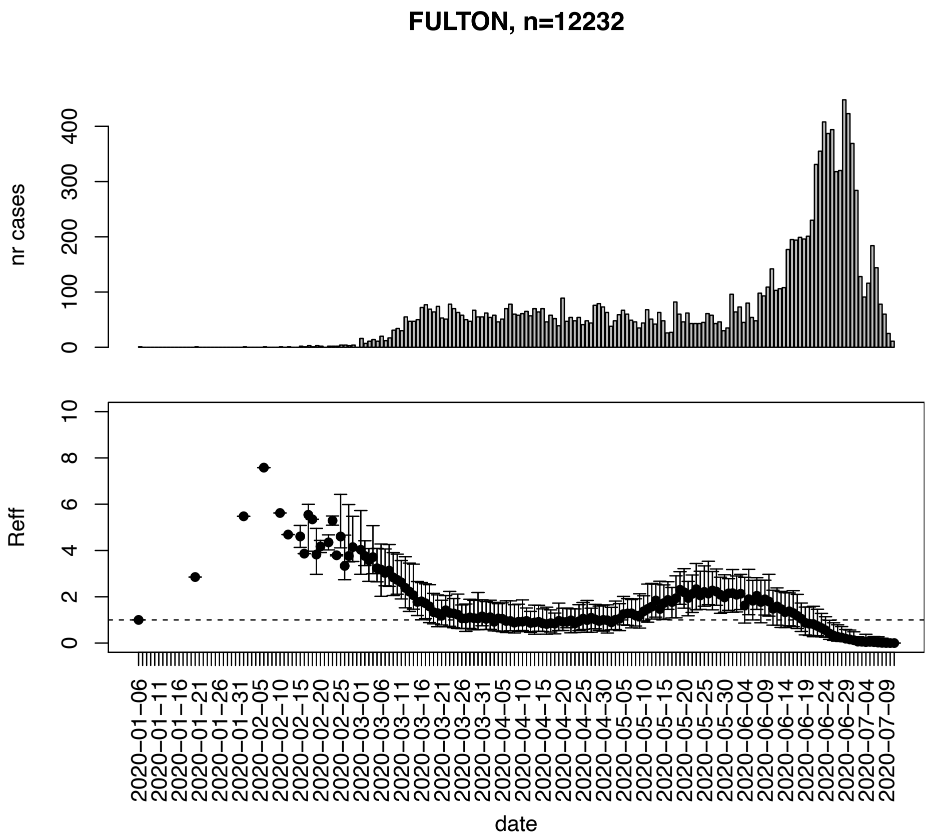

Appendix Figure 70. Epidemic curves and reproduction number estimates until July 13th in Fulton county.

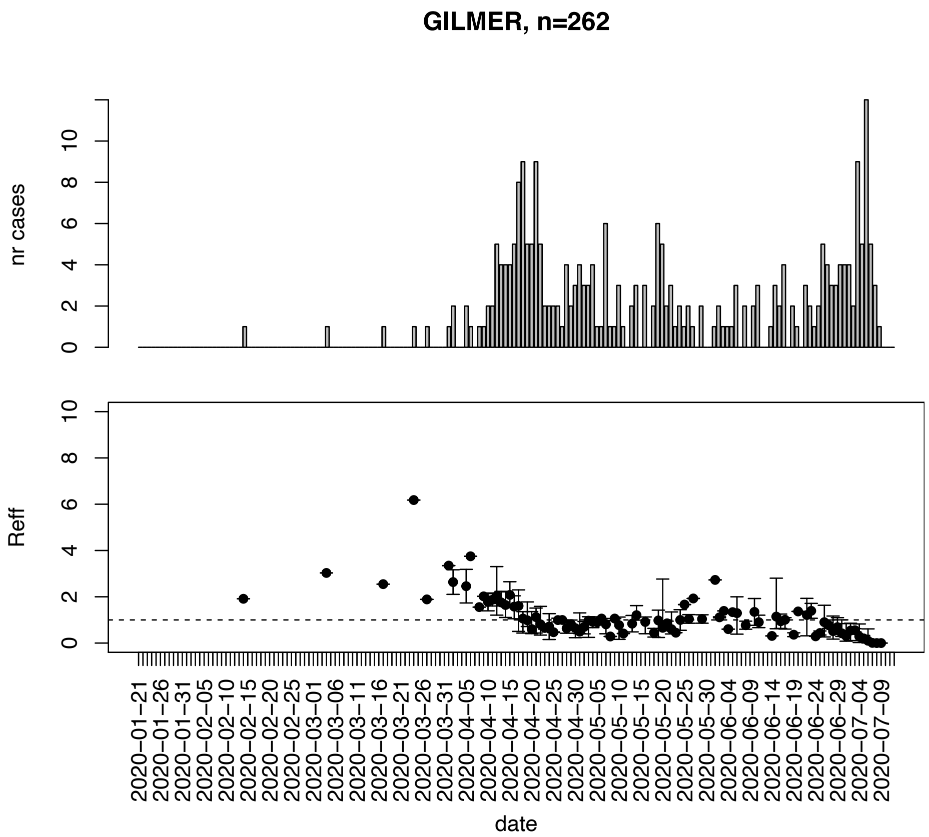

Appendix Figure 71. Epidemic curves and reproduction number estimates until July 13th in Gilmer county.

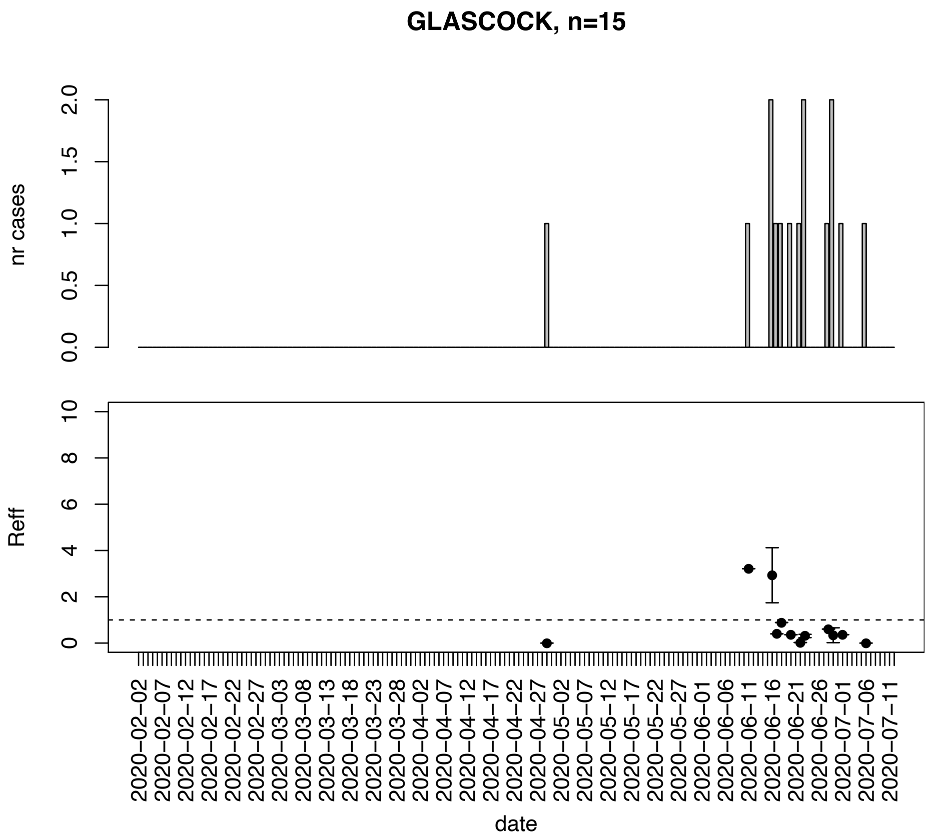

Appendix Figure 72. Epidemic curves and reproduction number estimates until July 13th in Glascock county.

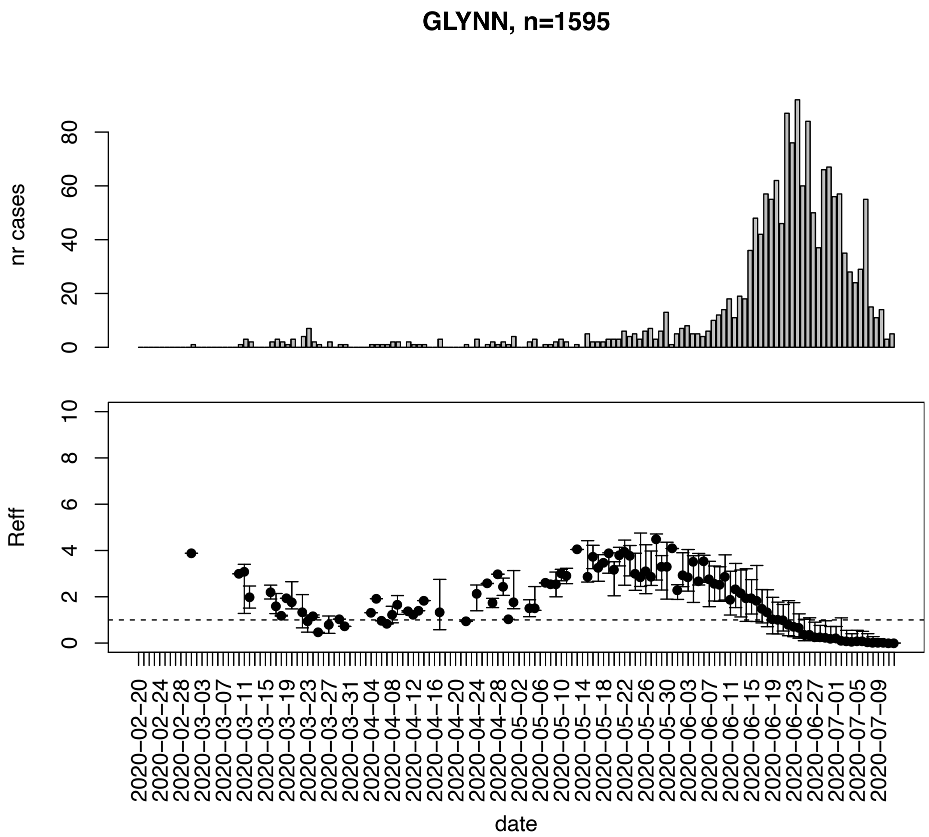

Appendix Figure 73. Epidemic curves and reproduction number estimates until July 13th in Glynn county.

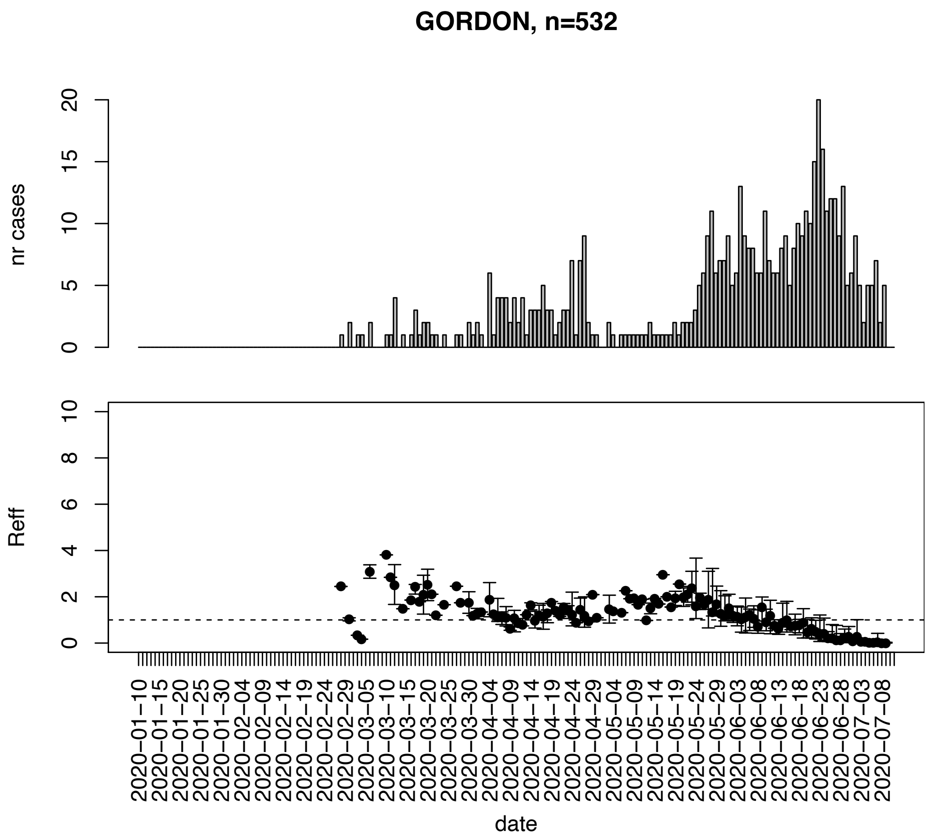

Appendix Figure 74. Epidemic curves and reproduction number estimates until July 13th in Gordon county.

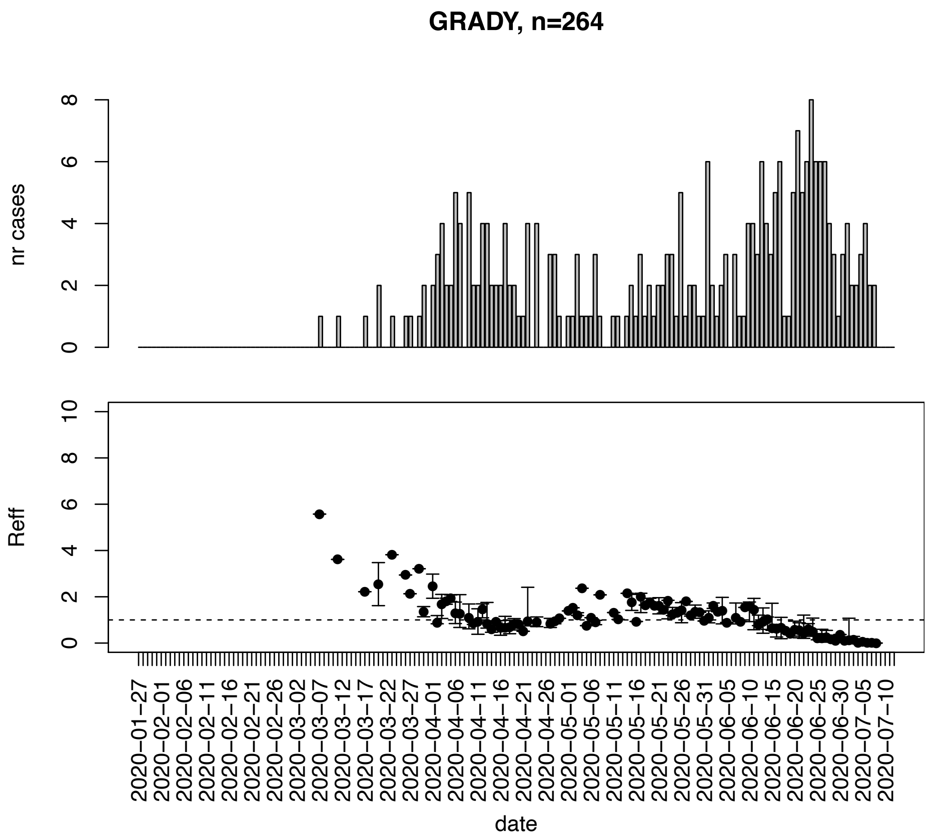

Appendix Figure 75. Epidemic curves and reproduction number estimates until July 13th in Grady county.

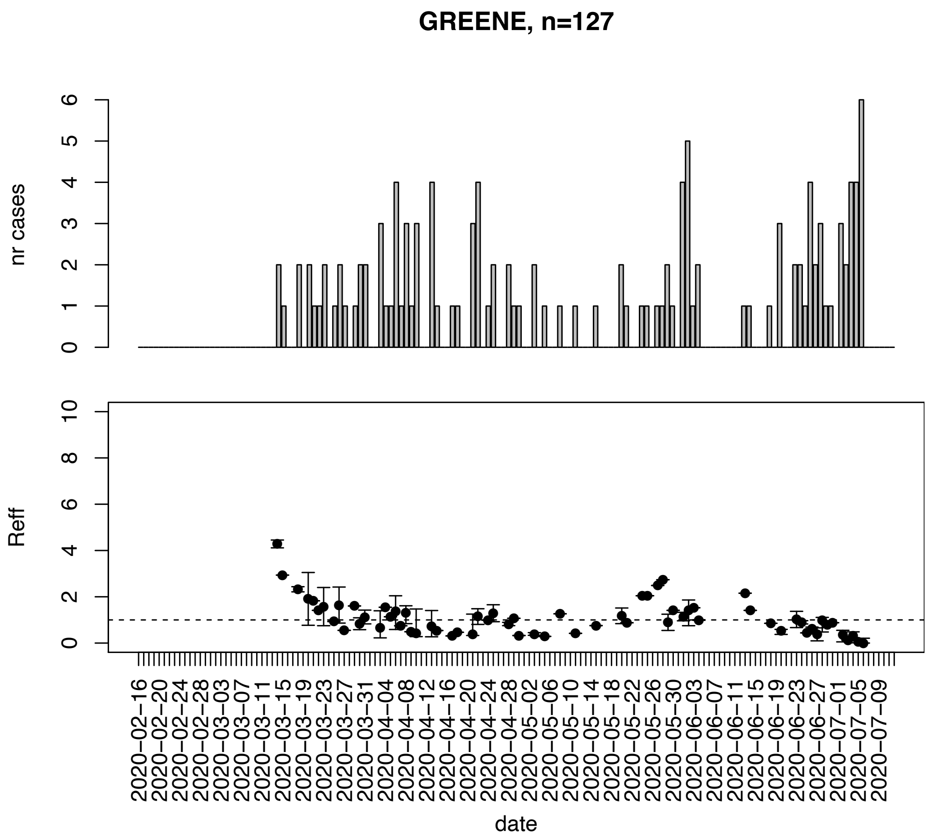

Appendix Figure 76. Epidemic curves and reproduction number estimates until July 13th in Greene county.

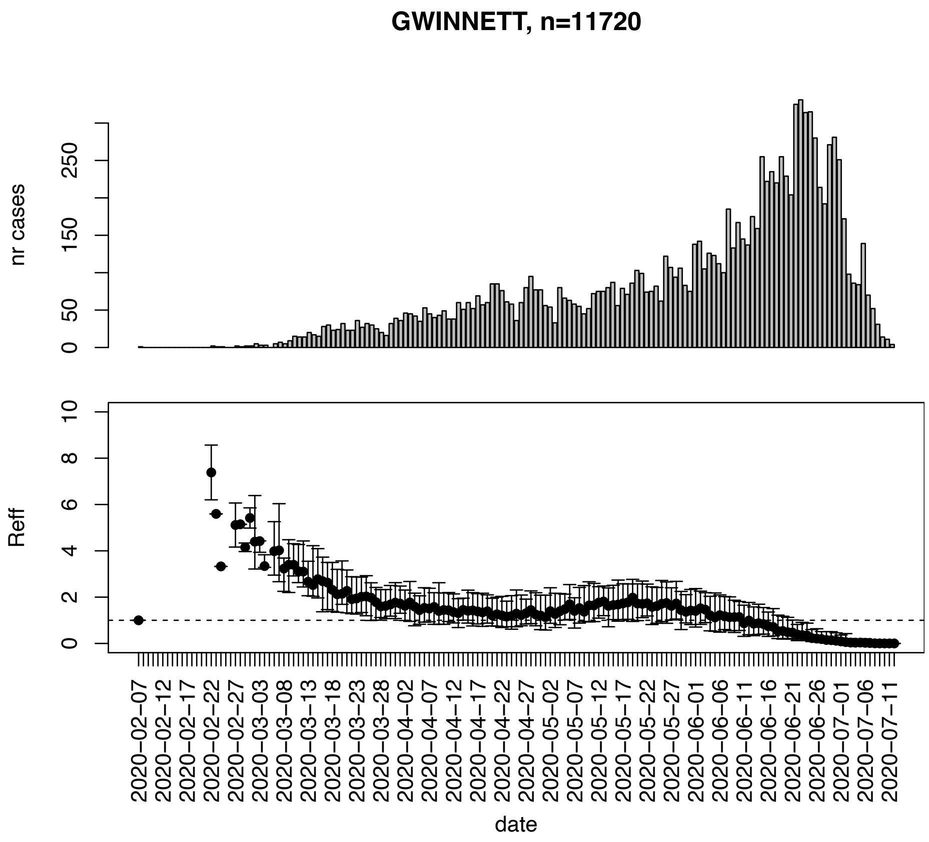

Appendix Figure 77. Epidemic curves and reproduction number estimates until July 13th in Gwinnett county.

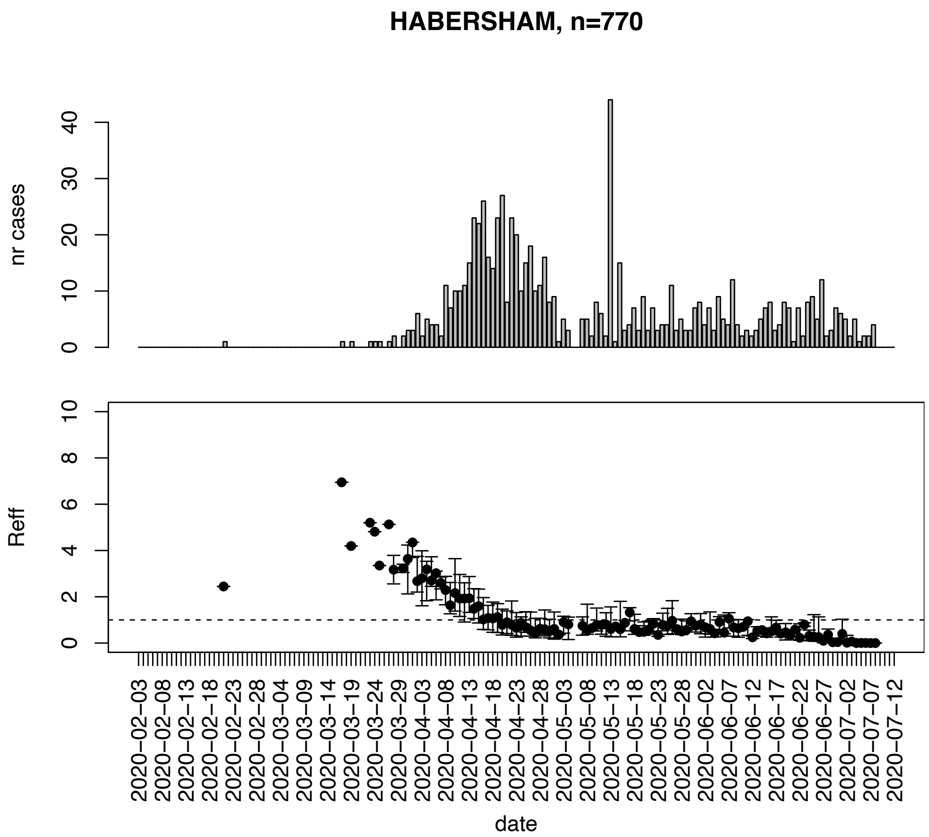

Appendix Figure 78. Epidemic curves and reproduction number estimates until July 13th in Habersham county.

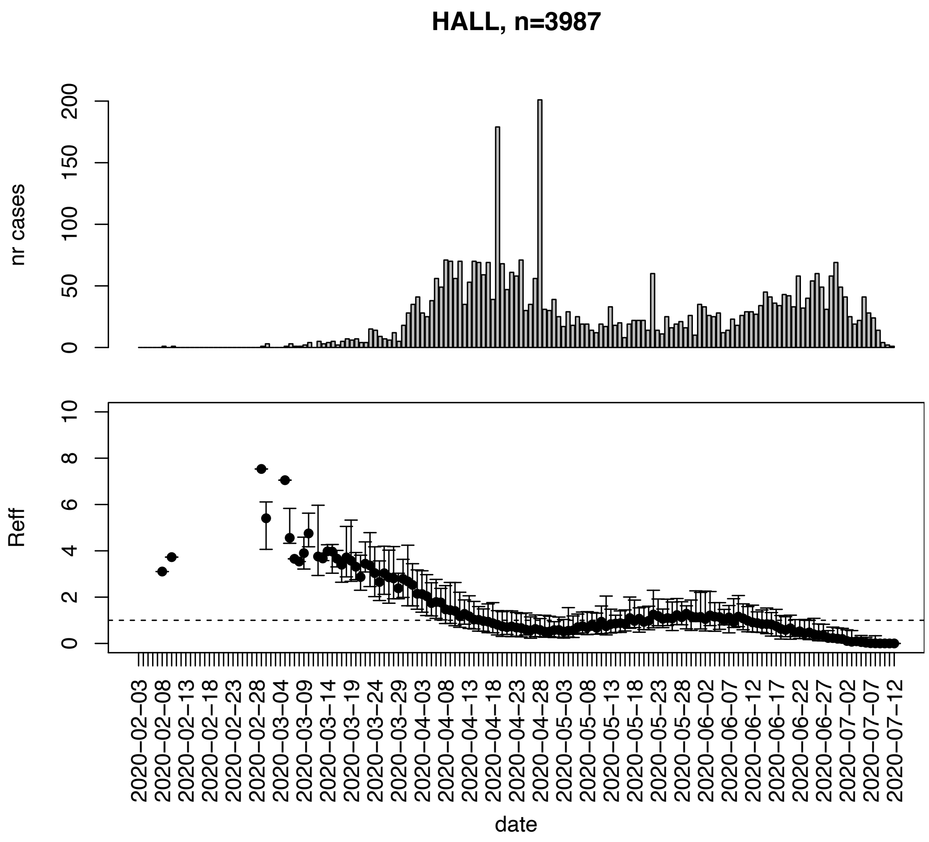

Appendix Figure 79. Epidemic curves and reproduction number estimates until July 13th in Hall county.

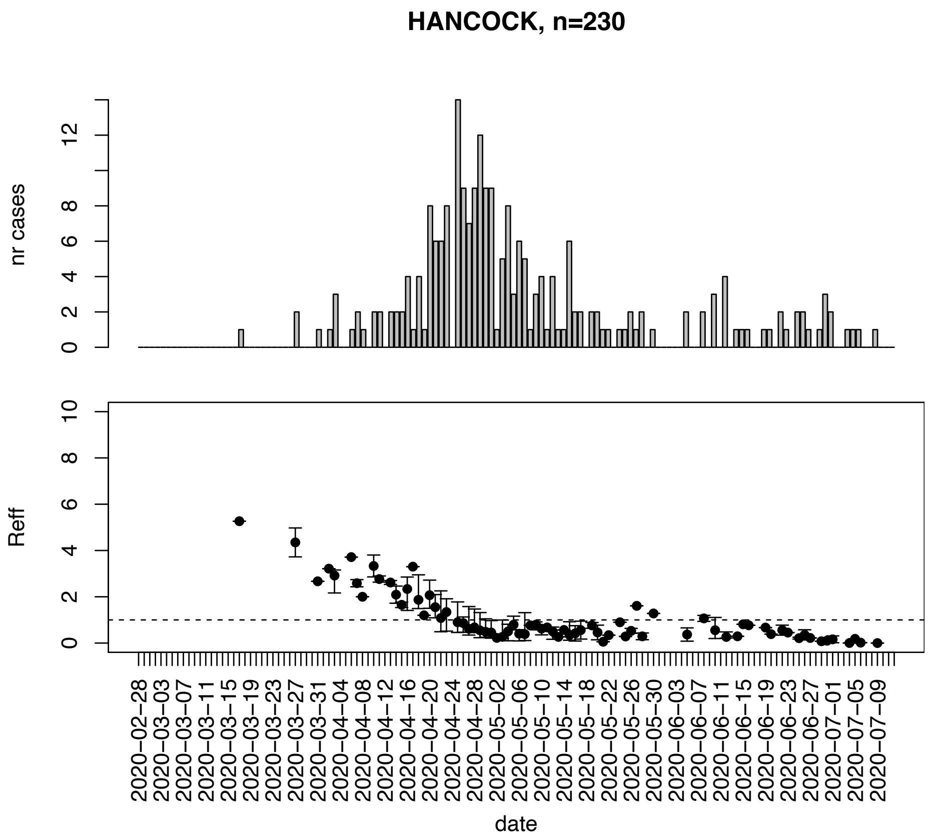

Appendix Figure 80. Epidemic curves and reproduction number estimates until July 13th in Hancock county.

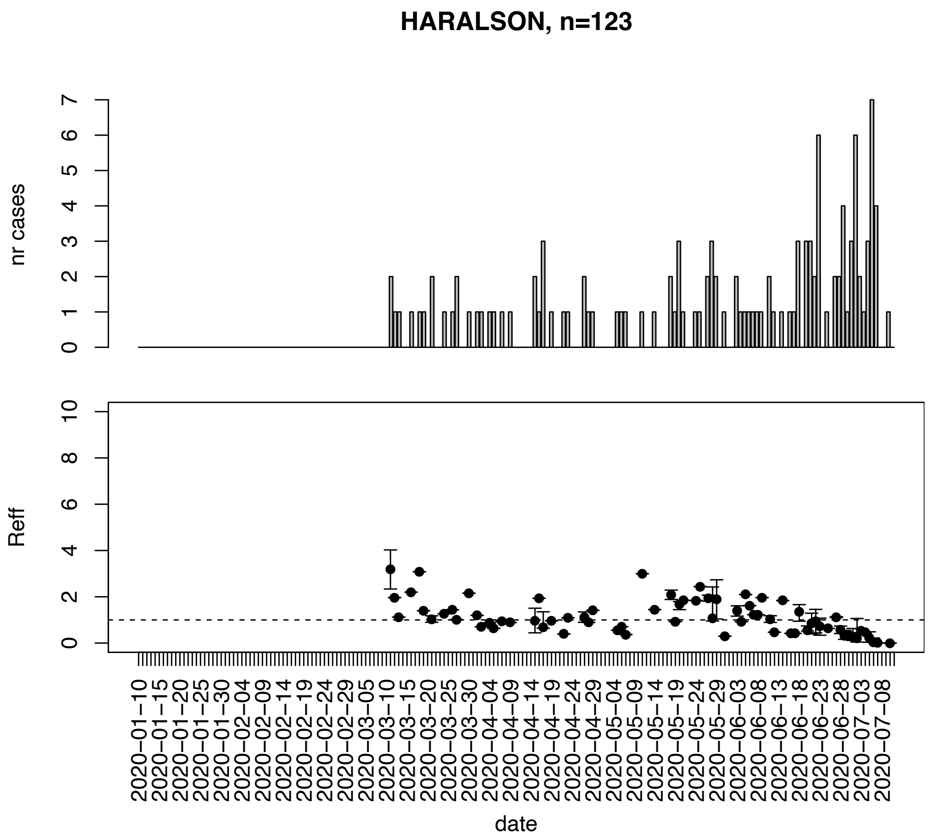

Appendix Figure 81. Epidemic curves and reproduction number estimates until July 13th in Haralson county.

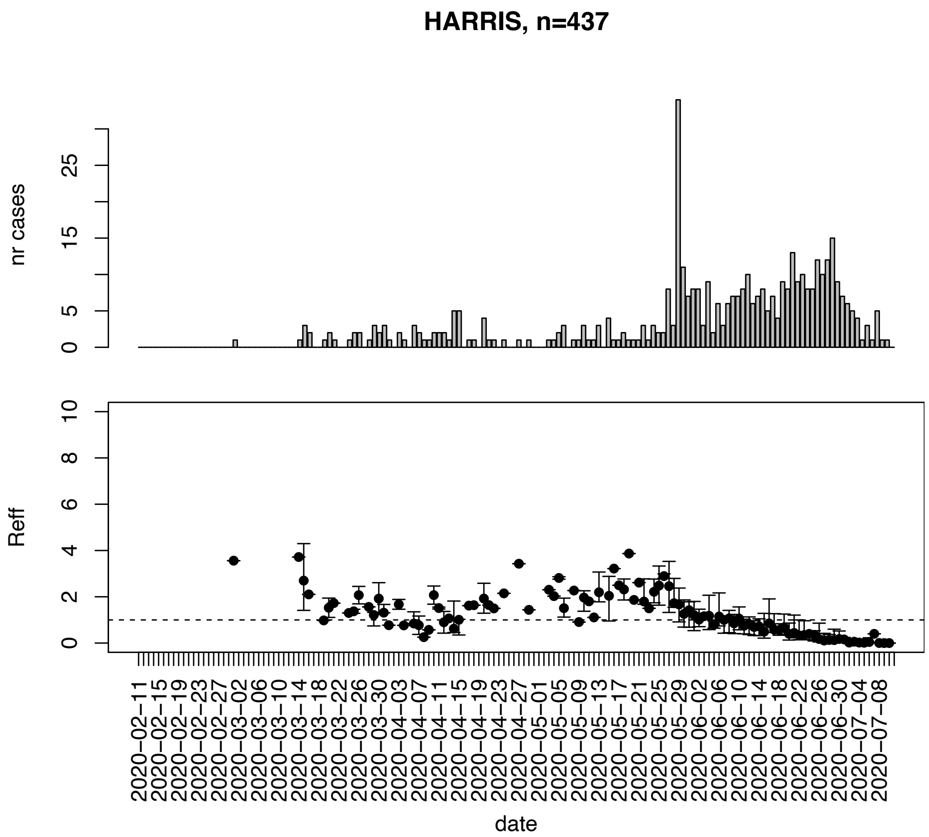

Appendix Figure 82. Epidemic curves and reproduction number estimates until July 13th in Harris county.

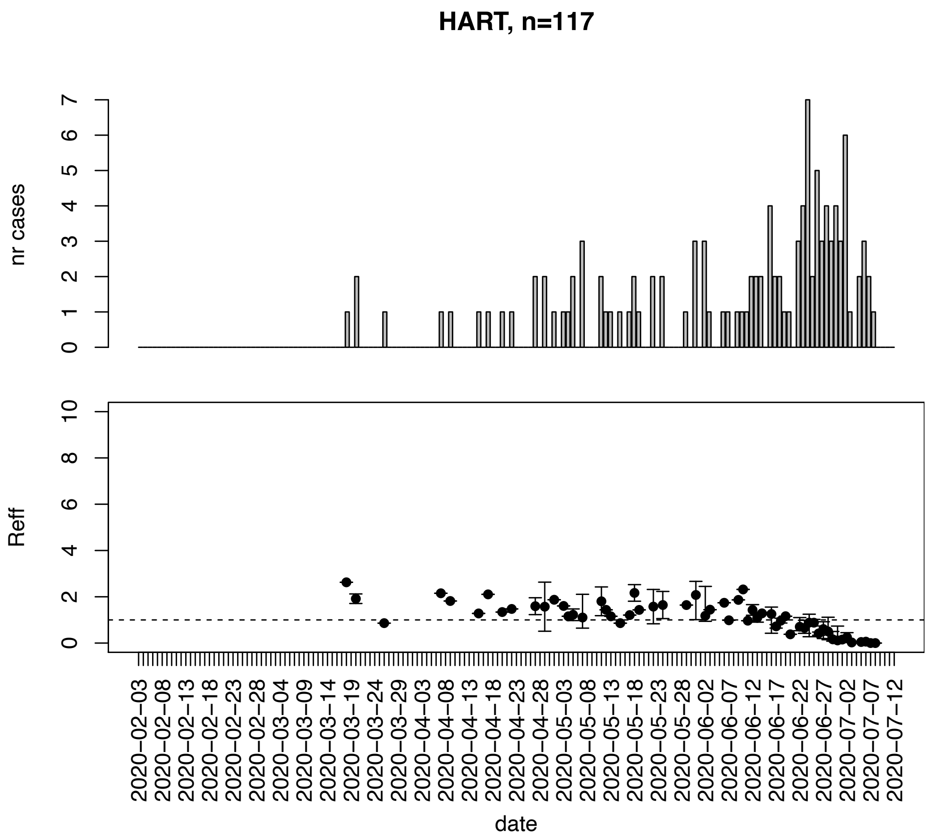

Appendix Figure 83. Epidemic curves and reproduction number estimates until July 13th in Hart county.

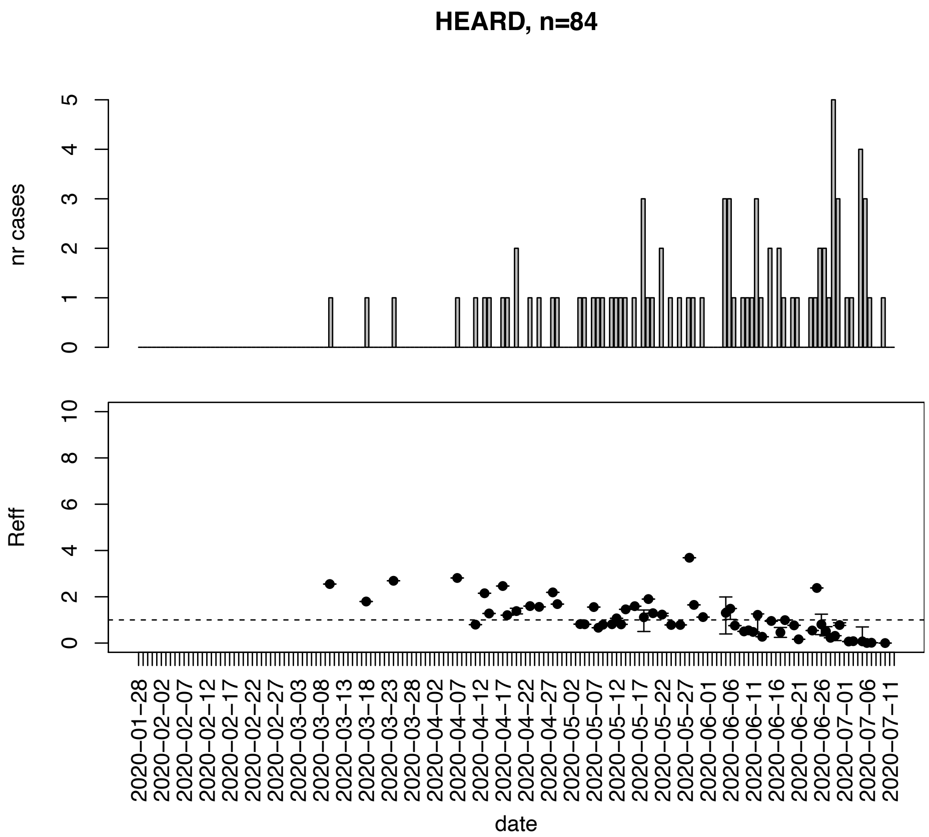

Appendix Figure 84. Epidemic curves and reproduction number estimates until July 13th in Heard county.

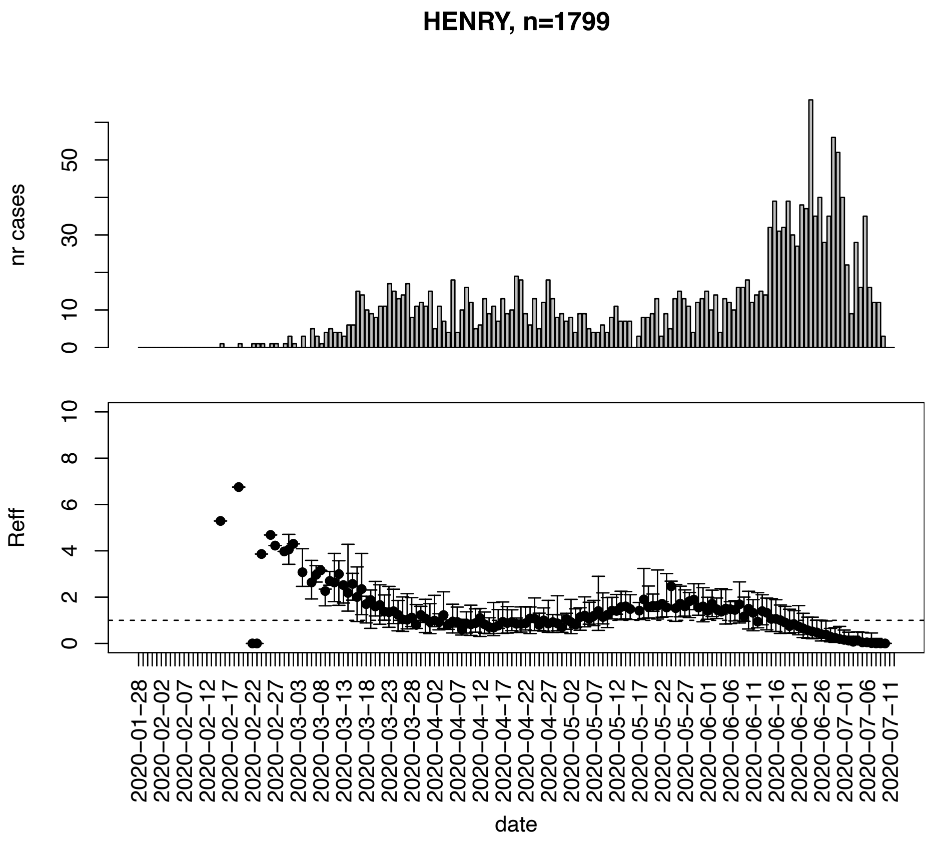

Appendix Figure 85. Epidemic curves and reproduction number estimates until July 13th in Henry county.

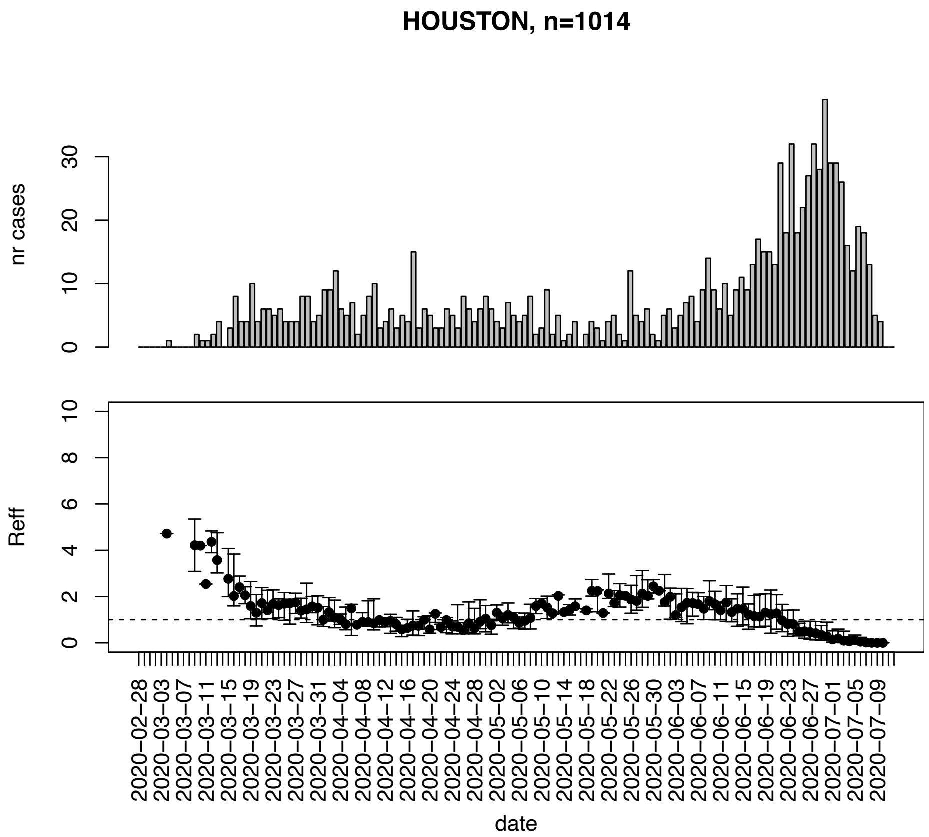

Appendix Figure 86. Epidemic curves and reproduction number estimates until July 13th in Houston county.

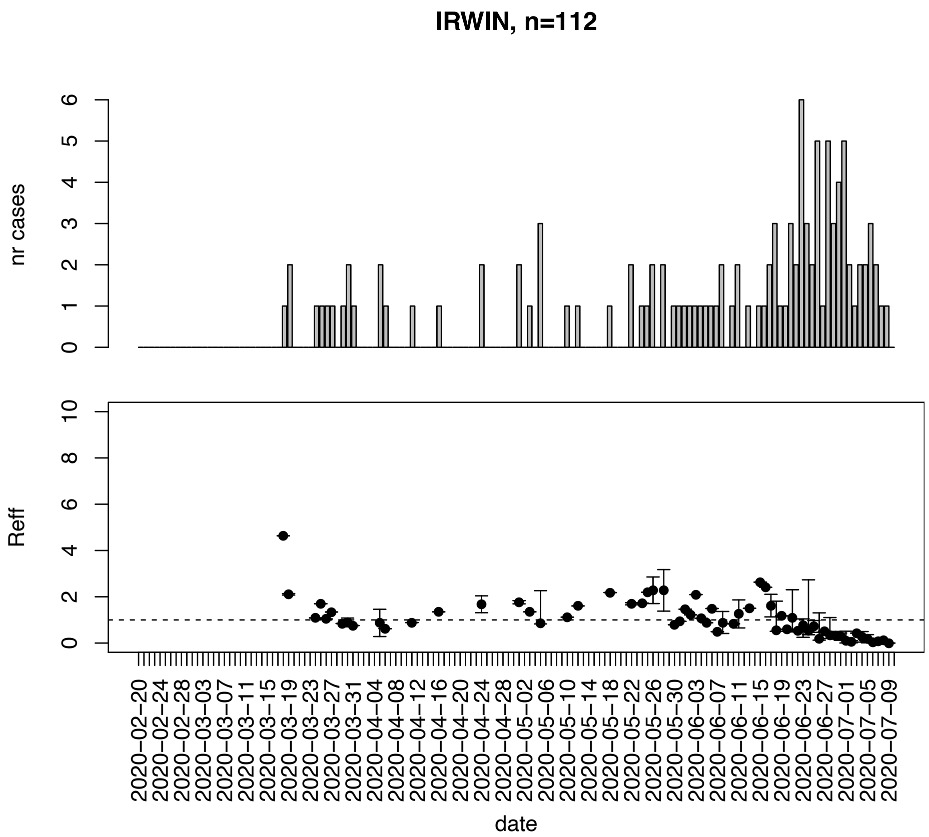

Appendix Figure 87. Epidemic curves and reproduction number estimates until July 13th in Irwin county.

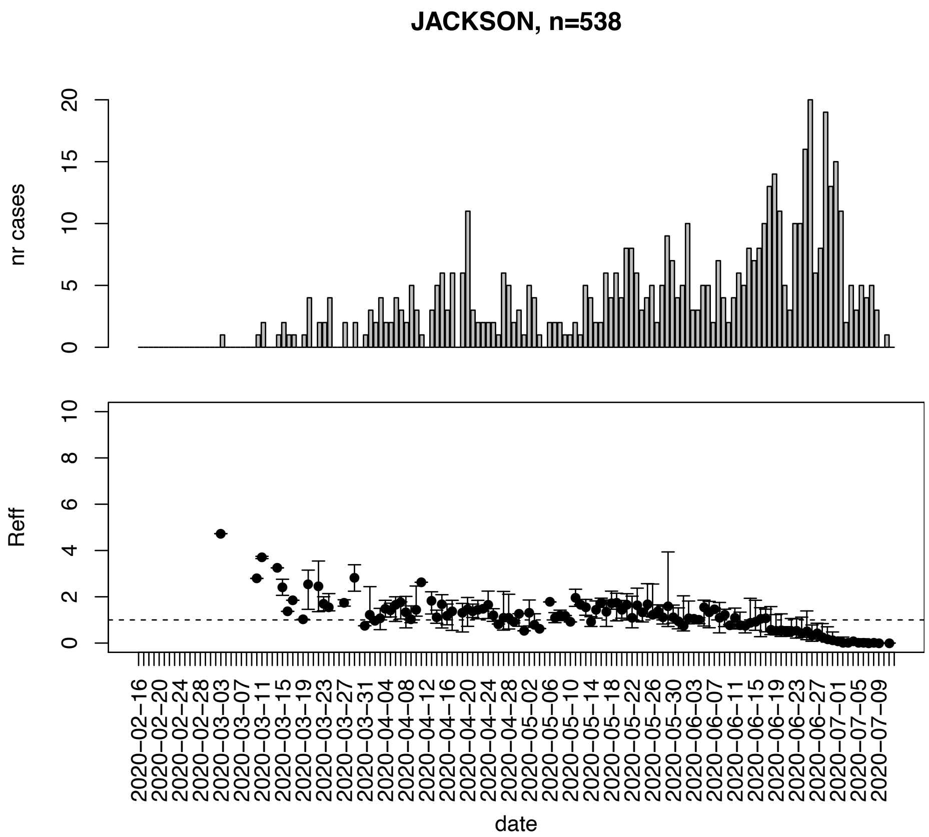

Appendix Figure 88. Epidemic curves and reproduction number estimates until July 13th in Jackson county.

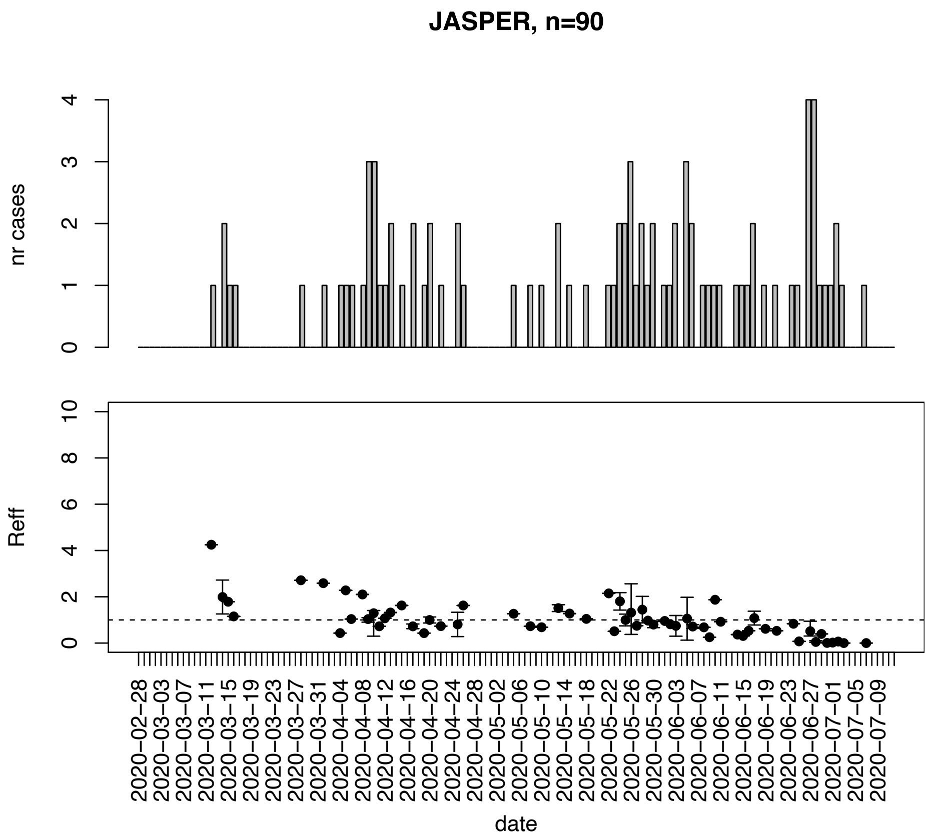

Appendix Figure 89. Epidemic curves and reproduction number estimates until July 13th in Jasper county.

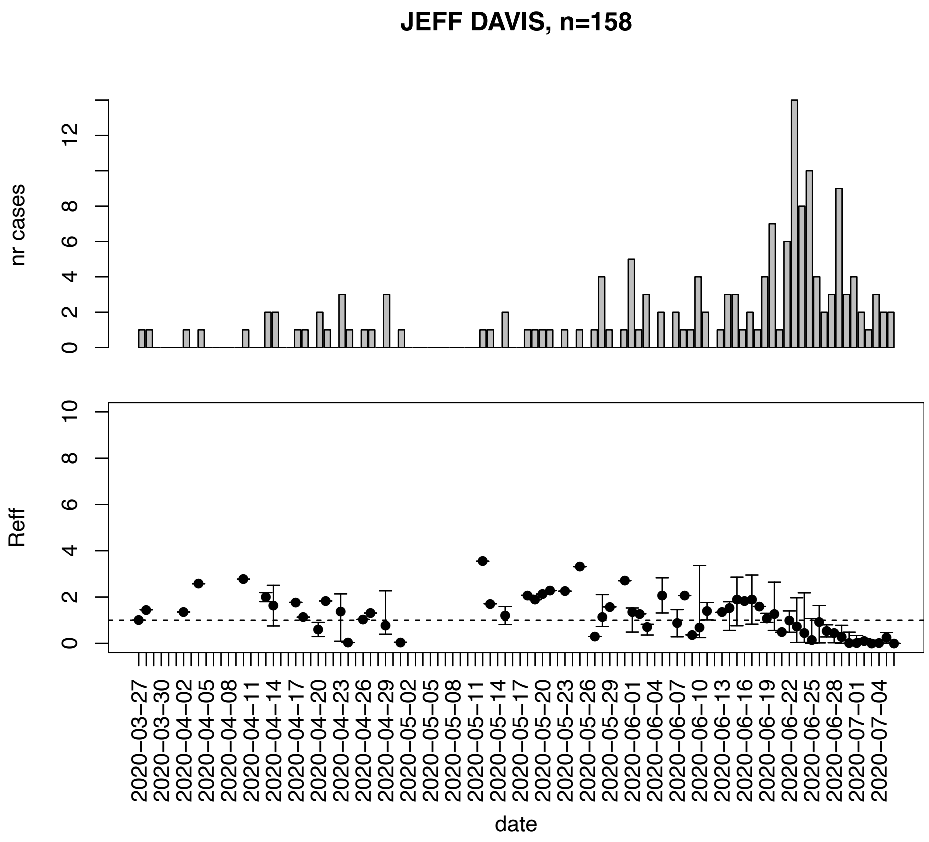

Appendix Figure 90. Epidemic curves and reproduction number estimates until July 13th in Jeff Davis county.

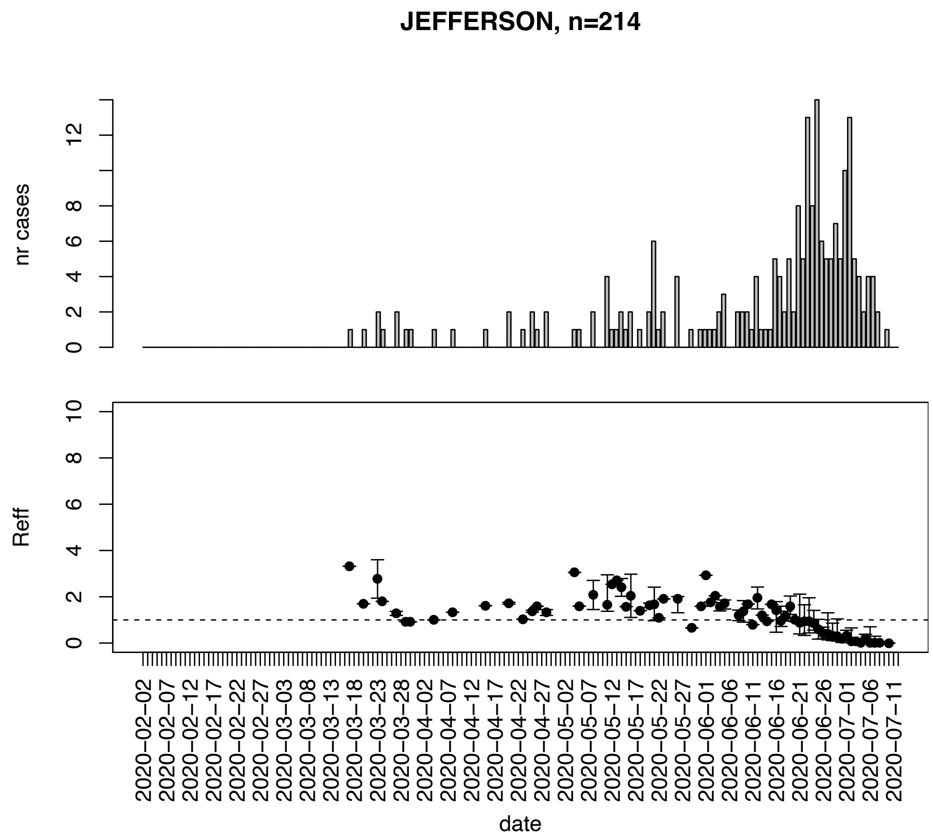

Appendix Figure 91. Epidemic curves and reproduction number estimates until July 13th in Jefferson county.

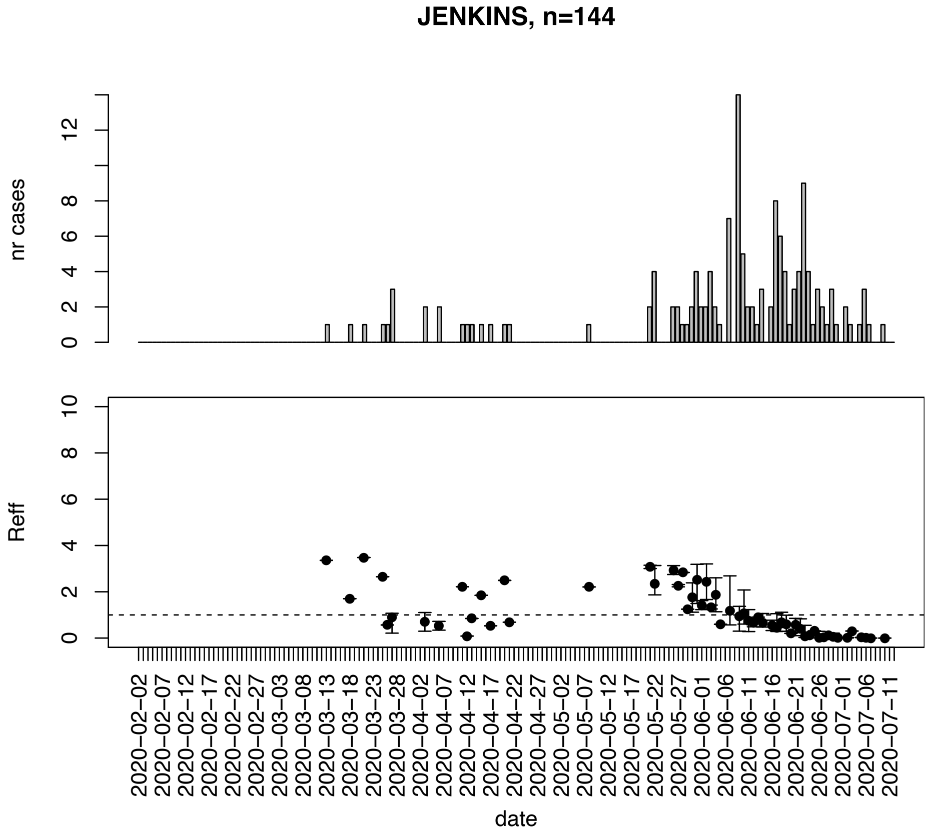

Appendix Figure 92. Epidemic curves and reproduction number estimates until July 13th in Jenkins county.

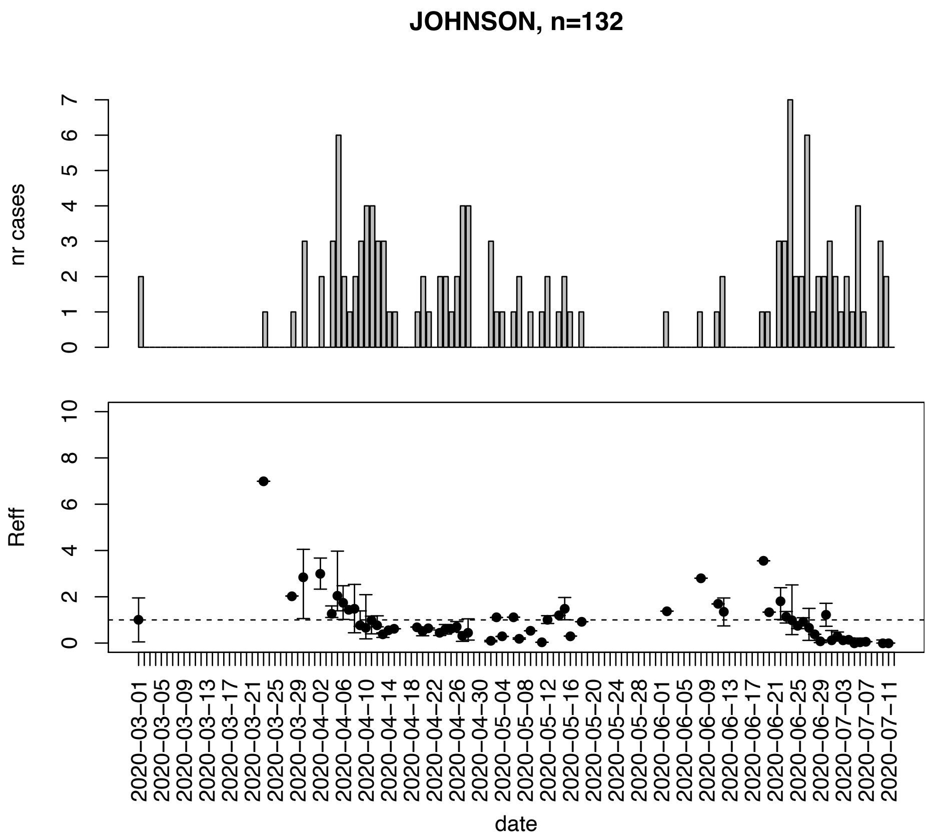

Appendix Figure 93. Epidemic curves and reproduction number estimates until July 13th in Johnson county.

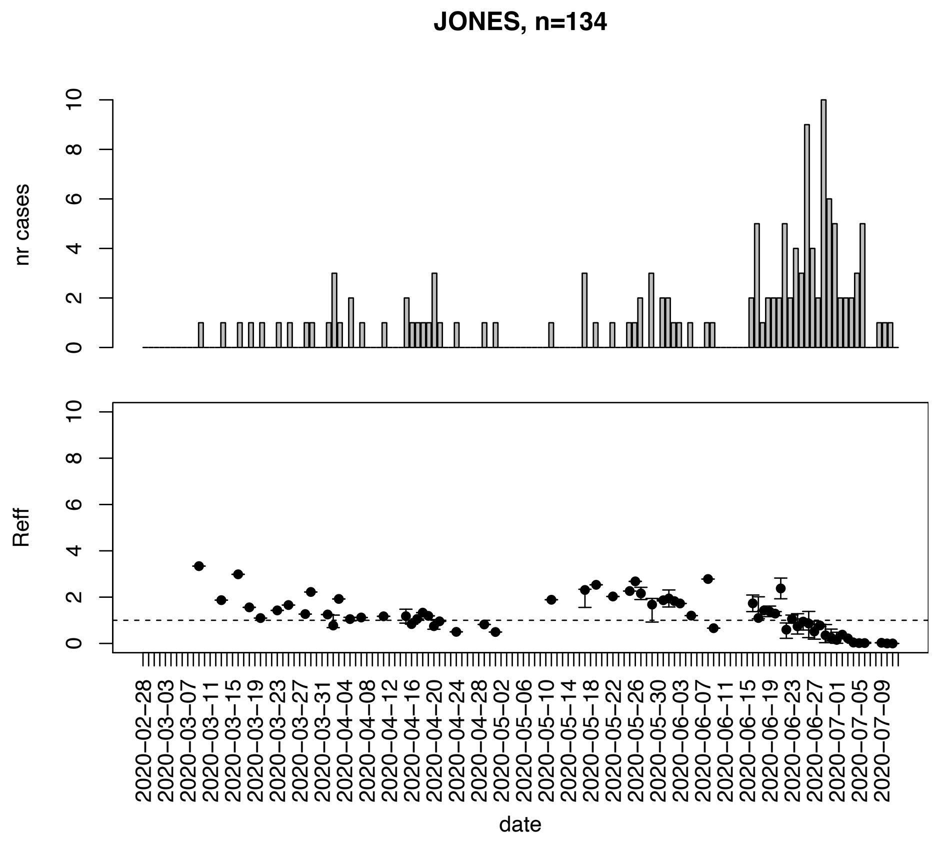

Appendix Figure 94. Epidemic curves and reproduction number estimates until July 13th in Jones county.

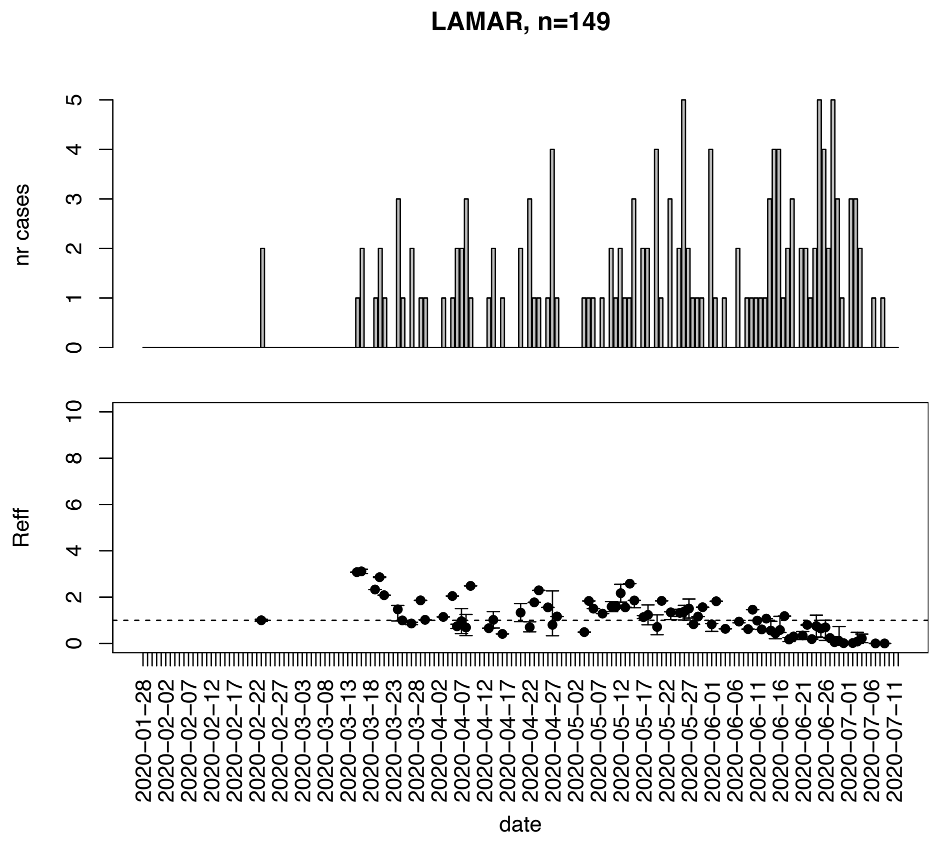

Appendix Figure 95. Epidemic curves and reproduction number estimates until July 13th in Lamar county.

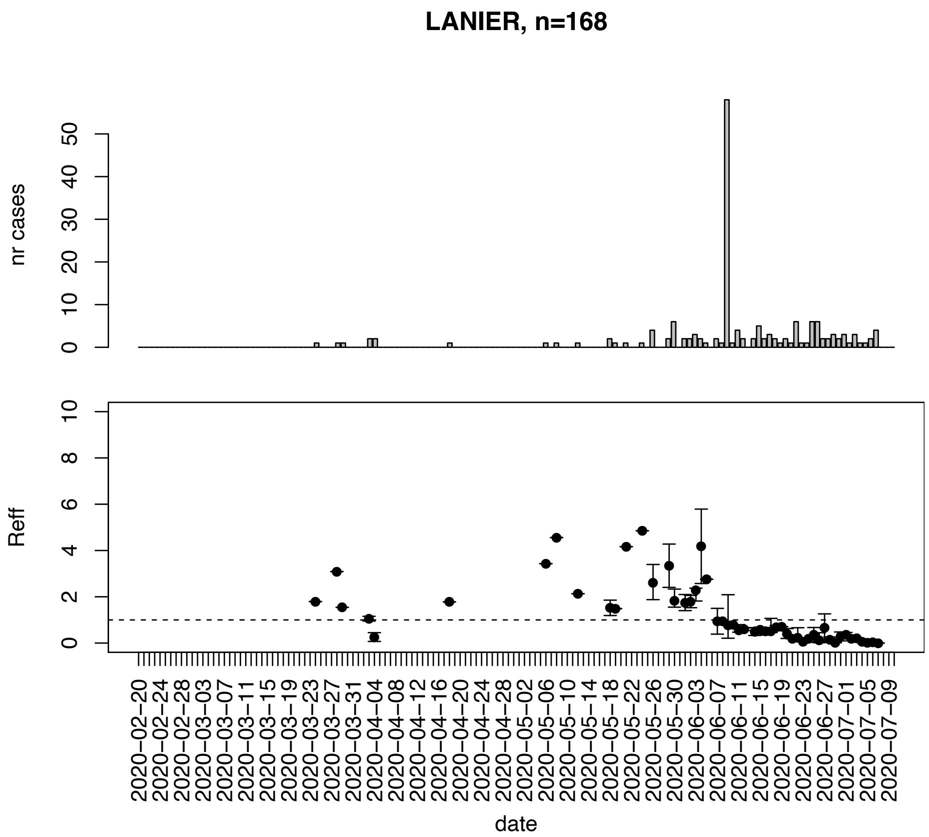

Appendix Figure 96. Epidemic curves and reproduction number estimates until July 13th in Lanier county.

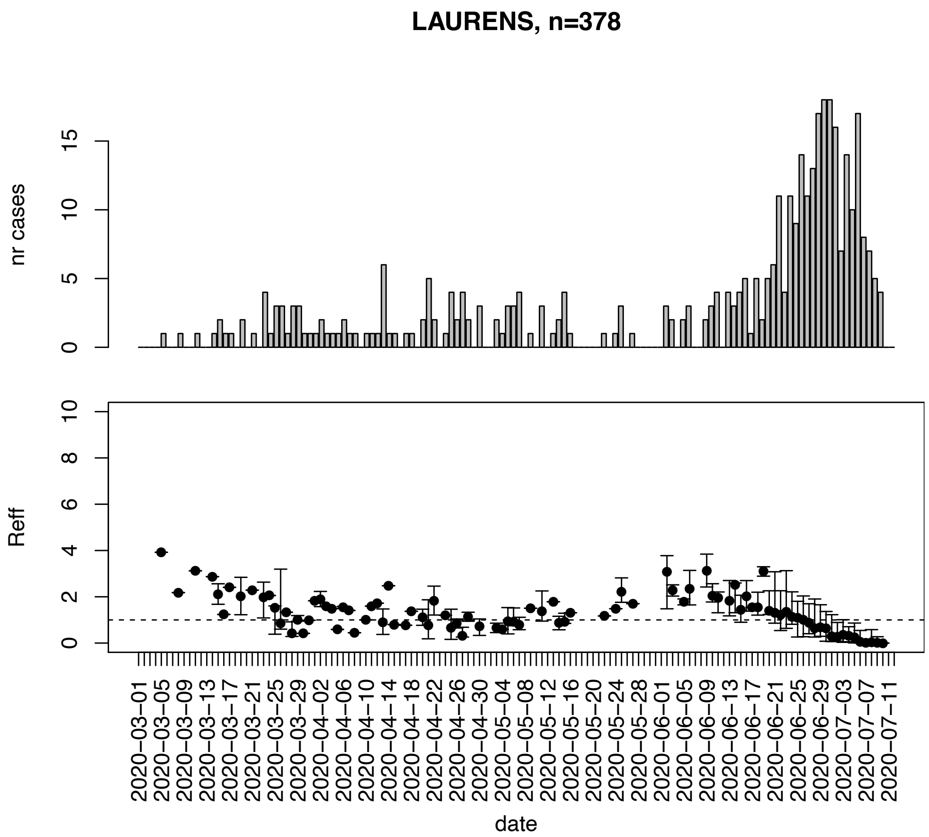

Appendix Figure 97. Epidemic curves and reproduction number estimates until July 13th in Laurens county.

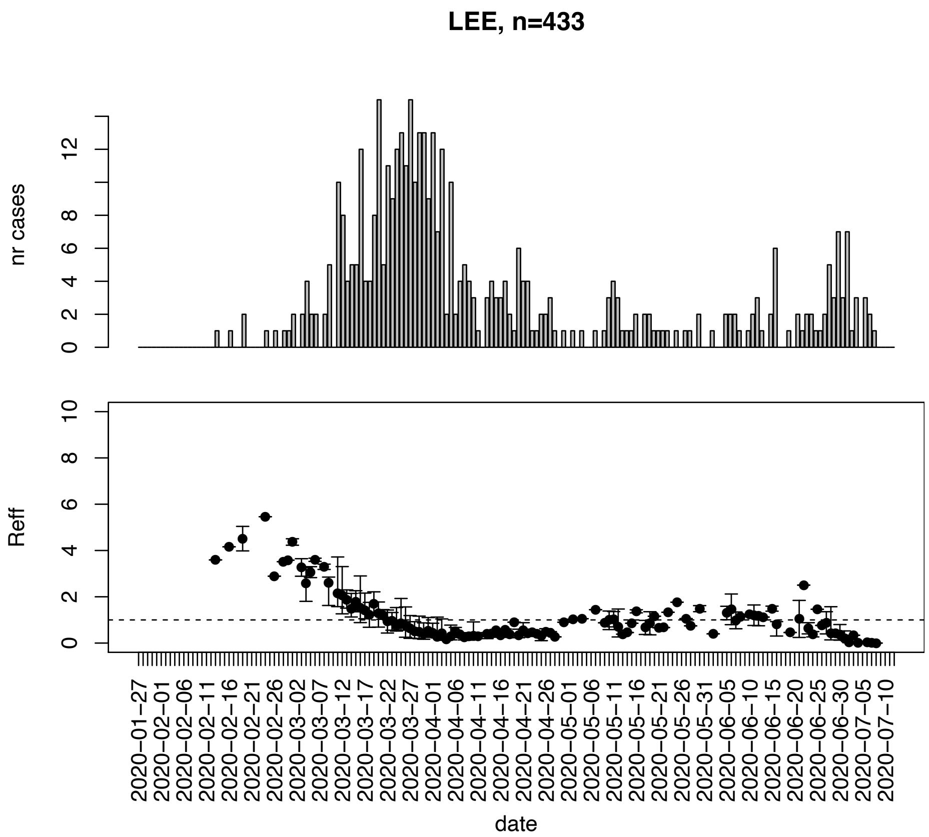

Appendix Figure 98. Epidemic curves and reproduction number estimates until July 13th in Lee county.

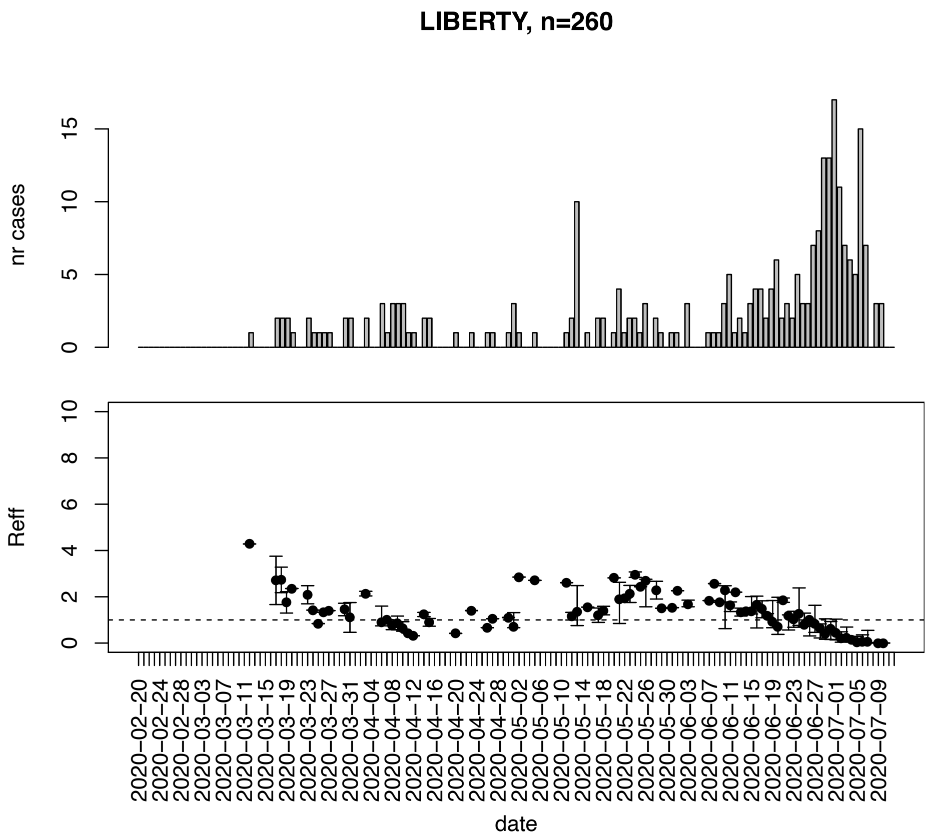

Appendix Figure 99. Epidemic curves and reproduction number estimates until July 13th in Liberty county.

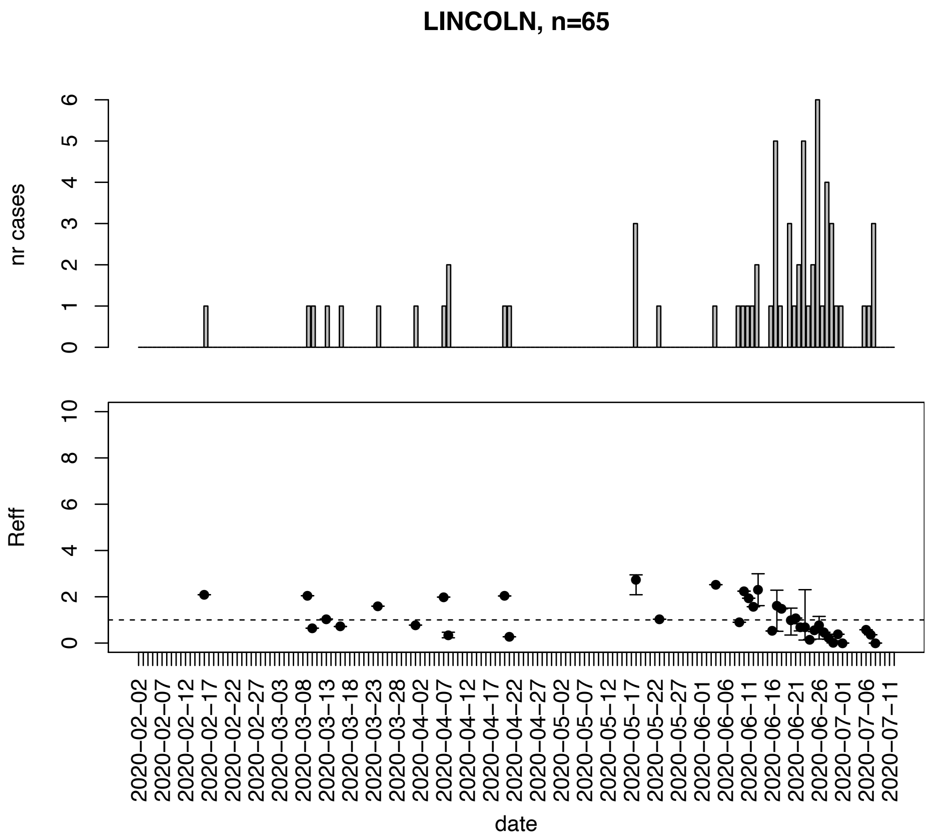

Appendix Figure 100. Epidemic curves and reproduction number estimates until July 13th in Lincoln county.

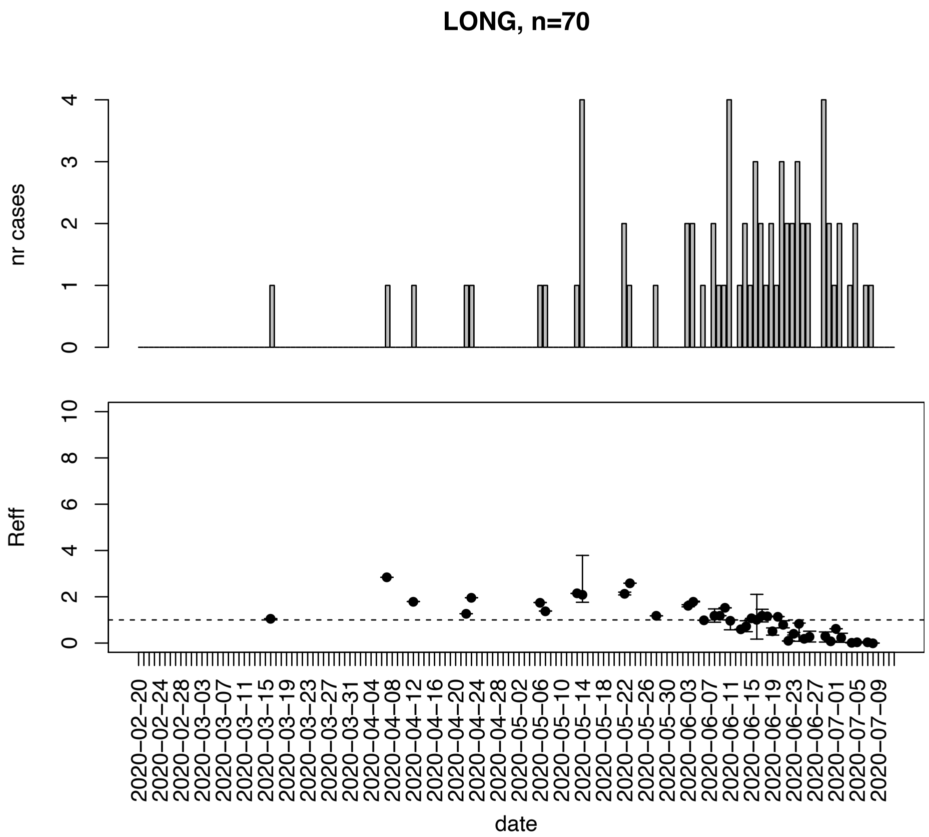

Appendix Figure 101. Epidemic curves and reproduction number estimates until July 13th in Long county.

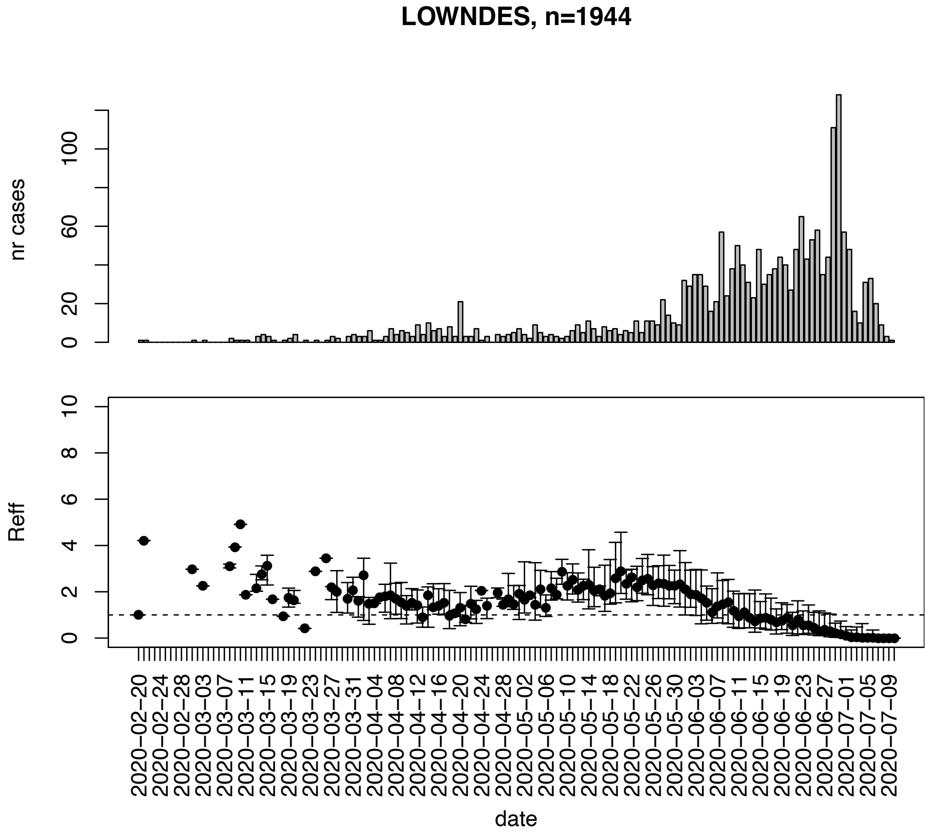

Appendix Figure 102. Epidemic curves and reproduction number estimates until July 13th in Lowndes county.

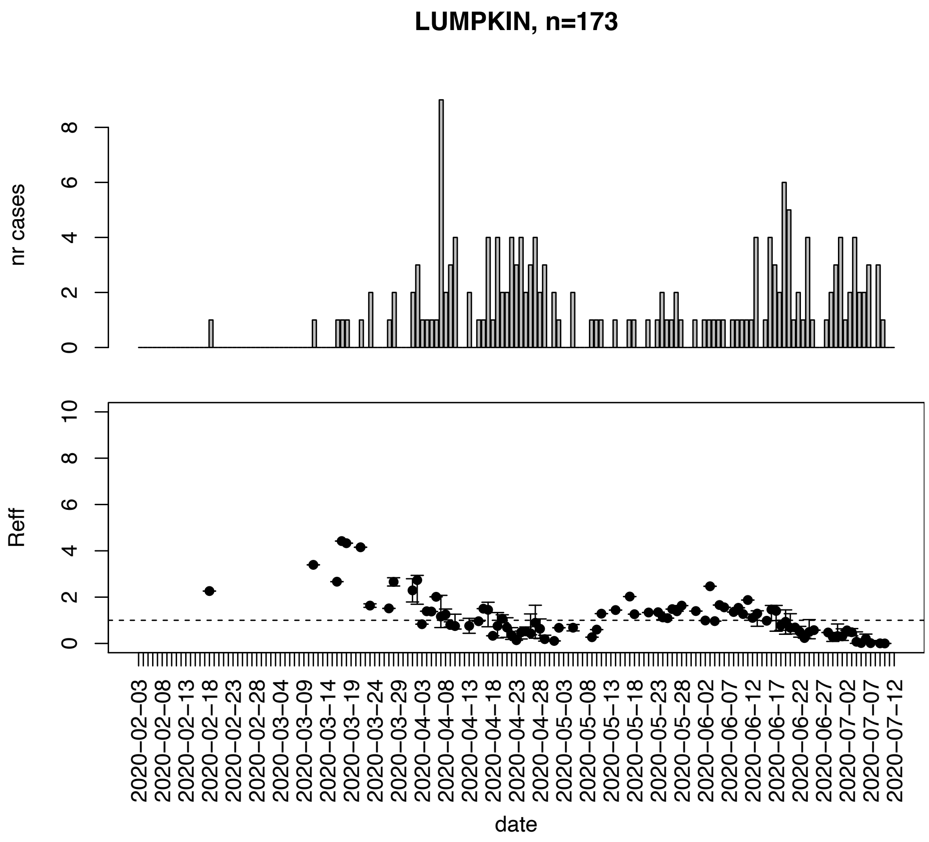

Appendix Figure 103. Epidemic curves and reproduction number estimates until July 13th in Lumpkin county.

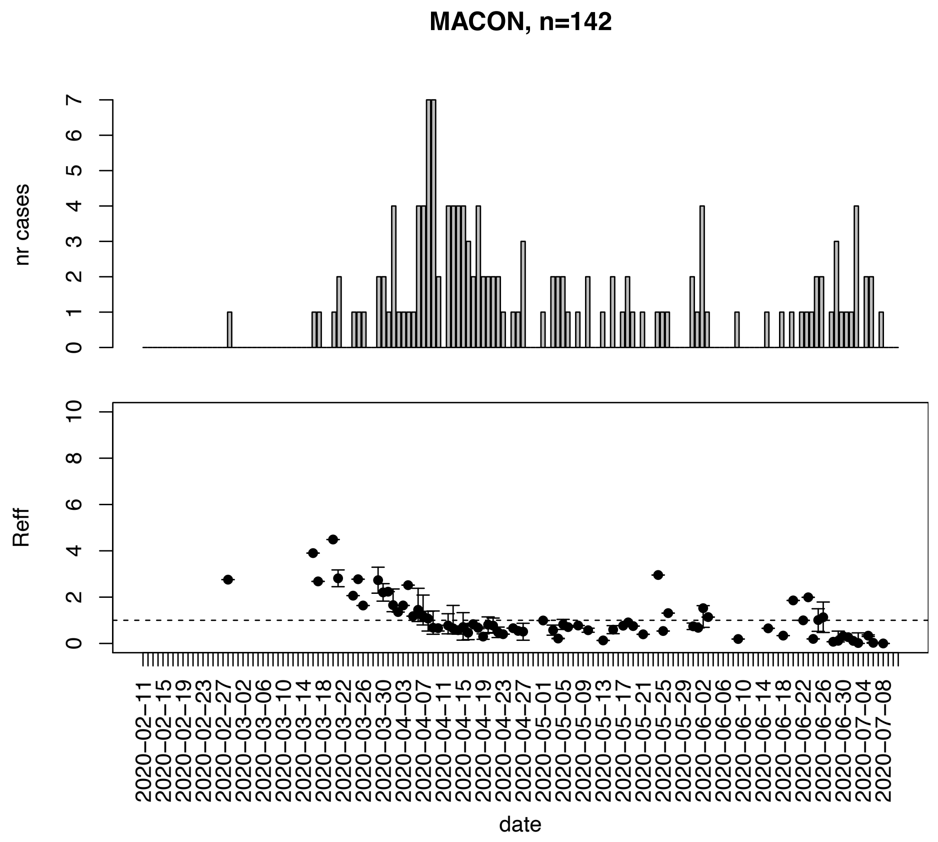

Appendix Figure 104. Epidemic curves and reproduction number estimates until July 13th in Macon county.

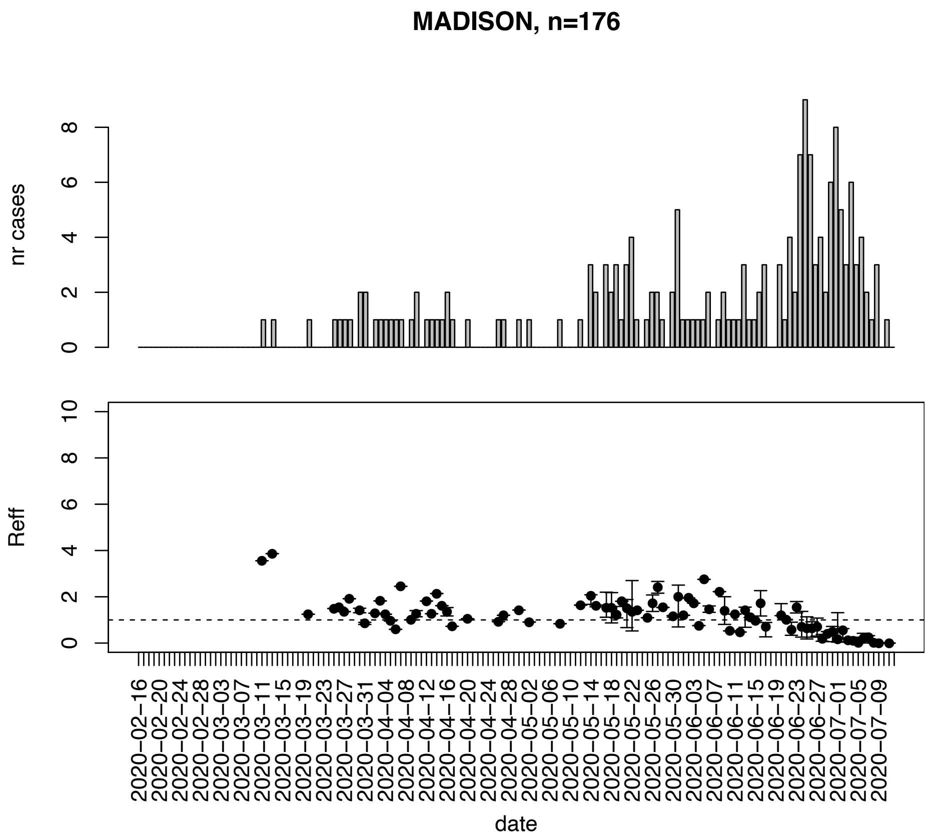

Appendix Figure 105. Epidemic curves and reproduction number estimates until July 13th in Madison county.

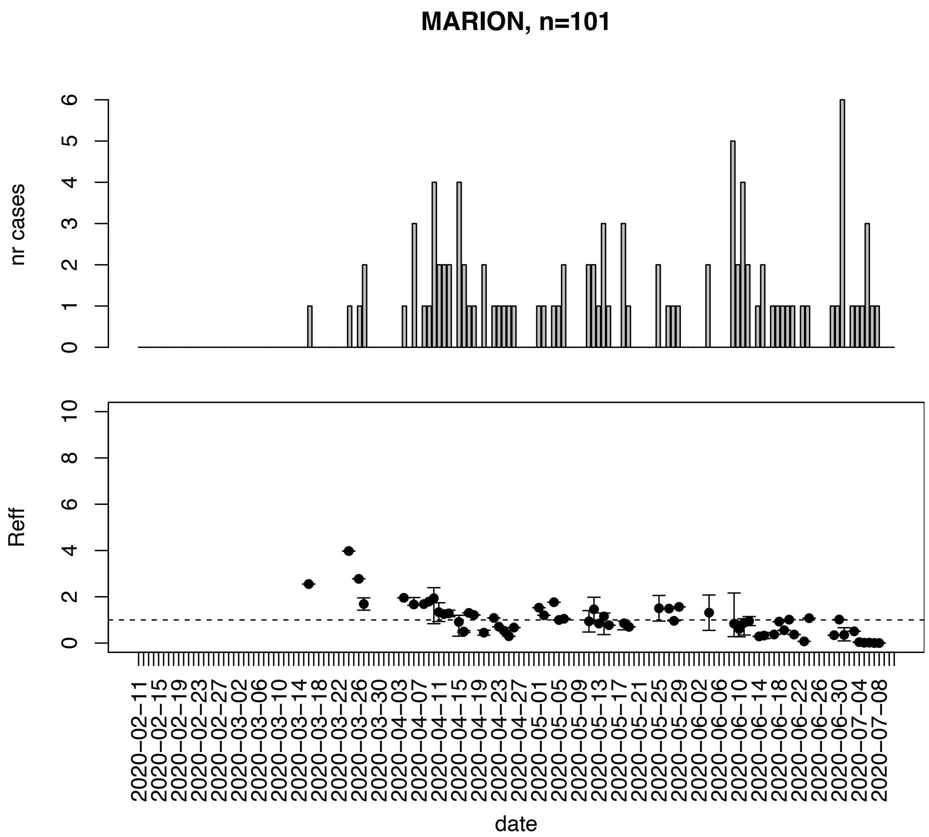

Appendix Figure 106. Epidemic curves and reproduction number estimates until July 13th in Marion county.

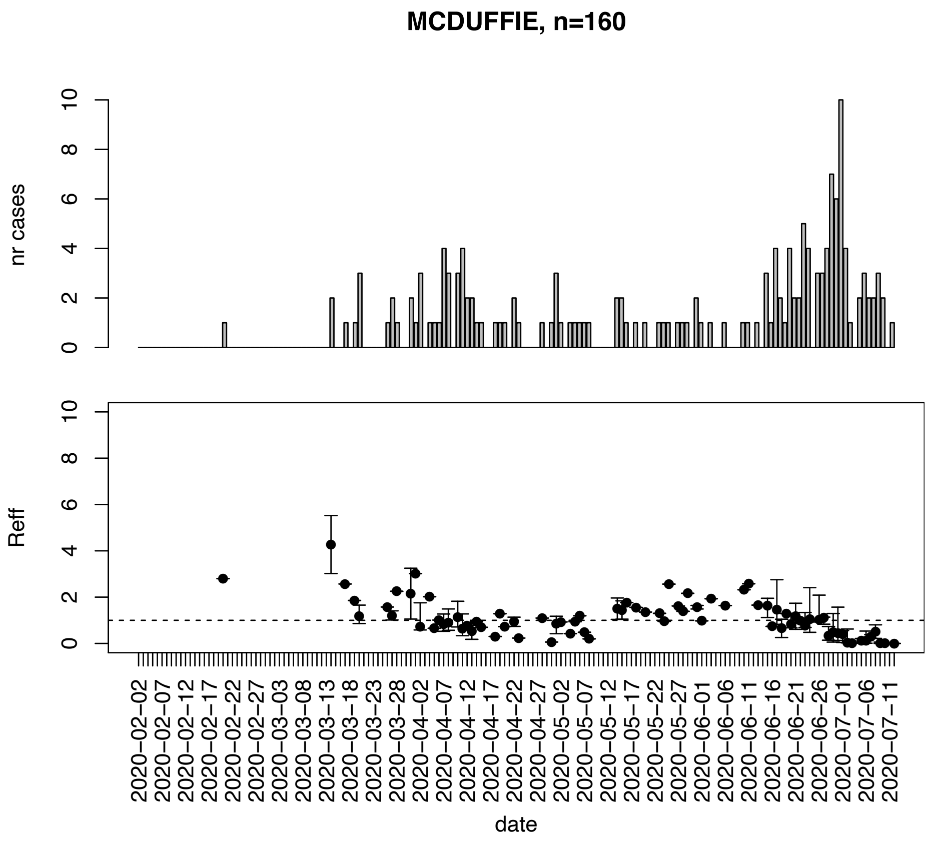

Appendix Figure 107. Epidemic curves and reproduction number estimates until July 13th in Mcduffie county.

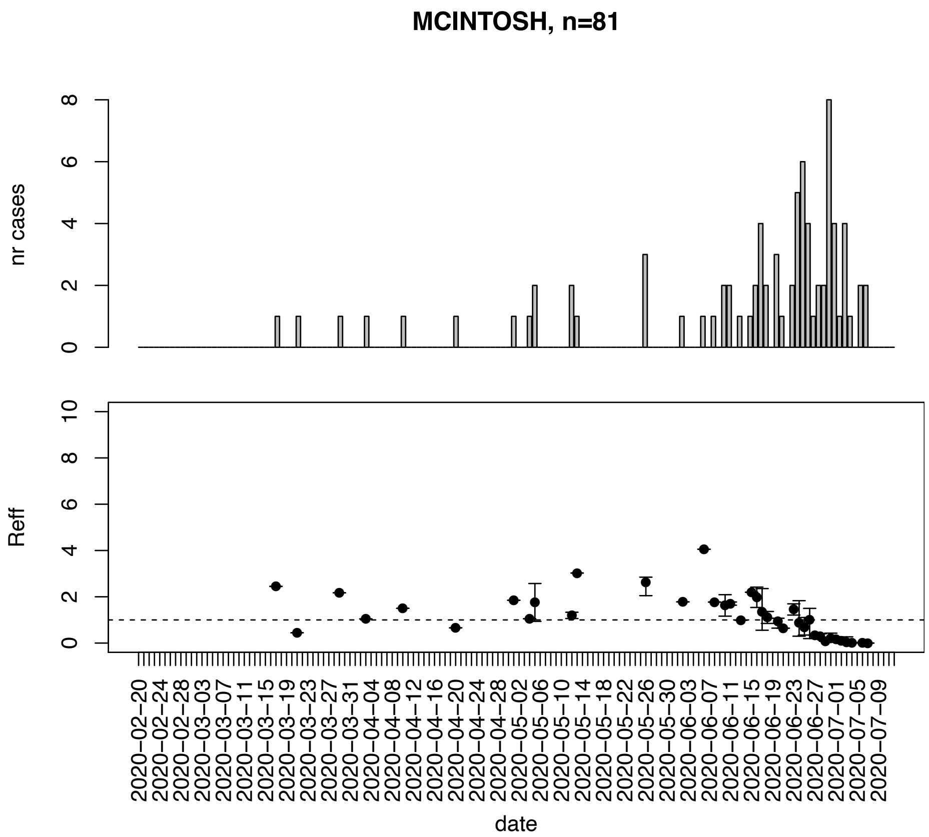

Appendix Figure 108. Epidemic curves and reproduction number estimates until July 13th in Mcintosh county.

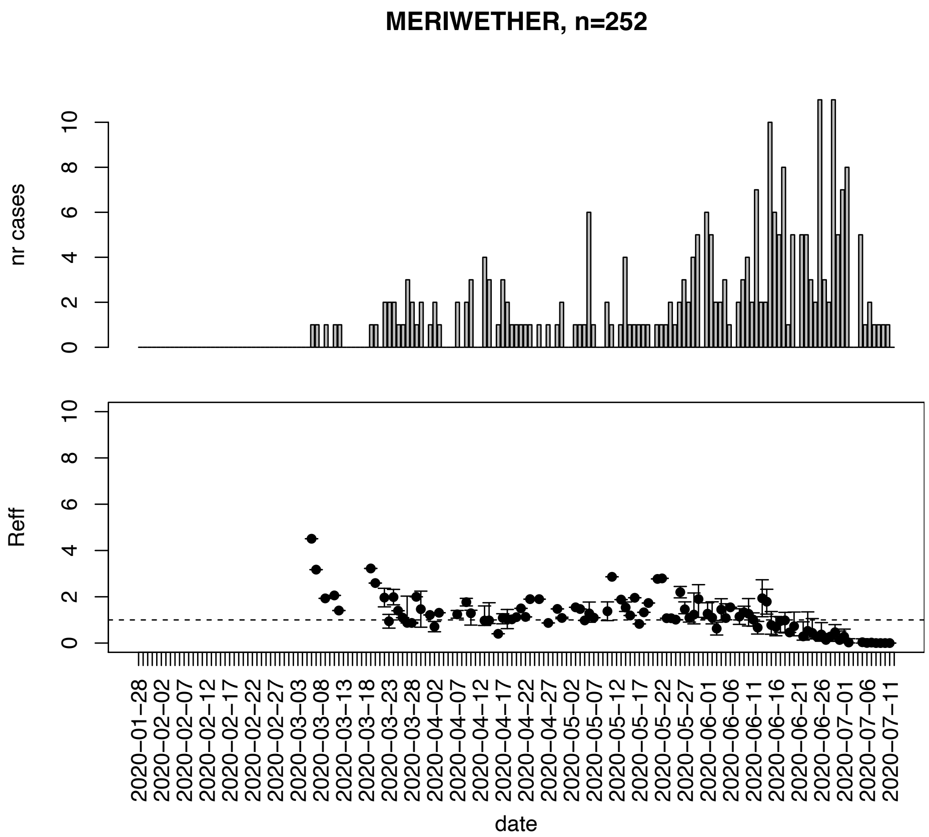

Appendix Figure 109. Epidemic curves and reproduction number estimates until July 13th in Meriwether county.

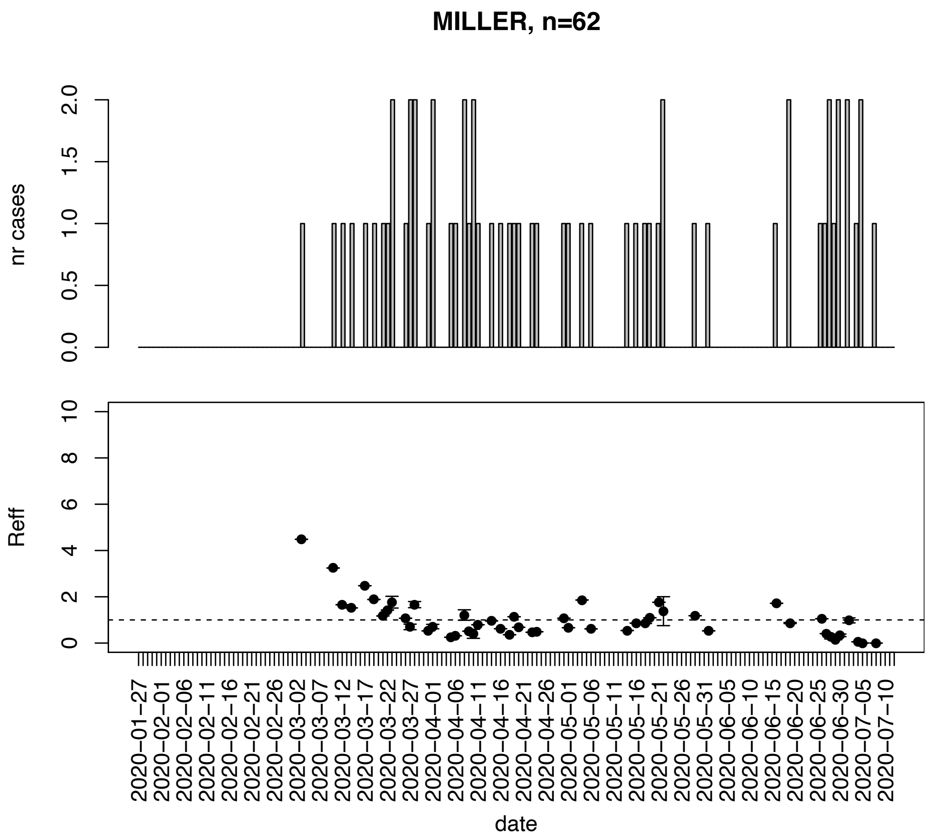

Appendix Figure 110. Epidemic curves and reproduction number estimates until July 13th in Miller county.

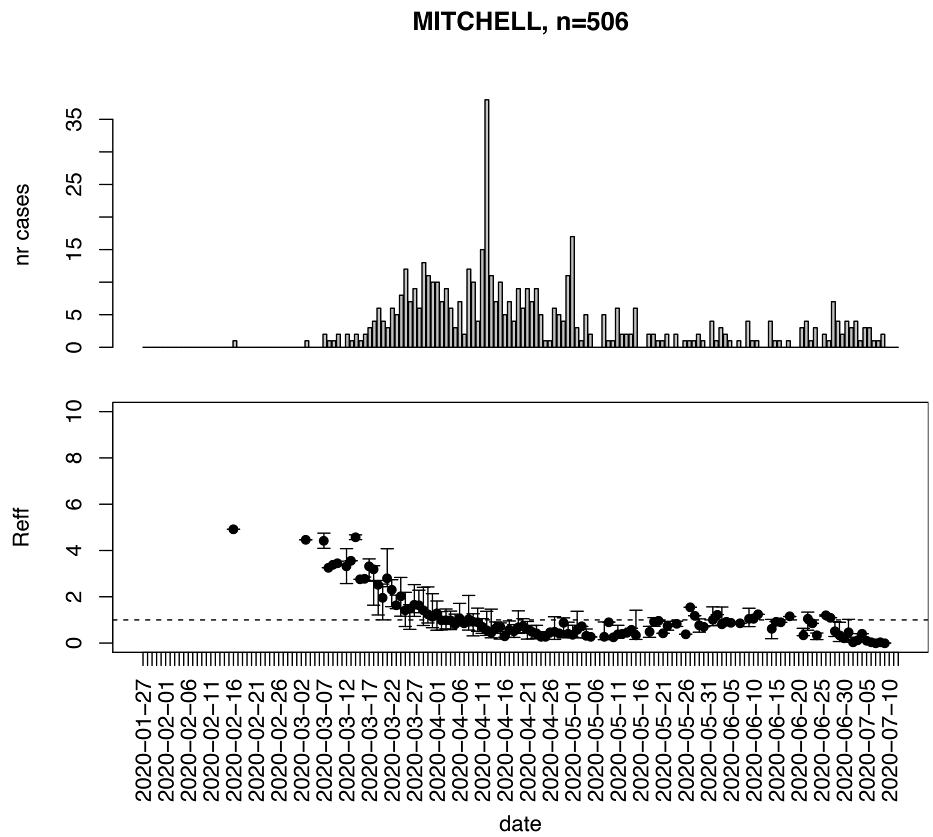

Appendix Figure 111. Epidemic curves and reproduction number estimates until July 13th in Mitchell county.

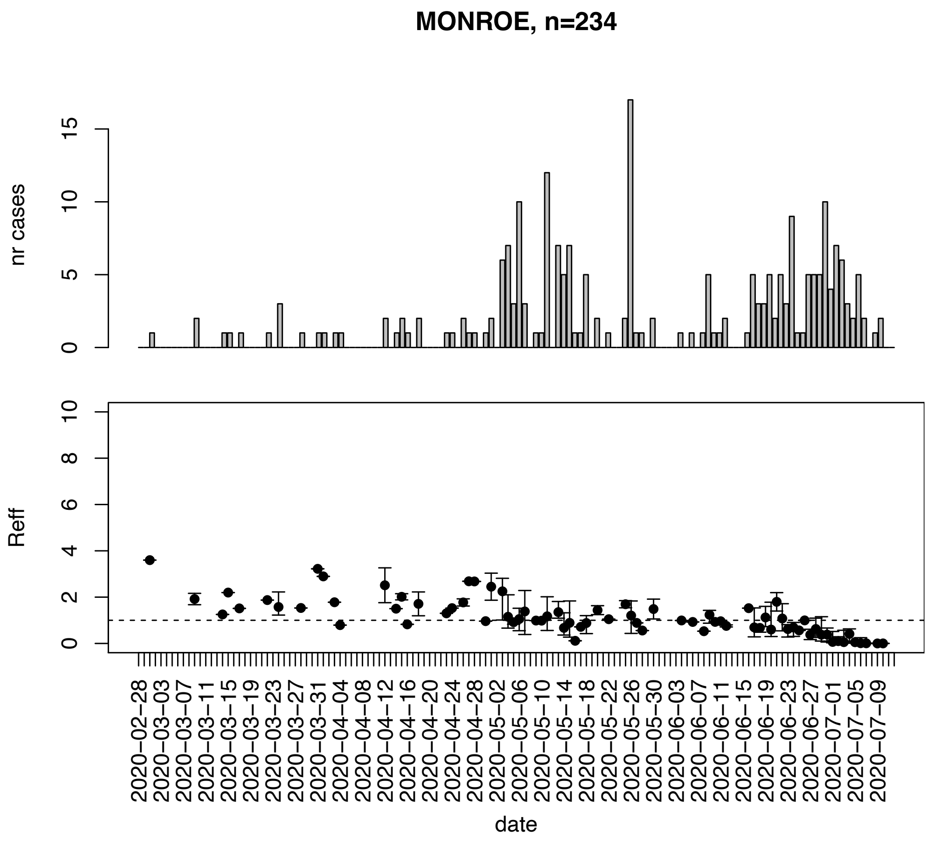

Appendix Figure 112. Epidemic curves and reproduction number estimates until July 13th in Monroe county.

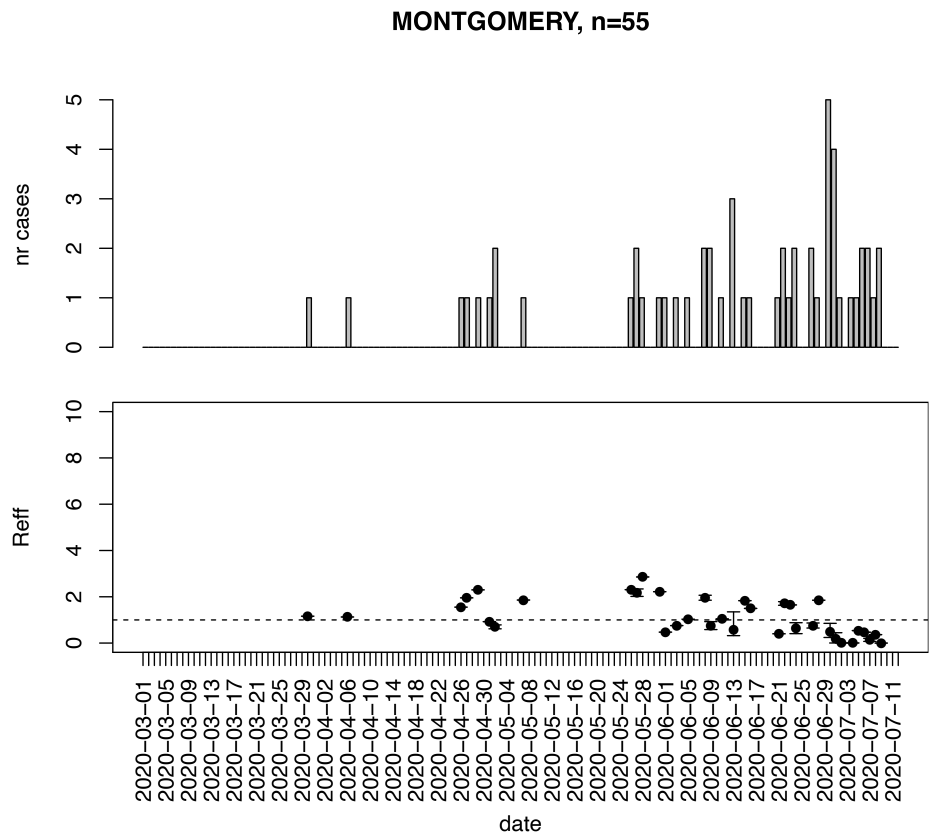

Appendix Figure 113. Epidemic curves and reproduction number estimates until July 13th in Montgomery county.

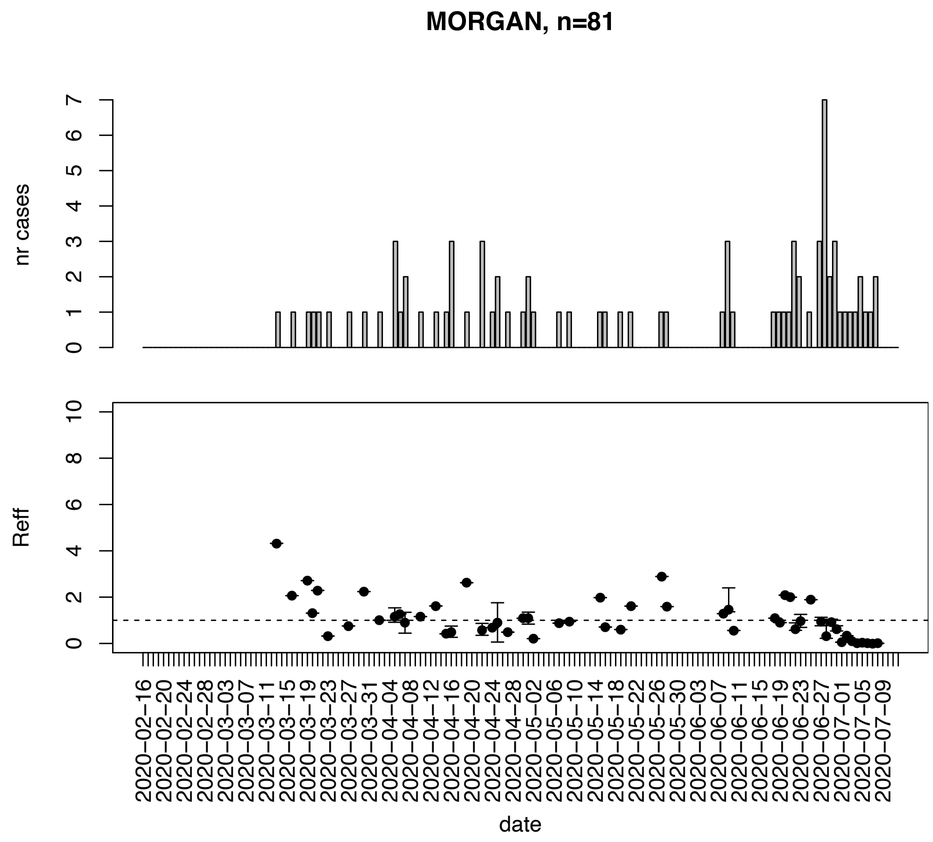

Appendix Figure 114. Epidemic curves and reproduction number estimates until July 13th in Morgan county.

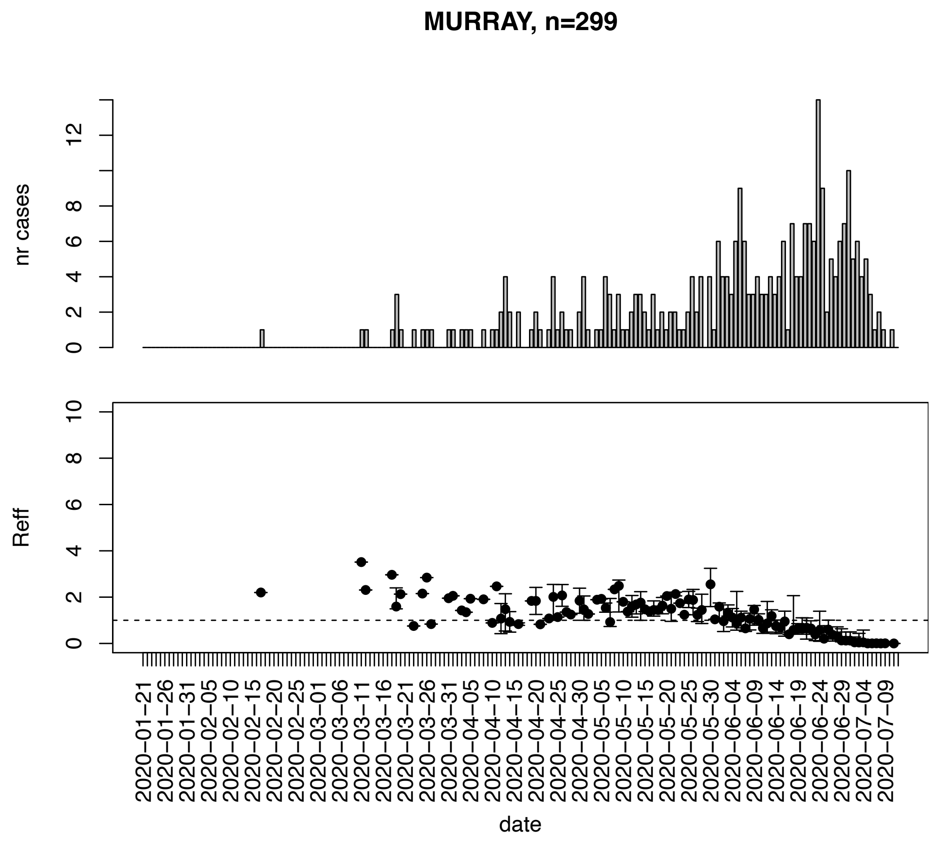

Appendix Figure 115. Epidemic curves and reproduction number estimates until July 13th in Murray county.

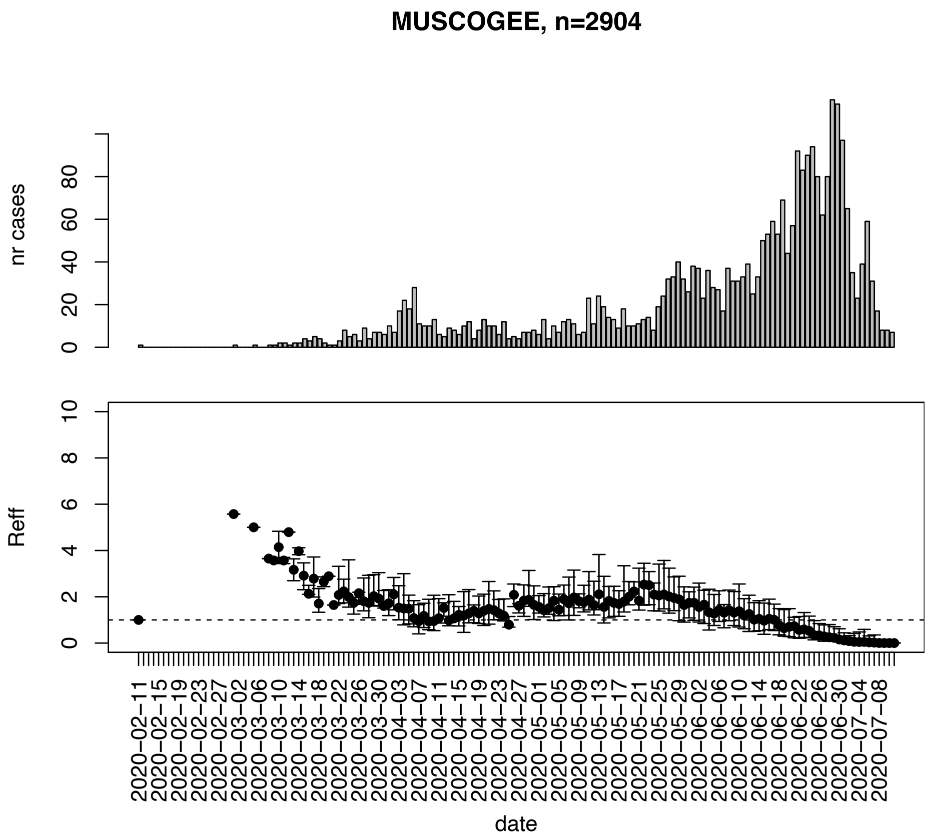

Appendix Figure 116. Epidemic curves and reproduction number estimates until July 13th in Muscogee county.

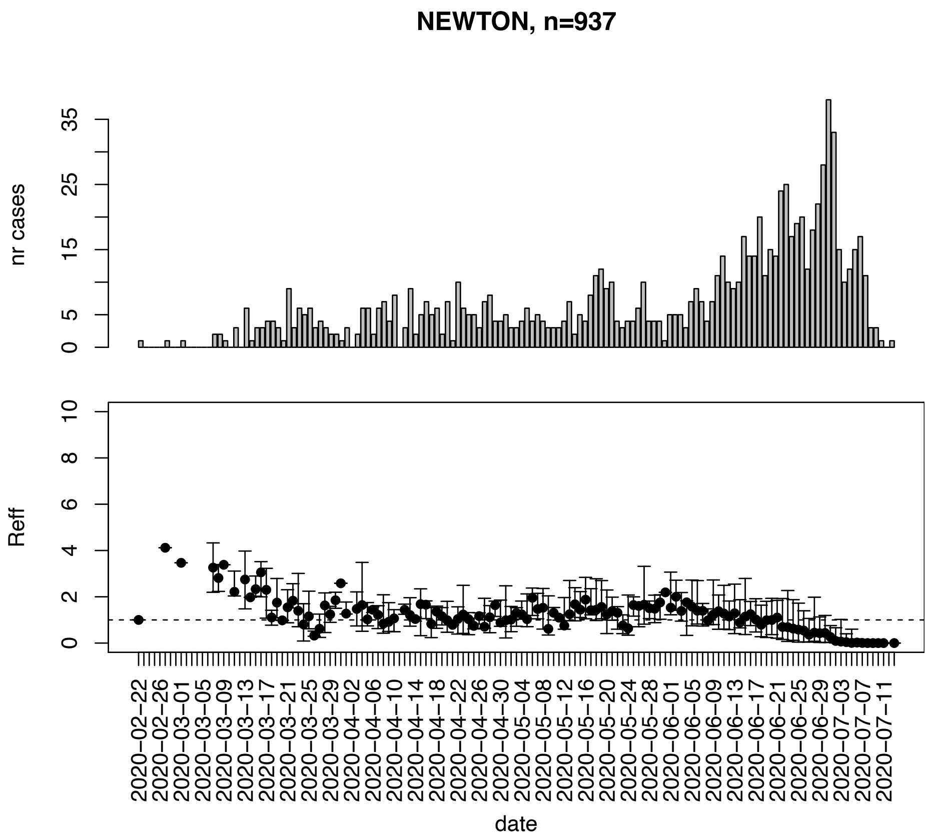

Appendix Figure 117. Epidemic curves and reproduction number estimates until July 13th in Newton county.

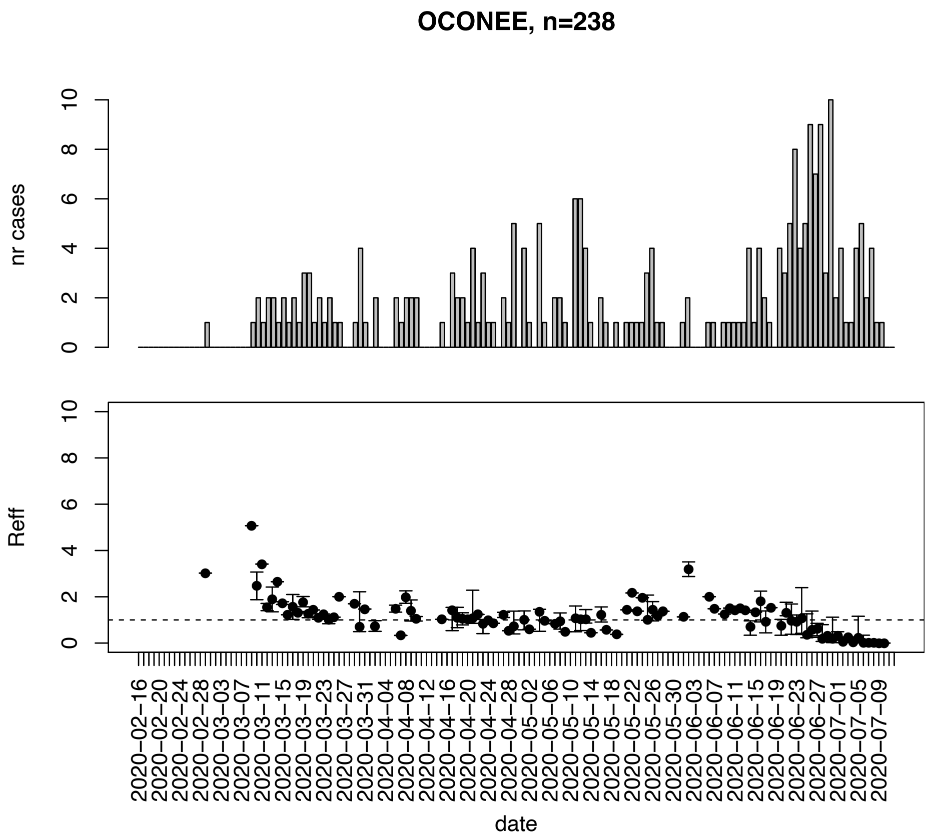

Appendix Figure 118. Epidemic curves and reproduction number estimates until July 13th in Oconee county.

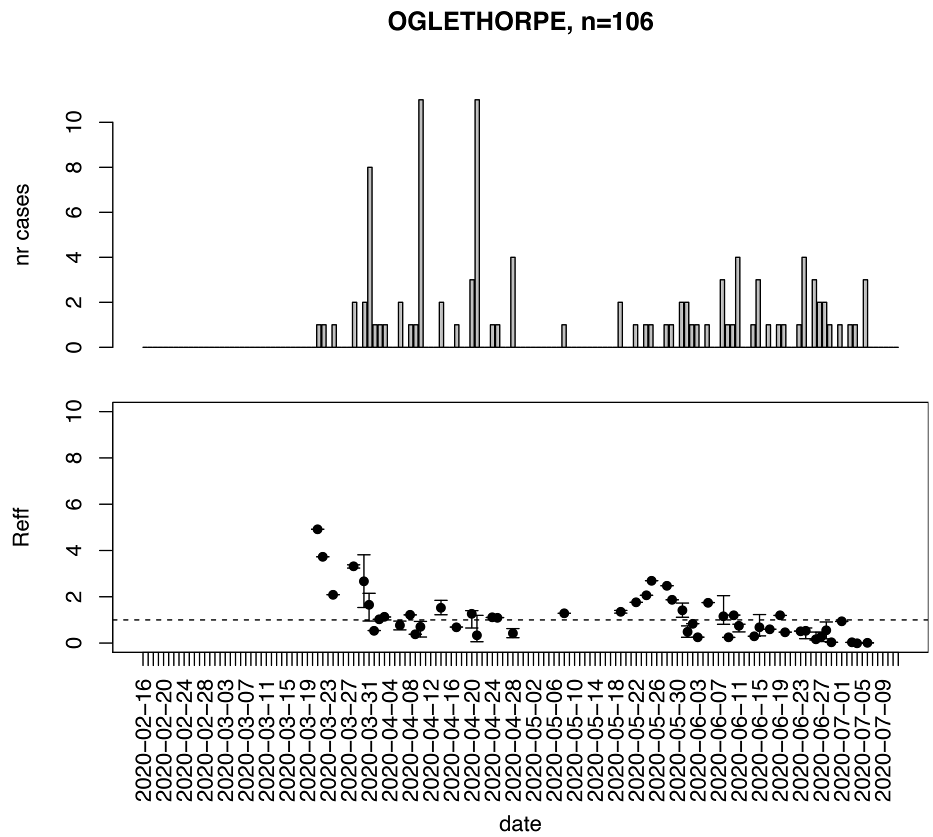

Appendix Figure 119. Epidemic curves and reproduction number estimates until July 13th in Oglethorpe county.

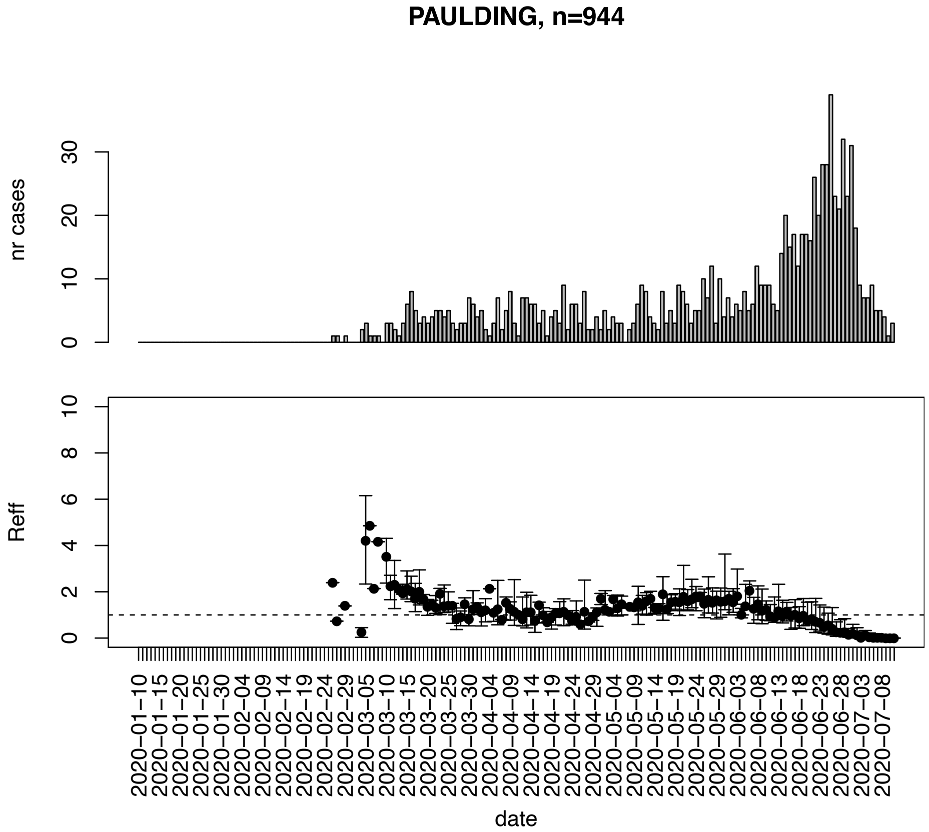

Appendix Figure 120. Epidemic curves and reproduction number estimates until July 13th in Paulding county.

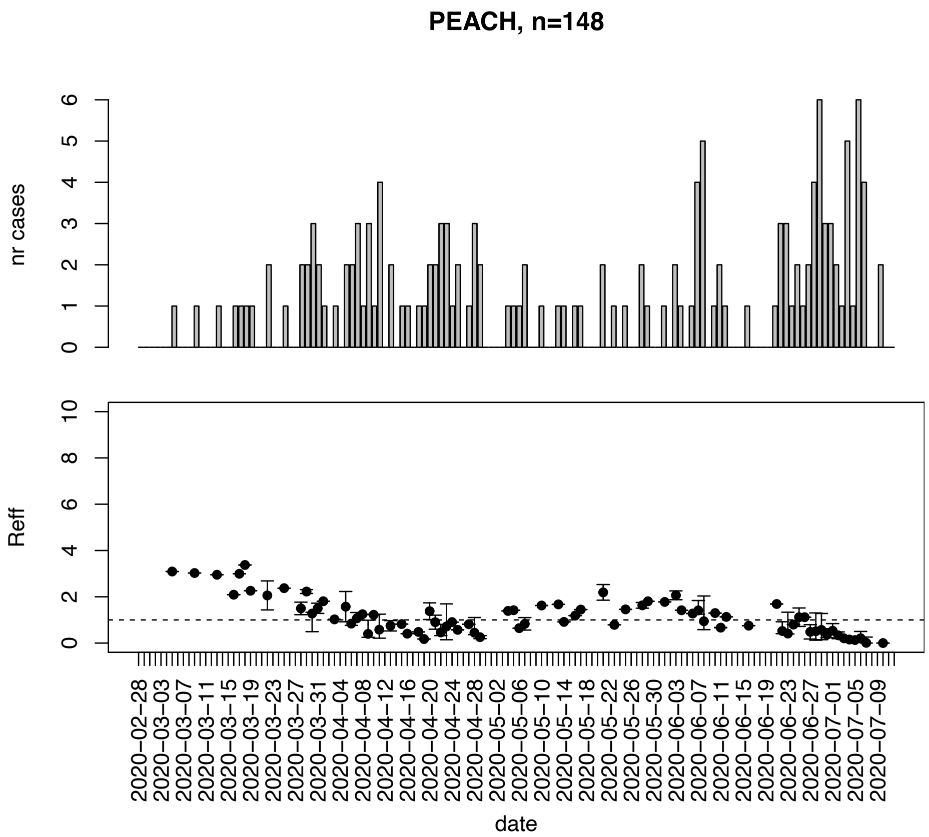

Appendix Figure 121. Epidemic curves and reproduction number estimates until July 13th in Peach county.

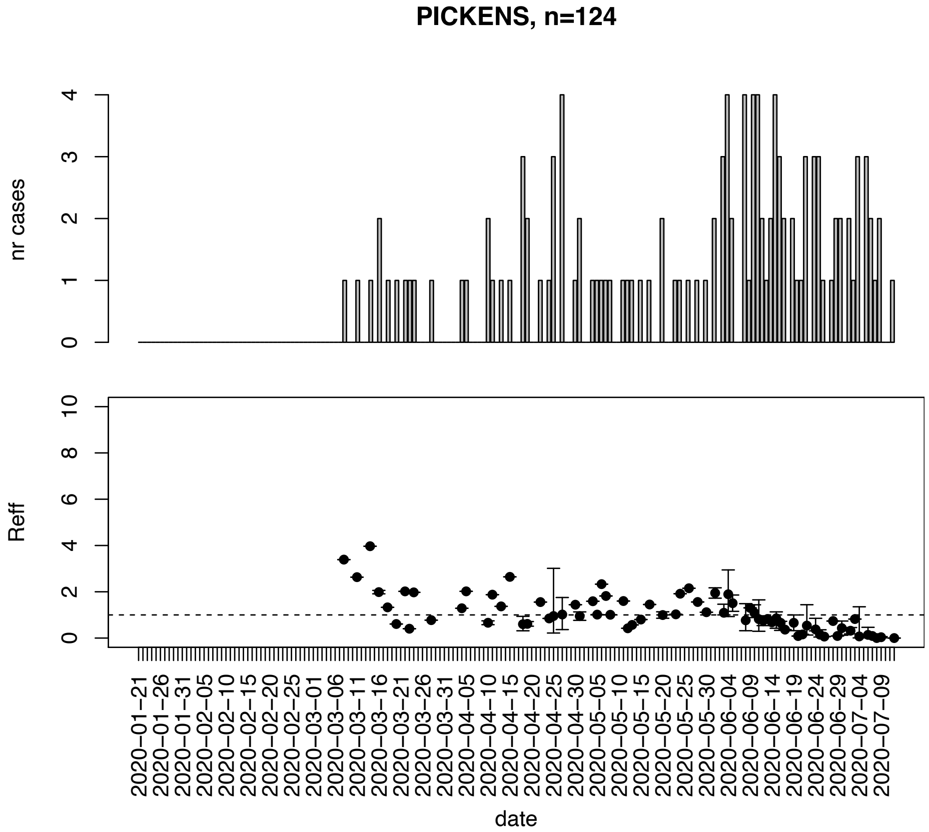

Appendix Figure 122. Epidemic curves and reproduction number estimates until July 13th in Pickens county.

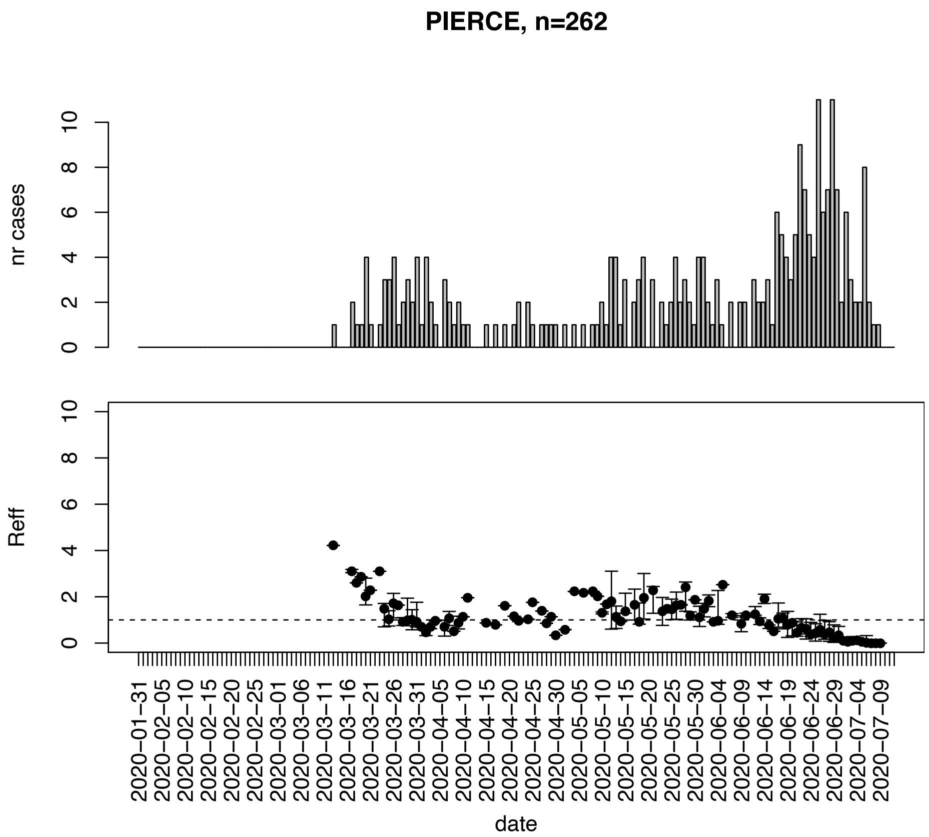

Appendix Figure 123. Epidemic curves and reproduction number estimates until July 13th in Pierce county.

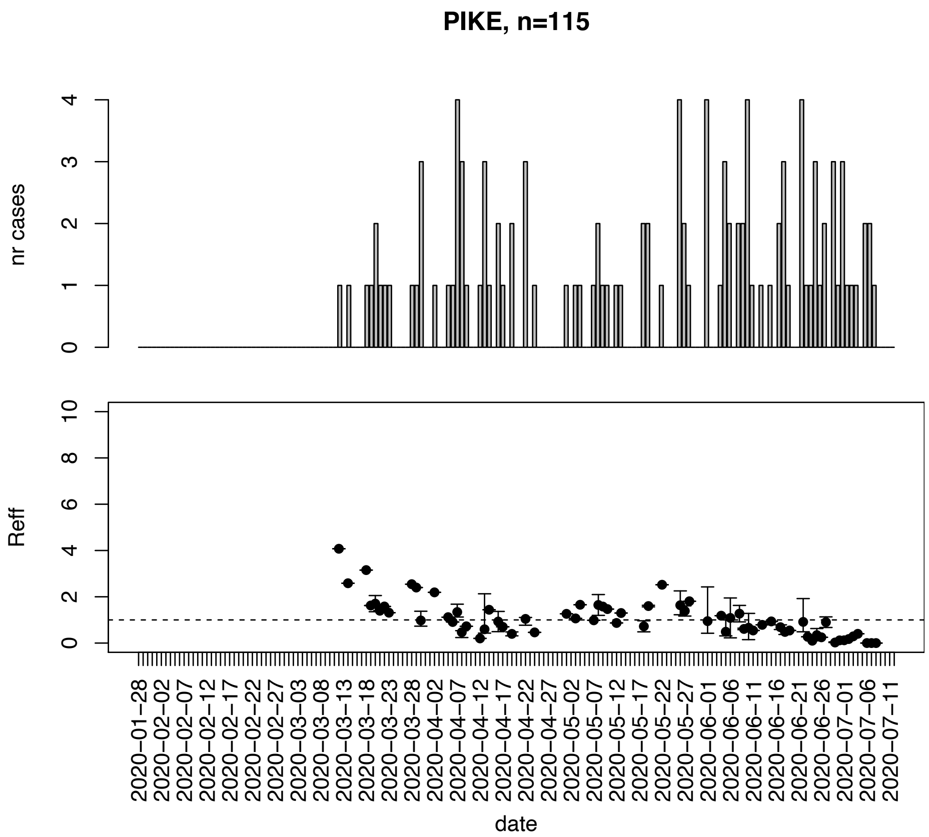

Appendix Figure 124. Epidemic curves and reproduction number estimates until July 13th in Pike county.

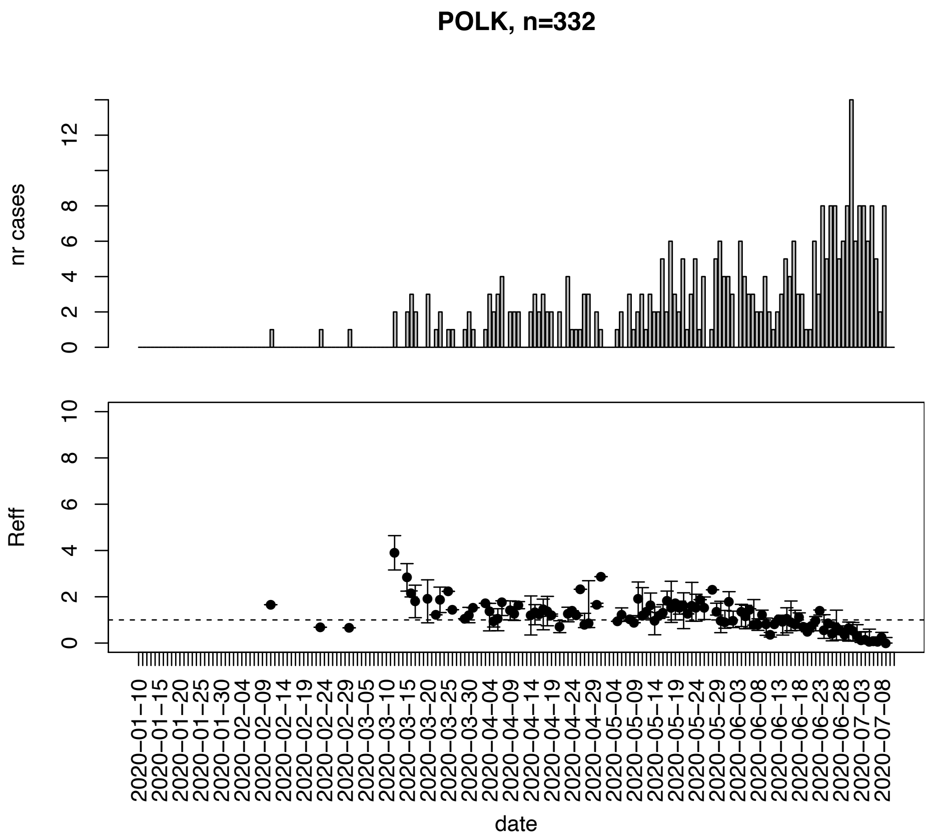

Appendix Figure 125. Epidemic curves and reproduction number estimates until July 13th in Polk county.

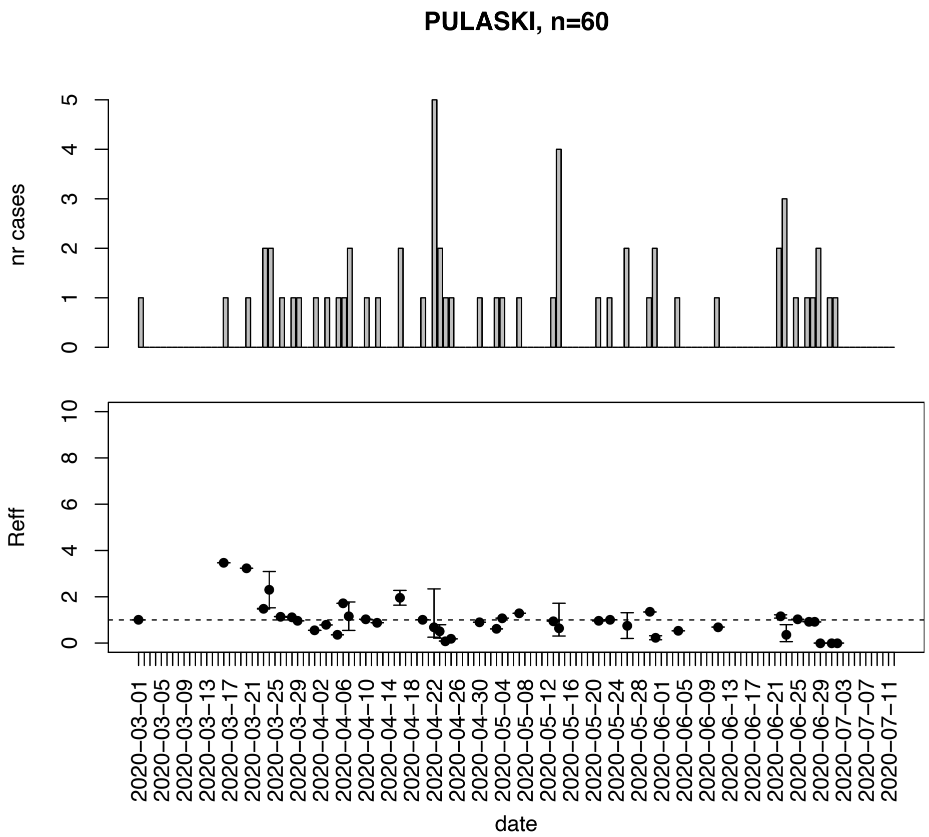

Appendix Figure 126. Epidemic curves and reproduction number estimates until July 13th in Pulaski county.

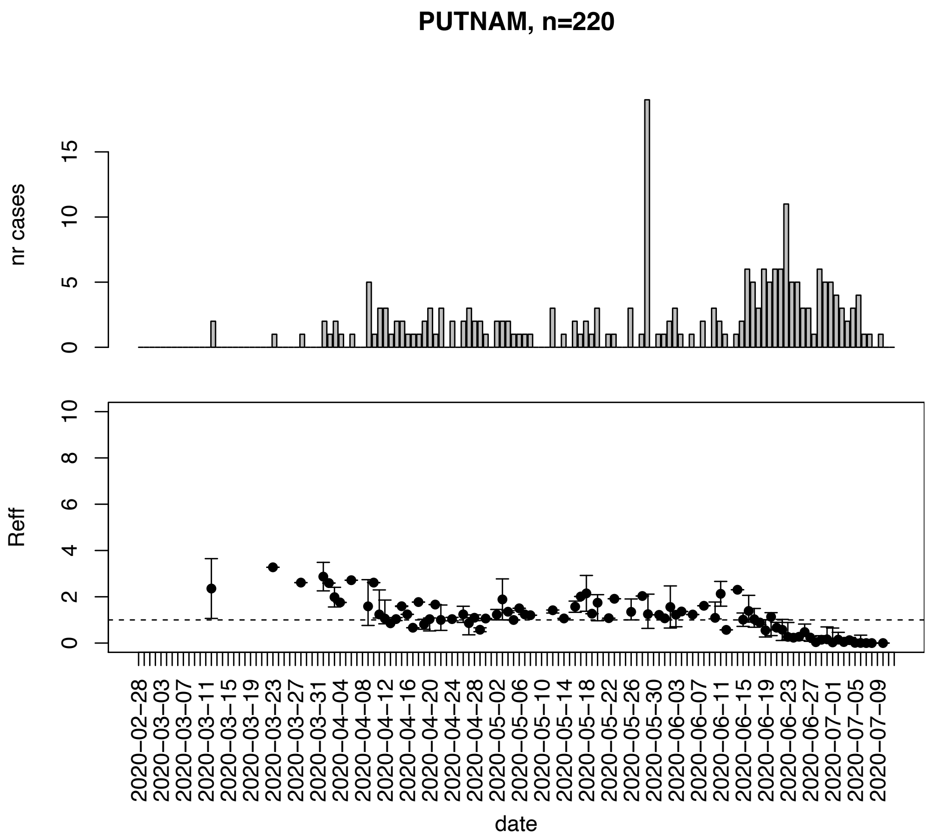

Appendix Figure 127. Epidemic curves and reproduction number estimates until July 13th in Putnam county.

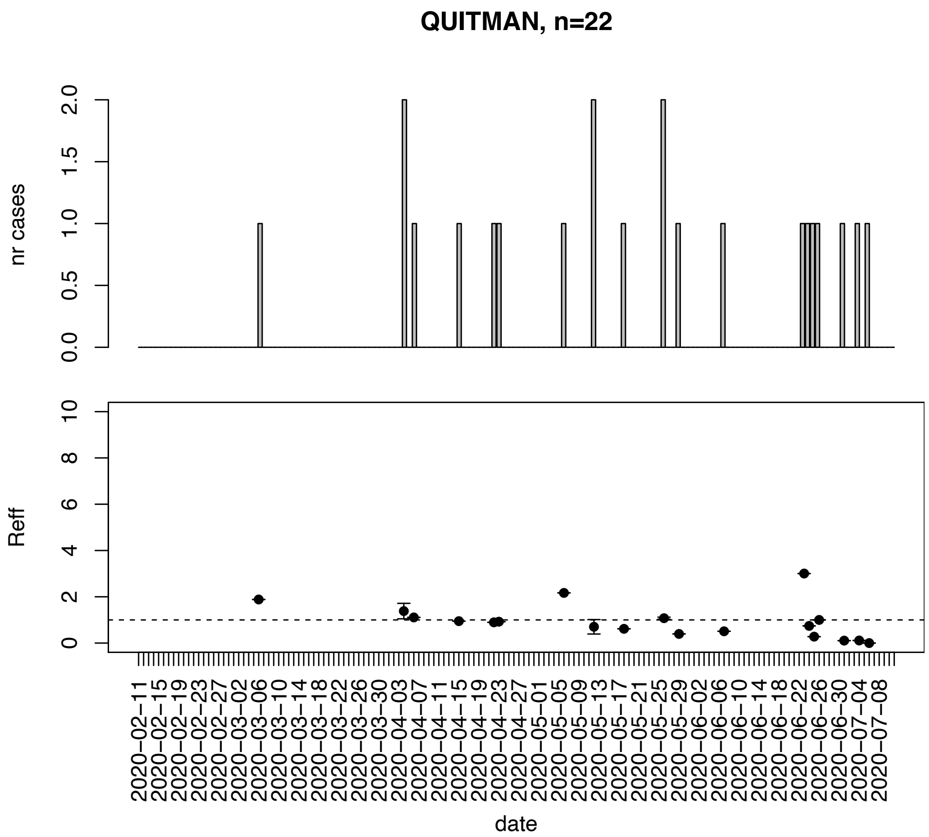

Appendix Figure 128. Epidemic curves and reproduction number estimates until July 13th in Quitman county.

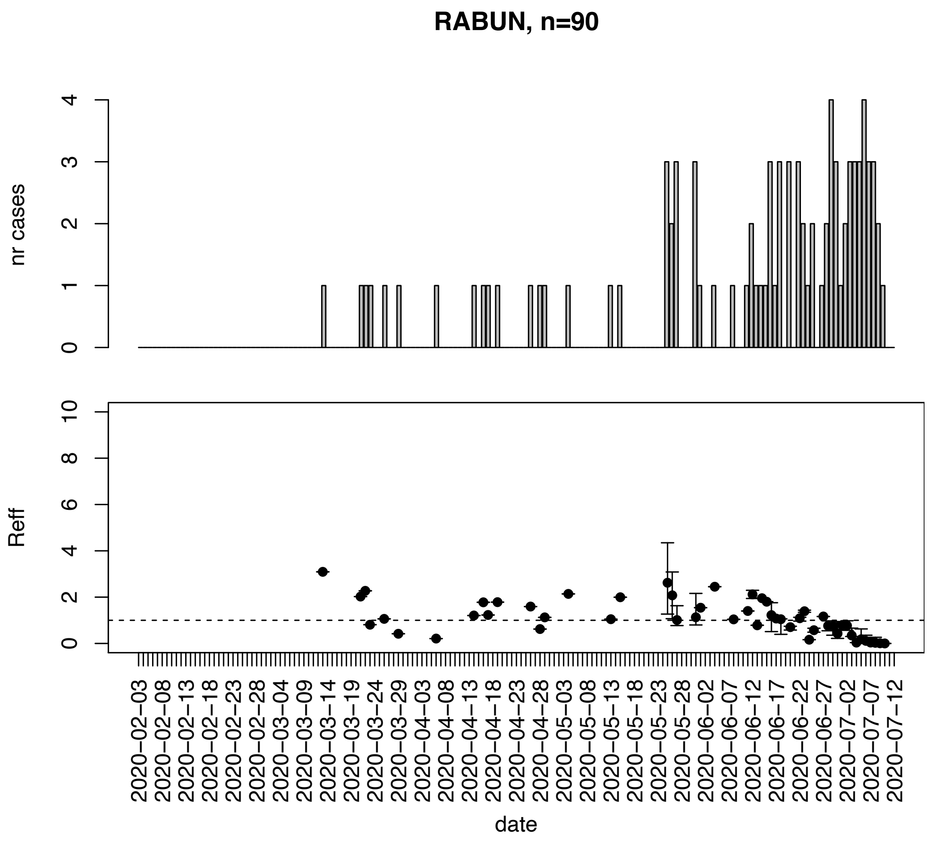

Appendix Figure 129. Epidemic curves and reproduction number estimates until July 13th in Rabun county.

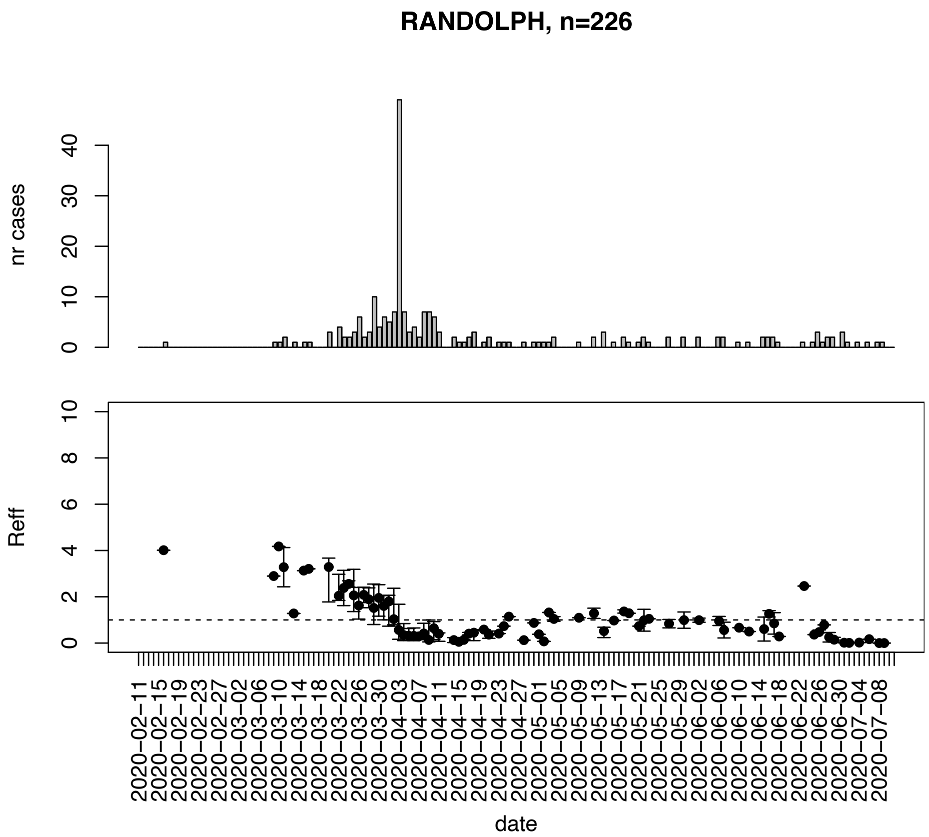

Appendix Figure 130. Epidemic curves and reproduction number estimates until July 13th in Randolph county.

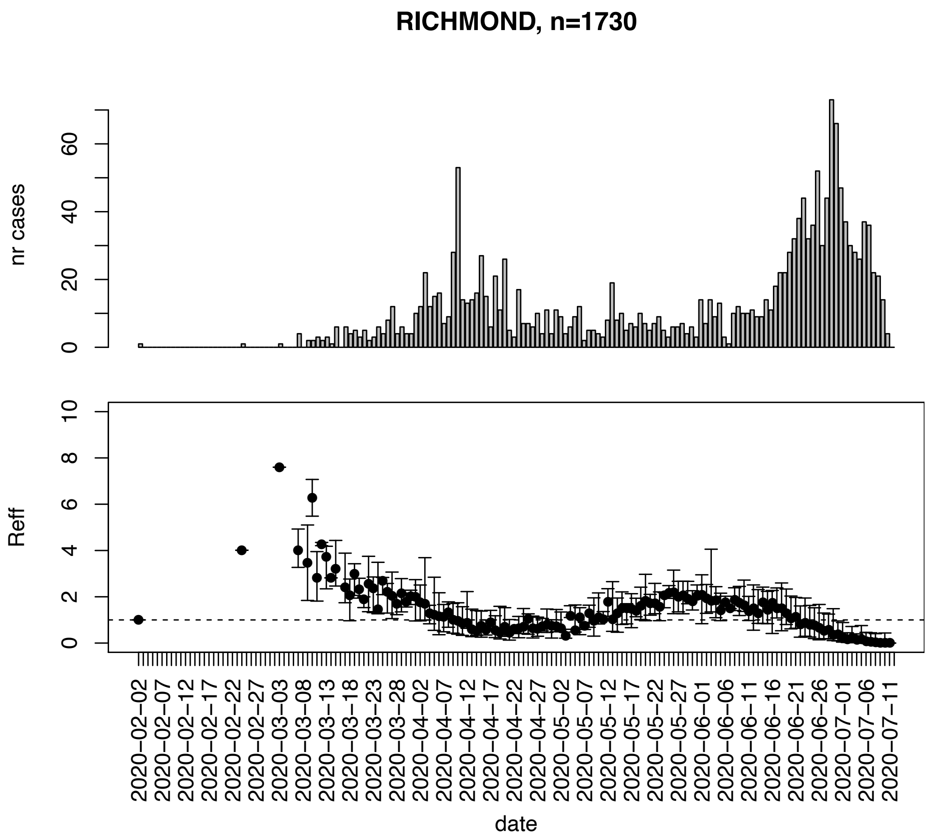

Appendix Figure 131. Epidemic curves and reproduction number estimates until July 13th in Richmond county.

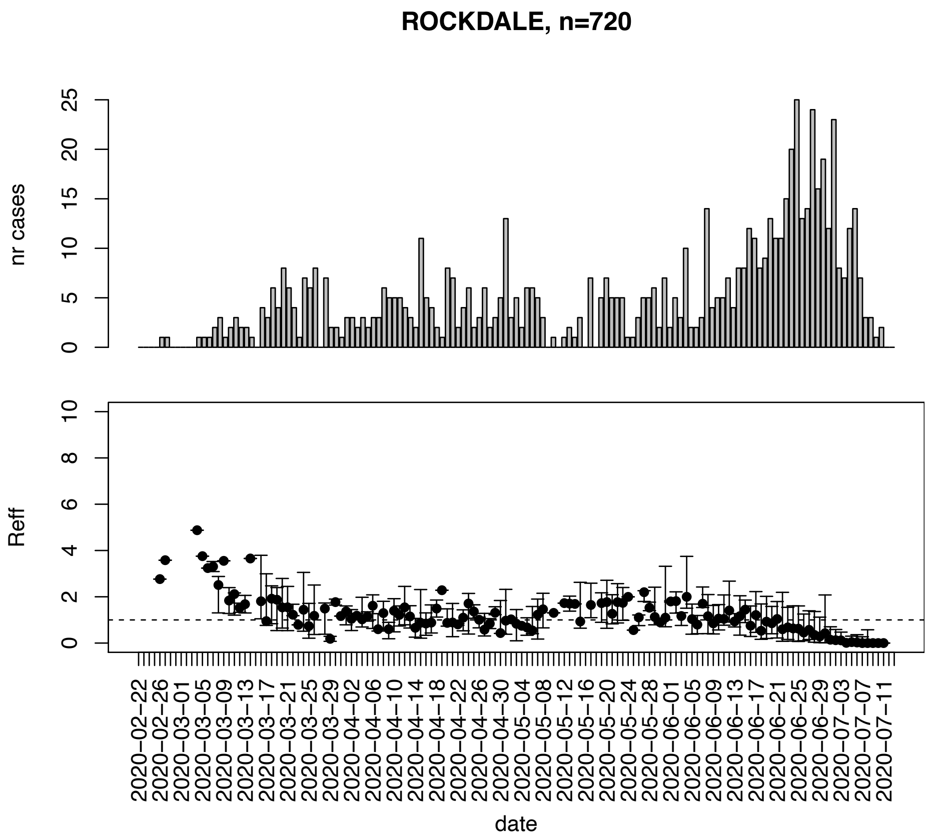

Appendix Figure 132. Epidemic curves and reproduction number estimates until July 13th in Rockdale county.

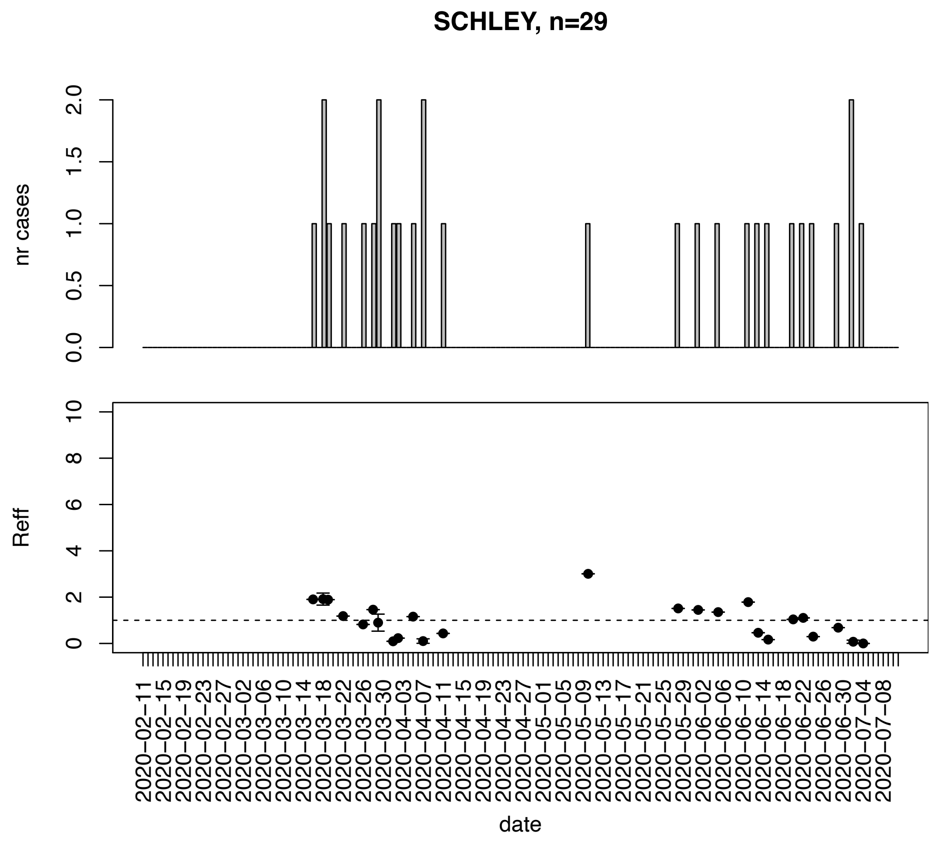

Appendix Figure 133. Epidemic curves and reproduction number estimates until July 13th in Schley county.

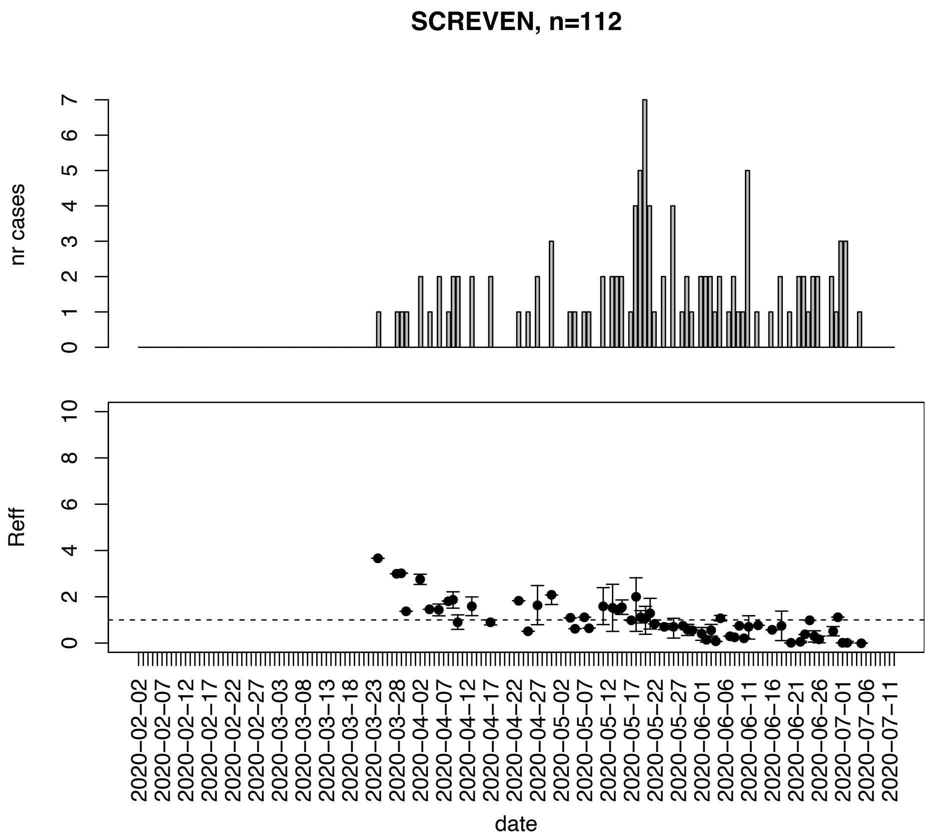

Appendix Figure 134. Epidemic curves and reproduction number estimates until July 13th in Screven county.

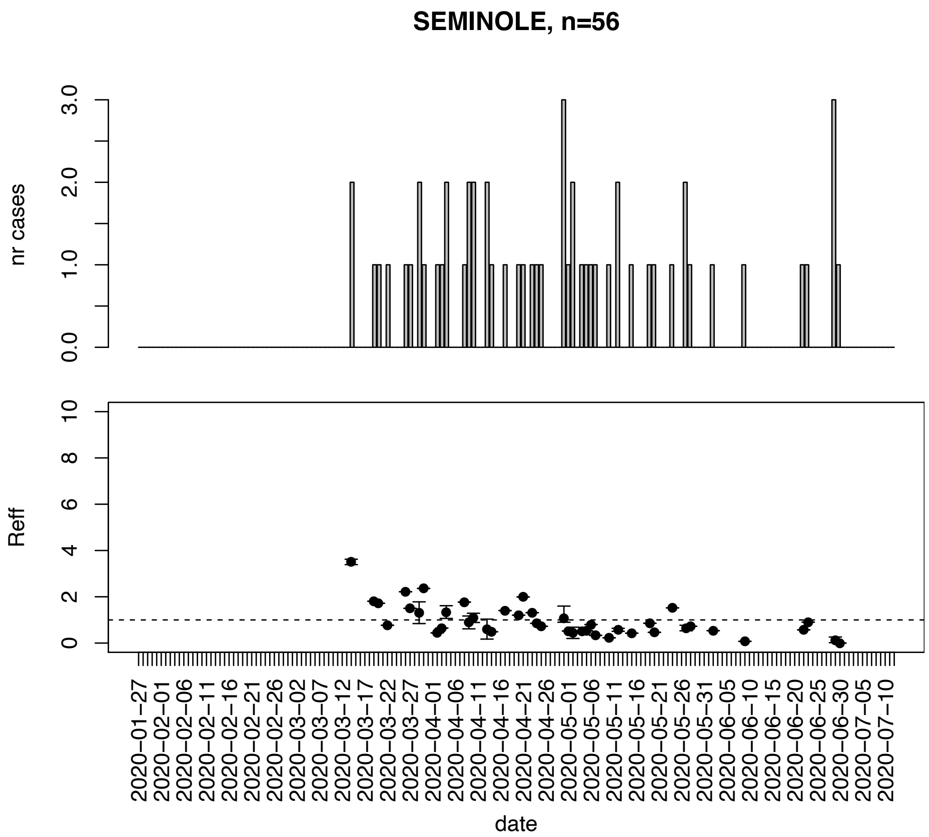

Appendix Figure 135. Epidemic curves and reproduction number estimates until July 13th in Seminole county.

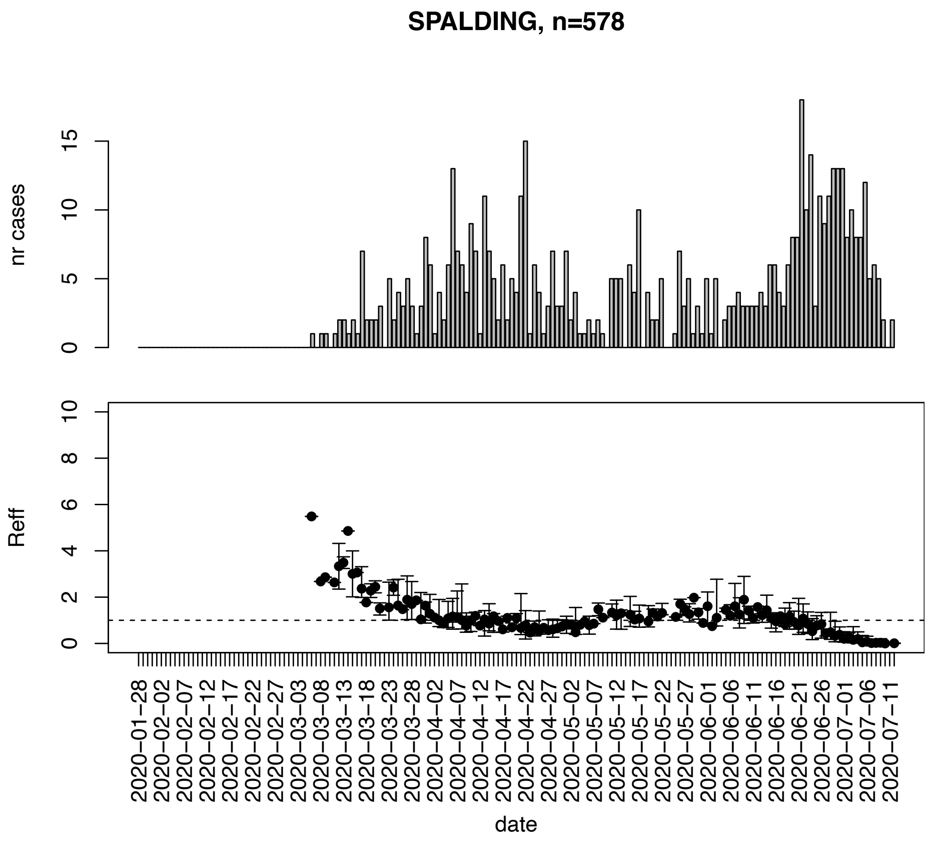

Appendix Figure 136. Epidemic curves and reproduction number estimates until July 13th in Spalding county.

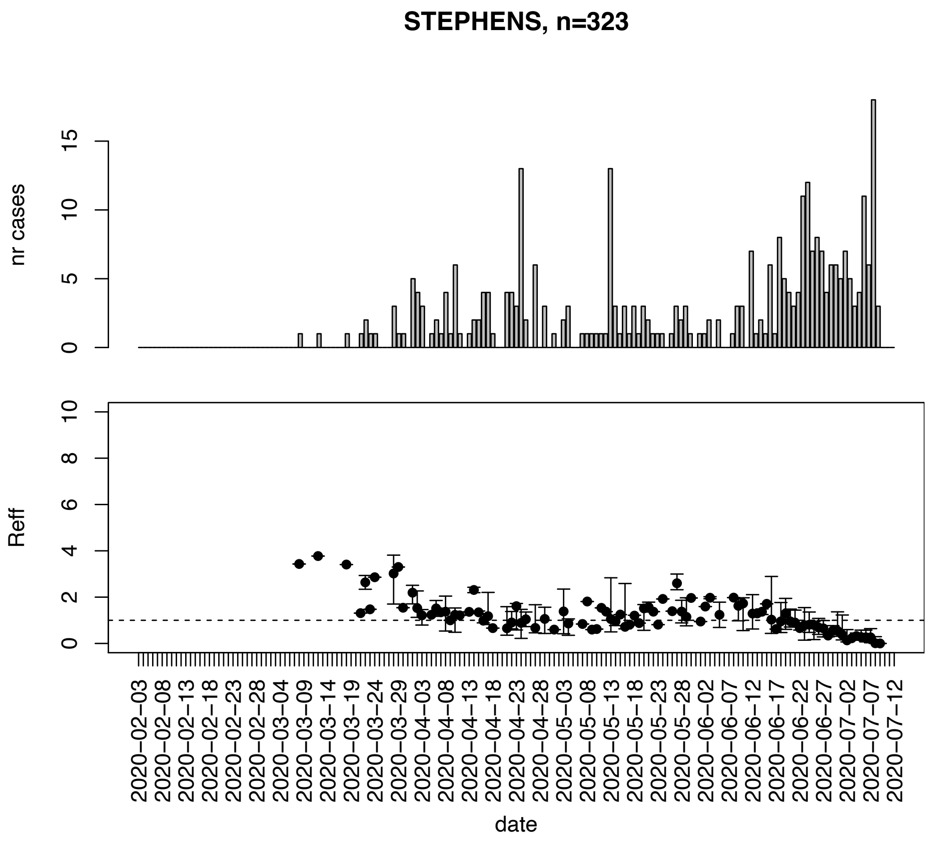

Appendix Figure 137. Epidemic curves and reproduction number estimates until July 13th in Stephens county.

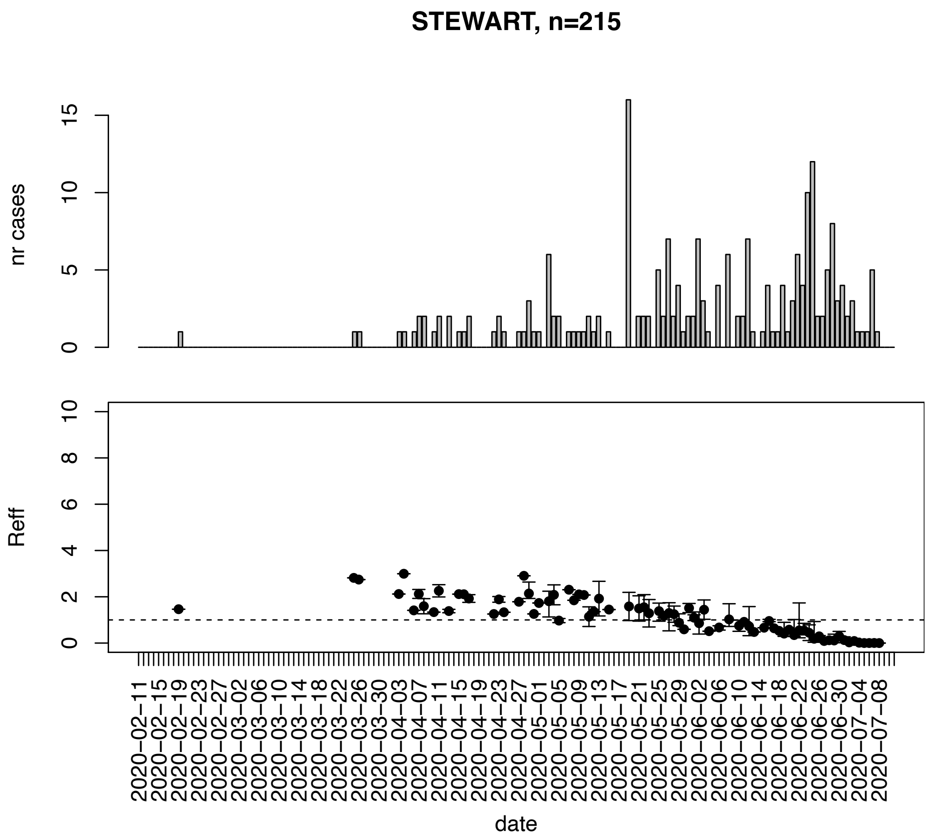

Appendix Figure 138. Epidemic curves and reproduction number estimates until July 13th in Stewart county.

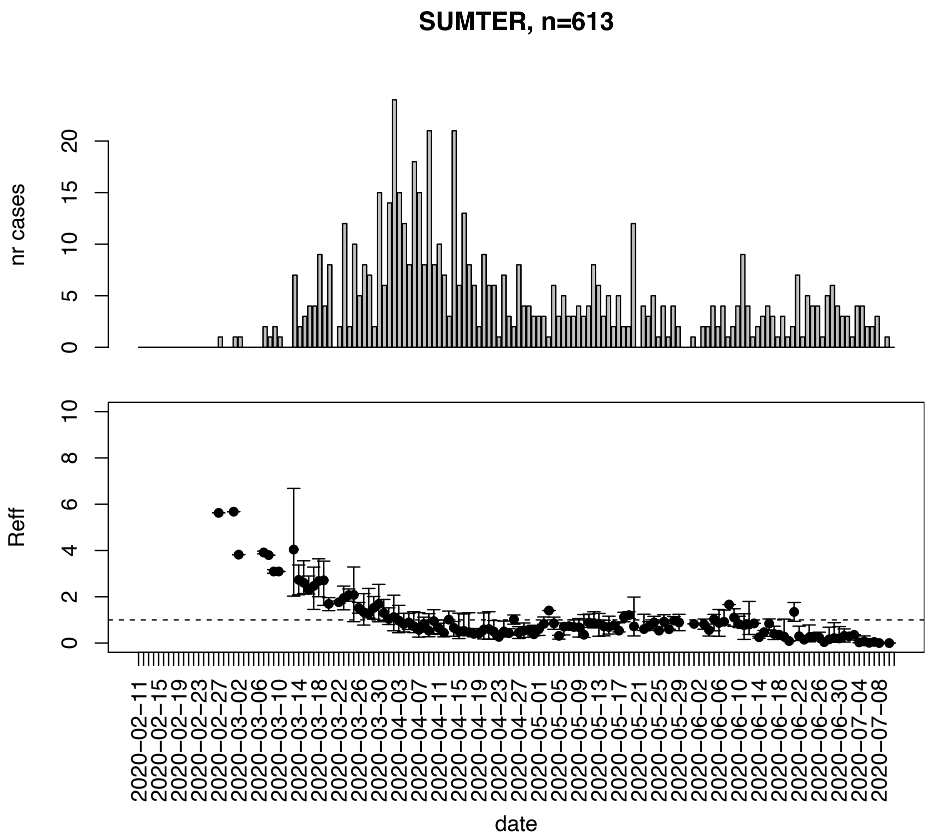

Appendix Figure 139. Epidemic curves and reproduction number estimates until July 13th in Sumter county.

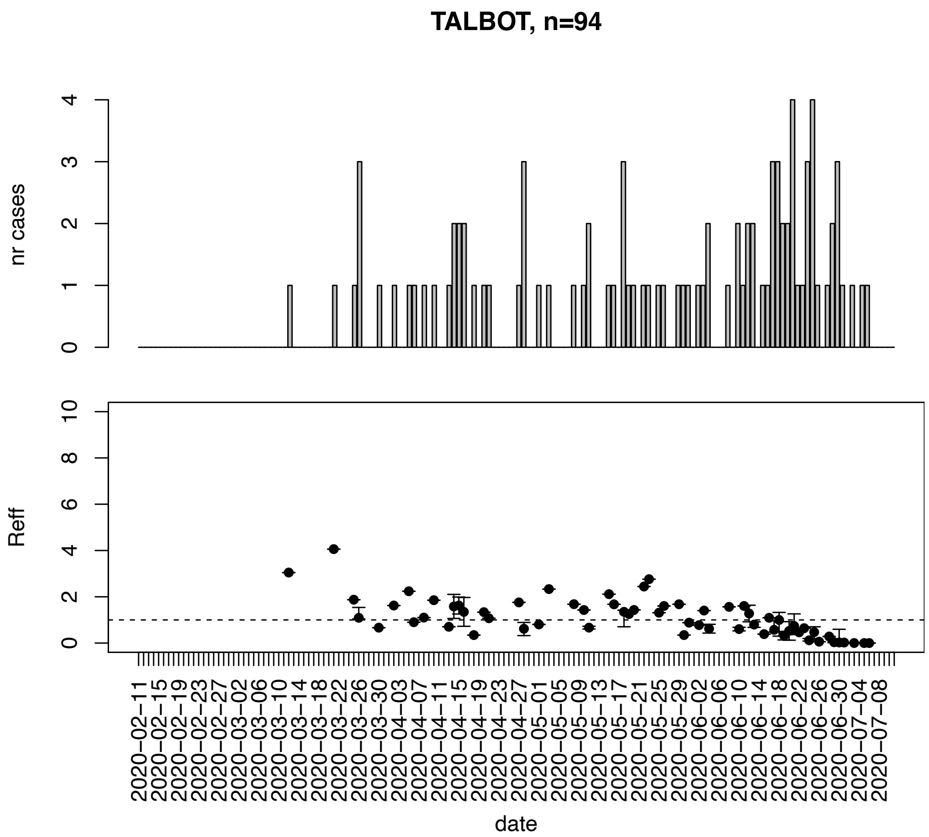

Appendix Figure 140. Epidemic curves and reproduction number estimates until July 13th in Talbot county.

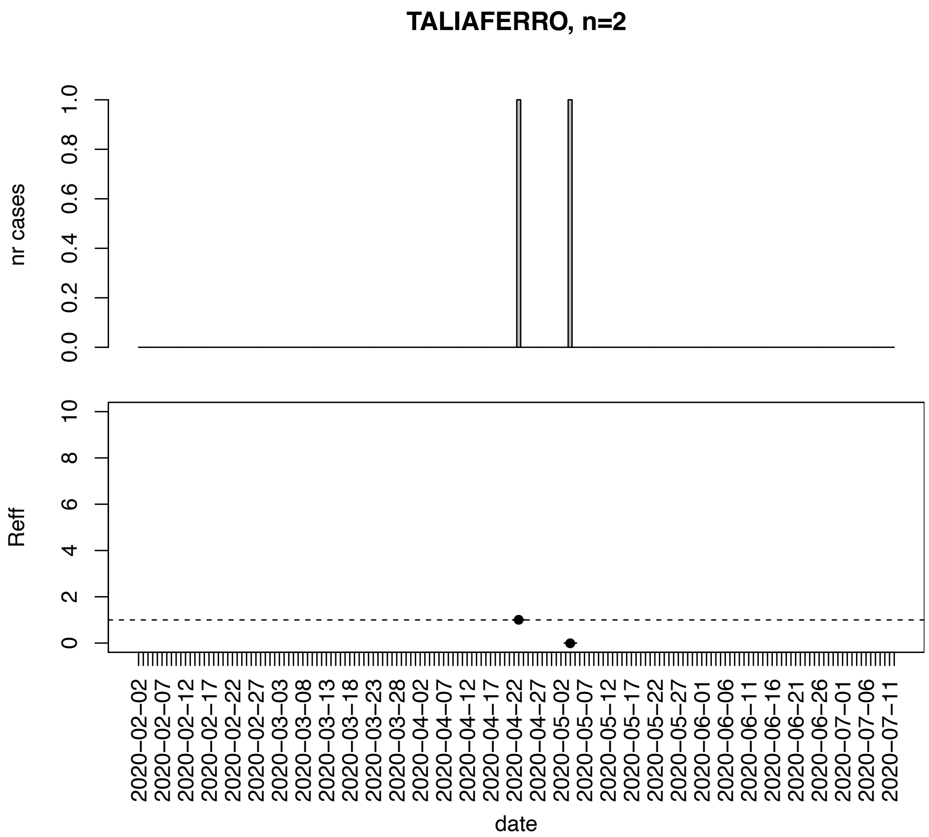

Appendix Figure 141. Epidemic curves and reproduction number estimates until July 13th in Taliaferro county.

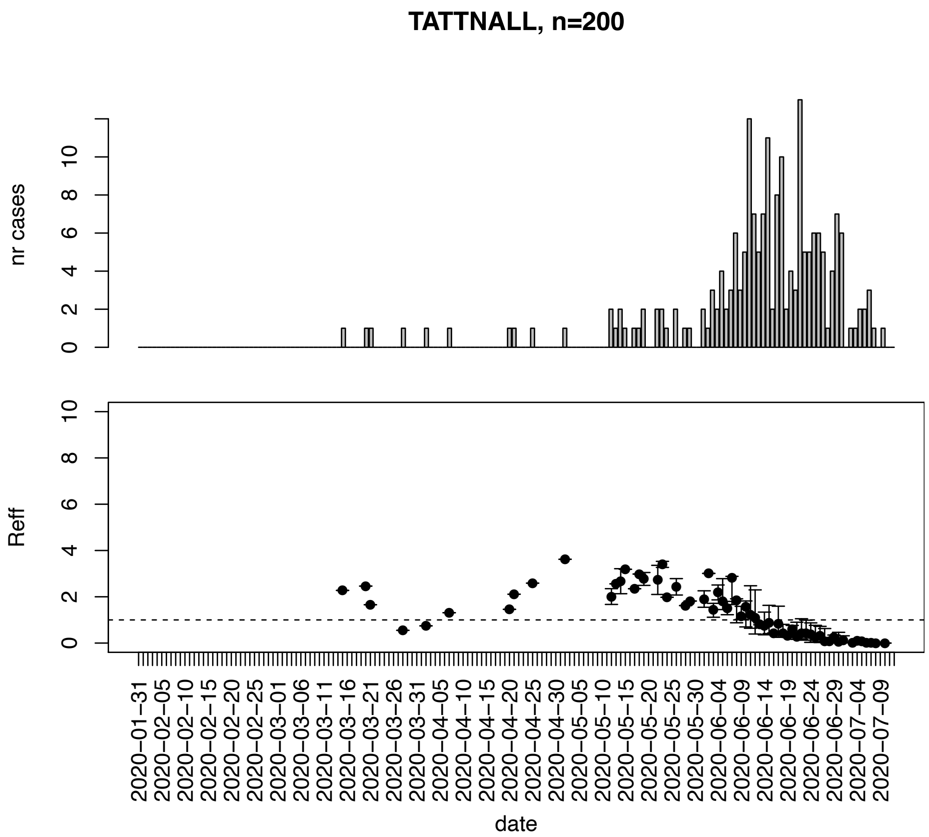

Appendix Figure 142. Epidemic curves and reproduction number estimates until July 13th in Tattnall county.

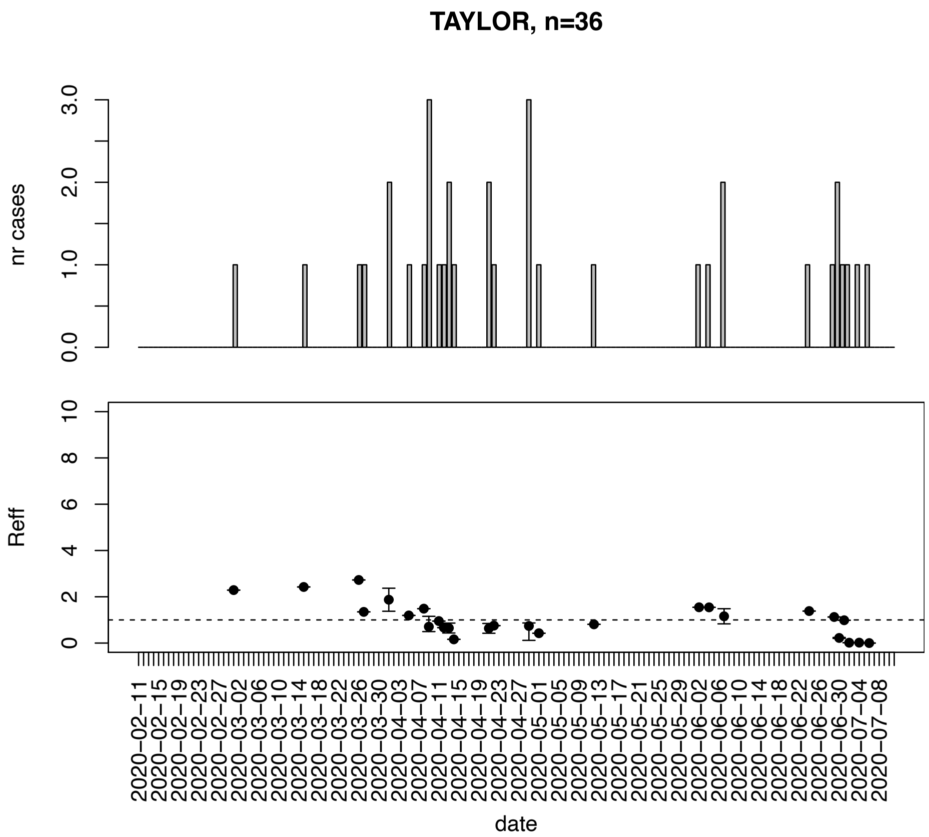

Appendix Figure 143. Epidemic curves and reproduction number estimates until July 13th in Taylor county.

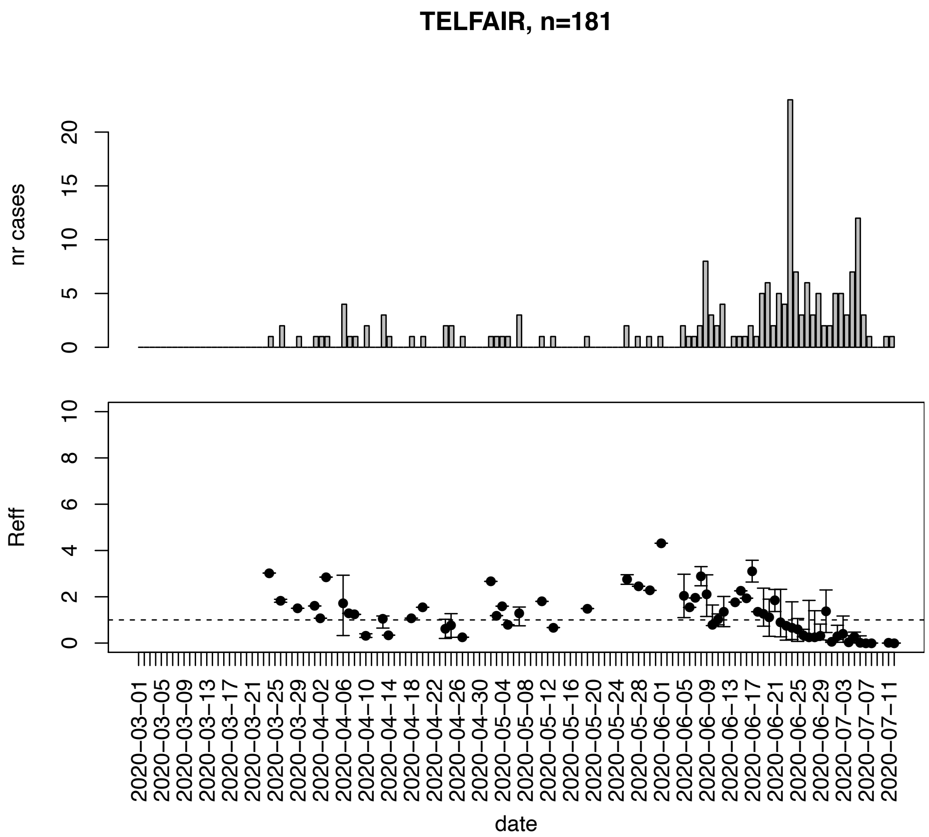

Appendix Figure 144. Epidemic curves and reproduction number estimates until July 13th in Telfair county.

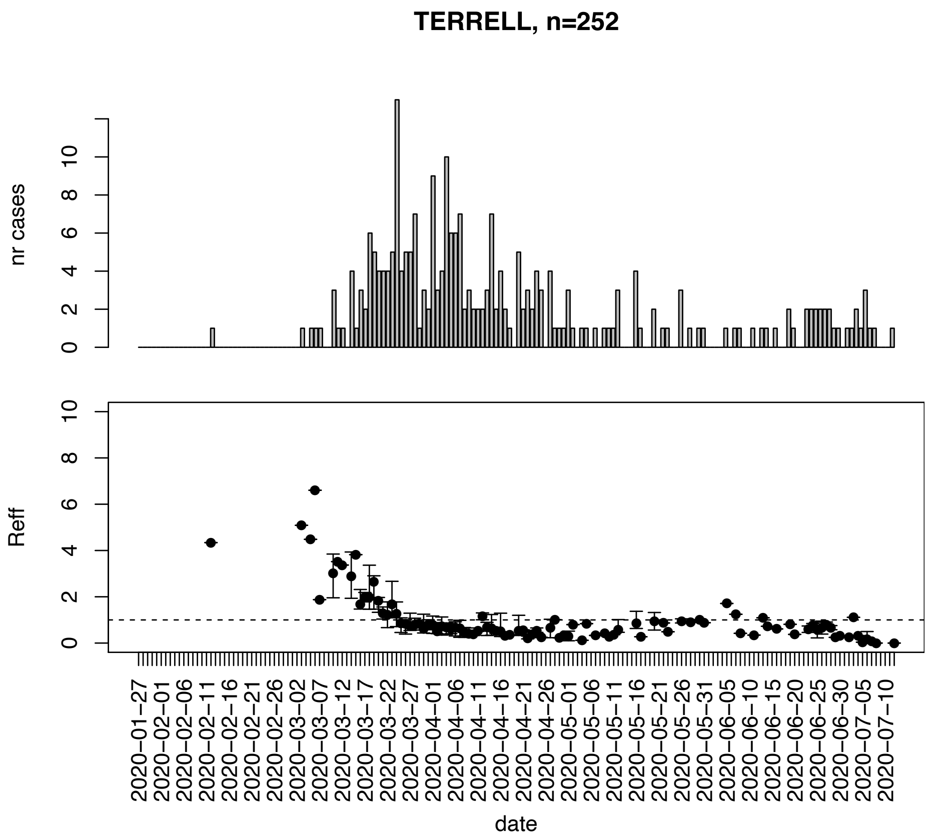

Appendix Figure 145. Epidemic curves and reproduction number estimates until July 13th in Terrell county.

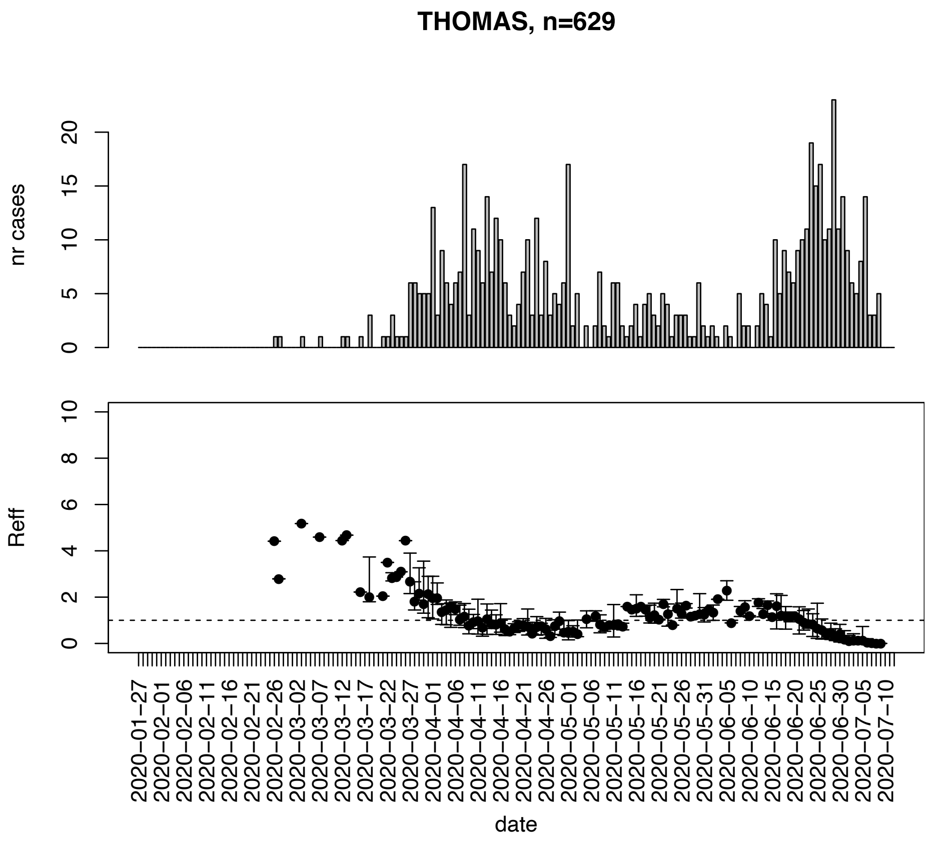

Appendix Figure 146. Epidemic curves and reproduction number estimates until July 13th in Thomas county.

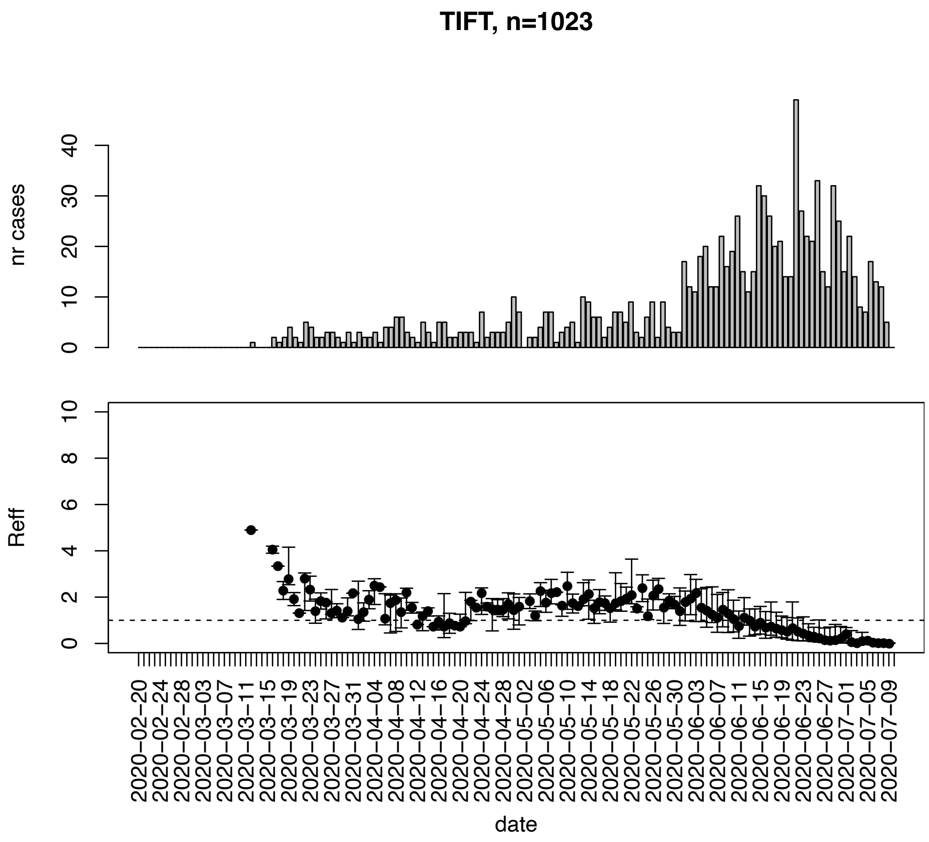

Appendix Figure 147. Epidemic curves and reproduction number estimates until July 13th in Tift county.

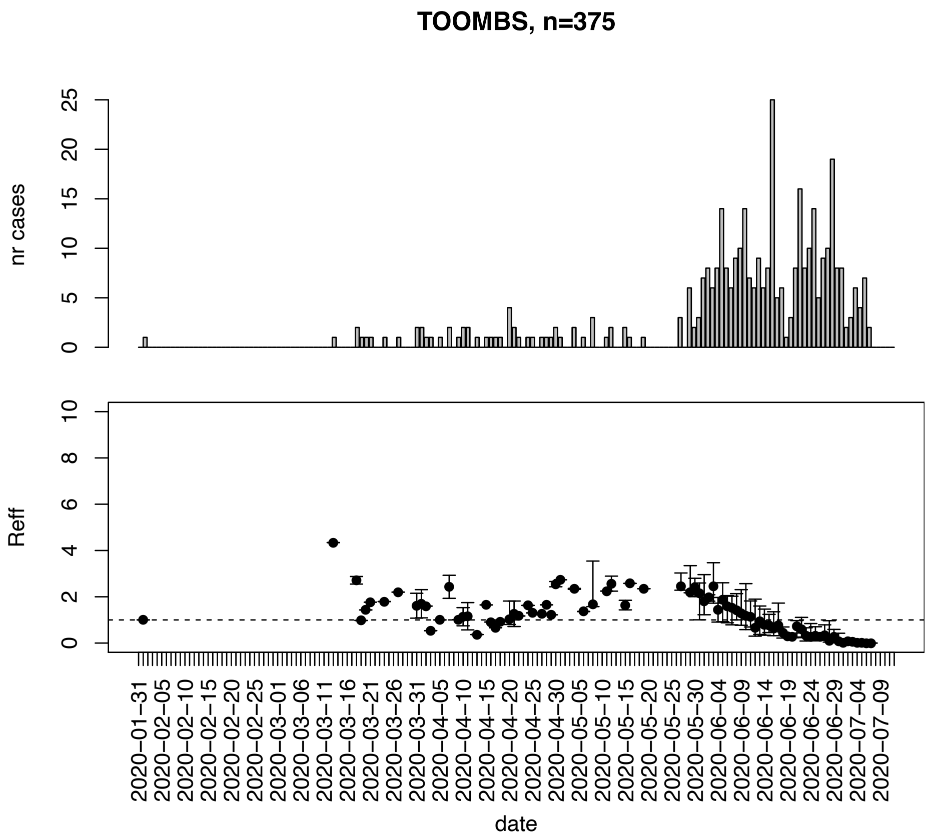

Appendix Figure 148. Epidemic curves and reproduction number estimates until July 13th in Toombs county.

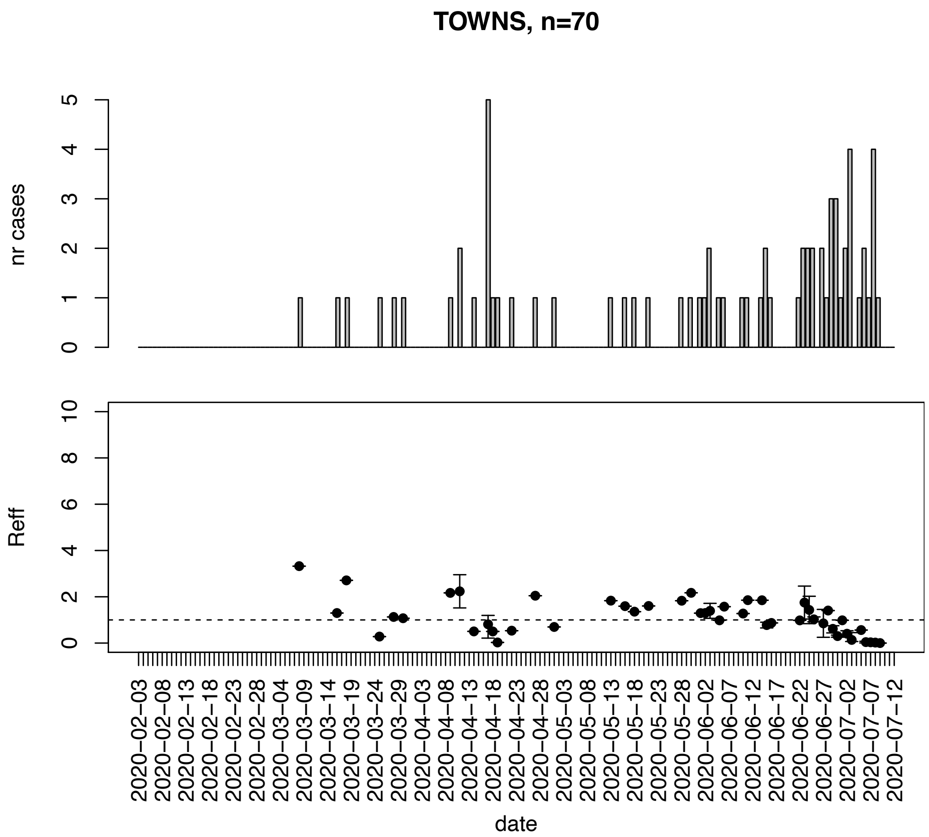

Appendix Figure 149. Epidemic curves and reproduction number estimates until July 13th in Towns county.

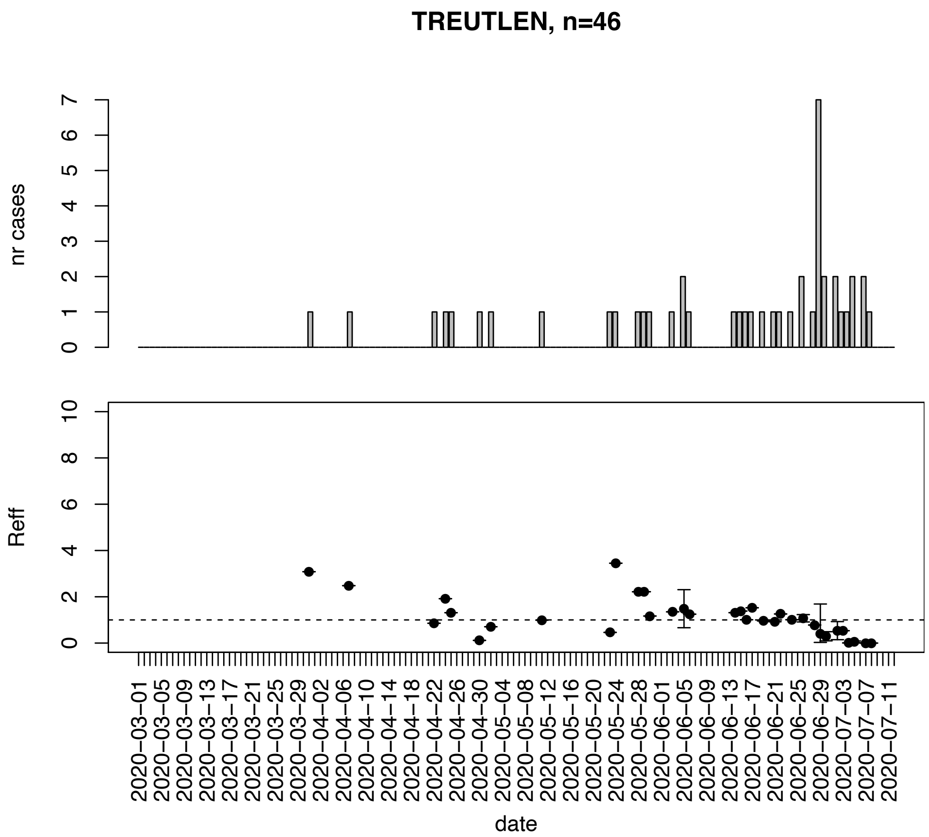

Appendix Figure 150. Epidemic curves and reproduction number estimates until July 13th in Treutlen county.

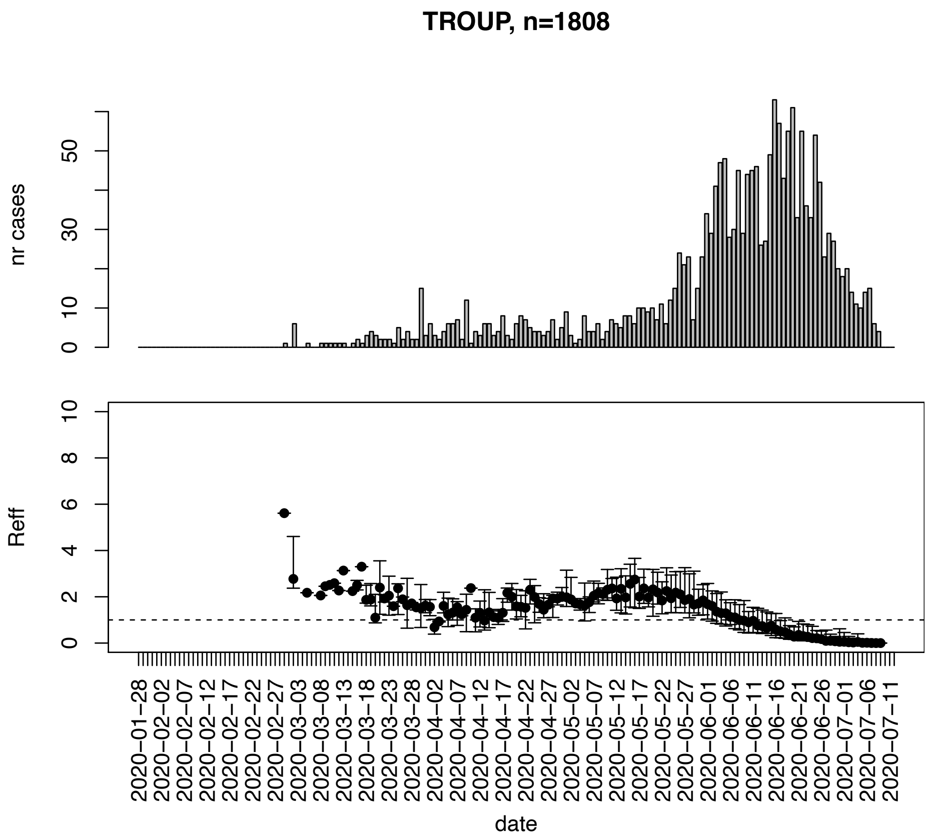

Appendix Figure 151. Epidemic curves and reproduction number estimates until July 13th in Troup county.

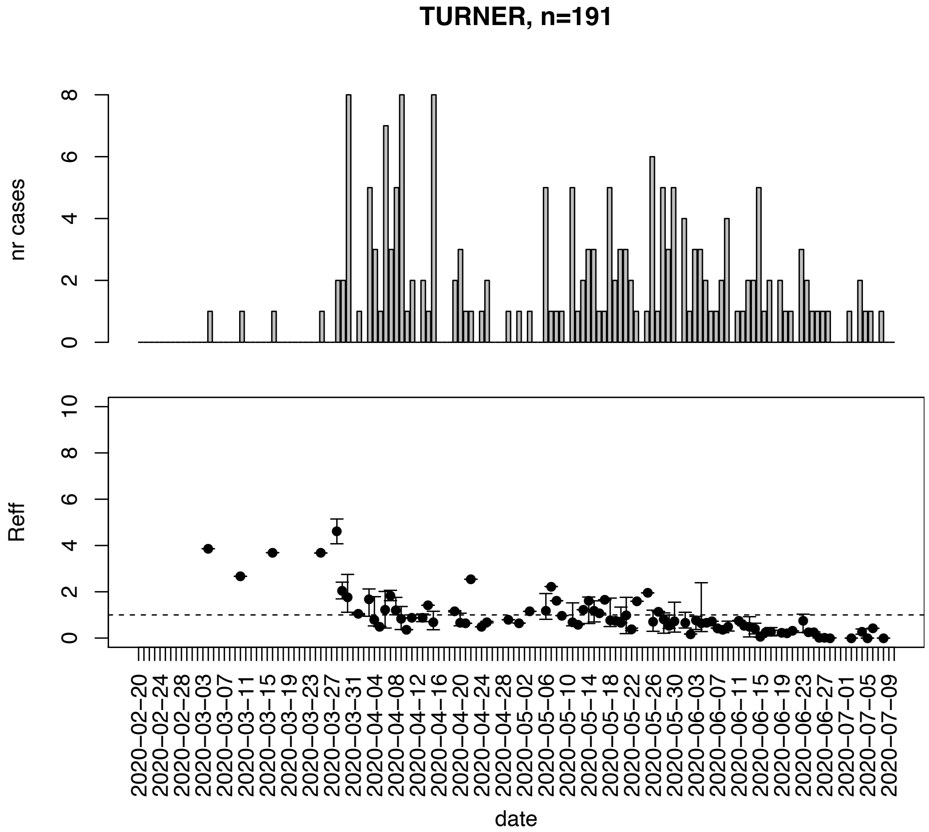

Appendix Figure 152. Epidemic curves and reproduction number estimates until July 13th in Turner county.

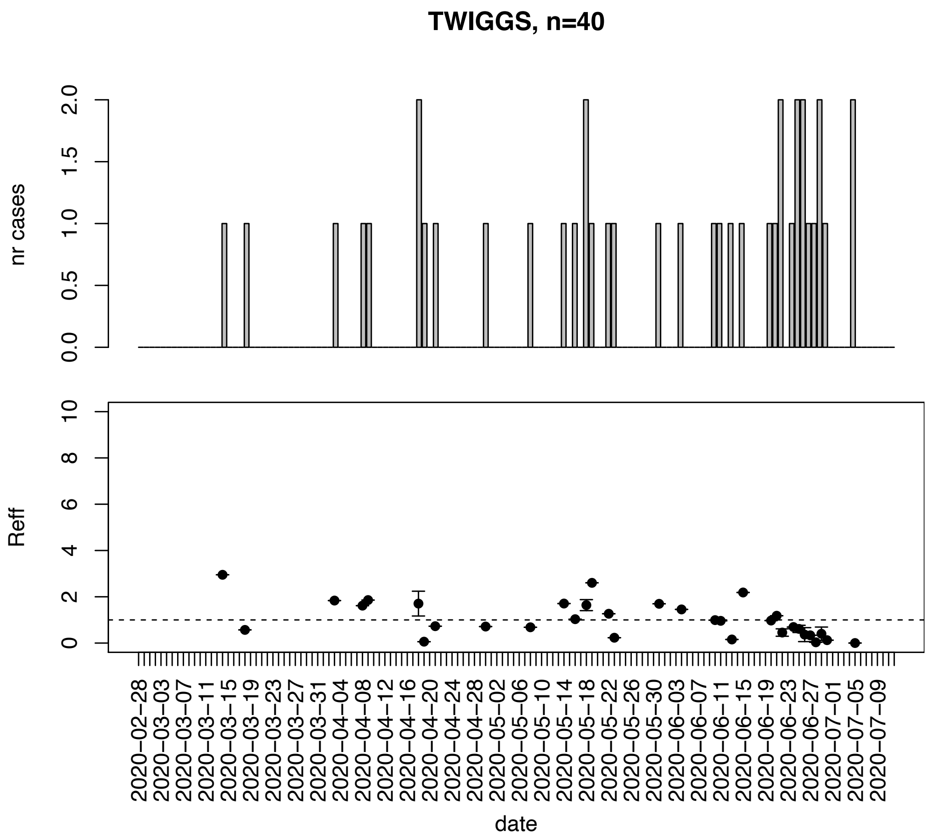

Appendix Figure 153. Epidemic curves and reproduction number estimates until July 13th in Twiggs county.

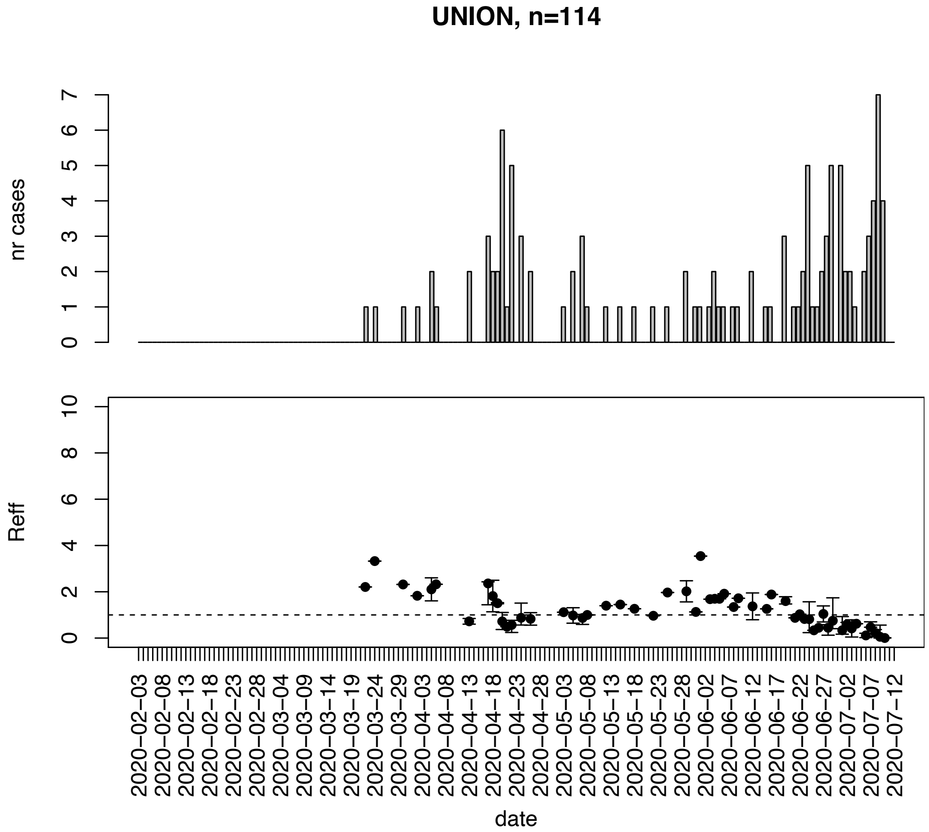

Appendix Figure 154. Epidemic curves and reproduction number estimates until July 13th in Union county.

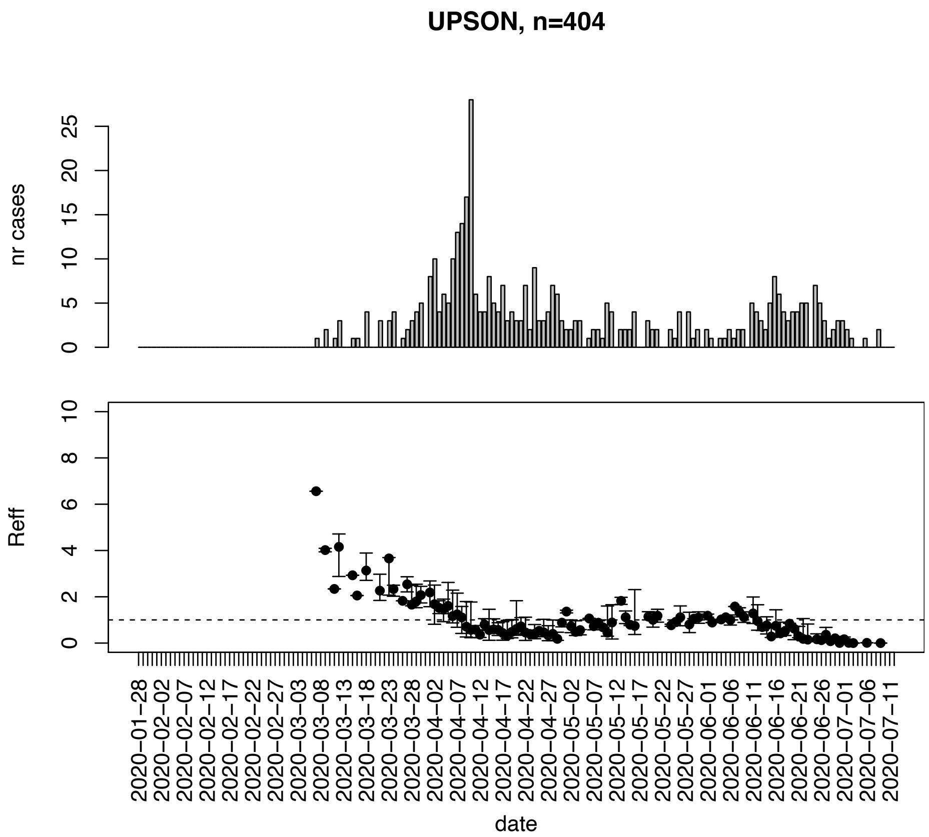

Appendix Figure 155. Epidemic curves and reproduction number estimates until July 13th in Upson county.

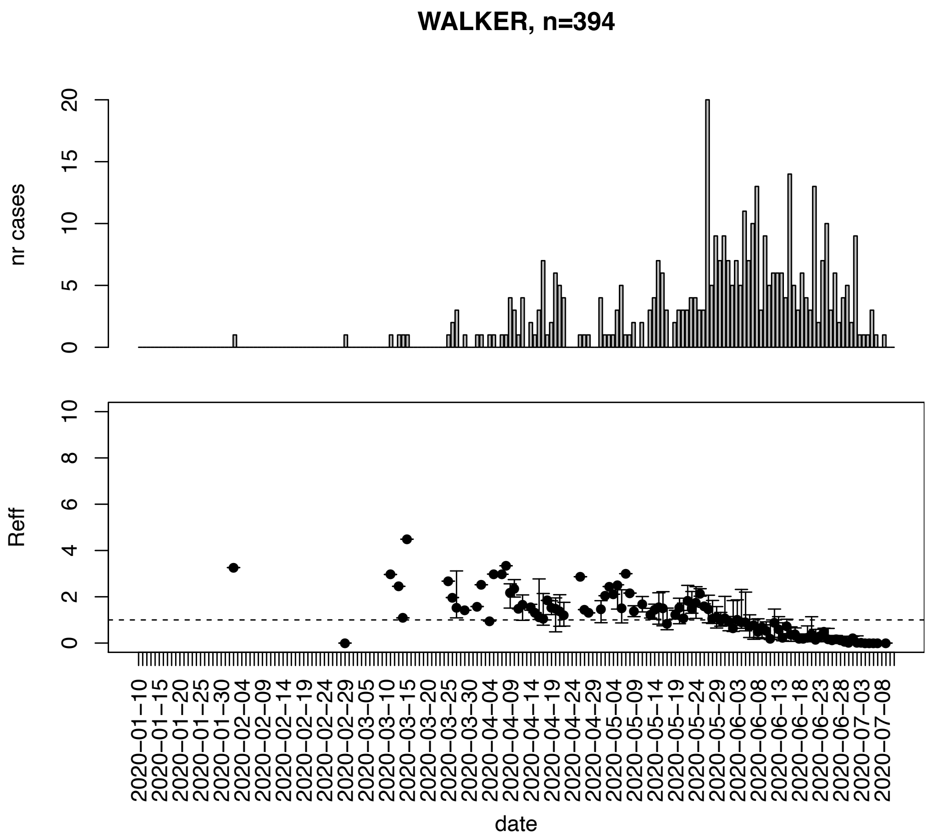

Appendix Figure 156. Epidemic curves and reproduction number estimates until July 13th in Walker county.

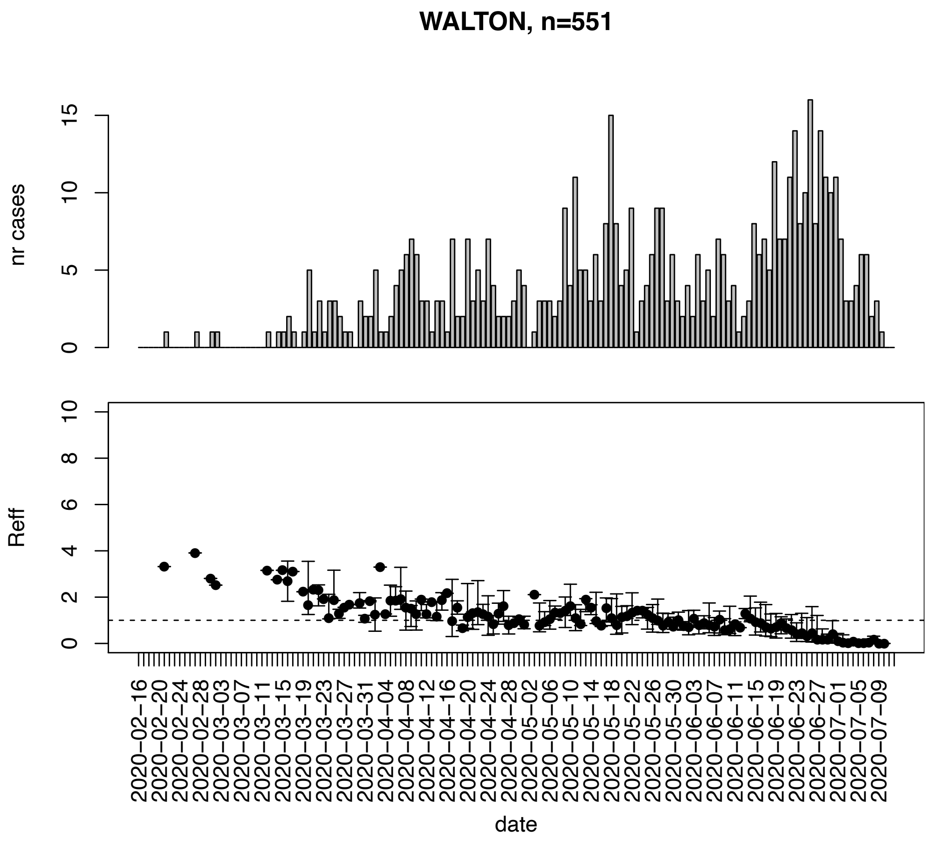

Appendix Figure 157. Epidemic curves and reproduction number estimates until July 13th in Walton county.

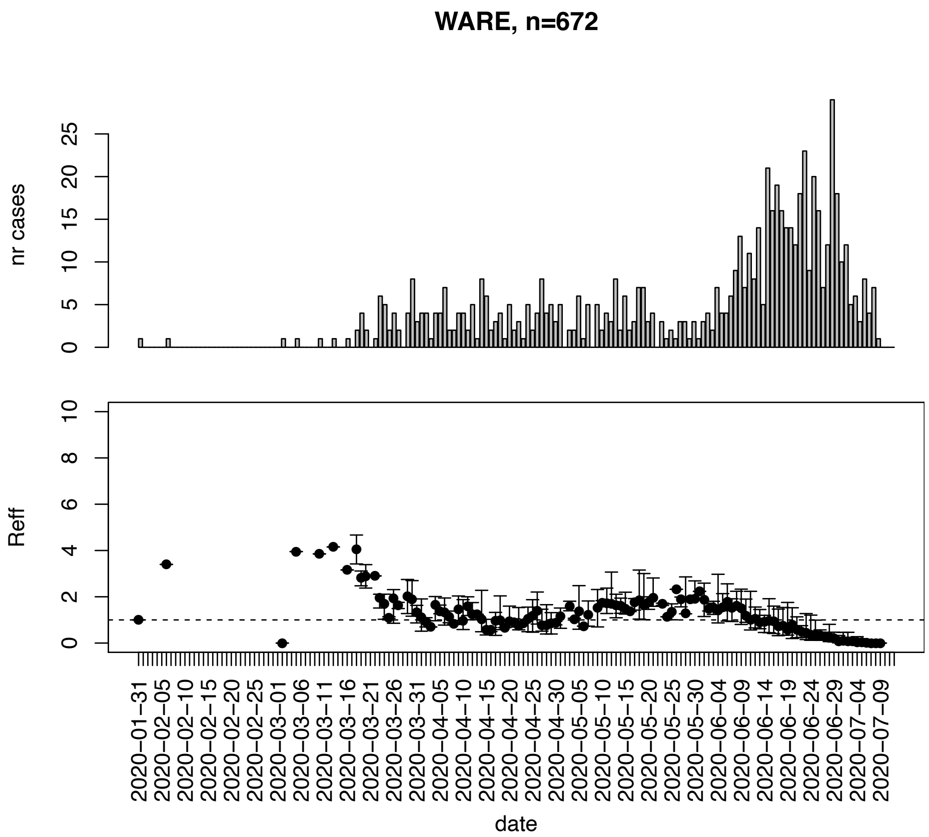

Appendix Figure 158. Epidemic curves and reproduction number estimates until July 13th in Ware county.

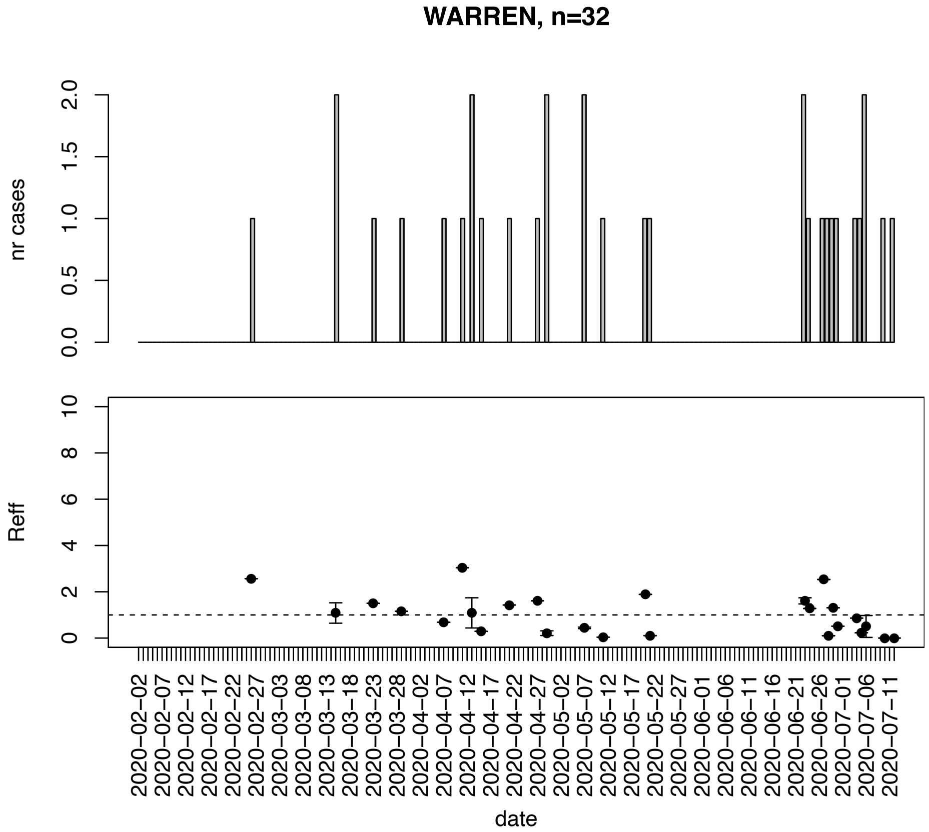

Appendix Figure 159. Epidemic curves and reproduction number estimates until July 13th in Warren county.

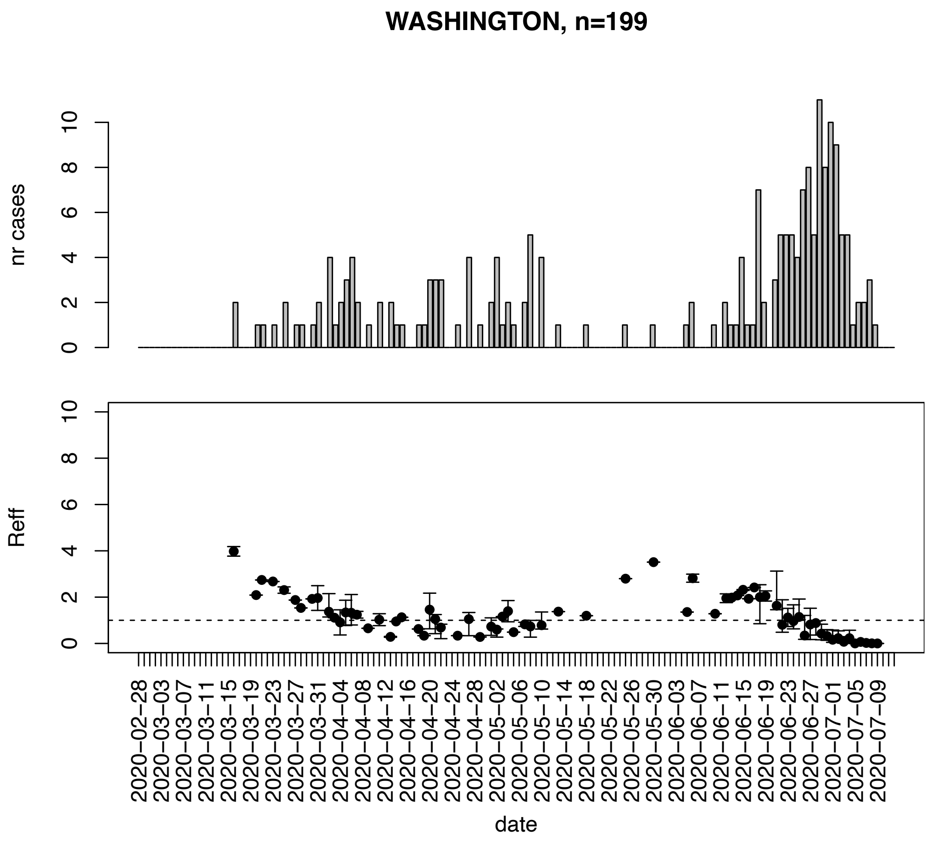

Appendix Figure 160. Epidemic curves and reproduction number estimates until July 13th in Washington county.

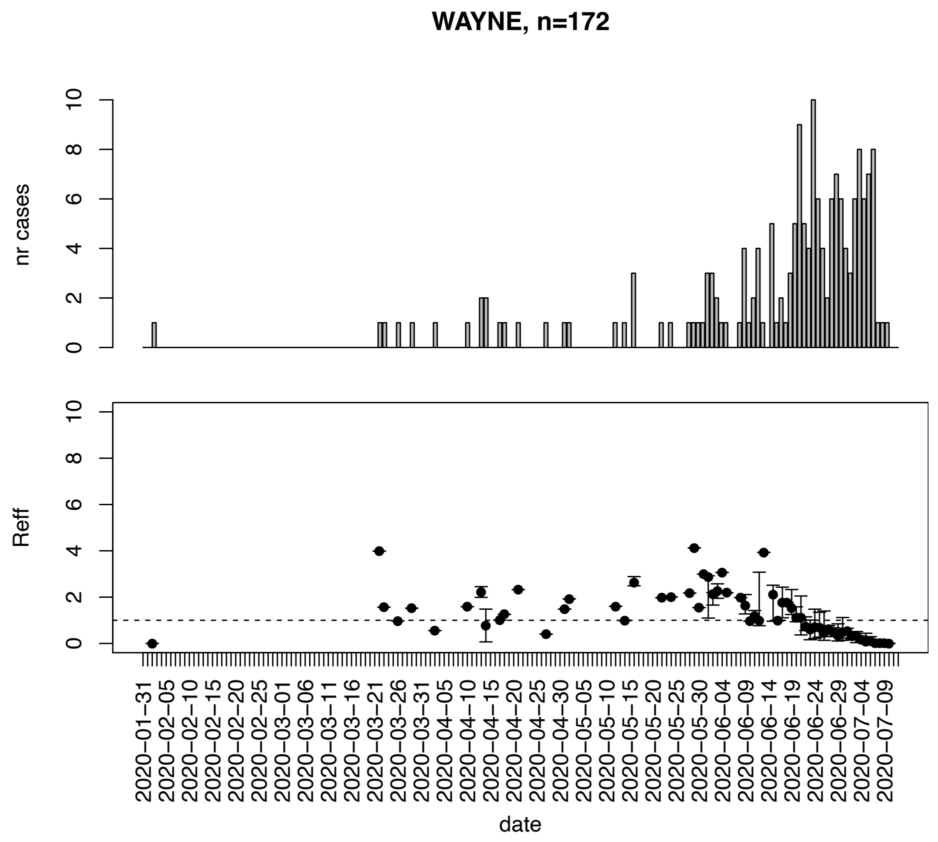

Appendix Figure 161. Epidemic curves and reproduction number estimates until July 13th in Wayne county.

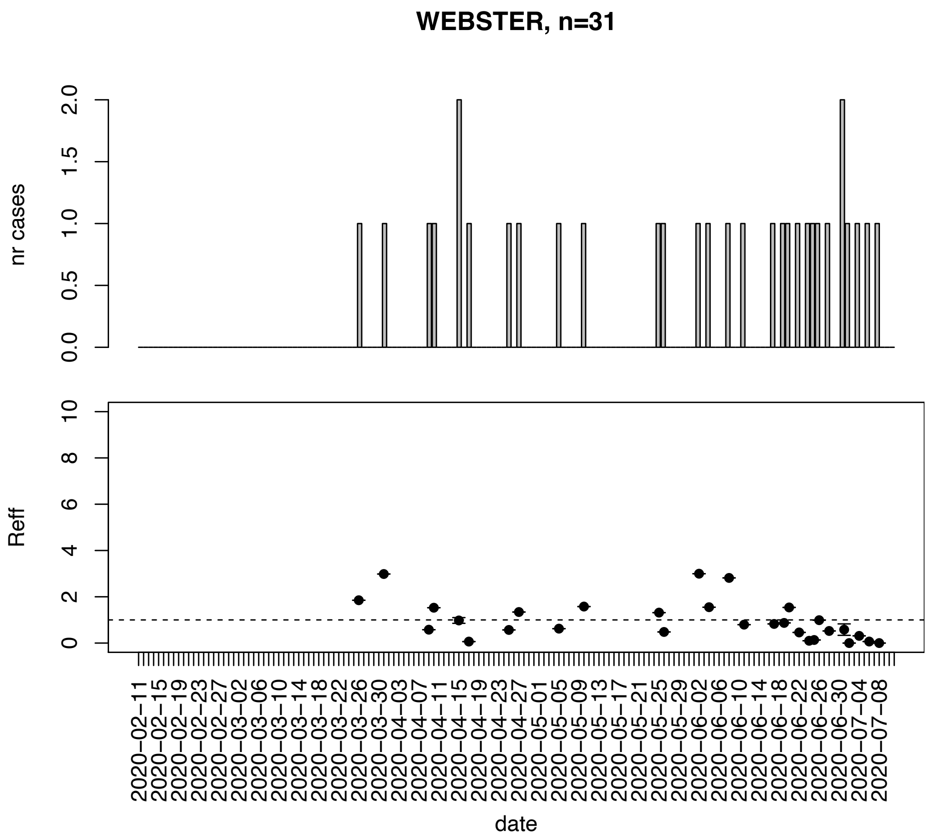

Appendix Figure 162. Epidemic curves and reproduction number estimates until July 13th in Webster county.

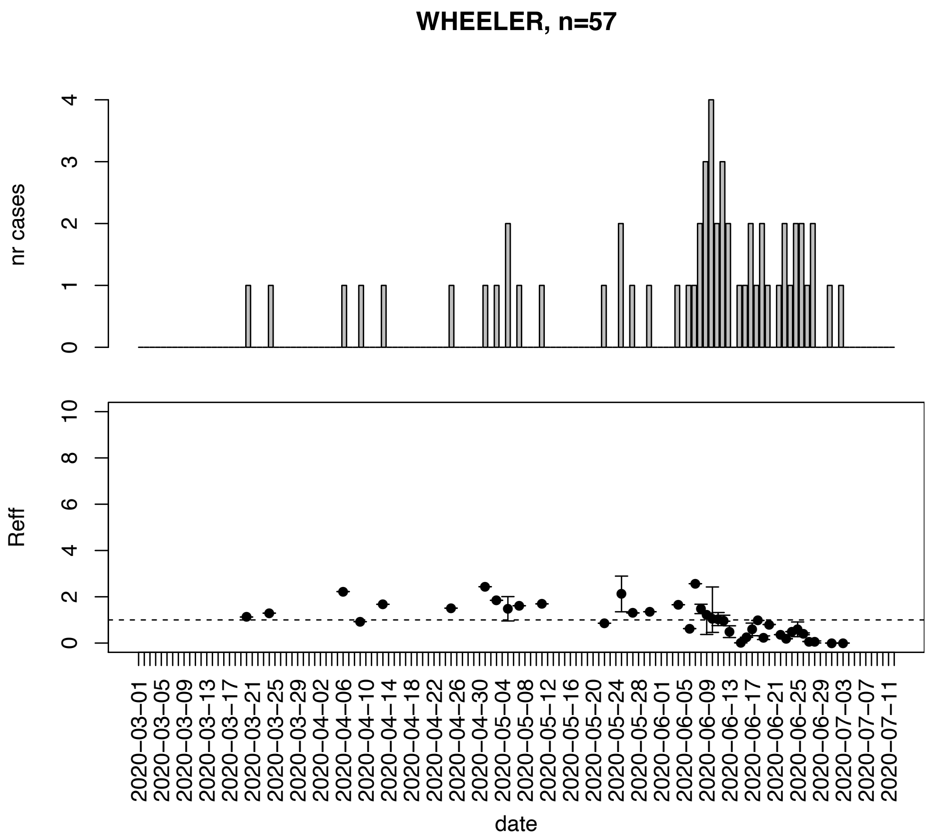

Appendix Figure 163. Epidemic curves and reproduction number estimates until July 13th in Wheeler county.

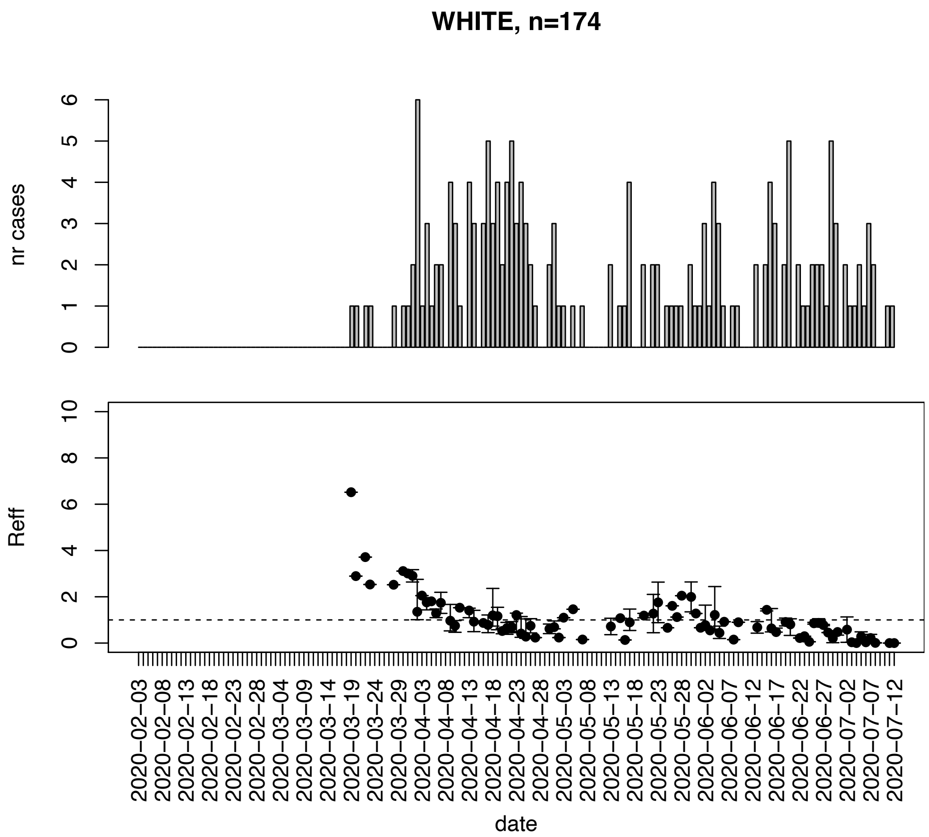

Appendix Figure 164. Epidemic curves and reproduction number estimates until July 13th in White county.

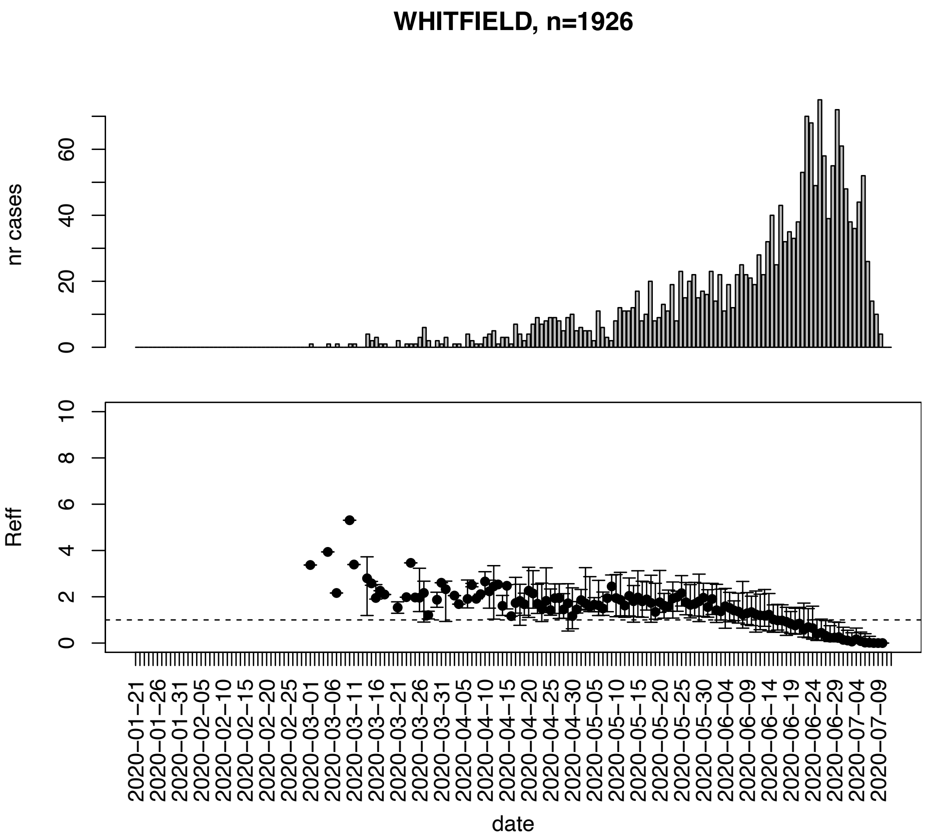

Appendix Figure 165. Epidemic curves and reproduction number estimates until July 13th in Whitfield county.

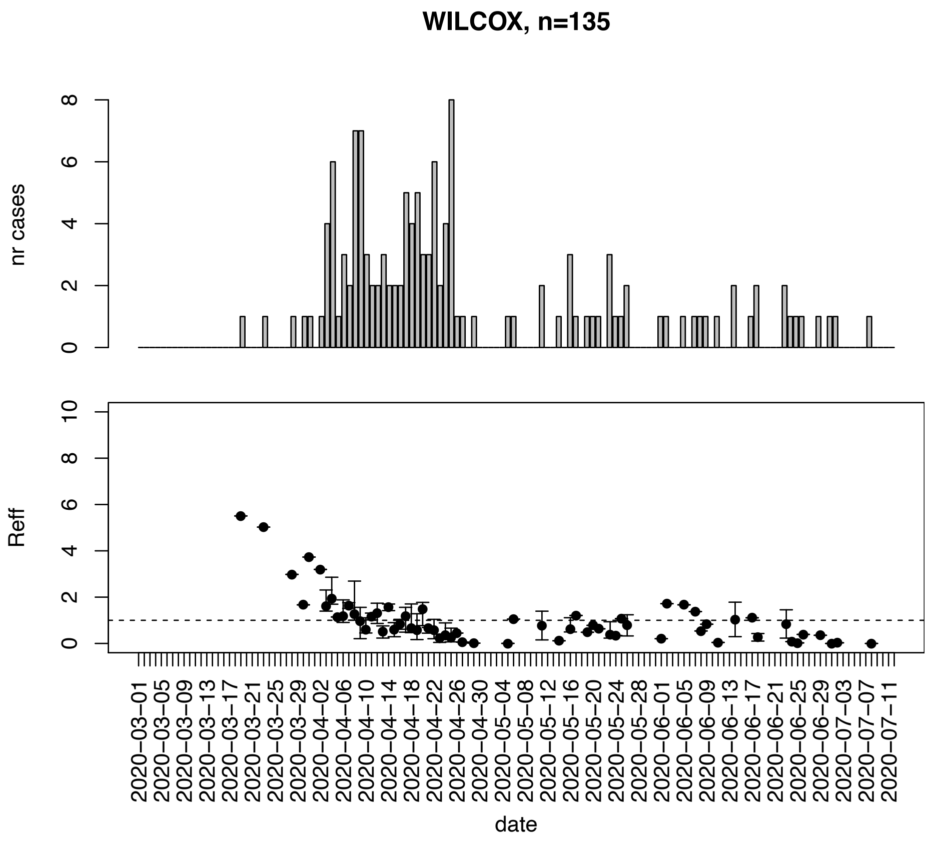

Appendix Figure 166. Epidemic curves and reproduction number estimates until July 13th in Wilcox county.

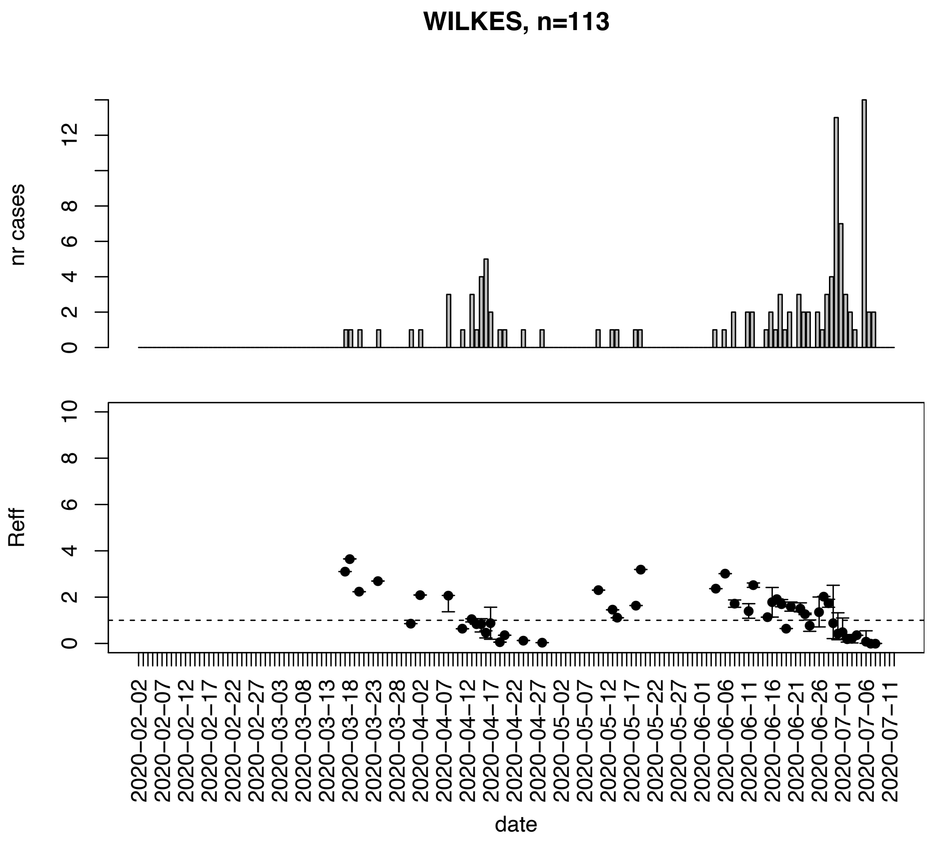

Appendix Figure 167. Epidemic curves and reproduction number estimates until July 13th in Wilkes county.

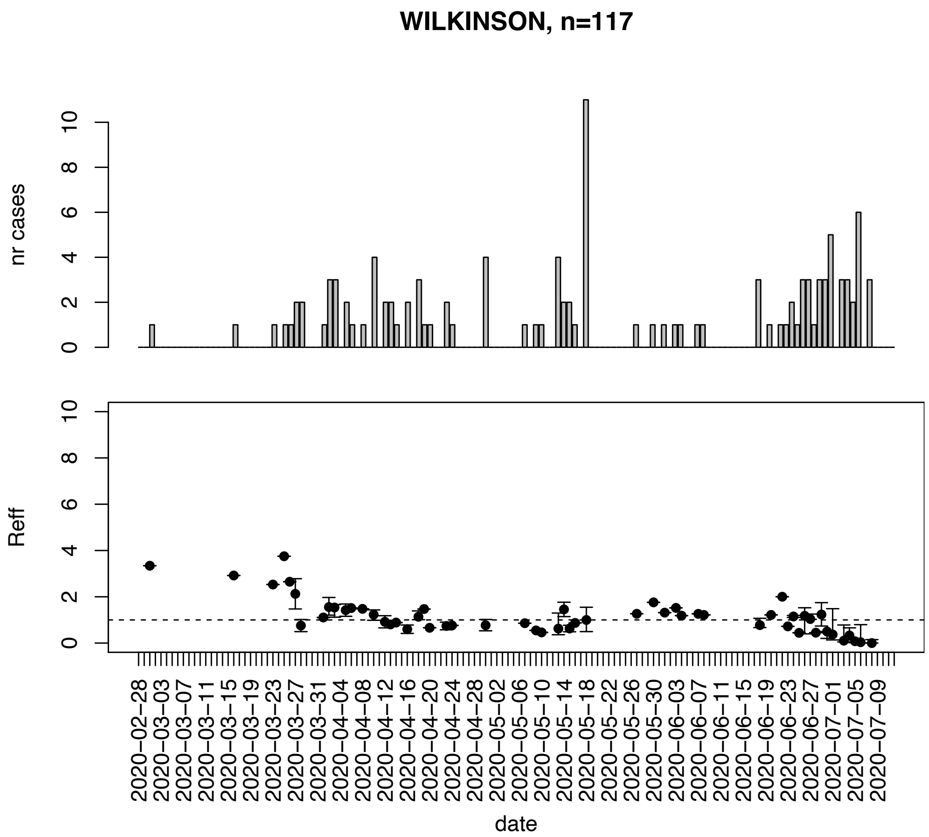

Appendix Figure 168. Epidemic curves and reproduction number estimates until July 13th in Wilkinson county.

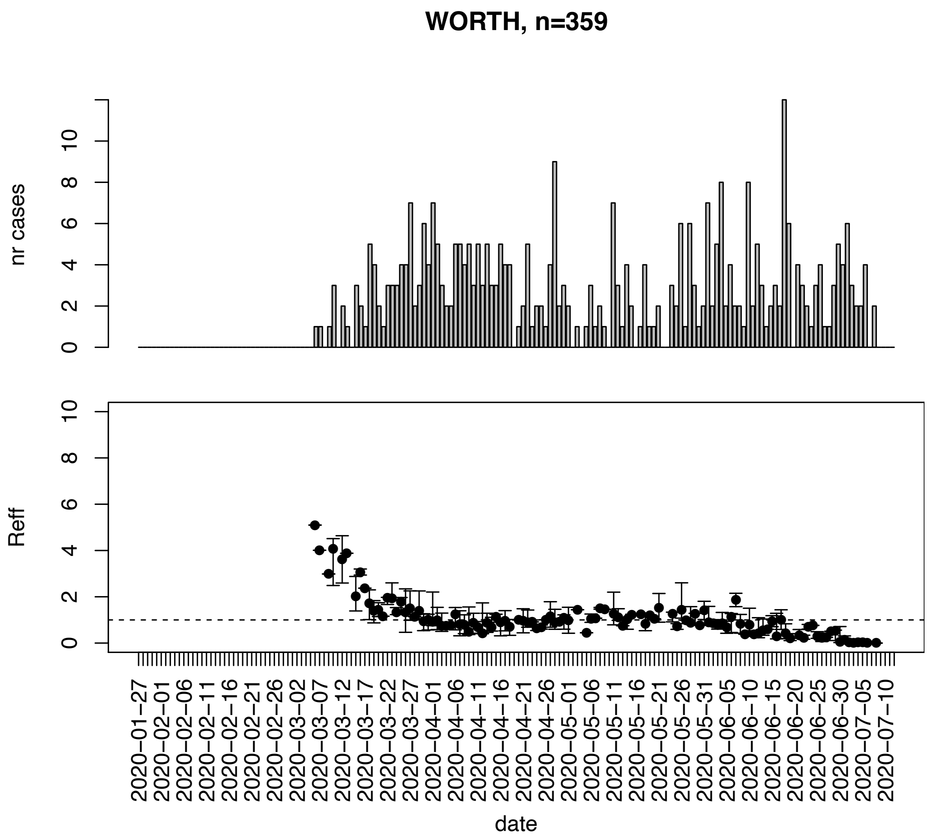

Appendix Figure 169. Epidemic curves and reproduction number estimates until July 13th in Worth county.
